# RESEARCH COMMUNICATIONS OF THE 33^rd^ ECVIM‐CA CONGRESS

**DOI:** 10.1111/jvim.16930

**Published:** 2023-12-05

**21–23 September 2023**

**Centre Convencions International Barcelona**

**Barcelona, Spain**

**Table:** 

**LIST OF ORAL RESEARCH COMMUNICATIONS**			
**ESVC—European Society of Veterinary Cardiology**			
Thursday 21 September			
ESVC‐O‐1	09.00–09.15	Grosso	Echocardiographic evaluation of main pulmonary artery and right pulmonary artery size in dogs with pulmonary hypertension
ESVC‐O‐2	09.15–09.30	Phetariyawong	Left ventricular myocardial protein profile in dogs with myxomatous mitral valve disease and dilated cardiomyopathy
ESVC‐O‐17	09.30–09.45	Vezzosi	The Mitral INsufficiency Echocardiographic (MINE) score in dogs with preclinical myxomatous mitral valve disease
ESVC‐O‐5	10.00–10.15	Sleeper	Gene therapy for the treatment of doberman dilated cardiomyopathy
ESVC‐O‐6	10.15–10.30	Carter	Combined physical examination variables and N‐terminal pro‐brain natriuretic peptide in predicting cardiac disease in asymptomatic cats with murmurs
ESVC‐O‐7	11.20–11.35	Edgerton	Investigation of the indications for and outcome of ambulatory electrocardiography in a referral population of dogs
ESVC‐O‐8	11.35–11.50	Cala	Transvenous closure of patent ductus arteriosus (PDA) with Nit‐Occlud® occlusion system in nine dogs and one cat with a body weight less than 3 kg
ESVC‐O‐9	11.50–12.05	Liu	Tetranectin as a potential biomarker for feline hypertrophic cardiomyopathy
ESVC‐O‐10	12.05–12.20	Pierce	Evaluation of the circulating renin‐angiotensin‐aldosterone system in healthy young dogs and dogs with right‐sided congenital heart disease
ESVC‐O‐11	12.2012.35	Escalda	Assessment of heart rate in dogs with atrial fibrillation: Are two days better than one?
ESVC‐O‐12	12.35–12.50	Rogg	Prevalence and progression of azotemia during treatment of congestive heart failure in cats
Friday 22 September			
ESVC‐O‐13	14.25–14.40	Romito	Clinical efficacy and tolerability of oral amiodarone and sotalol in dogs with tachyarrhythmias
ESVC‐O‐14	14.40–14.55	Goodrich	Incidence of restenosis and associated risk factors in dogs undergoing balloon valvuloplasty for pulmonic stenosis
ESVC‐O‐15	14.55–15.10	Szatmari	Do practicing veterinarians follow the ACVIM consensus guidelines for staging myxomatous mitral valve degeneration in dogs?
ESVC‐O‐16	15.10–15.25	Chang	The Pulmonary hypertension Remodeling/hemodynamic‐Induced Manifestations on Echocardiography (PRIME) score for predicting the severity of canine pulmonary hypertension
ESVC‐O‐3	15.25–15.40	Reimann	Renin‐angiotensin‐aldosterone and phosphodiesterase system gene polymorphisms associated with congestive heart failure in Cavalier King Charles Spaniels with myxomatous mitral valve disease
ESVC‐O‐18	15.40–15.55	Wong	Left atrial rupture secondary to myxomatous mitral valve disease in 33 dogs (2017–2022)
ESVC‐O‐19	16.30–16.45	Van de Watering	Ultrasonographic assessment of abdominal aortic flow to evaluate hemodynamic significance of left‐to‐right shunting patent ductus arteriosus in dogs
ESVC‐O‐20	16.45–17.00	McLaughlin	Timing and patterns of resolution of lung ultrasound B‐lines compared to lung auscultation and respiratory rates in hospitalized dogs with cardiogenic pulmonary edema
ESVC‐O‐21	17.00–17.15	Climent‐Pastor	Use of intravenous nitroglycerin in the treatment of acute left‐sided congestive heart failure in dogs and cats
ESVC‐O‐22	17.15–17.30	Szatmari	How confident are practicing veterinarians in recognizing, and differentiating pathologic from innocent murmurs in puppies?
ESVC‐O‐23	17.30–17.45	Kramer	Implantation of a novel transcatheter mitral valve in a pig heart model
ESVC‐O‐24	17.45–18.00	Boz	End‐diastolic forward flow and restrictive physiology in dogs with pulmonary stenosis
**SCH—Society of Comparative Hepatology**			
Thursday 21 September			
SCH‐O‐1	09.15–09.30	Proksch	Cobalamin status derangements in dogs with portosystemic shunt
SCH‐O‐2	09.30–09.45	Da Silva	Association between hyperlipidaemia and selected cholestatic markers in 75 dogs with suspect acute pancreatitis
SCH‐O‐3	09.45–10.00	Palizzotto	Hepatic AA amyloidosis in shelter cats: clinico‐pathological data and light microscopic findings
SCH‐O‐4	10.00–10.15	BEDEL	Prevalence of canine cholangitis/cholangiohepatitis, a retrospective study based on 263 liver biopsy cases (2013–2021)
SCH‐O‐5	10.15–10.30	Dröes	Point‐of‐care viscoelastometric evaluation of dogs with chronic hepatitis
**ESVNU—European Society of Veterinary Nephrology and Urology**			
Thursday 21 September			
ESVNU‐O‐1	09.00–09.15	Tang	Risk factors and short‐term implications associated with macroscopic nephrocalcinosis in cats with chronic kidney disease
ESVNU‐O‐2	09.15–09.30	Tang	Pilot study evaluating the detection of nephrocalcinosis using ultrasonography in cats with chronic kidney disease
ESVNU‐O‐3	09.30–09.45	Pantaleo	Evaluation of urinary podocin and nephrin as markers of podocyturia in dogs with leishmaniosis
ESVNU‐O‐4	09.45–10.00	Pantaleo	Evaluation of urinary amylase to creatinine ratio as a marker of renal damage in dogs with leishmaniosis undergoing conventional anti‐Leishmania treatment
ESVNU‐O‐5	10.00–10.15	Jiwaganont	Analysis of serum proteomic in cats with polycystic kidney disease‐1 gene mutation
ESVNU‐O‐6	10.15–10.30	Bennett	Progression of chronic kidney disease in cats following subcutaneous ureteral bypass device placement for ureteral obstruction compared to cats with idiopathic chronic kidney disease
ESVNU‐O‐7	11.20–11.35	Mourou	Subcutaneous ureteral bypass placement is associated with a high complication rate but a prolonged survival in cats: a retrospective study of 94 cases (2014–2021)
ESVNU‐O‐8	11.35–11.50	Hardy	‘Interpretating discordant symmetric dimethylarginine and creatinine concentrations in relation to glomerular filtration rate
ESVNU‐O‐9	11.50–12.05	Hawes	The neutrophil‐to‐lymphocyte ratio (NLR) in cats with chronic kidney disease (CKD)
Friday 22 September			
ESVNU‐O‐10	09.00–09.15	Biscop	Diagnostic value of cell cycle arrest biomarkers, tissue inhibitor of metalloproteinase‐2 (TIMP‐2) and insulin‐like growth factor binding protein 7 (IGFBP7), to identify dogs with acute kidney injury
ESVNU‐O‐11	09.15–09.30	Biscop	Diagnostic value of urinary to serum neutrophil gelatinase‐associated lipocalin (NGAL) ratio and fractional excretion of NGAL to differentiate dogs with acute kidney injury from healthy dogs, dogs with chronic kidney disease and critically ill dogs
ESVNU‐O‐12	09.30–09.45	Tagliasacchi	Urinary neutrophil gelatinase‐associated lipocalin (NGAL): A rapid lateral flow test in canine practice
ESVNU‐O‐13	09.45–10.00	Pichard	Validation of a three‐dimensional ultrasound device for non‐invasive bladder volume measurement in dogs and cats
ESVNU‐O‐14	10.00–10.15	Pichard	Genitourinary dysplasia in dogs and cats: A case series
ESVNU‐O‐15	10.15–10.30	Tang	ACVIM Award Winning abstract: A Pilot Study to Identify Plasma Calciprotein Particles in Cats with Chronic Kidney Disease
**ESCG—European Society of Comparative Gastroenterology**			
Friday 22 September			
ESCG‐O‐1	09.00–09.15	Sung	Fecal abundance of *Prevotella copri*, *Ruminococcus gnavus*, and Genus *Collinsella* in cats with chronic enteropathy
ESCG‐O‐2	09.15–09.30	Giordano	Intestinal microbiota and fecal concentrations of fatty acids, sterols, and bile acids in cats with chronic enteropathy
ESCG‐O‐3	09.30–09.45	Lyngby	Association of serum and fecal microRNA profiles in healthy cats and cats with gastrointestinal cancer or chronic inflammatory enteropathy
ESCG‐O‐4	09.45–10.00	Simpson	Serum metabolomic analysis of dogs with chronic enteropathies
ESCG‐O‐5	10.00–10.15	Cagnasso	Investigation of fecal microbiome and metabolome perturbations in dogs with protein‐losing enteropathy
ESCG‐O‐6	10.15–10.30	Vecchiato	Fecal microbial transplantation effect on clinical outcome and fecal microbiota and metabolome in dogs with chronic enteropathy refractory to diet
ESCG‐O‐7	11.20–11.35	Huber	Plasma proteome signatures of canine acute haemorrhagic diarrhoea syndrome (AHDS)
ESCG‐O‐8	11.35–11.50	Pilla	Combined omeprazole and carprofen induced fecal dysbiosis and decreased short‐chain fatty acid production in healthy dogs
ESCG‐O‐9	11.50–12.05	Siegrist	Functional cobalamin deficiency (normal cobalamin with increased methylmalonic acid concentration) is uncommon in dogs
ESCG‐O‐10	12.05–12.20	Tolbert	Intestinal lymphangiectasia is a common finding in healthy soft‐coated wheaten terriers
ESCG‐O‐11	12.20–12.35	Stavroulaki	Early‐life antibiotic exposure and susceptibility to chronic diarrhea during adulthood in cats
ESCG‐O‐12	12.35–12.50	Remmel	Congenital partial colonic agenesis in cats: clinical, biological, diagnostic imaging, endoscopic and histopathologic characterization. A retrospective study of 17 cases
**ESVIM—European Society of Veterinary Internal Medicine**			
Friday 22 September			
ESVIM‐O‐1	14.25–14.40	Bailey	Do dogs with either immune‐mediated polyarthritis or steroid‐responsive meningitis arteritis differ in their presentation and response to treatment compared to dogs with both diseases?
ESVIM‐O‐2	14.40–14.55	Bergum Hjellegjerde	Utility of screening diagnostic imaging in identifying potential triggers of associative immune‐mediated polyarthritis (IMPA) in dogs
ESVIM‐O‐3	14.55–15.10	Shalvey	Evaluation of the utility of haematological ratios as biomarkers in dogs with forebrain disease
ESVIM‐O‐4	15.10–15.25	Leynaud	Epidemiological and hematological variables are useful to specify the underlying disease processes associated with feline non‐regenerative anemia: a retrospective study of 440 cases (2018–2022)
ESVIM‐O‐5	15.25–15.40	Rösch	Pharmacokinetics of orally administered immunosuppressive dosage of cyclosporine A over 10 days in healthy cats
ESVIM‐O‐6	15.40–15.55	Knies	Prevalence of persistent hypertension and situational hypertension in a population of elderly cats in The Netherlands
ESVIM‐O‐7	16.30–16.45	Mochel	A Non‐Invasive Model of Preclinical Metabolic Syndrome to Study the Effects of Sodium Glucose Transporter‐2 Inhibitors in Dogs
ESVIM‐O‐8	16.45–17.00	Niinikoski	Assessment of risk factors for sleep‐disordered breathing in dogs
ESVIM‐O‐9	17.00–17.15	Langton	The comparison of sinonasal clotrimazole distribution using trephination or catheterisation in a cadaver study
ESVIM‐O‐10	17.15–17.30	Fastrès	Lung microbiota assessment in dogs with bronchomalacia
ESVIM‐O‐11	17.30–17.45	Jaffey	Clinical performance of a point‐of‐care Coccidioides antibody test to diagnose pulmonary coccidioidomycosis in dogs
ESVIM‐O‐12	17.45–18.00	Rizzoli	Generation of a comprehensive molecular cell atlas of the healthy canine lung
**ISCAID—International Society for Companion Animal Infectious Diseases**			
Saturday 23 September			
ISCAID‐O‐1	08.00–08.15	Museux	Update on the distribution and seasonal occurrence of vector‐borne diseases in dogs in France
ISCAID‐O‐2	08.15–08.30	Elgueta	Seropositivity to the louping ill flavivirus in dogs in the UK
ISCAID‐O‐3	08.30–08.45	Strobl	Comparison of prognostic factors in feline panleukopenia in juvenile and adult cats
ISCAID‐O‐4	14.25–14.40	Weidinger	Comparison of antibody response after feline panleukopenia virus vaccination in kittens with and without gastrointestinal parasitic infection
ISCAID‐O‐5	14.40–14.55	Evason	Performance of a molecular diagnostic as compared to routine centrifugal‐flotation for fecal gastrointestinal parasite identification
ISCAID‐O‐7	15.10–15.25	Murillo	Leishmania infantum‐specific production of IL‐2 in stimulated blood in dogs with different states of infection
ISCAID‐O‐9	15.25–15.40	Fernández	Severe babesiosis in recently splenectomised dogs due to suspected or confirmed Babesia vulpes infection
ISCAID‐O‐10	16.30–16.45	Almendros	Molecular detection of Babesia spp. in community and privately‐owned cats in Hong Kong
ISCAID‐O‐11	16.45–17.00	Wenderlein	Detection of pathogenic Leptospira spp. serogroups in Europe between 2017 and 2020 applying a gene‐based molecular approach
ISCAID‐O‐12	17.00–17.15	Griebsch	Serological evidence of exposure to Leptospira in dogs in Sydney, New South Wales, Australia
ISCAID‐O‐13	17.15–17.30	Tam	Leptospira in community and privately‐owned cats in Hong Kong: serology and urinary shedding
ISCAID‐O‐14	17.30–17.45	Lutz	Plasma procalcitonin and C‐reactive protein in dogs with suspected bacterial pneumonia or non‐bacterial pulmonary diseases
ISCAID‐O‐15	17.45–18.00	Scahill	Antimicrobial use in 6270 European dogs: a retrospective cohort study (2019–2021)
**ESVONC—European Society of Veterinary Oncology**			
Friday 22 September			
ESVONC‐O‐1	14.25–14.40	Žagar	Biologic behaviour of canine preputial, scrotal and vulvar cutaneous mast cell tumours: a single‐centre retrospective analysis of 102 dogs (2002–2022)
ESVONC‐O‐2	14.40–14.55	Loddo	Canine large granular lymphocyte (LGL) lymphoma: A retrospective study of 42 cases.
ESVONC‐O‐3	14.55–15.10	Lecot	Treatment and outcome of myeloma‐related disorders in cats: a multicentric retrospective study of 50 cases
ESVONC‐O‐4	15.10–15.25	Chalfon	Flow cytometry for detection and quantification of nodal metastasis in dogs with treatment‐naïve firstly occurring cutaneous mast cell tumour
ESVONC‐O‐5	15.25–15.40	Ubiali	Evaluation of programmed death‐ligand 1 expression in canine lymphomas using flow cytometry
ESVONC‐O‐6	15.40–15.55	Lyseight	Flow cytometry expression in canine T cell lymphoma; presentation, prognosis and response to lomustine‐based protocols used in the naïve setting
ESVONC‐O‐7	16.30–16.45	Agnoli	Peripheral blood and bone marrow involvement do not worsen outcome in 50 dogs with nodal peripheral t‐cell lymphoma receiving alkylating‐rich chemotherapy
ESVONC‐O‐8	16.45–17.00	Hawkes	Comparison of first‐line CHOP‐19 and CHOP‐25 in the treatment of canine aggressive peripheral nodal B‐cell lymphomas: a European multicentric retrospective cohort study
ESVONC‐O‐9	17.00–17.15	Treggiari	Incidence of gastrointestinal toxicity and treatment outcome in dogs with multicentric lymphoma receiving doxorubicin or epirubicin as part of a multi‐agent chemotherapy protocol
ESVONC‐O‐10	17.15–17.30	Scheemaeker	Optimization of radioactive iodine uptake in canine thyroid carcinomas using recombinant human thyroid stimulating hormone
ESVONC‐O‐11	17.30–17.45	Guerra	Serum lactate dehydrogenase acts as a potential prognostic biomarker of canine appendicular osteosarcoma
ESVONC‐O‐12	17.45–18.00	Capuano	Investigation and description of circulating MicroRNAs in healthy and T‐cell lymphoma‐bearing dogs: a prospective, two‐arm study
**ESVE—European Society of Veterinary Endocrinology**			
Saturday 23 September			
ESVE‐O‐1	08.00–08.15	Foale	Induction of hepatic insulin production using AAV gene therapy in naturally‐occuring canine diabetes mellitus; a potential future treatment?
ESVE‐O‐2	08.15–08.30	Tardo	Effect of two diets on glycemic variability and glycemic control assessed by flash glucose monitoring system in diabetic dogs
ESVE‐O‐3	08.30–08.45	Niessen	Efficacy and safety of once daily oral sodium‐glucose co‐transporter‐2‐inhibitor velagliflozin compared to twice daily insulin injection therapy in diabetic cats
ESVE‐O‐4	09.00–09.15	Miceli	Increased insulin‐like growth factor 1 concentrations in a population of non‐diabetic cats with overweight/obesity
ESVE‐O‐5	09.15–09.30	Norman	A genome‐wide association study investigating the genetic basis of hyperthyroidism in domestic cats.
ESVE‐O‐6	09.30–09.45	Menzel	Effect of duration of hyperthyroidism and degree of thyroid pathology on recovery of pituitary‐thyroid axis and creatinine concentration in radioiodine‐treated cats followed up over a 1‐year period
ESVE‐O‐7	09.45–10.00	Williams	Survival times of radioiodine treated hyperthyroid cats with and without iatrogenic hypothyroidism and investigation of effect of levothyroxine supplementation on survival time
ESVE‐O‐8	10.00–10.15	Travail	Serum parathyroid hormone concentration as a predictor of post‐operative hypocalcemia in dogs diagnosed of primary hyperparathyroidism and treated with parathyroidectomy
ESVE‐O‐9	10.15–10.30	van den Berg	Metabolite profiling in canine pheochromocytomas, cortisol‐secreting adrenocortical tumors, and normal adrenals
ESVE‐O‐10	11.20–11.35	Golinelli	Addition of cabergoline to trilostane treatment for dogs with pituitary‐dependent hypercortisolism
ESVE‐O‐11	11.35–11.50	Jankovic	Electrophoretic patterns of proteinuria in dogs with Cushing's syndrome and glomerular disease
ESVE‐O‐12	11.50–12.05	Fracassi	Comparison of urinary cortisol and basal serum cortisol as a screening test for hypoadrenocorticism in dogs
ESVE‐O‐13	12.05–12.20	Roberts	Clinical findings, treatment and outcomes in cats with spontaneous hypoadrenocorticism: 41 cases
ESVE‐O‐14	12.20–12.35	Watson	Identification of somatic mutations in feline adrenal tumours causing primary hyperaldosteronism
ESVE‐O‐15	12.35–12.50	Chamagne	Evaluation of serum steroid profile in healthy cats, cats with chronic kidney disease and cats with primary hyperaldosteronism
**ESVCN—European Society of Veterinary Comparative & Nutrition**			
Saturday 23 September			
ESVCN‐O‐1	15.10–15.25	Dobenecker	Short‐term oral intake of inorganic phosphate additives causes an increase of FGF23 in cats and dogs
ESVCN‐O‐2	15.25–15.40	Blanchard	Beneficial response to a very low‐carbohydrate home‐cooked diet in dogs with refractory chronic enteropathy: pilot study of 25 cases
ESVCN‐O‐3	15.40–15.55	Pignataro	Follow‐up in 167 homemade diets for maintenance and pathologic conditions in dog
ESVCN‐O‐4	15.55–16.10	Bela	Effects of Lactobacillus reuteri NBF 2 DSM 32264 supplementation on healthy Certosino cat performance

**LIST OF POSTER RESEARCH COMMUNICATIONS**		
		
**ESVC—European Society of Veterinary Cardiology**		
ESVC‐P‐1	Kiss	Cilostazol as medical treatment of bradycardia of cardiac origin in dogs
ESVC‐P‐2	Alibrandi	Smartphone‐based 6‐lead ECG: a new device for electrocardiographic recording in dogs
ESVC‐P‐3	Costa‐Rodríguez	Correlation of serum N‐terminal pro B‐type natriuretic peptide with pulmonary hypertension in heartworm‐infected dogs during adulticide treatment
ESVC‐P‐4	Kempker	The good, the bad and the breeder**—**Development of genetic HCM‐variants in cat populations from routine testing in the last 10 years
ESVC‐P‐6	Baisan	Assessment of precordial R‐peak time in dogs with left sided volume overload secondary to myxomatous mitral valve disease and dilated cardiomyopathy
ESVC‐P‐7	Cimerman	Inflammatory and immune variables as predictors of survival in dogs with myxomatous mitral valve disease
ESVC‐P‐8	Romito	Clinical findings and outcome in 27 cats with transient myocardial thickening
ESVC‐P‐9	Falcón Cordón	Associations between thoracic radiographic changes and severity of pulmonary hypertension diagnosed via Doppler echocardiography in dogs with heartworm disease (Dirofilaria immitis)
ESVC‐P‐10	Georgiades	Clinical presentation and survival in dogs with acquired atrial septal defect secondary to myxomatous mitral valve disease
ESVC‐P‐11	Davis	Echocardiographic classification of dogs with aortic stenosis: potential utility of a novel staging system
ESVC‐P‐12	Sakarin	Serum proteomic profiles in dogs affected with pulmonary hypertension secondary to degenerative mitral valve disease
ESVC‐P‐13	Välimäki	The impact of intravenous medetomidine and vatinoxan on echocardiographic evaluation of dogs with ACVIM stage B1 mitral valve disease
ESVC‐P‐14	Santana	Percutaneous closure of patent ductus arteriosus with the Vet‐PDA Occluder® device in dog
ESVC‐P‐15	Szatmari	How do practicing veterinarians treat stage B and C myxomatous mitral valve degeneration in dogs?
ESVC‐P‐16	Van Renterghem	Ratio of tricuspid annular plane systolic excursion on systolic pulmonary arterial pressure as a prognostic factor in canine pre‐capillary pulmonary hypertension
ESVC‐P‐17	Panprom	The effect of anesthesia drug on cardiac function parameters in cats
ESVC‐P‐18	Chang	Aortic insufficiency in dogs and cats: A retrospective study of clinical characteristics, echocardiographic findings, and prognosis in non‐infectious and non‐congenital cases
ESVC‐P‐19	Di Grado	Respiratory rate and breathing pattern in dogs and cats admitted to the intensive care unit at two small animal hospitals in Sweden
ESVC‐P‐20	Lee	Temporal cardiomegaly induced by non‐cardiogenic respiratory distress in dogs with myxomatous mitral valve disease
ESVC‐P‐21	Legrand	Association of decreased bodyweight‐independent tricuspid annular plane systolic excursion (TAPSE) measured in M‐mode with clinical signs of heart failure and cardiac death in dogs with pre‐capillary pulmonary hypertension
ESVC‐P‐22	Baisan	Respiratory sinus arrhythmia dynamics in dogs with various classes of naturally occurring myxomatous mitral valve disease
ESVC‐P‐23	Sukumolanan	Proteomics and coagulation markers in cats with asymptomatic and symptomatic hypertrophic cardiomyopathy
ESVC‐P‐24	Matos Rivero	Clinical and echocardiographic findings in dogs with heartworm caval syndrome
ESVC‐P‐25	Iwasa	The prognostic value of circulating cardiac and renal biomarkers in dogs with myxomatous mitral valve disease
**SCH—Society of Comparative Hepatology**		
SCH‐P‐1	Cattaneo	A retrospective study investigating the grade and temporal progression of feline gall bladder sludge and association with concurrent disease
SCH‐P‐2	Jolly‐Frahija	Biliary hamartoma: a case series in small animals
**ESVNU—European Society of Veterinary Nephrology and Urology**		
ESVNU‐P‐1	Gil	Capillary electrophoresis in urine from healthy cats: comparison with canine patterns
ESVNU‐P‐2	Lea	One‐step, non‐surgical placement of permanent low‐profile cystostomy tubes in nine dogs
ESVNU‐P‐3	Liu	Comparison between urine protein‐creatinine ratios of samples obtained from healthy cats at home and in hospital
ESVNU‐P‐4	Bestwick	The prevalence of disease in diagnosis‐naïve older cats presented for health screening to two UK‐based veterinary clinics
ESVNU‐P‐5	Jaturanratsamee	Ultrasound assessments of cats associated with polycystic kidney disease caused by feline PKD1 genetic mutation
ESVNU‐P‐6	Schäfer	Association of Fibroblast growth factor‐23 (FGF‐23) with parameters of renal dysfunction in dogs and cats
ESVNU‐P‐7	Daza	Alterations in canine magnesium levels caused by Leishmania infantum‐induced chronic renal disease
ESVNU‐P‐8	Vigouroux	Effect of subcutaneous bypass (SUB) on proteinuria in cats with ureteral obstruction.
ESVNU‐P‐9	Daza	Development of Cardiorenal Syndrome type IV (Chronic renocardiac syndrome) in cats attended to a nephrology service
**ESCG—European Society of Comparative Gastroenterology**		
ESCG‐P‐1	Chrysovergi	Serial evaluation of the effects of antibiotic use on the intestinal microbiome in kittens using the feline dysbiosis index
ESCG‐P‐2	Moraiti	Serial measurement of serum specific pancreatic lipase and trypsin‐like immunoreactivity concentrations in dogs and cats with diabetes mellitus
ESCG‐P‐3	Holmberg	Comparative study of video capsule endoscopy findings in dogs with chronic enteropathy and in healthy dogs
ESCG‐P‐4	Mathews	Characterization of the intestinal microbiome of dogs with inflammatory brain disease compared to dogs with non‐inflammatory brain disease and healthy control dogs
ESCG‐P‐5	Díaz‐Regañón	Interrelation between microbiota and intraepithelial and lamina propria duodenal T‐lymphocytes in dogs with inflammatory bowel disease
ESCG‐P‐6	Karra	Evaluation of fecal microbiota transplantation as adjunct management of cats with chronic enteropathies in a controlled, blinded, randomized clinical trial
ESCG‐P‐7	Ludvigsson	Chronic enteropathy and emotional health in dogs—a prospective comparative pilot study
ESCG‐P‐8	Peyron	Overview of faecal pathogens isolated from faeces of French young cats and dogs using PCR technique over a year (2022)
ESCG‐P‐9	Karra	Serial measurement of serum‐specific pancreatic lipase and trypsin‐like immunoreactivity concentrations in cats during treatment for chronic enteropathy
ESCG‐P‐10	Tang	Relationship between 1,2‐O‐dilauryl‐rac‐glycero glutaric acid‐(6’‐methylresorufin) ester (DGGR) lipase activity and clinical signs in dogs with chronic gastrointestinal signs over time
ESCG‐P‐11	Cocci	Prevalence of duodenal lymphangiectasia in French Bulldogs with obstructive airway syndrome undergoing endoscopic examination of the respiratory tract and upper digestive tract: retrospective study in 29 cases (2021–2022)
ESCG‐P‐12	Spalla	Holter analysis in dogs with acute and chronic diarrhea
ESCG‐P‐13	Keller	Psyllium husk powder increases defecation frequency and fecal score, bulk and moisture in healthy cats
ESCG‐P‐14	Hanifeh	Fecal microbiota transplantation in dogs with tylosin responsive enteropathy—A proof of concept study
ESCG‐P‐15	Signorelli	Characterization of underlying causes, treatment approaches, short and long‐term outcome in young cats with chronic recurrent gastrointestinal signs
ESCG‐P‐16	Correa Lopes	Effect of various storage conditions on bacteria viability in fecal microbiota transplantation preparations
ESCG‐P‐17	Méric	Fecal proteolytic activities in dogs with chronic inflammatory enteropathies
ESCG‐P‐18	Smith	Protein‐losing enteropathy in Pugs: a different form of disease?
ESCG‐P‐19	Hawes	Mortality rate and associated characteristics prior to hospital discharge in dogs with protein‐losing enteropathy
**ESVONC—European Society of Veterinary Oncology**		
ESVONC‐P‐1	Kleiter	Implementation of an Elekta Infinity linear accelerator with advanced treatment technology
ESVONC‐P‐2	Marceglia	Platelet count changes in cats with lymphoma following repeated vincristine administration
ESVONC‐P‐3	Einhorn	Outcomes and prognostic factors in canine T cell lymphoma treated with lomustine, vincristine, cyclophosphamide, and prednisone chemotherapy
ESVONC‐P‐5	Treggiari	Retrospective evaluation of lung lobectomy and adjuvant treatment for primary pulmonary carcinoma in dogs: a multi‐institutional study
ESVONC‐P‐6	Hanael	Thermographic assessment of soft tissue sarcomas in dogs
ESVONC‐P‐7	Odatzoglou	Feasibility of using flow cytometric analysis of Ki‐67 expression in canine cutaneous mast cell tumours**—**a pilot study
ESVONC‐P‐8	Lollo	Round cell tumours and the feline tarsus: a fatal attraction
ESVONC‐P‐9	Ramos	Surgery and Adjuvant Electrochemotherapy Compared to Surgery Alone for Treatment of Canine Soft Tissue Sarcomas
ESVONC‐P‐10	Hawkes	Evaluation of staging in 504 dogs with aggressive peripheral nodal B cell lymphoma across 16 Oncology referral centres
ESVONC‐P‐11	Ghisoni	A retrospective clinico‐pathologic study of 37 dogs with urethral carcinoma (2012–2022)
ESVONC‐P‐12	Larsen	A retrospective study of clinical outcome in response to short chemotherapy protocol for feline intermediate‐ and large cell lymphoma
ESVONC‐P‐13	Mulder	Erythrocyte and platelet characteristics as indicators of metastasis in canine carcinomas and sarcomas
ESVONC‐P‐14	Del Castillo	Is chemotherapy useful in feline mammary tumours?
ESVONC‐P‐15	Garcia‐de la Virgen	Toceranib (Palladia®) as a first line therapy in macroscopic canine oral squamous cell carcinoma: a case series
ESVONC‐P‐16	Vázquez‐Martínez	Evaluation of the CLOP protocol as induction in canine non‐indolent T‐cell lymphoma
**ESVE—European Society of Veterinary Endocrinology**		
ESVE‐P‐1	Galdhar	Radioimmunoassay (RIA) enabled thyroid profile in healthy dogs: effect of age, sex, breed, and diet
ESVE‐P‐2	Bennaim	Machine‐learning as a diagnostic tool for naturally occurring hypercortisolism in dogs
ESVE‐P‐3	Reeves	A prospective study to examine the natural variation of interstitial glucose levels in healthy adult dogs utilising a continuous glucose monitoring system
ESVE‐P‐4	Guse	Signalment, clinicopathological findings, management practices and occurrence of comorbidities in cats with diabetes mellitus in Germany: prospective study of 148 cases
ESVE‐P‐5	Egbert	Thyroid hormone concentrations in healthy Irish Setters and Rhodesian Ridgeback dogs: a diagnostic comparison
ESVE‐P‐6	Carvalho	Interpretation of screening tests for hypercortisolism by first‐opinion western european veterinary surgeons
ESVE‐P‐8	Stadler	Perceptions of first‐opinion veterinarians and owners in Switzerland about treatment of feline hyperthyroidism
ESVE‐P‐9	Kurtz	Urinary aldosterone to creatinine ratio and fractional excretion of sodium and potassium in healthy cats, cats with primary hyperaldosteronism and cats with secondary hyperaldosteronism
ESVE‐P‐10	Blunschi	The effect of treatment modality on changes in quality of life in hyperthyroid cats
ESVE‐P‐11	López‐Gómez	Hypercortisolism in cats in relation with diabetes mellitus: clinical and laboratory abnormalities, blood pressure and survival under trilostane treatment
ESVE‐P‐12	Martineau	Addison detect: a machine learning predictive model web application to assess canine hypoadrenocorticism likelihood using signalment and routine bloodwork results
ESVE‐P‐13	Norman	Longitudinal evaluation of blood pressure in cats treated for hyperthyroidism
ESVE‐P‐14	Miceli	Feline hypersomatotropism without concurrent diabetes mellitus: 21 cases (2014–2023)
ESVE‐P‐15	Rothlin Zachrisson	Stress or success—hair cortisol concentrations in relation to cat characteristics, health and reproductive status in cats
ESVE‐P‐16	Del Baldo	Evaluation of urinary corticoid‐to‐creatinine ratio using a canine‐specific chemiluminescent cortisol assay (immulite 2000) in the diagnosis of canine hypercortisolism
ESVE‐P‐17	Slon	Circulating concentrations of betatrophin in dogs with diabetes mellitus
ESVE‐P‐18	Rey Amunategui	Comparison of symmetric dimethylarginine, plasma aldosterone concentration, renin activity and systolic blood pressure between diabetic and non‐diabetic cats with hypersomatotropism
ESVE‐P‐19	Rey Amunategui	Increased insulin‐like growth factor 1 concentrations in a population of non‐diabetic cats with chronic kidney disease
ESVE‐P‐20	Allenspach	Healthy canine and human derived pituitary gland organoids exhibit comparable cellular complexity and hormone secretion
ESVE‐P‐21	Strey	Canine hypoadrenocorticism—evaluation of aldosterone concentration and thyroid autoantibodies in dogs
ESVE‐P‐22	Nagata	Urinary steroid profiling using liquid chromatography‐tandem mass spectrometry for the diagnosis of canine Cushing's syndrome
ESVE‐P‐23	Hulsebosch	Normal thyroid values in 3‐week and 8‐week‐old healthy kittens
ESVE‐P‐24	Langlois	Desoxycorticosterone pivalate (DOCP) pharmacodynamics in dogs with hypoadrenocorticism
**ISCAID—International Society for Companion Animal Infectious Diseases**		
ISCAID‐P‐1	Breu	Seroprevalence of toxoplasma‐specific antibodies in European cats suspected of toxoplasmosis
ISCAID‐P‐2	Khor	Serum cardiac troponin I as an indicator of myocarditis in dogs diagnosed with leptospirosis
ISCAID‐P‐3	Pavlin	Dynamics of selected inflammatory markers during treatment of feline infectious peritonitis
ISCAID‐P‐4	Boontuboon	The association between immune‐mediated thrombocytopenia diagnosed by flow cytometry and canine monocytic ehrlichiosis
ISCAID‐P‐5	Fastrès	Impact of DNA extraction methods and culture‐independent approaches on canine lung mycobiota analysis
ISCAID‐P‐6	Thomsen	Characterisation of Clinical Bleeding Diathesis, and Laboratory Haemostatic Aberrations and Survival in 180 Dogs (2005–2019) Naturally Infected with Angiostrongylus vasorum
ISCAID‐P‐8	Kehl	Resistance of Leishmania infantum to allopurinol in dogs in Europe—a digital droplet PCR assay for quantifying S‐adenosylmethionine synthetase (METK) gene copy number
ISCAID‐P‐10	Roels	Prevalence and risk factors for Mycoplasma spp. positivity in cat blood donor units from Portugal, Spain and Belgium in 2022: retrospective study on 7573 blood donations from 4121 healthy donor cats
ISCAID‐P‐11	Wilczek	A multivalent vaccine against Lyme borreliosis applied to dogs using different vaccination schedules: characterization of the humoral immune response
ISCAID‐P‐12	Tam	Leptospira in dogs in Hong Kong: serology and urinary shedding
ISCAID‐P‐13	Murillo	Leishmania infantum specific IL‐2 production after oclacitinib and ciclosporin treatments in ex vivo stimulated canine blood
ISCAID‐P‐14	GONZÁLEZ	Evaluation of complementary stem cell therapy in the treatment of kidney disease secondary to canine leishmaniasis
ISCAID‐P‐15	Tangmahakul	Advantage of matrix‐assisted laser desorption/ionization time‐of‐flight (MALDI‐TOF) mass spectrometry for bacterial identification in cats with pyothorax
ISCAID‐P‐16	Jiménez	The effect of oclacitinib and ciclosporin A on IL‐17a production against Leishmania infantum stimulated canine blood
ISCAID‐P‐17	Gori	Serum amino acid profile in canine sepsis and its relationship with survival
ISCAID‐P‐18	Mateu	Comparison of Real‐Time PCR and culture of bronchoalveolar lavage fluid in dogs for the detection of Bordetella bronchiseptica infection: a retrospective study of 12 cases
ISCAID‐P‐19	García Rodríguez	Co‐infections and risk factors in stray cats in Gran Canaria (Spain): Dirofilaria immitis, Bartonella henselae, feline leukemia and feline immunodeficiency
**ESVIM—European Society of Veterinary Internal Medicine**		
ESVIM‐P‐1	García‐Guasch	Characterisation of brachycephalic obstructive airway syndrome in cats using barometric whole‐body plethysmography
ESVIM‐P‐2	PETITPRE	Dorsomedial nasopharyngeal masses with a benign appearance in dogs: a retrospective study of 99 cases (2019–2022)
ESVIM‐P‐3	Grobman	Incidence and characterization of aerophagia in dogs using videofluoroscopic swallow studies
ESVIM‐P‐4	Chen	Spectrographic analysis of lung sound for identifying visual characteristics of crackles in dogs
ESVIM‐P‐5	Bottero	Clinical evaluation and endoscopic classification of gastrointestinal findings in 220 dogs with brachicephalic syndrome
ESVIM‐P‐6	Geisen	Heinz Body Formation in 13 Multimorbid Dogs following Metamizole Administration
ESVIM‐P‐7	Schortz	Root cause analysis of medication‐related incidents in small animal veterinary practices: An Analysis of Incident Reports
ESVIM‐P‐8	Jaffey	Effects of short‐term oral 25‐hydroxyvitamin D supplementation on leukocyte cytokine production in healthy dogs
ESVIM‐P‐9	Viitanen	Serum procalcitonin as a diagnostic biomarker in dogs with bacterial respiratory diseases
ESVIM‐P‐11	SU	Clinical efficacy of feline recombinant interferon‐omega as an additional agent in refractory feline chronic gingivostomatitis
ESVIM‐P‐12	Doulidis	Determination of the plasma proteome of healthy dogs using Liquid Chromatography—Mass spectrometry
ESVIM‐P‐13	Ostronic	Pharmacodynamics of Standard and Low Dose Once Daily Esomeprazole in Healthy, Medium to Large Breed Dogs
ESVIM‐P‐16	Gaspar	Current epidemiology and therapeutic trends of feline chronic rhinitis—a retrospective study
ESVIM‐P‐17	Carvalho	Owners’ perspective about the use of mirtazapine transdermal ointment in cats—a survey‐based study
ESVIM‐P‐18	López Bailén	Outcome and prognostic factors in dogs diagnosed with immune‐mediated thrombocytopenia in Ireland (2009–2020)
ESVIM‐P‐19	Pimenta da Silva	Evaluation of association between hemoparasite infection risk and blood phenotype, gender and breed in potential feline blood donors of portugal and spain
ESVIM‐P‐20	Álvarez	Prevalence, age and disease associations in Yorkshire terriers with increased blood urea nitrogen
ESVIM‐P‐22	Mateu	Clinical and imaging findings, bacterial isolates and antimicrobial resistance in 14 dogs with tracheal and/or bronchial collapse: a retrospective study of 14 cases
ESVIM‐P‐23	Boot	Clinical manifestations, endoscopic findings and outcome of palatine tonsil crypt foreign bodies in dogs: 7 cases (2020–2022)

*The European College of Veterinary Internal Medicine – Companion Animals (ECVIM‐CA) Congress and the Journal of Veterinary Internal Medicine (JVIM) are not responsible for the content or dosage recommendations in the abstracts. The abstracts are not peer reviewed before publication. The opinions expressed in the abstracts are those of the author(s) and may not represent the views or position of the ECVIM‐CA. The authors are solely responsible for the content of the abstracts*.

## ESCG‐O‐1

### ESCG—European Society of Comparative Gastroenterology

#### Fecal abundance of *Prevotella copri*, *Ruminococcus gnavus*, and Genus *Collinsella* in cats with chronic enteropathy

##### CH Sung^1^, CC Chen^1^, S Marsilio^2^, B Chow^3,4^, KA Zornow^5^, JE Slovak^5^, R Pilla^1^, JA Lidbury^1^, JM Steiner^1^, SL Hill^3,6^, J Suchodolski^1^

###### ^1^Gastrointestinal Laboratory, Department of Small Animal Clinical Sciences, Texas A&M University, College Station, TX, USA; ^2^UC Davis School of Veterinary Medicine, Department of Veterinary Medicine and Epidemiology, University of California‐Davis, Davis, CA, USA; ^3^Veterinary Specialty Hospital, San Diego, CA, USA; ^4^VCA Animal Specialty and Emergency Center, Los Angeles, CA USA; ^5^Animal Medical Center, New York, NY, USA; ^6^Flagstaff Veterinary Internal Medicine Consulting, Flagstaff, Arizona, USA

Previous studies showed that *Prevotella copri*, *Ruminococcus gnavus* and genus *Collinsella* were found to be the most prevalent bacterial species in fecal samples of healthy cats and cats with chronic enteropathy (CE), accounting for up to 45%, 16%, and 40%, respectively, based on 16S rRNA sequencing and metagenomic DNA shotgun sequencing analysis. *Prevotella* and *Collinsella* are known to be the commensal gut bacteria in humans, with *P. copri* being the predominant species among the genus *Prevotella*. Human patients with inflammatory bowel disease (IBD) have been reported to have an expansion of *Prevotella*, *Collinsella* and *R. gnavus*, compared to healthy patients. *R. gnavus* is a mucin‐degrading bacterium associated with bile acid metabolism, converting primary bile acids into iso‐type bile acids and preventing the formation of secondary bile acids. However, the abundance of these bacterial groups in cats has not been determined using targeted quantitative methods. The aim of this study was to assess the fecal abundances of *P. copri*, *R. gnavus*, and *Collinsella* in cats with CE and in healthy control cats. This study included fecal samples from 71 cats with CE and 52 healthy cats from previous studies. Fecal abundances of *P. copri*, *R. gnavus* and genus *Collinsella* were measured using quantitative PCR. Mann‐Whitney *U* tests were used to compare the abundances of these bacteria between groups. Statistical significance was set at *P* < .05. The results showed that the abundance of *P. copri* was significantly lower (*P* < .0001) in cats with CE (log DNA, median[range]: 11.0 [9.5–15.4]) than in healthy control cats (13.5 [10.1–16.3]). The fecal abundance of *R. gnavus* was also significantly lower (*P* < .0001) in cats with CE (6.9 [6.1–11.6]) than in healthy control cats (9.9 [6.1–12.0]). However, the abundance of genus *Collinsella* did not differ between groups (*P* = .08). In conclusion, this study revealed decreased fecal abundances of *P. copri* and *R. gnavus* in cats with CE, which stands in contrast to findings in humans with IBD.

**Disclosures**

Chi‐Hsuan Sung, Chih‐Chun Chen, Rachel Pilla, Jonathan A Lidbury, Jörg M Steiner and Jan S Suchodolski are employed by the Gastrointestinal Laboratory at Texas A&M University, which provides assays for intestinal function and microbiota analysis on a fee‐for‐service basis.

## ESCG‐O‐2

### ESCG—European Society of Comparative Gastroenterology

#### Intestinal microbiota and fecal concentrations of fatty acids, sterols, and bile acids in cats with chronic enteropathy

##### MV Giordano^1^, PE Crisi^1^, A Gramenzi^1^, D Cattaneo^2^, L Corna^2^, CH Sung^3^, MK Tolbert^3^, JM Steiner^3^, J Suchodolski^3^, A Boari^1^

###### ^1^University of Teramo, Teramo, Italy; ^2^Endovet Professional Association, Rome, Italy; ^3^Texas A&M University College Station, USA

Feline chronic enteropathies (FCE), include food‐responsive‐enteropathy (FRE), inflammatory bowel disease (IBD), and small cell lymphoma (SCL), and are common causes of chronic gastrointestinal signs in cats. Changes in intestinal microbiota and fecal metabolites have been reported in dogs and humans with chronic enteropathy, however, similar changes in cats are not completely understood. Therefore, this study aimed to evaluate the fecal microbiota and metabolome in cats with FCE in comparison to healthy cats (HC).

A total of 34 cats with FCE (13 FRE, 15 IBD, 9 SCL) and 27 HC were enrolled. To assess the intestinal microbiota, the feline Dysbiosis Index (DI) was evaluated using qPCR assays. Twenty‐seven targeted fecal metabolites were measured by targeted gas chromatography‐mass spectrometry analysis, including selected fecal fatty acids, sterols, and bile acids. Statistical analysis was based on univariate and multi‐variate principal coordinate analysis (PCA) in Metaboanalyst 5.0.

The DI was significantly higher (*P <* 0.0001) in cats with FCE (median: 1.3, range: −2.4 to 3.8), compared to HC (median: −2.3, range: −4.3 to 2.3), but no difference was found among the FCE subgroups. The abundance of *Faecalibacterium (P <* 0.0001), *Bacteroides (P <* 0.0001*)*, *Fusobacterium* (*P* = 0.0398), and *Bifidobacterium (P* = 0.0004), was significantly decreased in cats with FCE compared to HC. Fecal concentrations of myristic acid (*P* = 0.0114), stearic acid (*P* = 0.0059), arachidonic acid (*P* = 0.0047), erucic acid (*P* = 0.0346), and nervonic acid (*P* = 0.0217) were significantly increased in cats with FCE compared to HC. Fecal concentrations of cholesterol (*P <* 0.001), brassicasterol (*P <* 0.001), and lathosterol (*P* = 0.0372) were significantly increased in cats with FCE, while the phytosterols coprostanol (*P* = 0.0033), campesterol (*P* = 0.0498), fucosterol (*P* = 0.002), beta‐sitostanol (*P* = 0.0015), and sitostanol (*P <* 0.0001) were significantly decreased in cats with FCE. Based on PCA analysis, the fecal fatty acid and sterol profiles of cats with FRE were more similar to healthy cats, and different from cats with IBD and SCL. No differences in fecal bile acids were found between FCE and HC.

Cats with FCE have alterations in the intestinal microbiota and fecal fatty acids and sterol profiles compared to healthy cats. Although based on univariate analysis no significant differences were found among the FCE subgroups, on multi‐variate analysis cats with FRE were more similar to healthy cats, suggesting potentially less severe changes in gut function compared to cats with IBD and SCL.

**Disclosures**

Chi H Sung, Tolbert MK, Steiner JM, and Suchodolski J are employed by the Gastrointestinal Laboratory at Texas A&M University, which provides assays for intestinal function and microbiota analysis on a fee‐for‐service basis.

## ESCG‐O‐3

### ESCG—European Society of Comparative Gastroenterology

#### Association of serum and fecal microRNA profiles in healthy cats and cats with gastrointestinal cancer or chronic inflammatory enteropathy

##### JG Lyngby^1^, L Brogaard^2^, AT Kristensen^1^, MF Fredholm^1^, C Bjørnvad^1^, S Salavati Schmitz^3^, E Skancke^4^, J Morris^5^, N Dupont^1^, D Argyle^3^, A Sánchez^6^, A Spohr^7^, K Graarup‐Hansen^8^, L Nielsen^1^, S Cirera^1^

###### ^1^ University of Copenhagen, Frederiksberg C, Denmark; ^2^ DTU Kgs., Lyngby, Denmark; ^3^ The University of Edinburgh Easter Bush, Midlothian, UK; ^4^ Norwegian University of Life Sciences, Ås, Norway; ^5^ University of Glasgow, Glasgow, UK; ^6^ Universitat Autònoma de Barcelona, Barcelona, Spain; ^7^ Evidensia Faxe Animal Hospital, Faxe, Denmark; ^8^ Evidensia Karlslunde Animal Hospital, Greve, Denmark

Differentiation of gastrointestinal cancer (GIC) from chronic inflammatory enteropathies (CIE) in cats can be challenging and often require extensive diagnostics. MicroRNAs (miRNAs) represent a promising avenue as non‐invasive biomarkers in serum and feces for diagnosis of GIC. The objective was to identify serum and fecal miRNAs with diagnostic potential for differentiation between cats with GIC, CIE, and healthy cats. We hypothesized that cats with GIC will display serum and fecal miRNA profiles that differ significantly from healthy cats and cats with CIE.

Ten healthy cats, 9 cats with CIE, and 10 cats with GIC, all client‐owned, were recruited in an international multicenter observational prospective case‐control study. Serum and fecal samples were screened using small RNA sequencing for miRNAs that differed in abundance between cats with GIC, CIE, and healthy cats. Using the identified miRNAs combined with relevant miRNAs from the literature the potential diagnostic biomarker was confirmed using reverse transcription quantitative real‐time PCR (RT‐qPCR).

Serum miR‐223‐3p was found to distinguish between cats with GIC and CIE with an AUC of 0.900 (95% CI 0.760–1.0), sensitivity of 90% (95% CI 59.6%–99.5%), and specificity of 77.8% (95% CI 45.3%–96.1%). Serum miR‐223‐3p likewise showed promise in differentiating a subgroup of cats with small cell lymphoma (SCL) from those with CIE. No fecal miRNAs could distinguish between cats with GIC and CIE.

In conclusion, serum miR‐223‐3p might potentially serve as a noninvasive diagnostic biomarker of GIC in cats, in addition to providing a much needed tool for the differentiation of CIE and SCL.

**Disclosures**

Charlotte R Bjørnvad: Research collaboration: C&D foods and Royal Canin. Consultant: Novozymes, Boehringer Ingelheim, Chr. Hansen, Vuffeli

**Source of Funding**

This study was funded by the Independent Research Fund Denmark—Technology and Production, Grant/Award Number: DFF‐6111‐00124

## ESCG‐O‐4

### ESCG—European Society of Comparative Gastroenterology

#### Serum metabolomic analysis of dogs with chronic enteropathies

##### W Simpson, P Miller, P Loftus, M Rishniw, E Frederick, J Wakshlag

###### Cornell University, Ithaca, USA

Chronic enteropathies (CE) in dogs are categorized by serum albumin concentration and response to treatment. The metabolic consequences of different CE phenotypes and the ability of serum metabolites to distinguish between them are unclear. This study aimed to characterize the serum metabolome of dogs with and without protein losing enteropathy (PLE).

Serum samples from 45 client‐owned dogs: 30 CE (23 non‐PLE and 7 PLE) and 15 healthy controls were prospectively archived at −80°C. This was approved by the Institutional Animal Care and Use Committee. Metabolomic analysis was performed using ultrahigh performance liquid chromatography‐tandem mass spectroscopy (Metabolon). Raw data was extracted, peak‐identified and QC processed using Metabolon's hardware and software. Statistical calculations included Welch's two‐sample *t*‐test and principal components analysis (PCA). Metabolites with *P* < 0.05 and a false discovery rate of *q* < 0.1 were considered significant.

Metabolomics identified 835 biochemicals: 755 named and 80 with unknown identity. Many metabolites were differentially impacted by CE (n, up/down): non‐PLE: control = 247, 65/182; PLE: control = 316, 62/254; PLE: non‐PLE = 241, 50/191. PCA revealed clear separation along component 1 (14.05%), with some overlap between the non‐PLE and control groups, and the PLE group clustering independently of these cohorts. Biochemical pathways impacted by CE (PLE + non‐PLE) included nitrogen, amino acid, lipid and benzoate metabolism, energetics and enterohepatic circulation. Levels of the predominate free fatty acids (C16:0, C16:1, C18:0, C18:1), betaine, glutamine, N‐acetylaspartate, phenylalanine, tyrosine, kyneurenine, alpha‐hydroxyisocaproate, cysteine, proline, urea, fumarate, 5‐dodecenoylcarnitine, bilirubin, equol sulfate, alpha‐tocopherol, primary and secondary bile acids, bilirubin and biliverdin were lower in CE (PLE + non‐PLE) compared to controls. Butyrate: isobutyrate, 2‐oxoarginine, S‐methylcysteine and octadecenedioylcarnitine (C18:1‐DC) were increased in CE (PLE + non‐PLE) relative to controls. The CE non‐PLE group had lower levels of aspartate, leucine, citrate and aconitate, and higher levels of sebacate and erucate than controls. Increases in dimethylglycine, glutamate, 1‐methyl‐histidine, creatine, 3‐hydroxybutyrate and metabolites of purine, pyrimidine and ascorbate, and decreases in methionine, plasmalogens, cholesterol, and a subset of sphingomyelins and benzoates were restricted to CE PLE.

This global metabolic profiling study reveals pleiotropic metabolic perturbations associated with CE and PLE that may serve as biomarkers and targets for therapeutic intervention.

**Disclosures**

Dr. Simpson is a Member of the Scientific Advisory Boards for Farmina Pet Foods and Nestle Purina Petcare. He is a paid consultant for Kerry. Conflict of interest was managed by the Office of Research Integrity and Assurance (ORIA) at Cornell University.

**Source of Funding**

Farmina Pet Foods

## ESCG‐O‐5

### ESCG—European Society of Comparative Gastroenterology

#### Investigation of fecal microbiome and metabolome perturbations in dogs with protein‐losing enteropathy

##### F Cagnasso^1^, J Suchodolski^2^, A Borrelli^1^, E Bottero^3^, E Benvenuti^3^, MK Tolbert^2^, CC Chen^2^, PR Giaretta^2^, P Gianella^1^

###### ^1^Department of Small Animal Sciences, University of Turin, Grugliasco, Italy; ^2^Gastrointestinal Laboratory, Department of Small Animal Clinical Sciences, Texas A&M University, College Station, USA; ^3^Associazione professionale Endovet, Cuneo, Italy

Canine protein‐losing enteropathy (PLE) is a severe gastrointestinal syndrome characterized gastrointestinal loss of proteins. While dysbiosis and fecal metabolome perturbations have been previously reported in dogs with chronic enteropathy, they have not been widely studied in dogs with PLE, and the effects of treatment are unknown. Characterization of microbiome and metabolome perturbations could improve the management of PLE. Therefore, the study aims were (i) to investigate gut microbiome and targeted fecal metabolites in dogs with PLE and (ii) evaluate whether treatment affects these changes at short‐term follow‐up.

Fifty‐eight dogs with a diagnosis of PLE and histopathological evidence of gastrointestinal inflammation were prospectively enrolled, and 50 healthy dogs were included as a control group in this observational study. Non‐gastrointestinal causes of hypoalbuminemia, defined by a serum albumin ≤2.8 g/dL, were ruled out prior to enrollment. Fecal samples were collected before endoscopy (T0) and after one month of therapy (T1). Microbiome alterations were investigated using qPCR assays for determination of the dysbiosis index (DI). Metabolome alterations were investigated using gas chromatography coupled with mass spectrometry for the determination of fecal sterols and fatty acids (FAs).

Median (min–max) canine chronic enteropathy clinical activity index (CCECAI) and serum albumin at T0 were 9 (3–17) and 1.8 g/dL (0.8–2.8) in dogs with PLE and had improved at T1 with median (min‐max) values of 5 (0–13) and 2.3 g/dL (1.2–3.2), respectively. Median (min‐max) DI of PLE dogs was −0.4 (−5.9–8.3) and was significantly higher (*P* < 0.0001) than median DI in healthy dogs that was −2.0 (−6.0 to 5.3). No significant associations were found between DI and CCECAI, and serum albumin, cobalamin, cholesterol or C‐Reactive Protein. DI did not significantly differ between T0 and T1. In PLE dogs, among FAs measured at T0, myristate, palmitate, linoleate, oleate, cis‐vaccenate, stearate, arachidonate, gondoate, docosanoate, erucate, and nervonate, were significantly increased (adjusted *P* < 0.001); arachidonate (adjusted *P* = 0.024) was significantly decreased at T1 compared to T0. At T0, among zoosterols measured, cholesterol (adjusted *P* = 0.0012) was significantly increased in PLE dogs and significantly decreased (adjusted *P* = 0.046) at T1 compared to T0. Total measured phytosterols were significantly decreased in PLE dogs at T0, and significantly increased at T1 (both adjusted *P* = 0.0012). Among measured phytosterols at T0, beta‐sitosterol, sitostanol, fusosterol, campesterol, and lathosterol were significantly decreased (all adjusted *P* < 0.05) in PLE dogs.

This observational study shows that microbiome and lipid metabolism perturbations occurred in dogs with PLE. These alterations partially recovered after treatment.

**Disclosures**

Jan S Suchodolski, M. Katherine Tolbert, Chih‐Chun Chen, and Paula R Giaretta are employed by the Gastrointestinal Laboratory at Texas A&M University, which provides assays for intestinal function and microbiota analysis on a fee‐for‐service basis.

## ESCG‐O‐6

### ESCG—European Society of Comparative Gastroenterology

#### Fecal microbial transplantation effect on clinical outcome and fecal microbiota and metabolome in dogs with chronic enteropathy refractory to diet

##### C Vecchiato^1^, D Erba^2^, F Sportelli^1^, C Delsante^1^, M Alonzo^2^, C Pinna^1^, M Sabetti^1^, J Suchodolski^3^, R Pilla^3^, P Giaretta^3^, M Pietra^1^, G Biagi^1^, F Procoli^2^

###### ^1^University of Bologna, Ozzano dell'Emilia, Italy; ^2^Anicura Ospedale Veterinario I Portoni Rossi Zola Predosa, Bologna, Italy; ^3^Gastrointestinal Laboratory, Texas A&M University College Station, TX, USA

Fecal microbiota transplantation (FMT) is an established treatment for recurrent *Clostridioides difficile* infection in humans, and it is currently being explored to modulate fecal microbiota (FM) in dogs with chronic enteropathy (CE). Little is known about the long‐term clinical efficacy and safety of FMT in dogs with CE. The study aim was to evaluate the effects of FMT on clinical outcome, FM, and metabolome in dogs with CE refractory to a 14‐day diet trial. In this prospective, longitudinal, multi‐centric study, 20 dogs (median age 41, range 10–118 months) were enrolled if they presented with chronic (≥three weeks duration) idiopathic GI signs with no improvement after an elimination diet trial of two weeks duration. Based on clinical response, dogs were treated with up to two FMTs administered by rectal enema within a two week‐period. Body weight (BW), fecal scores (FS), and canine IBD activity index (CIBDAI) were assessed at T0 (before FMT), and one, two and three months after the last FMT (T1, T2, and T3, respectively). Feces were collected to evaluate the dysbiosis index (DI) and fecal metabolome. Differences between T0 and the following time points were assessed by pairwise tests. Data are expressed as median (range). In total, 8/20 dogs received one FMT, and 12/20 received two FMTs. No side effects were noted during or after FMT(s) procedure. An improvement of clinical parameters was evident after FMT(s) up to T2. BW increased at each time point (*P* < 0.05) with no diet adjustments. After T2, clinical signs relapsed in two dogs, and another one was excluded due to a disease unrelated to CE or FMT procedure. At T0, CIBDAI was 5 (1–9) and decreased to 1 (0–5) at T1 and T2 (*P* < 0.0001), and to 1 (0–3) at T3 (*P* < 0.0001). At T0, FS was 4 (1–7), and decreased to 2 (1–5) at T1 and T2 (*P* < 0.01), and to 2 (2–4, *P* = 0.08) at T3. At T0, median DI was −0.1 (range −5.6 to 3.8), which decreased (*P* < 0.05) to −2.1 (−5.7 to 4.7) at T1. At T1 vs. T0, a significant increase of propionic acid (from 26% to 32%, *P* = 0.047) was detected in feces.This prospective study suggests that FMT is a safe procedure to modulate FM and can improve clinical signs in a subset of dogs with CE, with a long‐term response of at least 3 months.

**Disclosures**

All financial relationships are as follows: Vecchiato CG, speaking and consultancies: Monge, Prolife‐Zoodiaco. Delsante C, consultancies: Pettech Suchodolski JS, Pilla R, and Giaretta PR are employees of the Gastrointestinal Laboratory at Texas A&M University that offers testing including Dysbiosis Index on a free‐for‐service basis. JSS is the Purina Petcare Endowed Chair for Microbiome Research, and has received consulting and/or speaking fees from IDEXX Laboratories, Purina Petcare, Royal Canin, Hill's Pet Nutrition, Blue Buffalo, Exegi Pharma, and Nutramax Laboratories. Biagi G, speaking and consultancies: Candioli, Innovet, Aurora Biofarma, Purina, CIAM, Ecuphar, Eland, Prolife‐Zoodiaco, Pettech. Procoli F, speaking fees: Purina, Nestle, Hill's, Royal Canin Waltham, Idexx Laboratories, NBF Lanes. The authors not mentioned declare no potential conflicts of interest.

## ESCG‐O‐7

### ESCG—European Society of Comparative Gastroenterology

#### Plasma proteome signatures of canine acute haemorrhagic diarrhoea syndrome (AHDS)

##### L Huber^1^, B Kuropka^3^, PG Doulidis^1^, E Baszler^1^, L Martin^1^, C Frizzo Ramos^2^, A Rodríguez‐Rojas^1^, IA Burgener^1^

###### ^1^Department for Companion Animals, Small Animal Internal Medicine, University of Veterinary Medicine, Vienna, Austria; ^2^The Interuniversity Messerli Research Institute, Medical University Vienna and University of Veterinary Medicine Vienna, Vienna, Austria; ^3^Protein Biochemistry, Institute of Chemistry and Biochemistry, Free University of Berlin, Berlin, Germany

Acute hemorrhagic diarrhea is a common complaint in dogs. In addition to causes like intestinal parasites, dietary indiscretion, intestinal foreign bodies, canine parvovirus infection, or hypoadrenocorticism, acute hemorrhagic diarrhea syndrome (AHDS) is an important and sometimes life‐threatening differential diagnosis. There is some evidence supporting the link between *Clostridium perfringens* toxins and AHDS. These toxins may be partially responsible for the epithelial cell injury, but the pathogenesis of AHDS is not fully understood. Recent studies have also suggested that severe damage to the intestinal mucosa and associated barrier dysfunction can trigger chronic gastrointestinal illnesses. Besides bloodwork and classical markers for AHDS, we focused mainly on the plasma‐proteome to identify systemic pathological alterations during disease and search for potential biomarkers to improve diagnosis. To accomplish this, we used liquid chromatography mass spectrometry to compare the proteomic profiles of 20 dogs with AHDS to 20 age‐, breed‐, and sex‐matched control dogs using surplus samples from previous prospective studies. The peptides were analyzed using an Ultimate 3000 reversed‐phase capillary nano liquid chromatography system connected to a Q Exactive HF mass spectrometer. We identified and quantified 207 plasmatic proteins, form which dozens showed significant altered levels in AHDS. SerpinA3, Lipopolysaccharide‐binding protein, Ig‐like domain‐containing protein, Glyceraldehyde‐3‐phosphate dehydrogenase and Serum amyloid A were the most abundant, while Paraoxonase, Selenoprotein, Amine oxidase, Apolipoprotein C‐IV were significantly less abundant. Our study will contribute to the understanding of the etiology and pathophysiology of AHDS. Furthermore, potential new biomarkers may help in confirming and verifying the diagnosis.

**Disclosures**

No disclosures to report

## ESCG‐O‐8

### ESCG—European Society of Comparative Gastroenterology

#### Combined omeprazole and carprofen induced fecal dysbiosis and decreased short‐chain fatty acid production in healthy dogs

##### R Pilla^1^, J Shih^1^, J Suchodolski^1^, K Messenger^2^, M Tolbert^1^

###### ^1^Texas A&M University, College Station, USA; ^2^North Carolina State University, Raleigh, USA

Proton pump inhibitors (PPIs) such as omeprazole are commonly used in conjunction with non‐steroidal anti‐inflammatory drugs (NSAIDs) to reduce adverse effects (e.g., gastrointestinal erosions, ulcers) associated with NSAID use. However, NSAID‐induced intestinal injury is believed to be modulated by microbiota, and PPIs have been shown to induce dysbiosis in several species. In a recent study, we demonstrated that the combination of omeprazole and carprofen (OC) increased fecal calprotectin and dysbiosis index and decreased four short chain fatty acid (SCFA)‐producing bacteria in dogs. Therefore, our goals were to further characterize the dysbiosis induced by OC in healthy dogs using 16S rRNA sequencing and assess SCFA production.

Leftover samples from a previous prospective sequential study including six healthy adult colony beagle dogs were used. Samples were collected at the end of the first (no intervention, baseline, B), second (carprofen 4mg/kg q24 PO, CAR) and third 7‐day periods (carprofen plus omeprazole 1 mg/kg q12h PO, OC). Interventions were separated by 4‐week washouts. 16S rRNA sequencing was performed and analyzed with QIIME2. Fecal SCFA were measured using an established GCMS assay. Univariate statistics were performed with Friedman's test and adjusted for multiple comparisons. Multivariate statistics was performed with ANOSIM.

Beta diversity (weighted UniFrac) showed significant differences for both interventions compared to baseline, however, size effect was larger in OC, and samples clustered visibly separate from B and CAR. At the genus level, the abundance of 6 genera associated with SCFA production, including *Turicibacter* (*q* = 0.045), *Ruminococcus* (*q* = 0.036), *Blautia* (*q* = 0.002), and unnamed genera within families Lachnospiraceae (*q* = 0.002), Clostridiaceae (*q* = 0.036) and Peptostreptococacceae (*q* = 0.036) was significantly decreased, while *Lactobacillus* (*q* = 0.036) was increased on OC compared to B. Acetate and propionate were significantly decreased by OC (*P* = 0.04 and 0.01, respectively) compared to B.

Our results are similar to the literature in humans, in which lactic acid bacteria are increased following PPI treatment, and the abundance of several SCFA‐producing bacteria are decreased. Acetate protects the intestinal barrier and has anti‐inflammatory properties, while propionate increases cell turnover and promotes intestinal epithelial repair. Therefore, the dysbiosis profile identified in our study, and associated decrease of acetate and propionate can help explain the previously increased fecal calprotectin induced by OC in healthy dogs. These data also support, in the absence of signs of or multiple risk factors for gastrointestinal bleeding, combined omeprazole and carprofen is not recommended and may be harmful.

**Disclosures**

RP, JS, JSS and KT are employees at the Texas A&M Gastrointestinal Laboratory, which offers multiple tests for the assessment of gastrointestinal function on a fee‐for‐service basis.

**Source of Funding**

The original trial was supported by the AVMF Research Grant for Pain Management in Dogs and the NIH‐T‐35 Interdisciplinary Biomedical Research Training Program.

## ESCG‐O‐9

### ESCG—European Society of Comparative Gastroenterology

#### Functional cobalamin deficiency (normal cobalamin with increased methylmalonic acid concentration) is uncommon in dogs

##### S. S. Siegrist^3^, M.H. Hersberger^2^, D.B. Brugger^4^

###### ^1^Clinic for Small Animal Anternal Medicine Zurich Switzerland; ^2^University Children's Hospital Zurich Zurich Switzerland; ^3^Clinic for Small Animal Internal Medicine Zurich Switzerland; ^4^Institute of Animal Nutrition and Dietetics, Vetsuisse Faculty University of Zur Zurich Switzerland

Cobalamin (Cbl) supplementation is recommended when Cbl is in the lower part of the reference interval (RI). This is based on a laboratory study measuring Cbl and methylmalonic acid (MMA) concentrations using leftover samples without exclusion of prior Cbl supplementation. Therefore we wanted to assess the prevalence of increased MMA concentrations in dogs with normal and subnormal Cbl concentrations. A secondary goal was to describe clinical findings of dogs with so‐called functional Cbl deficiency (normal Cbl, increased MMA).

We included dogs examined at our gastroenterology service between 11/2016 and 03/2021 that had Cbl and MMA measured from the same sample. Cbl supplementation within 6 months before consultation was an exclusion criterion. Cbl (RI, 192–738 pmol/L) was measured using a chemiluminescence assay with a lower detection limit of 111 pmol/L. MMA (RI, 393–1476 nmol/L) was measured using ultra‐performance liquid chromatography‐tandem mass spectrometry.

A total of 104 paired Cbl/MMA results were available. Cbl was low in 31 (30%) dogs with 11/31 (35%) dogs having undetectably low values. Cbl was normal in 68 (65%) and >RI in 5 (5%) of dogs. Increased MMA (median 2740 nmol/L) was found in 5/31 (16%) dogs with low Cbl including 4/11 (36%) dogs with undetectably low Cbl (median MMA 5790 nmol/L). Increased MMA (median 6070 nmol/L) was found in 4/68 (6%) dogs with normal Cbl and in no dog with Cbl >RI. A significant inverse correlation was found between serum Cbl and MMA concentrations (*r*_s_ = −0.256, *P* = 0.009). The diagnostic utility of serum Cbl to predict MMA concentration >RI was low with an AUC = 0.590 (95% CI, 0.331–0.849). The four dogs with functional Cbl deficiency were a Cocker Spaniel (10y) with suspicion of chronic pancreatitis (Cbl 260 pmol/l, MMA 1673 nmol/L), a Continental Bulldog (2y) with atopic dermatitis and food‐responsive diarrhea (Cbl 411 pmol/L, MMA 9620 nmol/L), an Australian Terrier (11y) with a hepatocellular adenoma and chronic enteropathy (Cbl 489 pmol/L, MMA 8940 nmol/L), and a Parson Russel Terrier (12y) with chronic pancreatitis, a non‐functional adrenal mass and nodular hepatopathy (Cbl 710 pmol/L, MMA 3200 nmol/L). Three dogs were supplemented with Cbl but it was difficult to recognize a clinical improvement after supplementation.

In conclusion, the majority of dogs with low Cbl (84%) and undetectable Cbl (64%) still have MMA concentrations within RI. Functional Cbl deficiency is rare in dogs and the clinical picture appears variable. Studies are needed to characterize the clinical significance of functional Cbl deficiency in dogs.

**Disclosures**

No disclosures to report

## ESCG‐O‐10

### ESCG—European Society of Comparative Gastroenterology

#### Intestinal lymphangiectasia is a common finding in healthy soft‐coated wheaten terriers

##### M Tolbert^1^, J Darrow^2^, L Grubb^3^, S Fitzgerald^3^, M Hong^1^, C Sung^1^, J Steiner^1^, J Suchodolski^1^

###### ^1^Texas A&M University, Raleigh, USA; ^2^North Carolina State University, Raleigh, USA; ^3^Triviumvet Waterford, Ireland

Soft‐coated wheaten terriers (SCWTs) have a familial predisposition to the development of protein‐losing enteropathy (PLE). SCWTs with PLE tend to be diagnosed in middle‐age with clinical signs supportive of PLE, including diarrhea, vomiting, and weight‐loss. Intestinal lymphangiectasia (IL) is thought to be the primary contributor to PLE in SCWTs. Evidence of IL may precede the development of biochemical changes or clinical signs. Based on previous work performed in Yorkshire Terriers, we hypothesized that IL and fecal long‐chain fatty acid and sterol loss would be a more common finding in healthy SCWTs and PLE dogs compared to healthy non‐SCWTs. In a prospective, IACUC‐approved study, dietary history, physical examination, minimum database including complete blood count, serum biochemistry profile, and urinalysis, fecal testing including fecal sterols and fatty acids, serum concentrations of cPLI, cTLI, cobalamin, and folate, and video capsule endoscopy (VCE) were performed in 12 healthy SCWTs, 9 healthy, age‐ and weight‐matched non‐SCWTs, and 8 dogs of various breeds with PLE (all samples collected after withholding food for a minimum of 16 hours). All healthy dogs had to be free of any chronic history of GI signs and have normal blood work and a negative fecal flotation to be enrolled. The presence and severity of GI erosions, ulcerations, and IL as identified by VCE was presented as descriptive data. Following log transformation, fecal fatty acids and sterols were compared among groups using one‐way ANOVA and post‐hoc Fisher's least significant difference test (adjusted *P <* 0.05). Dietary fat fed was similar between healthy non‐SCWT and SCWT dogs. Gastrointestinal erosions or ulcerations were identified in six healthy dogs, eight SCWTs, and eight dogs with PLE. IL was more common in SCWT (*n* = 8) and PLE dogs (*n* = 8) compared to non‐SCWT dogs (*n* = 1). There was a visual difference in clustering when evaluating fecal fatty acids and sterols, with all three groups clustering separately. Fecal sitostanol was significantly lower and nervonic acid significantly higher (*P* < 0.001 for both) in PLE dogs and SCWT dogs compared to healthy controls. Fecal cholesterol, arachidonic, stearic, and vaccenic acids (*P* < 0.01 for all) were significantly higher in PLE dogs compared to all groups. Gastrointestinal erosions are a relatively common finding in dogs, regardless of health status. However, clinically healthy SCWT dogs have changes such as IL and altered fecal fatty acids and sterols that mirror those observed in dogs with PLE.

**Disclosures**

Study partially funded by Triviumvet and partially by the TAMU GI lab. Two authors (Grubb and Fitzgerald) are employees Triviumvet. Five authors (Tolbert, Sung, Hong, Suchodolski, Steiner) are employees of the GI lab. Tolbert also serves on Triviumvet SAB.

**Source of Funding**

Triviumvet and TAMU GI Laboratory

## ESCG‐O‐11

### ESCG—European Society of Comparative Gastroenterology

#### Early‐life antibiotic exposure and susceptibility to chronic diarrhea during adulthood in cats

##### EM Stavroulaki^1^, GT Fosgate^2^, KT Moraiti^1^, PG Xenoulis^1^

###### ^1^ Faculty of Veterinary Science, Clinic of Medicine, University of Thessaly, Karditsa, Greece; ^2^ Department of Production Animal Studies, University of Pretoria, Onderstepoort, South Africa

There are multiple reports that antibiotic exposure causes a long‐term disturbance of the gastrointestinal microbiota. Based on human studies, antibiotic‐induced dysbiosis is associated with an increased susceptibility to gastrointestinal diseases. However, no such evidence currently exists for cats. The aim of this study was to investigate whether antibiotic use during the first year of life is related to the development of chronic diarrhea (i.e., more than 3 weeks’ duration) later in life (during adulthood) in cats.

A cross‐sectional, questionnaire‐based study was performed at a teaching and a private referral hospital in Greece. The questionnaire contained 54 questions related to the signalment, environment, diet, medications, antibiotic exposure, and clinical characteristics during the first year of life, as well as certain pathologic conditions currently present. The questionnaire was completed by veterinarians along with communication with the owners and access to the cats’ medical records. Univariate logistic regression was performed initially to screen for potential risk factors associated with chronic diarrhea during adulthood. Variables with a *P* < 0.2 were added into a multivariable logistic regression model and a backwards stepwise approach was used with statistical significance set at *P <* 0.05.

A total of 104 cats were included in the study. Of these, 55% (58/103) had received at least one course of antibiotics before the first year of age. A total of 11% (11/95) cats had chronic diarrhoea of which 91% (10/11) had received antibiotics. Cats with chronic diarrhoea during adulthood were 19.9 times more likely (95% CI; 1.9–208.3) to have received antibiotics before the first year of age (*P* = 0.012) when adjusting for potential confounding of age and diet in the multivariable model.

Similar to the findings of human studies, cats treated with antibiotics during early life are more likely to develop chronic diarrhoea during adulthood. Further studies are needed to determine the clinical significance of these findings and the exact pathogenetic association between early‐life antibiotic exposure and chronic diarrhea in cats.

**Disclosures**

No disclosures to report

## ESCG‐O‐12

### ESCG—European Society of Comparative Gastroenterology

#### Congenital partial colonic agenesis in cats: clinical, biological, diagnostic imaging, endoscopic and histopathologic characterization. A retrospective study of 17 cases

##### P Remmel, L Gros, J Mortier, V Freiche

###### Ecole Nationale Vétérinaire d'Alfort, Maisons‐Alfort, France

Congenital diseases of the large intestine have scarcely been reported and mostly include fistula, atresia, or colonic duplication. Cases of partial colonic agenesis have rarely been described. The purpose of this study was to report a cohort of cats diagnosed with partial colonic agenesis.

The colon was measured during colonoscopy or contrast‐radiographies and compared to the average length described in the literature (~30 cm, range [27–37] cm). 17 cats between 2016 and 2022 were retrospectively included and had a colon length that ranged from 8 to 20 cm. Continuous data were presented as median [minimum—maximum].

Median age of presentation was variable and long asymptomatic periods were common. Depending on the cases, partial colonic agenesis could then represent an incidental finding or the likeliest cause of clinical signs. Even if diarrhea was reported in most cases, no specific clinical or biological abnormality was associated with it. Abdominal ultrasound was useful and identified a short colon in 14/17 cats. Contrast abdominal x‐rays were performed in three cases and diagnosed the agenesis. Endoscopy remained necessary to confirm the diagnosis and identify associated lesions and complications. Among others, colonic stenosis was reported in 8/9 cases that had lifelong clinical signs and the shortest colon length (median length 13 vs. 18 cm, range [8–20] cm vs. [15–20] cm, *P* value = 0.04). This anatomical abnormality could promote chronic inflammation that might generate fibrosis and ultimately stenosis. Treatments and follow‐ups were reported and depended on a case by case management. One case had a colonic stenosis managed by transient stent placement that resolved associated dyschezia. Other cases were medically managed (antibiotics, glucocorticoids, hyper digestible diet) with mild to moderate improvement over time.

This study highlighted that after excluding infectious and food responsive colonic diseases, a partial colonic agenesis could be considered as a possible cause of chronic diarrhea and should be integrated in the differential diagnosis in young cats. Its early identification might prevent further complications such as colonic stenosis.

**Disclosures**

No disclosures to report

## ESVC‐O‐1

### ESVC—European Society of Veterinary Cardiology

#### Echocardiographic evaluation of main pulmonary artery and right pulmonary artery size in dogs with pulmonary hypertension

##### G Grosso^1^, R Tognetti^1^, O Domenech^2^, A Della Pina^1^, F Marchesotti^2^, V Patata^2^, T Vezzosi^1^

###### ^1^University of Pisa, San Piero a Grado, Pisa Italy; ^2^Anicura Istituto Veterinario Novara, Novara, Italy

Pulmonary artery (PA) is one of the main anatomic sites evaluated for the echocardiographic assessment of pulmonary hypertension (PH) in dogs according to the recent ACVIM guidelines. The diameter of the main PA (MPA) and the right pulmonary artery (RPA) distensibility index are two of the echocardiographic parameters used to assess the echocardiographic probability of PH in dogs. In humans, the echocardiographic evaluation of MPA size plays an important role for the diagnosis of PH. Few studies evaluated the echocardiographic assessment of PA branches in patients with PH. We hypothesized that PA branches could yield an higher diagnostic accuracy for the detection of PH in comparison to MPA. Therefore, the aim of this study was to evaluate the MPA and RPA size for the echocardiographic detection of an intermediate‐to‐high probability of PH in dogs.

This was a prospective, multicenter, observational study. The MPA diameter, the RPA maximum diameter (RPAmax) and the RPA minimum diameter (RPAmin) were normalized to bodyweight using allometric scales (MPA_N, RPAmax_N and RPAmin_N, respectively). The MPA was also indexed to the aortic root diameter (MPA/Ao). The classification and the different type of PH were assessed according to the ACVIM guidelines. The ROC curve analysis and the Youden Index were used to evaluate the diagnostic accuracy of PA size parameters and respective cut‐offs for the detection of an intermediate‐to‐high probability of PH.

Among a total of 410 dogs included, 274 dogs had PH and 136 dogs were healthy (control group). Among dogs with PH, 161 had pre‐capillary PH and 113 had post‐capillary PH. Dogs with PH had significantly higher MPA_N, MPA/Ao, RPAmax_N and RPAmin_N in comparison to the control group (*P* < 0.05). No differences in MPA_N, MPA/Ao, RPAmax_N and RPAmin_N were found between pre‐capillary and post‐capillary PH; therefore, all PH dogs were considered as a single group. Among dogs with PH, RPAmin_N showed the best diagnostic accuracy for the detection of an intermediate‐to‐high probability of PH [area under curve (AUC) = 0.96; *P* < 0.001; sensitivity(Se) = 87%, specificity(Sp) = 97%] in comparison to RPAmax_N (AUC = 0.81; *P* < 0.001; Se = 68%, Sp = 85%), MPA_N (AUC = 0.87; *P* < 0.001; Se = 83%, Sp = 79%) and MPA/Ao (AUC = 0.92; *P* < 0.001; Se = 84%, Sp = 90%).

In conclusion, this study confirms that the echocardiographic assessment of MPA size has an important role in the diagnosis of PH in dogs. However, our results suggest that the echocardiographic evaluation of PA branches, especially RPAmin_N, could have a higher diagnostic accuracy and could be a new useful tool for the detection of PH in dogs.

**Disclosures**

No disclosures to report

## ESVC‐O‐2

### ESVC—European Society of Veterinary Cardiology

#### Left ventricular myocardial protein profile in dogs with myxomatous mitral valve disease and dilated cardiomyopathy

##### Mister Phetariyawong^1^, J Häggström^1^, Å Ohlsson^1^, E Axelsson^2^, F Van steenbeek^3^, K Höglund^1^, M Oyama^4^, G Andersson^1^, J Bergquist^5^, I Ljungvall^1^

###### ^1^Swedish University of Agricultural Sciences, Uppsala, Sweden; ^2^Swedish board of agriculture, Jönköping, Sweden; ^3^Utrecht University, Utrecht, Netherlands; ^4^University of Pennsylvania School of Veterinary Medicine, Pennsylvania, USA; ^5^Uppsala University, Uppsala, Sweden

Myxomatous mitral valve disease (MMVD) and dilated cardiomyopathy (DCM) both manifest with eccentric left ventricular (LV) hypertrophy. To decipher underlying disease‐specific mechanisms, this study aimed to compare differentially expressed proteins (DEP) in the LV myocardium between MMVD, DCM, and control dogs.

Twenty‐two dogs with MMVD, 9 with DCM, and 17 controls were enrolled. Left ventricular lateral wall and posterior papillary muscle tissues were collected within 30 min after euthanasia. Liquid chromatography‐tandem mass spectrometry (LC‐MS/MS) was performed for protein identification. Proteins differing significantly (*P* < 0.05) between the three groups, and showing fold changes >1.2 or <0.8, were considered expressed at higher or lower levels, respectively, and further analysed by bioinformatical methods.

Proteins involved in cardiac hypertrophic response were expressed at higher levels and proteins involved in myocardial contractility‐ and protection at lower levels in diseased myocardium compared to in controls. Proteins involved in signaling pathways inducing myocardial fibrosis were expressed at higher levels in diseased myocardium and were lower in MMVD dogs compared to DCM dogs. Proteins involved in the serotonin signaling pathway were expressed at higher levels in MMVD dogs compared to in control‐ and DCM dogs. Proteins involved in cardiac muscle thin filament assembly, and mitochondrion organization were expressed at higher levels in MMVD than in DCM dogs.

In conclusion, the protein expression levels identified in the LV myocardial tissue samples likely reflects both potential disease‐specific mechanisms as well as chronic ongoing myocardial remodeling occurring in dogs affected by either MMVD or DCM.

**Disclosures**

‐ Dr. Ljungvall is involved in projects with/has received funding/grants from Nestle Purina, Boehringer Ingelheim, Stiftelsen djursjukhus i storstockholm, Agria Insurance Ltd/SKK, Forsbergs stiftelse and has received consulting fees from Boehringer Ingelheim, —Dr. Häggström is involved in projects with/has received funding/grants from Nestle Purina, Boehringer Ingelheim, Orion Pharma, Stiftelsen djursjukhus i storstockholm, Forsbergs stiftelse and has received consulting fees from Boehringer Ingelheim, Royal Canin, Orion Pharma. —Dr. Oyama has received grants, honoraria, consulting fees, programmatic support (funding for a research intern), and reimbursement of travel expenses from Ceva Sante Animale and Nestle Purina within the past five years.

**Source of Funding**

Agria Insurance Ltd/SKK—Thure F och Karin Forsbergs stiftelse—Sveland Animal Insurance—The government research council Formas—The Scholarship in Commemoration of His Majesty King Bhumibol Adulyadej's 90th Birthday Anniversary, Thailand

## ESVC‐O‐3

### ESVC—European Society of Veterinary Cardiology

#### Renin‐angiotensin‐aldosterone and phosphodiesterase system gene polymorphisms associated with congestive heart failure in Cavalier King Charles Spaniels with myxomatous mitral valve disease

##### MJ Reimann^1^, K Meurs^2^, J Stern^3^, J Møller^4^, T Martinussen^5^, I Ljungvall^6^, J Häggström^6^, LH Olsen^5^

###### ^1^University of Copenhagen, Borup, Denmark; ^2^North Carolina State University, Raleigh, USA; ^3^University of California‐Davis, Davis, USA; ^4^Copenhagen University Hospital Rigshospitalet, Copenhagen, Denmark; ^5^University of Copenhagen, Frederiksberg C, Denmark; ^6^Swedish University of Agricultural Sciences, Uppsala, Sweden

Myxomatous mitral valve disease (MMVD) is associated with changes in the renin‐angiotensin‐aldosterone and phosphodiesterase (PDE) systems. Gene polymorphisms with functional importance in genes involved in both systems have been identified in dogs.

The aim of the study was to determine the impact of selected angiotensin converting enzyme (ACE) and PDE5A polymorphisms on presence of congestive heart failure (CHF) due to MMVD in Cavalier King Charles Spaniels (CKCS).

In this retrospective study, 107 client‐owned CKCS with CHF and 75 control CKCS ≥8 years of age were included. Diagnosis of CHF was based on history of MMVD, previous or current clinical signs of CHF, echocardiographic changes compatible with severe MMVD and response to diuretic treatment. Blood samples were stored for PCR genotyping of selected ACE and PDE5A gene polymorphisms. Odds of having CHF were estimated using logistic regression including ACE and PDE5A gene polymorphisms as explanatory factors.

ACE polymorphism positive CKCS had reduced odds for CHF compared to negative wildtype CKCS (heterozygous odds ratio (OR) = 0.41, 95% confidence interval (95% CI) = 0.19;0.85, *P* = 0.018; homozygous OR = 0.40, 95% CI = 0.17;0.92, *P* = 0.032) while PDE5A polymorphism positive CKCS had increased odds for CHF compared to negative wildtype CKCS (heterozygous OR = 2.89, 95% CI = 1.07;8.30, *P* = 0.040; homozygous OR = 2.94, 95% CI = 1.09;8.40, *P* = 0.037).

In conclusion, selected ACE and PDE5A gene polymorphisms were associated with presence of CHF due to MMVD in CKCS. The findings indicate that in CKCS, ACE gene polymorphism might have a protective role while PDE5A gene polymorphism might be disadvantageous in relation to presence of CHF due to MMVD.

**Disclosures**

Maria J Reimann: Current affiliation is Boehringer Ingelheim Animal Health Nordics. Joshua Stern: Serves as an associate editor for the Journal of Veterinary Internal Medicine. Ingrid Ljungvall: Project collaboration with Boehringer Ingelheim. Lisbeth H Olsen: Participated in a scientific meeting in 2022 organized by Boehringer Ingelheim where accommodation was included.

**Source of Funding**

Independent Research Fund Denmark (Project no. 7017‐00131B)

## ESVC‐O‐5

### ESVC—European Society of Veterinary Cardiology

#### Gene Therapy for the Treatment of Doberman Dilated Cardiomyopathy

##### MM Sleeper^1^, NA Hammers^2^, HL Sweeney^2^

###### ^1^University of Florida Veterinary School, Gainesville, USA; ^2^University of Florida Department of Pharmacology and Therapeutics, Gainesville, USA

Dilated cardiomyopathy (DCM) is a common disease in Doberman pinschers, usually terminating in sudden death or congestive heart failure (CHF) in spite of medical management. According to one study I Dobermans, the 6‐month survival from the first episode of CHF was 7.5% with 2 dogs surviving to 12 months. Gene delivery is a tantalizing approach that could result in improved outcomes with various candidate transgenes. S100A1 is a Ca++ sensing protein in the cardiomyocyte. Its functional properties are caused mainly by increased SERCA2a activity, decreased diastolic SR Ca++ leakage and augmentation of the probability the ryanodine receptor will be in an open state. Apoptosis repressor with caspase recruitment domain (ARC) is a potent and multifunctional inhibitor of apoptosis, that can inhibit apoptosis mediated by both the death receptor and mitochondrial signaling pathways.

Prior to gene delivery, dogs were assessed for presence of vector directed neutralizing antibodies and were enrolled if the titer was <1:20. Gene delivery into the myocardium (*n* = 1) or coronary arteries (*n* = 10) was performed to cause overexpression of myocardial s100A1 and ARC (the two genes were piggybacked in one virus) in Doberman pinschers that had been diagnosed with DCM and compensated CHF. Dogs were assessed immediately prior to treatment and after treatment at 2, 4, 6, 9 and 12 months (or until death) with complete clinical laboratory testing, electrocardiography, echocardiography and quality of life questionnaire (FETCH).

For the coronary approach, delivery was considered fair in 1 dog, good in 3 dogs and excellent in 6 dogs. Dogs survived 1 to 16.5 months from the initial diagnosis of congestive heart failure with a mean survival of 6.9 months. For dogs with excellent viral delivery, mean survival was 7.6 months or 9 months if the dog that succumbed to a fatal arrhythmia in this group was removed. In the 10 dogs treated by coronary delivery, cause of death was: presumed fatal arrhythmia (*n* = 4); progressive CHF (*n* = 3); noncardiac cause (*n* = 2) and one dog remains alive. One of the presumed fatal arrhythmia dogs had a large intrathoracic mass. In the two dogs that were euthanized for noncardiac reasons, owners chose to euthanize based on progression of inflammatory bowel disease symptoms and progressive hyporexia (dog was not azotemic).

**Disclosures**

No disclosures to report

**Source of Funding**

Canine Health Foundation

## ESVC‐O‐6

### ESVC—European Society of Veterinary Cardiology

#### Combined physical examination variables and N‐terminal pro‐brain natriuretic peptide in predicting cardiac disease in asymptomatic cats with murmurs

##### R E Carter, J Novo Matos, C Partington

###### University of Cambridge, Cambridge, UK

Heart murmurs (HM) are often heard in cats asymptomatic for cardiac disease; they can be an indicator of disease but are also auscultated in cats with normal hearts. Echocardiography to detect those cats with cardiac disease might not be readily accessible to all owners. We aimed to determine whether signalment, physical examination and/or biomarker variables can predict the presence of cardiac disease, hypertrophic cardiomyopathy (HCM) or moderate‐severe left atrial enlargement (stage B2) in asymptomatic cats with a murmur. One‐hundred and sixty‐eight cats with murmurs were included in this single‐centre, prospective, cross‐sectional study. Forty‐six (46/168, 27%) cats had HCM, 38/168 (23%) had equivocal HCM, 4/168 (2%) had restrictive cardiomyopathy (RCM), 13/168 (8%) had congenital disease and 67/168 (40%) were normal. Twenty‐one cats were in stage B2: 14/21 (67%) had HCM, 3/21 (14%) had RCM and 4/21 (19%) had congenital disease. Cats with cardiac clinical signs, hyperthyroidism, systemic hypertension or anaemia weren't included. Binary logistic regression was used to identify variables that predict cardiac disease. Receiver operating characteristic curves were used to determine area under the curve (AUC) as well as sensitivity and specificity for N‐terminal pro‐brain natriuretic peptide (NT‐proBNP) cut‐offs. Data are presented as odds ratio (OR) (95% confidence interval) and AUC. Multivariable logistic regression found BCS ≥6/9, HM grade ≥3/6 and NT‐proBNP >143 pmol/L to be independent predictors of cardiac disease (OR 4.2 (1.5–14.2), OR 3.1 (1.3–7.2) and OR 11.4 (4.0–41.6) respectively). This model gave an AUC of 0.80, which was superior to NT‐proBNP alone (AUC 0.71; sensitivity 45% and specificity 92% for *a* > 143 pmol/L cut‐off). These variables were also found to be independent predictors of HCM (BCS ≥6/9, OR 4.9 (1.6–17.2); HM grade ≥3/6, OR 3.7 (1.2–13.1); NT‐proBNP >114 pmol/L, OR 17.6 (6.4–59.0)). However, AUC for this prediction model (0.86) was similar to NT‐proBNP alone (AUC 0.84; sensitivity 81% and specificity 76% for *a* > 114 pmol/L cut‐off). A HM grade ≥4/6 and an NT‐proBNP >145 pmol/L were independent predictors of stage B2 (OR 3.8 (1.2–13.1) and OR 12.0 (3.5–55.8) respectively) with an AUC of 0.84. NT‐proBNP as a sole test (AUC 0.79) detected stage B2 with 81% sensitivity and 76% specificity (>145 pmol/L cut‐off). High‐sensitivity troponin‐I was not a predictor of cardiac disease, HCM or stage B2. This study suggests that combining physical examination findings with NT‐proBNP helps predict asymptomatic heart disease and so might help to select cats that should undergo echocardiographic evaluation of their asymptomatic murmur.

**Disclosures**

No disclosures to report

## ESVC‐O‐7

### ESVC—European Society of Veterinary Cardiology

#### Investigation of the indications for and outcome of ambulatory electrocardiography in a referral population of dogs

##### FM Edgerton, G Culshaw, SA Dickson, Y Martinez‐Pereira, RR Blake

###### The University of Edinburgh, Roslin, UK

Ambulatory electrocardiography (AEG) is frequently used to assess arrhythmias in dogs. This study aimed to establish the demographics of dogs requiring AEG in one referral hospital and determine their relationships with indication for AEG and AEG diagnosis.

In this retrospective, single centre, observational study, collected data included: signalment, indication for AEG, AEG rhythm diagnosis, concurrent cardiac and non‐cardiac disease, and concurrent medications. Only first AEG analyses were included.

Over‐representation of breeds, gender types, indications for AEG and AEG diagnosis was determined by Friedman or chi‐squared tests. One‐sample proportion tests determined which indications and diagnoses were over‐represented within breeds. Potential influences of age and weight (binary logistic regression) and structural heart disease (chi‐squared test) were also determined. Significance was *P* < 0.05.

Five‐hundred and ninety dogs were included. Labradors, boxers and Cocker spaniels were over‐represented (*P* < 0.0001). Females were under‐represented because of low entire female numbers (41.36%; *P* < 0.0001). There was a right‐skew in age distribution with 11.86% <1.5 years, 35.08% 1.5–7 years and 53.05% >7 years. Commonest indications for AEG were syncope/collapse (46.44%) and arrhythmia found on auscultation/ECG (electrocardiography) (29.49%) (*P* < 0.0001). No arrhythmia (33.90%) and simple ventricular arrhythmia (17.62%) were the most common diagnoses (*P* < 0.0001). There were no linear relationships between either age or weight and AEG indication or diagnosis.

Within breeds, syncope/collapse was the most common indication for AEG in boxers (*P* < 0.0001), cavalier King Charles spaniels (CKCS) (*P* = 0.005), Cocker spaniels (*P* = 0.001), crossbreeds (*P* = 0.033), Labradors (*P* = 0.005), sheepdogs (*P* = 0.002) and West Highland white terriers (WHWTs) (*P* = 0.035) but arrhythmias on auscultation were also over‐represented in Labradors (*P* < 0.0001) and German shepherds (*P* = 0.002). No arrhythmia was the most common AEG diagnosis in boxers (*P* < 0.0001), Cocker spaniels (*P* < 0.0001), crossbreeds (*P* < 0.0001), German shepherds (*P* = 0.024), golden retrievers (*P* = 0.010), Labradors (*P* = 0.001) and sheepdogs (*P* = 0.005). Simple ventricular arrhythmias were also over‐represented in boxers (*P* < 0.0001), crossbreeds (*P* = 0.002) and great Danes (*P* = 0.034), whereas persistent atrial fibrillation was over‐represented in Labradors (*P* = 0.007) and sinus node dysfunction in WHWTs (*P* = 0.031). Dogs with structural cardiac disease were more likely to have an abnormal rhythm than those without structural cardiac disease (*P* = 0.001).

In our population, one third of AEGs found no arrhythmia even though syncope/collapse and a previously detected arrhythmia were the commonest indications for performing them. Both AEG indications and diagnoses were breed‐dependent but independent of age and weight. Our study supports identifying structural heart disease as an important component of arrhythmia assessment in combination with AEG.

**Disclosures**

All authors employed by the University of Edinburgh. Geoffrey Culshaw: Research funded by Kidney Research UK and Petsavers Rachel Blake: Receives consulting fees from University College Dublin and IDEXX

## ESVC‐O‐8

### ESVC—European Society of Veterinary Cardiology

#### Transvenous closure of patent ductus arteriosus (PDA) with Nit‐Occlud® occlusion system in nine dogs and one cat with a body weight less than 3 kg

##### A. Cala^1,2^, L. Ferasin^1,2^, O. Domenech^3^, M. Bini^3^, V. Valenti^4^, L. Venco^4^

###### ^1^Specialist Veterinary Cardiology Consultancy Ltd., Four Marks GU34 5AA, UK; ^2^The Ralph Veterinary Referral Centre, Marlow SL7 1YG, UK; ^3^Anicura Istituto Veterinario Novara, Granozzo con Monticello, 28060 Novara, Italy; ^4^Ospedale Veterinario Città di Pavia, 27100 Pavia, Italy

Closure of PDAs in small dogs and cats is challenging and can be achieved via surgical ligation or with a limited number of minimally‐invasive occlusion techniques. However, the use of a transvenous Nit‐Occlud PDA coil, already described in human cardiology, has not yet been reported in veterinary medicine.

Nit‐Occlud® PDA is made of a conic‐shaped nitinol spiral, where the windings positioned in the ductal ampulla taper gradually towards the pulmonary side. This conformation increases the stability of the device, preventing a “pull‐through” into the pulmonary artery. The device is pre‐mounted to a delivery cable, which allows device repositioning prior to its release. Once deployed, the coils at pulmonary side wind in a reverse conical direction, increasing device's diameter and promoting better anchoring. Due to these characteristics, this device appears ideal for the closure of small PDAs in dogs and cats. The aim of this study is to describe safety and efficacy of Nit‐Occlud® in dogs and cats.

We retrospectively reviewed the clinical records of 10 dogs and 1 cat that underwent PDA occlusion with a Nit‐Occlud® in 2021 and 2022 at three referral institutions. The median age of these patients was 6 months (range 2.5–38), with a mean body weight of 2.65 ± 0.26 kg. The mean minimum ductal diameter (MDD) was 1.90 ± 0.45 mm, while the mean ampulla diameter (AD) was 5.61 ± 2.00 mm. The Nit‐Occlud® device size was determined on echocardiographic measurement of MDD and AD, with the distal diameter being at least 3 mm larger than MDD but not more than 2 mm larger than the AD. Vascular access was obtained percutaneously from the right jugular vein. Following catheterization of the PDA, the distal windings were positioned in the aorta and retrieved into the ampulla. The distal loop was positioned in the pulmonary side before releasing the device. No complications were reported.

PDA closure was successfully achieved in 10 cases, with a mean procedure time of 57 ± 24 min. Trivial or no residual shunt was observed on transthoracic echocardiography in 10 cases prior to device deployment while, in one dog, the device was not released due to unsatisfactory occlusion. Patients underwent a follow‐up echocardiographic examination 2–5 months after the procedure, which showed complete ductal closure and reversed cardiac remodelling in all cases.

This study suggests that closure of PDA with Nit‐Occlud® is safe, feasible and highly successful in small dogs and cats weighing less than 3 kg.

**Disclosures**

No disclosures to report

## ESVC‐O‐9

### ESVC—European Society of Veterinary Cardiology

#### Tetranectin as a potential biomarker for feline hypertrophic cardiomyopathy

##### M M Liu^1,2^, W S Ding^1^, X N Chen^1^, Y Qin^1,2^, H Zhang^1,2^

###### ^1^School of Animal Science and Technology, Hainan University, Haikou, Hainan, China; ^2^One Health Institute, Hainan University, Haikou, Hainan, China

Tetranectin is a C‐type lectin which can enhance plasmin activation and be associated with extracellular remodelling in a variety of tissues. In humans tetranectin showed better performance in identifying severe heart failure than traditional cardiac biomarker NT‐proBNP. A previous proteomic study found circulating tetranectin changed in cats with primary cardiomyopathies, but whether this protein present in cat myocardium was not clear. The aim of this study is to evaluate tetranectin expression in feline hypertrophic cardiomyopathy (HCM) both at serum and myocardium levels.

Archived surplus serum samples from 18 client‐owned cats were used for circulating tetranectin study. For myocardium study, heart samples were collected from nine cats with unexpected death or euthanatized due to poor disease condition. Animal information, echocardiography data and other relevant clinical records were collected. Expression of tetranectin in cat serum and myocardium was detected by using immunoblotting, immunohistochemistry and RT‐PCR.

Circulating tetranectin level was significantly higher in preclinical HCM cats compares to controls and also to its CHF counterparts (*P* < 0.05), suggesting tetranectin might be a dynamic biomarker for HCM. Both tetranectin protein and gene expressions were detected in cat myocardium samples. Interestingly different immunoblotting patterns of tetranectin were observed between myocardium and serum samples, indicating tetranectin may have additional function in cat myocardium. When examining left ventricle myocardium from HCM cats, tetranectin was found localized in cardiomyocytes and cardiac fibroblasts, however whether these cells intake tetranectin from circulation or produce tetranectin by themselves remain unknown.

This is first report showing tetranectin expression in myocardium of cat and this protein holds potential to be a biomarker of feline HCM. Future work is warranted to determine the exact role of tetranectin in feline hypertrophic cardiomyopathy.

**Disclosures**

This project is funded by New Ruipeng Young Scholar Grant (Small Animal Clinical Research).

**Source of Funding**

New Ruipeng Pet Group Inc and New Ruipeng Charity Foundation.

## ESVC‐O‐10

### ESVC—European Society of Veterinary Cardiology

#### Evaluation of the circulating renin‐angiotensin‐aldosterone system in healthy young dogs and dogs with right‐sided congenital heart disease

##### KV Pierce^1^, MK Ames^2^, CE Atkins^1^

###### ^1^North Carolina State University, Raleigh, USA; ^2^UC Davis School of Veterinary Medicine, Davis, USA

It has been well described in human and veterinary medicine that activation of the renin‐angiotensin‐aldosterone system (RAAS) contributes to cardiovascular and kidney disease progression. RAAS activation has been studied in older dogs with myxomatous mitral valve disease (MMVD). The use of RAAS‐suppressant drugs is considered part of the standard of care in treating dogs with congestive heart failure secondary (CHF) to MMVD. Currently, no studies have been performed evaluating dogs with spontaneously occurring right‐sided congenital heart defects, such as pulmonary valve stenosis (PS) or tricuspid valve dysplasia (TVD). There is no consensus on whether to start RAAS modulating medications prior to the development of right‐sided CHF, which tends to be clinician‐dependent. Better understanding the RAAS profile in dogs with right‐sided congenital heart defects is the first step toward optimizing therapeutic interventions and outcomes for these dogs. The aim of this study was to define RAAS profiles in young healthy dogs to provide control data and to determine if RAAS is activated in right‐sided congenital heart disease (PS, TVD).

Fourteen dogs with TVD, 18 dogs with severe PS (PG > 80 mmHg), and 22 age‐ and weight‐matched heathy controls were enrolled in this prospective study. Diagnostics performed included physical examination, blood pressure, complete blood count, chemistry profile, urinalysis, urine aldosterone concentration, echocardiogram, and a serum sample for RAAS profile. Serum samples for renin‐angiotensin system (RAS) fingerprint (RAAS profile) and urine samples for urine aldosterone concentrations were stored in −80**°**C freezer and batched. Dogs were excluded if concurrent systemic illnesses were present or if receiving medications that modulate RAAS.

RAAS profiles including serum angiotensin I, II, III, IV, (1,7), and (1,5) aldosterone concentrations and the composite markers Ang II/Ang I Ratio—angiotensin converting enzyme‐surrogate (ACE‐S), [Ang I + Ang II]—plasma renin activity‐surrogate (PRA‐SA), Aldosterone/Ang II Ratio—adrenal response‐surrogate (AA2), Alternative RAS/Total RAS—proportion of alternative RAS activation (ALT‐S = Ang1‐5+1‐7/Ang1‐5+1‐7+AngI+AngII) were established. Reference intervals were determined for the healthy young dog control group and compared to TVD and severe PS groups. There were differences in the expression of the components of the RAS fingerprint between the healthy control group, TVD group, and severe PS group. This study provides reference ranges for RAAS profiles in young dogs and dogs with right‐sided congenital heart defects, which is a step toward optimizing medical management and outcomes for these dogs.

**Disclosures**

Dr. Pierce is a speaker and consultant for Ceva Sante Animale; Dr. Atkins is a consultant for Ceva Sante Animale, Vetoquinol, and Boehringer Ingelheim; Dr. Ames is a consultant for Ceva Sante Animale and Elanco. No funding or financial support was received from any of the companies listed. Funding was provided via grants from ACVIM and NCSU CVM.

**Source of Funding**

Funding was provided via two grants: American College of Veterinary Internal Medicine (ACVIM) Diplomate Research grant and the North Carolina State University College of Veterinary Medicine (CVM) Intramural grant.

## ESVC‐O‐11

### ESVC—European Society of Veterinary Cardiology

#### Assessment of heart rate in dogs with atrial fibrillation: Are two days better than one?

##### J Escalda^1^, B Pedro^2^, J Novo Matos^1^, A Mavropoulou^3^, J Dukes‐McEwan^4^, J Neves^2^, C Linney^2^, A Gelzer^5^

###### ^1^University of Cambridge, Cambridge, UK; ^2^Willows Veterinary Centre and Referral Service, West Midlands, UK; ^3^Plakentia Veterinary Clinic, Athens, Greece; ^4^University of Liverpool, Neston, UK; ^5^University of Pennsylvania Philadelphia, Pennsylvania, USA

The 24‐h Holter derived mean heart rate (meanHR) is considered the gold standard for assessing ventricular response rate in dogs with atrial fibrillation (AF). However, daily variation in meanHR of dogs with AF, and how this may affect clinical decision making, has not been fully investigated.

We aimed to compare the meanHR in dogs with AF during 24‐h versus 48‐h Holter monitoring, to assess if longer heart rate monitoring in AF has an impact on clinical management and to assess between‐day variation in meanHR. We hypothesised that assessment of meanHR in dogs with AF during a 48‐h Holter is equivalent to 24‐h Holter monitoring.

Dogs enrolled in this study were part of a multicentred, prospective, longitudinal study. Only dogs with controlled congestive heart failure and a 48‐h Holter at the first visit after starting anti‐arrhythmic therapy were included. MeanHR on day 1 (24‐Holter1), day 2 (24‐Holter2) and over 48 h (48‐Holter) were analysed separately. A meanHR <125 bpm was considered to reflect optimal rate control; a difference in meanHR >10 bpm between days was considered clinically significant. Daily variability in frequency of ventricular premature complexes (VPCs) was also analysed. Data are presented as median [range] or mean (± standard deviation).

Twenty‐five dogs were included. There was no difference in meanHR between 24‐Holter1 versus 48‐Holter (141.8 bpm (±31.80) vs. 141.6 bpm (±32.24), *P* = 0.83) and 24‐Holter1 versus 24‐Holter2 (141.8 bpm (±31.80) vs. 141.4 bpm (±33.1) *P* = 0.83). Between‐day variation in meanHR was low (ΔmeanHR^Day1vs48hrs^ 3 bpm (±2.01), ΔmeanHR^day1vsday2^ 5 bpm (±4.03), coefficient of variation 24‐Holter1 vs. 24‐Holter2 was 3.2%). Bland‐Altman analysis showed excellent agreement on meanHR between 24 Holter1 versus 48‐Holter (bias 0.16 ±3.7, 95% limits of agreement [−7,7]). 2/25 (8%) dogs had clinically significant differences in meanHR between 24‐Holter1 versus 48‐Holter, where rate control success would have been classified differently. There was no difference in number of VPCs between days (23 VPCs [0–54618] 24‐Holter1 vs. 26 VPCs [0–56857] 24‐Holter2, *P* = 0.65). Daily variability in VPCs frequency was 51.4% (±33.4).

This study suggests that 24‐h derived meanHR is equivalent to 48‐h meanHR, suggesting no clinical advantage in performing longer Holter monitoring in dogs with AF. Daily variability in VPCs frequency in AF is similar to that previously reported in dogs.

**Disclosures**

No disclosures to report

## ESVC‐O‐12

### ESVC—European Society of Veterinary Cardiology

#### Prevalence and progression of azotemia during treatment of congestive heart failure in cats

##### S Rogg^1^, JL Ward^1^, D Kundu^1^, JP Mochel^1^, MA Tropf^1^, AK Masters^2^, DB Adin^3^

###### ^1^Iowa State University, Ames, USA; ^2^University of Minnesota College of Veterinary Medicine, Minneapolis, USA; ^3^University of Florida College of Veterinary Medicine, Gainesville, USA

Cats diagnosed with congestive heart failure (CHF) often present with pre‐existing azotemia (creatinine ≥1.6 mg/dL), which can worsen after furosemide treatment due to acute kidney injury or exacerbation of underlying chronic kidney disease. It remains unknown how common azotemia is before or after treatment of CHF in cats, and what risk factors might predict its progression.

Medical records of 116 client‐owned cats with CHF presenting to a referral veterinary teaching hospital over a 12‐year period were evaluated. Inclusion criteria were an echocardiographic diagnosis of heart disease, clinical and radiographic evidence of CHF, and renal values and electrolytes performed at least twice after diagnosis of CHF. Clinical and echocardiographic variables were recorded at the time of CHF diagnosis. Subsequent measurements of renal values and electrolytes were recorded and correlated to clinically relevant timepoints, and furosemide dosage administered between timepoints was calculated. An analysis of variance was performed to assess for changes in renal values over time, while Kruskal‐Wallis rank‐sum and Chi‐square testing were performed to assess for pairwise changes in renal values and occurrence of azotemia between clinical timepoints.

Median creatinine value prior to furosemide treatment was 1.5 mg/dL (interquartile range [IQR] 1.0–2.1 mg/dL), with 44% of cats being azotemic at baseline. During initial hospitalization and treatment with a median furosemide dosage of 2.0 mg/kg/day, median creatinine increased to 2.2 mg/dL (IQR 1.7–2.8 mg/dL). At the first outpatient recheck visit, median creatinine had returned to 1.5 mg/dL (IQR 1.2–2.1 mg/dL). At subsequent recheck periods up to 12 months from initial diagnosis, median creatinine increased to 1.8 mg/dL (IQR 1.4–2.3 mg/dL); during this time, furosemide dose progressively decreased from 1.50 mg/kg/day (within 1 month of diagnosis) to 1.32 mg/kg/day (12 months after diagnosis). Creatinine increased over time (*P* < 0.001) and was significantly different from pre‐furosemide values at all subsequent timepoints (*P* < 0.02 for all pairwise comparisons), except for the first outpatient recheck visit. The proportion of cats with azotemia increased over time from 44% (pre‐furosemide) to 69% (12 months after diagnosis; *P* = 0.0097). Kidney injury (increase in creatinine of ≥0.3 mg/dL) was documented in 46% of cats during hospitalization and in 26%–47% of cats during subsequent outpatient rechecks within 12 months.

Pre‐existing azotemia and progressive kidney injury were common during treatment of CHF in cats despite decreasing furosemide dosage over time. These results have potential implications for clinical management and furosemide dosing in cats with CHF.

**Disclosures**

Drs. Ward and Mochel have received consulting fees and honoraria from Ceva Santé Animale. Dr. Adin acknowledges research support from Nestle Purina PetCare and is a consultant and sponsored lecturer for Ceva Animal Health and Boehringer Ingelheim. No other authors have a conflict of interest.

## ESVC‐O‐13

### ESVC—European Society of Veterinary Cardiology

#### Clinical efficacy and tolerability of oral amiodarone and sotalol in dogs with tachyarrhythmias

##### R Romito, M Mazzoldi, D Dondi, F. Fasoli, C Cipone

###### University of Bologna, Ozzano dell'Emilia, Italy

Amiodarone and sotalol are frequently used in canine tachyarrhythmias; however, studies assessing the frequency of possible related adverse effects (PAEs) are limited in dogs. Therefore, the aim of this study was to provide data on the clinical efficacy and tolerability of amiodarone and sotalol in tachyarrhythmic dogs.

For the purpose of this retrospective cohort study, dogs with tachyarrhythmias treated with oral amiodarone (A‐dogs) or sotalol (S‐dogs) were searched in the medical database of one veterinary hospital. Signalment, historical, clinical, clinicopathological (including T4 and TSH), radiographic, electrocardiographic (including 6‐/12‐lead ECG and 24‐hour Holter), echocardiographic, therapeutic and outcome data were retrieved. Dogs were enrolled only if data were available at least at two time points (i.e., pre‐ and post‐therapy). For both ventricular (VT) and supraventricular tachyarrhythmias (SvT), efficacy was assessed by investigating the arrhythmias severity (classified according to the Lown‐Wolf grading system [LW‐gs], ranging from 0 to 5) before and after prescribing the antiarrhythmics. In dogs with atrial fibrillation (AF), efficacy was assessed by investigating the mean heart rate (mHR) before and after prescribing the antiarrhythmics. Concerning PAEs, both in A‐dogs and S‐dogs, attention was paid to the development of selected clinical (weakness, hypotension, gastrointestinal signs) and electrocardiographic (QT‐interval prolongation, atrioventricular blocks [AVB]) signs. In A‐dogs, development of laboratory results suggestive of liver injury, hypothyroidism and cytopenia were also assessed. In S‐dogs, development of systolic dysfunction was also recorded.

Sixty‐four dogs were enrolled: 45 with VT, 9 with SvT and 10 with VT+SvT. A‐dogs and S‐dogs were 24 and 40, respectively. Concerning triggers of tachyarrhythmias, heart disease, systemic diseases or both were documented in 33, 14 and 17 dogs, respectively. According to the LW‐gs, the severity of VT and SvT decreased after prescribing the antiarrhythmics both in A‐dogs (before: median value 5 [range: 4–5]; after: 3 [1–4]) and S‐dogs (before: 5 [4–5]; after: 3 [1–5]). In dogs with AF, the mHR decreased after prescribing the antiarrhythmics both in A‐dogs (before: 220 [215–240] beats/minute; after: 144 [135–145] beats/minute) and S‐dogs (before: 215 [196–220] beats/minute; after: 133 [111–140] beats/minute). In A‐dogs, PAEs included decreased T4 combined with increased TSH (*n* = 2), QT‐interval prolongation (*n* = 2), diarrhea (*n* = 1), anemia (*n* = 1) and neutropenia (*n* = 1). In S‐dogs, PAEs included weakness (*n* = 2), second‐degree AVB (*n* = 2), hypotension (*n* = 1) and first‐degree AVB (*n* = 1). Neither clinicopathological nor electrocardiographic PAEs were associated with clinical signs. All PAEs resolved after antiarrhythmic discontinuation.

In tachyarrhythmic dogs, oral amiodarone and sotalol are effective and usually safe drugs.

**Disclosures**

No disclosures to report.

## ESVC‐O‐14

### ESVC—European Society of Veterinary Cardiology

#### Incidence of restenosis and associated risk factors in dogs undergoing balloon valvuloplasty for pulmonic stenosis

##### S Goodrich^1^, J Loureiro^1^, Y Martinez‐Pereira^2^, J Novo Matos^3^, L Gardner^3^, T Sparks^4^, J Silva^1^

###### ^1^North Downs Specialist Referrals, Bletchingley, UK; ^2^The Royal (Dick) School of Veterinary Studies, University of Edinburgh, Edinburgh, UK; ^3^Department of Veterinary Medicine, University of Cambridge, Cambridge, UK; ^4^Waltham Petcare Science Institute Melton, Mowbray, UK

Balloon valvuloplasty (BVP) is the treatment of choice for severe pulmonic stenosis (PS) in dogs. Restenosis is a reported complication following BVP for which there is only limited information available. The aims of this study are to report BVP outcomes and the incidence and associated risk factors for restenosis.

Clinical records from three referral centres were reviewed and dogs with severe PS undergoing BVP between 2008 and 2022 were included. Dogs with incomplete records, significant concurrent cardiac or systemic diseases and/or in congestive heart failure were excluded.

Procedure outcome was assessed at initial follow‐up (1‐month post‐BVP) and classified as successful, partial improvement or persistent stenosis, as follows: successful if there was an improvement of pulmonary pressure gradient (PG) to <80 mmHg; partial improvement if there was an improvement but PG remained ≥80 mmHg; persistent stenosis if there was no improvement. An improvement was defined as reduction in PG by at least 33.9 mmHg or in pulmonary maximal velocity (PAVmax) by at least 0.87 m/s. Restenosis was defined as an increase in PG by at least 33.9 mmHg or PAVmax by at least 0.87 m/s, between the initial follow‐up and a subsequent examination. Binary logistic regression was performed to assess risk factors associated with restenosis. The following variables were considered: age, breed, pre‐BVP PG, PS type, procedure outcome, balloon:annulus ratio, and balloon type.

One hundred and fifteen dogs were identified with data on pre‐BVP, immediately post‐BVP, and initial follow‐up. Of these, 73/115 (63%) dogs were classified as successful; 15/115 (13%) dogs as partial improvement; and 23/115 (23%) dogs as persistent stenosis. There was a significant difference in procedure outcomes between Type A and Type B PS, with an increased proportion of Type B PS classified as persistent stenosis (*P* = 0.008). Seventy‐six dogs had long‐term follow‐up data available (at least 1 subsequent examination after initial follow‐up). Restenosis was observed in 13/76 (17%) dogs at a median of 417 days [91–2137] after initial follow up. Logistic regression showed an association between Type B PS and restenosis (odds ratio 7.13, 95% confidence interval 1.7–30.2). No other risk factors were identified.

This study suggests that dogs with Type B PS have a lower BVP success and a higher incidence of restenosis. Incidence of restenosis was similar to previous publications.

**Disclosures**

No disclosures to report

## ESVC‐O‐15

### ESVC—European Society of Veterinary Cardiology

#### Do practicing veterinarians follow the ACVIM consensus guidelines for staging myxomatous mitral valve degeneration in dogs?

##### V Szatmári, E Muis, M van Staveren Utrecht University, Utrecht, Netherlands

###### The most recent guidelines on diagnosis and management of myxomatous mitral valve degeneration were published in 2019. The present study aimed to investigate how closely first opinion veterinary practitioners follow the staging recommendations.

Digital questionnaires with multiple choice and open questions were sent out to veterinary practices via newsletters of various professional organizations. Participation was voluntary. Only the 363 fully completed questionnaires were analyzed of the 524 responses.

Three quarters of the respondents find at least one adult dog a week with a newly recognized murmur. The ACVIM Consensus Guidelines were used by only 60% of the respondents. The two main reasons for not using the staging system were difficulties to identify the stages (53%) and unawareness of the existence of the guidelines (23%).

Regarding the ease of differentiating stage B1 from B2, 43% found this distinction difficult, whereas for 29% it was easy and 28% replied with neutral. For differentiation, 36% of the respondents used echocardiography, 16% thoracic radiographs, 24% both and 17% did not differentiate these stages. The reason for not differentiating B1 from B2 were financial constraints of the owner (55%) or the veterinarians’ perception that the stage would not matter (21%).

Regarding diagnosing stage C, 67% of the respondents found its detection easy, while 10% found it difficult, and 23% responded with neutral. The most fitting findings of pulmonary oedema in the history were thought to be dyspnoea (93%), followed by exercise intolerance (90%), cough (67%), collapse (27%), lack of appetite (21%) and weight loss (9%). Regarding characteristic physical examination findings for pulmonary oedema, dyspnoea was chosen by 75% of the respondents, tachycardia by 54%, weak femoral pulses by 31%, increased lung sounds by 60%, decreased lung sounds by 26%, a horizontal damping line by 28%. To confirm pulmonary oedema, 98% of the respondents would take thoracic radiographs.

The most striking finding of this study was that 40% of the respondents do not use the proposed staging system for myxomatous mitral valve disease. The most likely reason for this is limited availability and/or expertise in echocardiography.

**Disclosures**

No disclosures to report

## ESVC‐O‐16

### ESVC—European Society of Veterinary Cardiology

#### The Pulmonary hypertension Remodeling/hemodynamic‐Induced Manifestations on Echocardiography (PRIME) score for predicting the severity of canine pulmonary hypertension

##### WT Chang^1^, L García‐Guasch^2^, IP Chan^3^, JA Montoya‐Alonso^4^, JI Matos^4^, PY Lo^5^, MC Lam^5^, CH Lin^1^

###### ^1^National Taiwan University, 2.TACS‐Alliance Research Center, Taipei, Taiwan; ^2^IVC Evidensia Hospital Veterinari Molins & Hospital Veteriaria del Mar, Barcelona, Spain; ^3^National Chung Hsing University, Taichung, Taiwan; ^4^University of Las Palmas de Gran Canaria, Las Palmas, de Gran Canaria, Spain; ^5^TACS‐Alliance Research Center, Taipei, Taiwan

Peak tricuspid regurgitation velocity (pTRV) is typically used to estimate pulmonary arterial pressure in dogs but is not always available. Echocardiographic remodeling changes have been proposed to assess the probability of canine pulmonary hypertension (CPHT). This study aimed to develop a composition score from routinely acquired echocardiographic images for predicting CPHT severity.

This multicenter study included 118 dogs; 59 echocardiographs were retrospectively reviewed to generate a weighted scoring system for predicting pTRV by multiple linear regression and 59 echocardiographs were used for validation. The accuracy for discriminating CPHT severity was evaluated using the area under the receiver operating characteristic curve (AUC).

A 25‐point weighted pulmonary hypertension remodeling/hemodynamic‐induced manifestations on echocardiography (PRIME) score was established, comprising semiquantification of right ventricular (RV) wall thickening (0/1/2 points), RV dilation (0/2/4/6 points), right atrial enlargement (0/2/4/6 points), pulmonary artery enlargement (0/2/4/6 points), intraventricular septum flattening (0/2/4 points), and midsystolic notching of the RV outflow profile (0/1 point).

Moderate (pTRV ≥ 3.4 m/s) and severe (pTRV ≥ 4.3 m/s) CPHT could be correctly predicted in 78% cases in the validation group with cutoff values of 4 and 9, respectively. Prediction further improved to 89% after excluding 12 cases with significant left atrial enlargement (echocardiographic left atrial/aorta ratio ≥ 2.0). Overall accuracy for predicting moderate‐to‐severe CPHT was excellent (AUC, 0.973; sensitivity, 87.0%; specificity, 91.7%). The PRIME score was positively correlated with pTRV (*r*_s_ = 0.89, *P <* 0.001).

Thus, the PRIME score can adequately predict CPHT severity, especially in non‐left heart disease cases.

**Disclosures**

A part of this study was supported by National Taiwan University, the award from TACS‐Alliance Research Center, and the grant from National Science and Technology Council, Taiwan

**Source of Funding**

NSTC 111‐2313‐B‐002‐062‐/TACS‐A 2023 Award / Support from National Taiwan University

## ESVC‐O‐17

### ESVC—European Society of Veterinary Cardiology

#### The Mitral INsufficiency Echocardiographic (MINE) score in dogs with preclinical myxomatous mitral valve disease

##### T Vezzosi^1^, G Grosso^1^, L Vatne^2^, F Porciello^3^, E Dall'Aglio^4^, C Guglielmini^5^, E Broch^6^, D Dickson^7^, M Croce^8^, V Patata^8^, F Marchesotti^8^, R Tognetti^1^, M Rishniw^9^, O Domenech^8^

###### ^1^University of Pisa San Piero a Grado, Pisa, Italy; ^2^Anicura Oslo Animal Hospital, Oslo, Norway; ^3^University of Perugia, Perugia, Italy; ^4^Clinica Veterinaria Milano Sud Peschiera Borromeo, Milano, Italy; ^5^University of Padua, Padova, Italy; ^6^Anicura Clinica Veterinaria CMV Varese, Varese, Italy; ^7^HeartVets, Porthcawl, UK; ^8^Anicura Istituto Veterinario Novara, Novara, Italy; ^9^College of Veterinary Medicine, Cornell University Ithaca, New York, USA

The ACVIM guidelines are used for the clinical classification of dogs with myxomatous mitral valve disease (MMVD). The Mitral INsufficiency Echocardiographic (MINE) score has been described as an easy‐to‐use echocardiographic severity classification of MMVD, proven to be clinically effective since associated with survival. The aim of this study was to test the MINE score in a sample of dogs with preclinical MMVD larger than in the original study, also evaluating a possible simplification of the score in this stage.

Clinical usefulness of the MINE score was tested by evaluating its association with the median time to cardiac event, defined with the occurrence of congestive heart failure or cardiac death. Long‐term outcome was assessed by telephone interviews with the owners. Survival was analysed using Kaplan‐Meier curves, logrank tests and Cox's proportional hazards.

This retrospective, multicentre observational study included 749 dogs with preclinical MMVD imaged between 2011 and 2021, of which 200 (27%) developed a cardiac event according to an updated follow‐up in December 2022. The sample included 374 dogs in stage B1 and 375 in stage B2. Based on the multivariate analysis, the left atrium‐to‐aorta ratio (LA/Ao) [hazard ratio(HR) = 1.78; 95%CI 1.07–2.99; *P* = 0.03], the left ventricular end‐diastolic diameter normalized for body weight (LVIDDn) (HR = 6.54; 95%CI 2.87–14.86; *P* < 0.001) and the E‐wave transmitral peak velocity (E‐vel) (HR = 2.98; 95%CI 1.54–5.76; *P* = 0.001) were predictors of a cardiac event; the fractional shortening (FS%) was not (*P* = 0.06). Thus, a simplified version of the MINE score was redefined only including LA/Ao, LVIDDn and E‐vel, still maintaining the original cut‐offs and the severity classes: mild, moderate, severe and late‐stage. The median time to cardiac event was significantly different (*P* < 0.001) between the severity classes: mild [2604 days, 95% confidence interval (CI) 2344–2604 days], moderate (1216 days, 95%CI 998–1882 days) and severe (718 days, 95%CI 599–980 days). Among stage B1, no differences in median time to cardiac event were found between mild and moderate cases (*P* = 0.238). Among stage B2, severe cases had a shorter median time to cardiac event (718 days, 95% CI 599–980 days) in comparison to moderate cases (1141 days, 95% CI 980–1725 days) and mild cases (not available; *P* < 0.001).

In conclusion the simplified version of the MINE score is clinically effective for a severity classification of preclinical MMVD and can be useful for risk‐stratification in this stage. Dogs in stage B2 classified as severe according to the MINE score could represent a proposal for the definition of an “advanced” B2 stage.

**Disclosures**

No disclosures to report

## ESVC‐O‐18

### ESVC—European Society of Veterinary Cardiology

#### Left atrial rupture secondary to myxomatous mitral valve disease in 33 dogs (2017–2022)

##### S Wong, S Siess, G Kramer

###### Atlantic Coast Veterinary Specialists, Bohemia, USA

A rare complication of canine myxomatous mitral valve disease (MMVD) is left atrial rupture (LAR), which can lead to life‐threatening cardiogenic shock and death. This retrospective case series aims to describe the clinical parameters, echocardiographic findings, treatments, and prognosis of LAR secondary to MMVD. Medical records, thoracic radiographs and echocardiograms of dogs with suspected LAR at a single referral center from January 2017 to September 2022 were reviewed. Thirty‐three dogs with characteristic valvular lesions, mitral regurgitation and evidence of pericardial effusion (*N* = 31) and/or pericardial/intracardiac thrombus (*N* = 18) were included. Twenty‐five dogs had radiographic evidence of left sided congestive heart failure (LCHF), 12 were in cardiac tamponade (4 received pericardiocentesis), and 15 were treated with pimobendan prior to the LAR event. Mean LA:Ao was 2.59 (SD = 0.44), LVIDdN (*M* = 1.90, SD = 0.68), FS (*M* = 49.65%, SD = 8.26), MV E‐vel (*M* = 1.21 m/s, SD = 0.30), MINE score (*M* = 9.84, SD = 1.11). Twenty‐seven dogs were discharged; at the time of analyses 8 dogs were still alive. Mean survival time was 238.96 days (Median = 108.0, SD = 354.09, range 1.0–1191.0) for the deceased, and 467.88 days (Median = 299.0, SD = 411.48, range 193.0–1387.0) for those still alive. Multiple simple linear regressions conducted indicated that LCHF, echocardiographic variables, and MINE score did not significantly predict survival time after LAR (Ps > 0.05). This study suggests short‐term mortality for LAR secondary to MMVD is not as high as previously believed.

**Disclosures**

No disclosures to report

## ESVC‐O‐19

### ESVC—European Society of Veterinary Cardiology

#### Ultrasonographic assessment of abdominal aortic flow to evaluate hemodynamic significance of left‐to‐right shunting patent ductus arteriosus in dogs

##### A Van de Watering^2^, G Santarelli^1^, S Van Rossem^1^, M Baron Toaldo^3^, A Hulsman^1^, NJ Beijerink^4^, V Szatmari^1^

###### ^1^Utrecht University, Utrecht, Netherlands; ^2^ Utrecht University, Utrecht, Netherlands; ^3^Vetsuisse Faculty, Zurich, Switzerland; ^4^Veterinaire specialisten, Vught, Netherlands

Patent ductus arteriosus (PDA) is a common congenital heart disease in dogs, and can cause congestive heart failure and death if left‐to‐right shunting is substantial. In humans, hemodynamic significance of PDAs is evaluated through echocardiography and ultrasonographic assessment of the descending aortic flow to establish need for closure. Retrograde or absent end‐diastolic flow usually indicates a hemodynamically significant PDA (hsPDA). The purposes of this study were to describe the aortic flow patterns in dogs without cardiac disease and dogs with left‐to‐right shunting PDA, and to establish their accuracy to detect hsPDA.

In this multicenter, prospective, observational study, a control group of apparently healthy dogs without echocardiographic abnormalities and a group of dogs with left‐to‐right shunting PDA were examined. Dogs with PDA that underwent ductal closure were re‐examined after the procedure. All dogs received a complete echocardiogram and duplex Doppler ultrasonographic assessment of abdominal aortic flow using pulsed‐wave Doppler on the aortic segment between the renal arteries and aortic trifurcation. In dogs with PDA, the hemodynamic significance of the shunt was estimated based on: left atrium to aorta ratio; left ventricular internal diameter in diastole, normalized for body weight; left ventricular volume in diastole, indexed to body surface area. The PDA was classified as hsPDA if one or more of these variables were above accepted limits of normality.

A total of 15 control dogs and 28 dogs with left‐to‐right shunting PDA were included. In the PDA group, 21/28 dogs were diagnosed with hsPDA and 7/28 with non‐hsPDA. In 4/21 dogs with hsPDA, 2/7 dogs with non‐hsPDA and 2/15 control dogs, tachycardia prevented assessment of end‐diastolic aortic flow. Anterograde end‐diastolic flow was observed in 12/15 control dogs and 3/7 dogs with non‐hsPDA. Absent end‐diastolic flow was observed in 1/15 control dogs, 1/21 dogs with hsPDA and 2/7 dogs with non‐hsPDA. Retrograde end‐diastolic flow was observed only in dogs with hsPDA (16/21). Six dogs with hsPDA and 2 dogs with non‐hsPDA were reassessed after successful PDA closure and all showed an anterograde end‐diastolic flow. Sensitivity and specificity of retrograde end‐diastolic flow to detect hsPDA in the absence of tachycardia were 94% and 100%, respectively; for absent diastolic flow, these were 5% and 40%, respectively.

In conclusion, duplex Doppler ultrasonographic assessment of abdominal aortic flow was feasible in all dogs. However, assessment of end‐diastolic flow was precluded by tachycardia in some cases. A retrograde end‐diastolic flow allowed accurate discrimination of hsPDAs and non‐hsPDAs.

**Disclosures**

No disclosures to report

## ESVC‐O‐20

### ESVC—European Society of Veterinary Cardiology

#### Timing and patterns of resolution of lung ultrasound B‐lines compared to lung auscultation and respiratory rates in hospitalized dogs with cardiogenic pulmonary edema

##### C McLaughlin^1^, T DeFrancesco^1^, J Ward^2^, M Pabon^2^

###### ^1^North Carolina State University, Raleigh, USA; ^2^Iowa State University College of Veterinary Medicine, Ames, USA

Together with clinical and radiographic findings, lung ultrasound (LUS) has become an important tool in the early recognition and expeditious treatment of cardiogenic pulmonary edema. However, only limited information as to the utility of LUS for in‐patient monitoring of the resolution of pulmonary edema exists in veterinary medicine. In addition to serial thoracic radiographs, in‐hospital monitoring of the resolution of pulmonary edema is often based on monitoring respiratory rate, respiratory effort and serial lung auscultation. In most cases pulmonary crackles are considered characteristic, though not specific, for pulmonary edema. Auscultation is also a subjective assessment dependent on experience and skill of the clinician. There is a strong interest in the development of techniques for cardiopulmonary exam that are more sensitive and objective than traditional auscultation. It has been suggested that LUS can function as a “visual stethoscope” to supplement auscultatory findings and might improve diagnostic accuracy of the physical examination. The primary aim of this study was to describe the pattern and time course in the resolution of B‐lines in dogs hospitalized for treatment of left‐sided congestive heart failure (CHF). The secondary aim of this study was to compare the lung ultrasound findings in patients with left‐sided CHF with traditional monitoring by auscultation and respiratory rate. Twenty‐five dogs presenting for left‐sided CHF of any cause were prospectively enrolled (23 had degenerative mitral valve disease, and two had dilated cardiomyopathy). Lung auscultation, LUS findings and respiratory rates were recorded within the first 2 hours of presentation and then at 3, 6, 12 and 24 h later. Lung auscultation and LUS were performed using the the VetBLUE protocol sites. The anonymized LUS video clips were archived and evaluated later by a blinded investigator who quantified the number of B‐lines present at each site. Radiographs were also performed at or before presentation and 24 hours later. The pattern as well as timing of resolution of B lines in these cases was described over the first 24 hours of hospitalization. These findings were compared to the presence or absence of auscultatory crackles and respiratory rate. This study suggests that B lines resolve in a predictable way in dogs with L‐CHF and may still be present after crackles are no longer auscultated.

**Disclosures**

No disclosures to report

**Source of Funding**

ACVIM Pacemaker Grant

## ESVC‐O‐21

### ESVC—European Society of Veterinary Cardiology

#### Use of intravenous nitroglycerin in the treatment of acute left‐sided congestive heart failure in dogs and cats

##### A Climent‐Pastor^1^, Ll Dominguez^1^, I De Blas^2^, AM Girol^1^, V Herrería^1^, P Sebastián‐Marcos^1^, D Casamián‐Sorrosal^1^

###### ^1^Veterinary Teaching Hospital & Department of Animal Medicine and Surgery School, Valencia, Spain; ^2^Faculty of Veteriary Sciences, University of Zaragoza, Zaragoza Spain

Treatment with intravenous nitroglycerin (IV‐NTG), a nitrate venodilator, for acute congestive heart failure (CHF) is reported in humans and anecdotally in dogs, but not in cats. The primary aim was to describe the clinical use of IV‐NTG in dogs and cats (Groups iNTG‐dog;iNTG‐cat) and report adverse events and clinical outcome. The secondary aim was to compare selected variables between iNTGs groups and patients treated with standard therapy prior to the regular use of IV‐NTG in our institution (Group NoiNTG‐dog; NoiNTG‐cat).

Observational retrospective study. Fifty dogs and 32 cats with left‐sided CHF. IV‐NTG was added to standard CHF‐therapy (1 mcg/kg/min gradually increasing up to 6 mcg/kg/min). Group comparison (it‐cat vs. NoiNTG‐cat and iNTG‐dog vs. NoiNTG‐dog) performed for: average daily dose of furosemide (Fur), days of hospitalization, highest creatinine, lowest systolic blood pressure (SBP) and in‐hospital mortality. *P* < 0.05 considered significant.

Group iNTG‐dog (*n* = 29) were 17 males/12 females [age 11 years (0.3–15)]. Group iNTG‐cat (*n* = 14) were 9 males/5 females [age 10.5 years(1–13)]. No differences observed regarding age/sex with the corresponding NoiNTG groups. Most dogs had mitral valve disease (79.3% in iNTG‐dog/66.7% in NoiNTG‐dog) and most cats had hypertrophic cardiomyopathy (50% in iNTG‐cat/77.7% in NoiNTG‐cat), but other congenital/acquired diseases were present.

IV‐NTG was overall well tolerated. Adverse events occurred in 6 cases (13.9%). Phlebitis and necrosis due to extravasation were observed in 3 dogs (10.34% iNTG‐dogs) and 1 cat (7.1% iNTG‐cats). No extravasation occurred in the NoiNTG groups. Persistent hypotension which resolved after halting IV‐NTG occurred in 1 dog and 1 cat. However, the lowest SBP was no different between the iNTG vs. NoiNTG cat and dog groups.

Length of hospitalization and highest creatinine during hospitalization was not different between iNTG vs. NoiNTG groups for both species. The iNTG‐dog group was given a higher Fur during hospitalization (*P* < 0.001) and had a higher in‐hospital mortality (8/29; 27.58%) than NoiNTG‐dogs (0/21; 0%) (*P* = 0.015). No difference was observed for these two variables in cats.

In conclusion, the use of IV‐NTG is reported for first time in a large group of dogs and cats. Treatment with IV‐NTG is overall well tolerated in both species however, close monitoring is warranted as extravasation may occur, leading to tissue necrosis. Hypotension requiring discontinuation of the therapy may be required in a small number of cases. A clinical benefit of IV‐NTG therapy on top of standard left‐sided CHF was not observed in this retrospective study. Further studies (randomized controlled trials) are warranted.

**Disclosures**

No disclosures to report

## ESVC‐O‐22

### ESVC—European Society of Veterinary Cardiology

#### How confident are practicing veterinarians in recognizing, and differentiating pathologic from innocent murmurs in puppies?

##### V. Szatmári, E. Muis, M. van Staveren Utrecht University, Utrecht, Netherlands

###### A recent study showed that the median age of dogs when their congenital heart disease got diagnosed was unnecessary high. In addition, only 10% of these 271 dogs got referred for murmur investigation by the breeders’ veterinarian, before they were sold to a new owner. The present study aimed to evaluate possible causes for these findings by investigating the confidence level of first opinion practicing veterinarians in recognizing cardiac murmurs in puppies and differentiating pathologic from innocent murmurs.

Digital questionnaires were sent out to veterinary practices via newsletters of various national professional organizations. The questionnaire consisted of multiple choice and open questions. Participation was voluntary.

Only the 363 fully completed questionnaires were analyzed of the 524 responders. Though 88% of the respondents reported that recognizing a murmur was easy for them, 68% of the respondents found differentiating pathologic from physiologic murmurs difficult. Detecting a murmur with an intensity of at least 3 out of 6 would prompt 76% of the respondents to recommend immediate referral. Ninety‐five percent of the respondents agreed with the statement that severe congenital heart disease cannot be excluded in puppies without clinical signs. While surgical therapy of a severe congenital heart disease would be recommended even in asymptomatic puppies by 87% of the respondents, only 63% of them thought that surgical therapy in a younger age would carry a better prognosis.

The present study showed that most respondents thought to have sufficient auscultation skills. However, interpreting the auscultation findings might be the reason for late referral of puppies with a loud murmur, as (1) a third of the respondents found it challenging to tell whether a murmur was pathologic, and (2) a quart of the respondents would not refer a pup with a clearly pathologic murmur.

**Disclosures**

No disclosures to report

## ESVC‐O‐23

### ESVC—European Society of Veterinary Cardiology

#### Implantation of a novel transcatheter mitral valve in a pig heart model

##### A Kramer

###### Atlantic Coast Veterinary Specialist, Bohemia, USA

We previously presented data of a transcatheter mitral valve that was implanted in purpose bred dogs. The valve performed well hemodynamically, but there were complications with perforation of the LV apex by the attachment shaft. A new iteration of the valve was developed to address that complication. The aim of this study was to test the device in a pig heart model prior to implantation into purpose‐bred dogs.

Valves were implanted through an atrial transeptal approach in three pig hearts using the Cardiac BioSimulator platform. The hearts were previously purchased from a slaughter house. Imaging and hemodynamic data were collected including echocariographic and intracardiac video imaging, heart rate (HR), left atrial pressure (LAP), aortic pressure (AP), and cardiac output (CO). Data was collected for each valve pre‐implantation and for an hour post‐implantation.

All three valves were successfully implanted. Valves were tested under a variety of hemodynamic conditions. HR ranged from 110 BPM to 180 BPM and CO ranged from 1.4 to 5 L/min. Post‐implantation, in heart 1 the mean LAP and mean AP ranged 11.2–13.4 mm Hg and 69.3–73.7 mm Hg, respectively. Heart 2 ranged from 11.4–13.2 and 64.9–81.9 mm Hg respectively. Heart 3 ranged from 18.9–22.1 mm Hg and 59.3–64.9, respectively. There were no significant differences in mean LAP and mean AP prior to implantation and post‐implantation.

These data support moving to the next stage of testing in purpose bred dogs.

**Disclosures**

George Kramer is inventor of the valve used in the study and the major shareholder in Ultravet Medical Devices which is developing the valve and funded the study.

**Source of Funding**

Ultravet Medical Devices

## ESVC‐O‐24

### ESVC—European Society of Veterinary Cardiology

#### End‐diastolic forward flow and restrictive physiology in dogs with pulmonary stenosis

##### E Boz^1^, C Pergamo^2^, M Papa^2^, D Pradelli^2^, C Rossi^2^, S Signorelli^2^, C Bussadori^2^

###### ^1^Clinica veterinaria Gran Sasso, Milano, Italy; ^2^Clinica veterinaria Gran Sasso, Milano, Italy

The study of the function of the right ventricle (RV) is a topic of great interest in the scientific community and some studies have evaluated parameters of the right ventricular systolic function and have correlated them to various RV pathologies and possible clinical findings. Less information was obtained on right ventricular diastolic function. In veterinary medicine the characteristics of the restrictive RV and the presence of end‐diastolic forward flow (EDFF) have not yet been described. This type of flow is an antegrade flow that is observed with the Doppler study of the pulmonary artery and occurs at the end of the diastolic phase. The EDFF coincided with premature pulmonary valve opening. Studies carried out in human medicine with catheterization of the RV demonstrated that this flow occurred when RV end‐diastolic pressure equalled or exceeded pulmonary arterial diastolic pressure. In human medicine the study of RV restrictive filling pattern has been reported as a sign of postoperative outcome in repaired tetralogy of Fallot (TOF) but it has also been considered in some studies on patients with pulmonary valve pathologies such as stenosis or atresia. Pulmonary valve stenosis (PVS) is one of the most common congenital heart diseases in dogs. The echocardiographic parameter that is taken as a reference to evaluate the severity of the disease is the peak gradient of the pulmonary antegrade flow. In patients with a pulmonary valve peak gradient greater than 60 mmHg pulmonary valvuloplasty is recommended. For our retrospective study we consider a total of 200 dogs with PVS divided into 132 dogs with type A and 68 type B, seen at a reference veterinary clinic from 2020 to 2023. All these dogs underwent echocardiographic examination to observe the morphological characteristics of the PVS and consider the presence or absence of restrictive RV with EDFF. In the group of type A PVS 34 patients were classified as having mild PVS, 28 moderate PVS, and 70 severe PVS. In the group of type B PVS 7 were mild, 12 moderate and 49 severe. Independent‐sample t‐test analysis revealed that the severity of PVS is greater in dogs with EDFF, both in the group of patients with type A stenosis and in patients with type B stenosis. The study suggest that RV restrictive physiology is common in dogs with severe PVS.

**Disclosures**

No disclosures to report

## ESVCN‐O‐1

### ESVCN—European Society of Veterinary & Comparative Nutrition

#### Short‐term oral intake of inorganic phosphate additives causes an increase of FGF23 in cats and dogs

##### B Dobenecker, S Hering, C. Steffen

###### Ludwig‐Maximilians‐Universität, München Oberschleissheim, Germany

For technical reasons, for example, as texturiser, urine acidifier (prevention of uroliths), palatability enhancer, or preservative, pet food may contain considerable amounts of soluble phosphates from inorganic sources (Pi) such as phosphoric acid. In dogs it was already demonstrated that several Pi sources can disrupt the phosphate homeostasis, for example, by increasing serum FGF‐23 concentrations. FGF‐23 plays a crucial regulatory role and is acknowledged as early marker of renal disease. The aim of this study was to investigate the effect of dietary Pi intake from phosphoric acid (H_3_PO_4_) combined with sodium phosphate (NaH_2_PO_4_) on serum concentrations of FGF‐23 in healthy adult cats and dogs.

Healthy adult European short hair cats (*n* = 11) and Beagles (*n* = 8) were fed a balanced control (CON) diet for either 28 (cats) or 18 (dogs) days followed by feeding an above‐maintenance level of phosphorus (HP) by adding H_3_PO_4_ and NaH_2_PO_4_.The Ca/P ratio was adjusted by using calcium carbonate. On the last day of the trial, blood samples were obtained in the fasted animals (pre) and 3 h postprandially (ppr) and analysed for serum FGF‐23 (sandwich ELISA by KAINOS Laboratories Inc., Tokyo, Japan), serum calcium and serum phosphate (photometric, modified vanadate molybdate method). For statistical evaluation, Student's t‐test and Shapiro‐Wilk test were used.

HP feeding (H_3_PO_4_+NaH_2_PO_4_) led to a lower serum phosphate concentration in the fasted animals and a significantly increased concentration above the reference range 3 h postprandially. The ppr serum CaxP product increased above the recommended limit in the HP groups. FGF‐23 was significantly increased both pre‐ and postprandially due to the dietary intake of phosphoric acid and sodium phosphate.

The results support previous findings that Pi significantly affects serum phosphate. As in patients with chronic kidney disease, FGF‐23 levels were found to be positively correlated with increasing serum phosphate levels. Increasing levels of FGF‐23, serum phosphate, and serum CaxP product are important parameters to evaluate stage and progression of kidney disease. The significant impact even of short‐term supply with dietary Pi on serum phosphate and FGF‐23 in healthy cats and dogs raises severe concerns regarding the safety of phosphate additives.

**Disclosures**

No disclosures to report

## ESVCN‐O‐2

### ESVCN—European society of Veterinary & Comparative Nutrition

#### Beneficial response to a very low‐carbohydrate home‐cooked diet in dogs with refractory chronic enteropathy: pilot study of 25 cases

##### PL Blanchard^1^, P Lecoindre^2^, A Corsaletti^2^, F Bedel^2^, M Maxime^1^, J Dumas^1^, A Lecoindre^2^

###### ^1^Animal Nutrition Expertise sarl, Arbonne, France; ^2^OnlyVet, Saint‐Priest, France

Chronic enteropathies in dogs are quite common. Although the cause can vary, there is an overlap of clinical signs and chronic inflammation of the intestinal mucosa. When reasoned medical management (prescription diets, antibiotic therapy (AB), immunosuppressive therapy (IST)) does not stabilize the symptoms, these enteropathies will be qualified as refractory chronic enteropathies (RCE). Such dogs may remain with various medical treatment for years. Low fat or novel protein home‐cooked diet has been proposed. But to our knowledge, never has been a very low‐carbohydrate home‐cooked diet (VLCHD), although dogs can digest and tolerate starch with various genetic and epigenetic capacities. We formulate the hypothesis that a lack of tolerance to starch activates various mechanisms and generates a chronic inflammatory state, and that a VLCHD may improve clinical signs of RCT.

Inclusion criteria: dogs coming to the specialist consultation, and diagnosed with RCE for at least 3 months. They are prescribed, for at least 2 months, a VLCHD, formulated to cover all nutrient requirements. The VLCHD included meat, raw canola oil, vegetables, Vit'i5^TM^ as a mineral‐vitamin supplement. Medical treatments were supposed to be stopped within one month. After 2 months, the diet was changed, with introduction of up to 20% of energy as starch (20CHD). Dogs were followed for 4 months. Wilcoxon test were used for CCECAI and body condition scores.

A total of 25 dogs were included and followed during 3 (*n* = 4) to 4 (*n* = 21) months. The decrease and normalization of the CCEICA score was significant (*P* < 0.01) within 2 weeks, and stayed low thereafter with at T0 of 6.72 ± 2.0 [2–11], T2wks of 2.64 ± 2.5 [0–9], T4m of 1.05 ± 1.8 [0–6] CCECAI mean ± SD [min–max].

Clinical signs and body condition score (*P* < 0.05) improved significantly with time. After 2 months of diet, most owners (15/25) refused the reintroduction of starch. In the 10 dogs switched to 20CHD, the CCEICA score increased again in 3/10 dogs (starch was stopped again in 2/10 and CCECAI got back to 0). Medical treatments were still maintained in the last month of follow up in 9/25 dogs (5 with AB, 2 with IST, 2 with both), sometimes for unclear reasons.

This pilot study suggests that a very low‐carbohydrate balanced home cooked diet could appear as a clinically beneficial option for dogs with refractory chronic enteropathy. Further studies are needed to understand the underlying mechanism, especially the effect on the microbiota of such a diet.

**Disclosures**

Dr Blanchard formulated the mineral‐vitamin supplement used to balance the home‐cooked diet proposed in the study

## ESVCN‐O‐3

### ESVCN—European society of Veterinary & Comparative Nutrition

#### Follow‐up in 167 homemade diets for maintenance and pathologic conditions in dog

##### G Pignataro, PE Crisi, LE Landolfi, B Belà, FI Fusaro, A Gramenzi

###### University of Teramo, Teramo, Italy

Homemade diets for dogs have gained popularity in recent times because of their high palatability and are considered more natural and healthier, but most important as a veterinarian's tool is total customization, as modifications can be made not only according to actual caloric needs, but especially in case of one or more diseases. Few publications in the literature demonstrate the benefits of homemade diets formulated and balanced by a veterinary nutritionist.

The aim of this study was to conduct a follow‐up of home diet to evaluate the response to the diet especially in dogs with diet‐related diseases.

167 dogs underwent nutritional examination and formulation of a personalized home diet, 69% of the dogs examined were purebred. Referrals for consulting were in 48 cases for chronic enteropathy, 47 for maintenance diet, 23 for dermopathy, in 29 for both gastrointestinal and dermatological problems, and in 9 for weight loss; the remaining cases were other diseases.

When follow‐up was conducted over a median of 14 months (minimum 5 to Maximum 24), 104 maintained the homemade diet, while 63 returned to the industrial diet after three months. The reasons for returning to the industrial diet were owner unavailability (27), adverse reactions (15), missed palatability for the dog (8), appearance of new diseases (5) and death of the animal (8).

Among the 30 dogs with maintenance purpose in 70% the coat improved, in 46% the frequency of stool decreased; stool consistency, appetite and energy remained unaltered.

Diet was required for 68 cases with mainly gastrointestinal and/or dermatological conditions as therapeutic support or treatment; 64 of these cases improved clinically with the satisfaction of the owner, leaving us to assume that these cases were related to adverse reactions to food (AFR).

Concerning the nine dogs with a weight loss purpose, six were able to achieve a correct Body Condition Score (BCS).

The clinical results obtained in this study are promising. The absence of endogenous allergens (e.g., mites), the customization of the homemade diet by the nutritionist for the selection of functional ingredients and supplementation, and especially the percentages of nutrients, significantly improve the overall health and living conditions of the dog, offering a new clinical nutritional tool to be considered in veterinary practice.

**Disclosures**

No disclosures to report

## ESVCN‐O‐4

### ESVCN—European society of Veterinary & Comparative Nutrition

#### Effects of Lactobacillus reuteri NBF 2 DSM 32264 supplementation on healthy Certosino cat performance

##### B Bela', G Pignataro, I Fusaro, PE Crisi, A Gramenzi

###### Faculty of Veterinary Medicine, University of Teramo, Teramo, Italy

The probiotic contribution on human and pet health is already known, however few studies evaluated the probiotic effect on specific breeds of dogs or cats.

The present study analyzed the effectiveness of a specific probiotic strain, *L. reuteri* NBF 2 DSM 32264, on the intestinal health of healthy Certosino adult cats by analyzing their bodyweight (BW), body condition score (BCS), fecal score (FS) and fecal moisture (FM). In addition, microbiological analyses were carried out to quantify bacterial species such as *E. coli* (for the total coliform count) and Lactobacilli. The research was conducted according to the directive 2010/63/EU and did not imply any form of animal suffering or health risk, since it focused on the administration of a natural substance. Healthy adult male and female non pregnant (age > 1 year) cats (*n* = 16, Certosino; 10 females, 6 males: 10 + 6 = 16) were selected for the study and were randomly assigned to the control group (*n* = 8; male: female = 1:1) and to the treated group (*n* = 8; male: female = 1:3). The control group fed the standard diet with the addition of a placebo while the treated group received the probiotic. The study lasted 35 days (+ 14 d acclimatation) in line with the time needed to assess any probiotic effects. BW data showed no differences between the two groups of cats. However, the fecal moisture was significantly lower at the end of the trial in the treated group and the beneficial effect of *Lactobacillus reuteri* DSM 32264 administration was also confirmed by the FS values recorded among the two groups of cats. Additionally, at the end of the study period, there was a significant increase of Lactobacilli in the treated group compared to the control group (*P* < 0.0001). The data collected in this study reported the ability of the probiotic strain *L. reuteri* DSM 32264 to improve fecal quality parameters such as fecal moisture and fecal quality in healthy Certosino adult cats showing an increase in Lactobacilli count and a little reduction of total coliforms.

**Disclosures**

No disclosures to report

## ESVE‐O‐1

### ESVE—European Society of Veterinary Endocrinology

#### Induction of hepatic insulin production using AAV gene therapy in naturally‐occurring canine diabetes mellitus; a potential future treatment?

##### D Foale

###### University of Nottingham, Loughborough, UK

Gene therapy is an effective treatment for human haemophilia B with a single intravenous injection of the vector curing patients at least 6 years. Gene therapy has also been reported to be efficacious in induced diabetic dogs, but an efficacious gene therapy treatment for naturally‐occurring canine diabetes mellitus has not been reported.

Our previous work showed lentiviral transduction of primary canine mesenchymal stromal cells led to short and long‐term insulin secretion. Following the successful adeno‐associated viral serotype 8 (AAV8) treatment of human haemophilia B, we utilized intravenous gene therapy to treat mice with both induced diabetes and naturally‐occurring diabetes and restored euglycaemia for more than one year.

We have now used a similar protocol to treat naturally‐occurring canine diabetes in three patients who received progressively increasing single intravenous doses of a single stranded serotype‐8 pseudotyped adeno‐associated virus (ssAAV2/8) vector encoding the codon‐optimised human proinsulin gene under a constitutive liver specific promoter. The first was an 8‐year old neutered male dog with naturally occurring diabetes and no co‐morbidities. No significant adverse effects of the treatment were noted and the dog remained well for over four years. Human c‐peptide was measurable in serum within 5 days of transfection at concentrations of up to 150 pmol/l, which rose to 280 pmol/l by 3 months post‐transfection, thereby proving successful transduction and sustained insulin production. Although the dog still required exogenous insulin to maintain glycaemic stability, his daily insulin dose requirement was reduced by 25%. No immunological response was seen to the transgene product but a marked virus‐neutralizing antibody response was seen against the vector, thereby negating the possibility of repeating the treatment with this vector serotype. The second dog (8‐year old neutered female) underwent the same regime but received a higher vector dose and also remained well for four years with no significant adverse effects and her exogenous insulin dose was reduced by 35%. The third dog (9‐year old neutered male) received the highest vector dose and became euglycaemic without exogenous insulin for nine weeks post‐transfection, but then experienced significant hypoglycaemia at home and was euthanased.

This is the first report of the use of an AAV‐8 gene therapy treatment in naturally occurring diabetic dogs and shows successful, efficacious transfection with evidence of sustained endogenous hepatic insulin production but establishing the dose of vector required to achieve sustained and safe euglycaemia in dogs using this technique is yet to be concluded.

**Disclosures**

(1) This work was in part supported by The Lollipop Foundation, a foundation established by Professor Calne to support translational medical science research. However, no financial gain has, or will, arise for The Lollipop Foundation or any of the authors as a result of this research (2) Professor Foale was a share‐holding partner at the veterinary centre where the research was performed. However, the centre performed all of the research pro bono and absorbed all of the associated clinical costs, so has not and will not receive any financial gain as a result of this research. (3) The Intellectual Property right for this research are held by Foale White Associates, a limited company established for the purpose of holding the IP, but not financial gain or benefit has been made by the company or its owners as a result of this work

**Source of Funding**

The Lollipop Foundation Dick White Referrals University of Cambridge National University of Singapore

## ESVE‐O‐2

### ESVE—European Society of Veterinary Endocrinology

#### Effect of two diets on glycemic variability and glycemic control assessed by flash glucose monitoring system in diabetic dogs

##### AM Tardo^1^, CG Vecchiato^1^, E Gherlinzoni^1^, A Corsini^2^, S Corradini^3^, F Del Baldo^1^, E Pagani^4^, F Fracassi^1^

###### ^1^University of Bologna, Ozzano dell'Emilia, Italy; ^2^University of Parma, Parma, Italy; ^3^Clinica Veterinaria dell’Orologio Sasso Marconi, Italy; ^4^Monge & C S.p.a. Monasterolo di Savigliano, Italy

The Freestyle Libre flash glucose monitoring system (FGMS) has revolutionized the management of dogs with diabetes mellitus. Thanks to this device, various metrics assessing glycemic variability (GV), which refers to glycemic excursions throughout the day or on different days, are affordable. In human medicine, GV is associated with short‐ and long‐term diabetic complications and is emerging as an additional glycemic target. This study aimed to assess GV and FGMS‐derived metrics in diabetic dogs receiving a commercial diet (CD) and a home‐cooked diet (HCD) in a randomized crossover trial.

Ten diabetic dogs on insulin treatment (6 insulin glargine 300 U/ml, 4 porcine lente insulin) with good glycemic control were prospectively enrolled. Dogs were randomly assigned to receive either a high‐fiber (total dietary fiber on a dry matter basis [TDF]: 18%) moderate‐carbohydrate CD or a moderate‐fiber (TDF: 9%) and carbohydrate HCD in a 2×6‐week period (CD‐HCD or HCD‐CD). Dogs were re‐evaluated every 2 weeks (T2‐T4‐T6 for each diet) and interstitial glucose (IG) was continuously monitored using the FGMS. At each time point, FGMS‐derived metrics including the percentage of time‐in‐range (TIR%, 70–250 mg/dL), time above range (TAR%, >250 mg/dL), time below range (TBR%, <70 mg/dL) and mean glucose (MG) were recorded. IG concentrations were analyzed for post‐prandial hyperglycemia (PPH; 30, 60, 90, and 120 min after meal) and glucose nadir. The following GV metrics were evaluated: standard deviation of mean glucose concentration (SD), within‐day percent coefficient of variation (%CV), between‐day %CV, and mean amplitude of glycemic excursion (MAGE). Differences between diets were analyzed by a repeated measure ANOVA fitting a crossover design with pairwise comparisons.

A total of 59219 IG concentrations were recorded. The TAR% was significantly lower (*P* = 0.04) and TBR% higher (*P* = 0.03) during the HCD period; however, the TIR% was not different between the two diets (*P* = 0.10). The PPH 30 min after meal (PPH‐30) tended to be lower (*P* = 0.056) in dogs receiving HCD, while no differences were found for the other time points. Glucose nadir and MG did not differ between HCD and CD. Regarding GV metrics, no differences were found between the two diets.

In conclusion, both CD and HCD can be considered valid dietary options in diabetic dogs, with no significant changes in terms of TIR% and GV metrics. The HCD decreased the TAR% and the PPH‐30 and increased the TBR%, suggesting a better glucose‐lowering effect of this dietary formulation. However, the consistency and reproducibility of HCD rely mainly upon the owner's effort.

**Disclosures**

Antonio Maria Tardo Financial support: Ph.D. scholarship funded by Monge. Speaking & consultancies: Dechra, MYLAV Veterinary Laboratory. Carla Giuditta Vecchiato Speaking & consultancies: Monge, Prolife‐Zodiaco. Andrea Corsini Speaking & consultancies: MYLAV Veterinary Laboratory, Boehringer Ingelheim Sara Corradini Speaking & consultancies: Idexx Laboratories Italy Francesca Del Baldo Speaking & consultancies: MYLAV Veterinary Laboratory, Boheringer Ingelheim Elena Pagani Is employed at Monge & C. S.p.a. Federico Fracassi Financial support: Dechra, MSD, Monge. Speaking & consultancies: Boehringer Ingelheim, Dechra, MSD, Royal Canin, Hill's, Nestlé Purina, MYLAV Veterinary Laboratory.

## ESVE‐O‐3

### ESVE—European Society of Veterinary Endocrinology

#### Efficacy and safety of once daily oral sodium‐glucose co‐transporter‐2‐inhibitor velagliflozin compared to twice daily insulin injection therapy in diabetic cats

##### SJM Niessen^1^, HS Kooistra^2^, Y Forcada^1^, CR Bjørnvad^3^, B Albrecht^4^, F Rössner^4^, E Herberich^4^, C Kroh^4^

###### ^1^Royal Veterinary College, London, UK; ^2^Utrecht University, Utrecht, Netherlands; ^3^Bjørnvad Holding, Copenhagen, Denmark; ^4^Boehringer Ingelheim Ingelheim, Germany

Treatment options for feline diabetes mellitus are limited and usually involve insulin injections and careful monitoring to avoid hypoglycaemia. Sodium‐glucose‐co‐transporter‐2‐inhibitors (SGLT‐2‐inhibitors) form a new category of anti‐diabetes drugs.

It was hypothesised that treatment with oral once daily SGLT‐2‐inhibitor velagliflozin is an alternative to insulin injections without inferior clinical, glycaemic, safety and quality of life (QoL) effects.

A prospective field study was conducted, involving 127 client‐owned diabetic cats (127/127 safety assessment; 116/127 efficacy assessment), randomized to 1 mg/kg oral SID velagliflozin liquid or titrated BID caninsulin injections. The primary efficacy outcome involved assessment for non‐inferiority (margin Δ: 15%) of velagliflozin compared to caninsulin on day 45; secondary endpoints included glycaemic and clinical assessments during 91 days.

On day 45, 29/54 (53.7%) of velagliflozin‐treated cats and 26/62 (41.9%) of caninsulin‐treated cats showed treatment success, proving non‐inferiority (non‐inferiority margin –11.8; upper one‐sided 97.5%‐confidence interval‐∞, 6.34). By day 91, QoL, and polyuria/polydipsia had improved in most cats; on blood glucose (BG) curves, mean BG was <14 mmol/L in 43/54 (79.6%) (velagliflozin), 38/62 (61.3%) (caninsulin); minimum BG <9 mmol/L in 42/54 (77.8%) (velagliflozin) and 42/62 (67.7%) (caninsulin); serum fructosamine <450 μmol/L in 41/54 (75.9%) (velagliflozin) and 38/62 (61.3%) (caninsulin).

Most frequent adverse events for velagliflozin were: loose stools/diarrhoea (*n* = 23, 37.7%), positive urine culture (*n* = 19, 31.1%), and non‐clinical hypoglycaemia (*n* = 8, 13.1%); for caninsulin: clinical and non‐clinical hypoglycaemia (*n* = 35, 53.0%), positive urine culture (*n* = 21, 31.8%) and loose stools/diarrhoea (*n* = 10, 15.2%). In four velagliflozin‐cases, and in none of the caninsulin‐cases, suspected diabetic ketoacidosis (DKA) was diagnosed (cats appeared ill and showed ketonuria; three were naïve cases; one was insulin pre‐treated); all presented with euglycaemia and three of four events occurred within the first week after velagliflozin start. In three of four cases DKA‐treatment was allowed by the owner which proved successful in all.

In conclusion, diabetes mellitus was successfully treated with once daily oral velagliflozin in most cats and was associated with marked sustained decreases in glycaemic parameters, no need to titrate dose, no clinical hypoglycaemia and improved QoL. Main side effect of note was loose stools. DKA proved rare and seemed to mostly occur soon after treatment start, implying initial regular screening for ketonuria or ketonaemia is advisable.

**Disclosures**

All authors have relationships (ad hoc as consultant or employed) with Boehringer Ingelheim, which is the company looking to market the investigated drug.

**Source of Funding**

Boehringer Ingelheim

## ESVE‐O‐4

### ESVE—European Society of Veterinary Endocrinology

#### Increased insulin‐like growth factor 1 concentrations in a population of non‐diabetic cats with overweight/obesity

##### DD Miceli^1,2^, A Jaliquias^3^, J García^3^, C Vecino^3^, F Gallelli^3^, OP Pignataro^2^

###### ; ^1^Veterinary Science Center, Maimonides University, Buenos Aires, Argentina; ^2^Institute of Experimental Biology and Medicine IBYME–CONICET, Buenos Aires, Argentina; ^3^Private practice, Buenos Aires, Argentina

Feline hypersomatotropism (HST) has been typically associated with diabetes mellitus (DM), whereas HST without concurrent DM had only been reported in a few case reports. Studies evaluating the prevalence of feline HST in the non‐diabetic population are lacking. Weight gain may be observed in diabetic cats with HST. The aim of the study was to evaluate circulating insulin‐like growth factor‐1 (IGF‐1) in non‐diabetic cats with overweight/obesity and to screen this population for the presence of HST. In this prospective study, fifty‐six cats with overweight/obesity from referral centers between March 2021 and March 2023 were evaluated. Serum IGF‐1 was measured as part of the routine tests for overweight/obesity. Non‐diabetic cats were included in the study if they had a body condition score (BCS) >6/9. Fifty‐four cats were Domestic Short‐Hair, 1 Domestic Long‐Hair and 1 Siamese. Thirty‐seven cats were neutered male and 19 cats were neutered female; median age was 8 years (range 2–15 years); median body weight was 7.6 kg (range 5.3–11 kg); 20 cats were classified as overweight (BCS 7/9), while 36 cats were classified as obese (BCS 8–9/9). Median serum IGF‐1 concentrations were 589 ng/ml (range 123–2450 ng/ml). Median serum IGF‐1 concentrations of cats with BCS 7/9, 8/9 and 9/9 were 562 ng/ml (range 123–1456 ng/ml), 566 ng/ml (range 151–1210 ng/ml), 608 ng/ml (range 284–2450 ng/ml), respectively. There was a positive linear correlation between serum IGF‐1 concentrations and body weight (*r* = 0.36, 95% CI 0.1–0.57 *P* = 0.006). Four out of 56 cats had IGF‐1 concentrations >1000 ng/ml, resulting in a 7.1% (95% confidence interval 1.9%–17.2%) HST prevalence rate in non‐diabetic cats with overweight/obesity. Eight cats (14.3%) had IGF‐1 concentrations between 800 and 1000 ng/ml. Intracranial imaging was performed in the four cats with IGF‐1 concentrations >1000 ng/ml and pituitary enlargement was detected in 1/4 cases on computed tomography. All of these cats had phenotypic changes consistent with acromegaly: prognathia inferior (4/4), abdominal enlargement (3/4), weight gain (3/4), broad facial features (2/4), polyphagia (2/4), broadening of paws (2/4), respiratory stridor (1/4) and degenerative arthropathy (1/4). A proportion of 7.1% of overweight/obese non‐diabetic cats from referral centers had serum IGF‐1 concentration and clinical signs compatible with HST/acromegaly. Likewise, serum IGF‐1 concentrations had a positive correlation with body weight in this population of cats. This study highlights that HST should not be suspected only in diabetic cats and emphasizes the relevance of screening different populations of non‐diabetic cats to increase the recognition of HST.

**Disclosures**

No disclosures to report

## ESVE‐O‐5

### ESVE—European Society of Veterinary Endocrinology

#### A genome‐wide association study investigating the genetic basis of hyperthyroidism in domestic cats

##### PA Norman^1^, RE Jepson^1^, HM Syme^1^, MD Wallace^2^, LJ Davison^1^

###### ^1^Royal Veterinary College, London, UK; ^2^Wellcome Trust Centre for Human Genetics, University of Oxford, Oxford, UK

Hyperthyroidism (HTH) is the most common feline endocrine disease, affecting up to 10% of cats >10 years old. Despite its high prevalence, HTH pathogenesis is not well characterised. However, it appears to be multi‐factorial with environmental, dietary and genetic risk factors.

This study aimed to identify genetic regions associated with HTH risk, utilising data from a genome‐wide association study (GWAS) previously investigating azotaemia and hypertension. This was feasible because >10% of participating cats developed HTH during long‐term follow‐up.

Genotype data from the Illumina 63K DNA feline array were analysed for 872 non‐purebred cats >8 years old, of which 102 developed HTH (total thyroxine [TT_4_] >55 nmol/L or institution of treatment). This GWAS investigated HTH as a dichotomous case‐control phenotype and also quantitatively by including age of onset, rate of progression and maximum TT_4_ value. Longitudinal data was used to confirm euthyroidism in controls (median [25th, 75th %] follow‐up 217 [0, 728] days). To prevent control misclassification, a tiered approach was developed for defining euthyroidism based on last age at follow‐up, and TT_4_ and TSH measurements. Euthyroid control groups were: most inclusive (*n* = 770), moderately inclusive (*n* = 527) and most strictly defined (*n* = 423).

Overall, 71 candidate variants nominally associated with feline HTH (EMP1 ≥ 1.00e^−5^ to EMP1 ≤ 0.0011). Notably, some variants are located in gene regions involved in signaling pathways for thyroid hormone synthesis, and others are linked to human thyroid carcinoma.

This study revealed novel genetic associations with feline HTH. Fine‐mapping of regions of interest and genotyping in additional cats is now being undertaken for validation.

**Disclosures**

Dr. Marsha Wallace is supported by a Clinical Research Fellowship at the Royal Veterinary College. Prof Harriet Syme: This work was not directly funded. Funding from the Evetts‐Luff foundation to support a feline PhD student (Paul Norman) Although they have never directly funded the research we do in hyperthyroidism support for the feline research clinic comes mainly from Royal Canin (Waltham), with additional funding from Idexx, and support for other PhD students from multiple drug companies including Boehringer Ingelheim and Ceva (of note both of these companies market treatments for hypertension) as well as charities such as PetPlan Charitable Trust, PetSavers. In the past year I have been a paid speaker for ESVE, ECVIM, BSAVA modular certificate, ACVIM, Hellenic Veterinary Medical Feline conference, and Renal week as well as receiving funding to support travel for the IRIS board meeting (from Zoetis). Prof Lucy Davison: Indirect funding supporting current research of LJD (data not presented here): Medical Research Council (Clinician Scientist Fellowship), American Kennel Club Canine Health Foundation, Morris Animal Foundation, UK International Coronavirus Network, Royal Canin, BSAVA PetSavers, UK Research and Innovation, Hong Kong Jockey Club, PetPlan Charitable Trust, Dechra Veterinary Products. Speaker honoraria in past 12 months: ACVIM, Improve International, ESVE Summer School. Prof Rosanne Jepson: Any form of support (financial or otherwise) for the study described in the abstract. Petplan Charitable Trust, Foundation for Feline Renal Research—funded the original GWAS Any form of support for other work that the authors are involved in: PetPlan Charitable Trust, Boehringer Ingelheim Financial relationships (which may be unrelated to the subject matter of the abstract) whereby the individual or a relative benefits by receiving a salary, royalties, consulting fees, speaker honoraria, ownership interests (e.g., stock or stock options), or other benefits: Ceva, Boehringer Ingelheim, Idexx, Hills

**Source of Funding**

Funding from the Evetts‐Luff foundation for PhD studies of Dr Paul Norman.

## ESVE‐O‐6

### ESVE—European Society of Veterinary Endocrinology

#### Effect of duration of hyperthyroidism and degree of thyroid pathology on recovery of pituitary‐thyroid axis and creatinine concentration in radioiodine‐treated cats followed up over a 1‐year period

##### AP Menzel, A Güssow, J Lin, V Patzelt, N Bauer, K Hazuchova

###### Small Animal Clinic, Internal Medicine, Justus Liebig University Gießen, Giessen, Germany

Radioiodine therapy (RAIT) is the gold standard for treatment of hyperthyroidism in cats, but data on recovery of the pituitary‐thyroid axis following RAIT is scarce. Our aim was to evaluate the effect of duration of hyperthyroidism and degree of thyroid pathology on changes in thyroxine (T4), thyrotropin (TSH) and creatinine concentration over a 1‐year period following RAIT.

Hyperthyroid cats presented for RAIT between April 2021 and April 2022 were prospectively enrolled. T4, TSH and creatinine were determined before, 1 week, and 1, 3, 6 and 12 months following RAIT. The effect of re‐examination timepoint following RAIT, duration of hyperthyroidism prior to RAIT (< 6, 6–12, 12–24, >24 months), and degree of thyroid pathology based on technetium‐99m scintigraphy (uni‐/bilateral adenoma, carcinoma) on log(T4+1), TSH and creatinine was investigated by mixed‐effects modeling. Cats with persistent hyperthyroidism were excluded from the analysis. Test results of cats with RAIT‐induced hypothyroidism from re‐examination timepoints after starting levothyroxine (whenever low to low normal T4, TSH above upper reference range, and newly‐diagnosed azotaemia [creatinine >140 μmol/L] occurred) were excluded from the analysis of T4/TSH. Significance was set at *P* < 0.05.

Fifty‐seven RAI‐treated cats, with disease duration of <6 (*n* = 16), 6–12 (*n* = 13), 12–24 (*n* = 12), >24 (*n* = 9) months, or unknown (*n* = 7) were enrolled. Unilateral adenoma, bilateral adenoma, and carcinoma was diagnosed in 20/57, 31/57, and 6/57, respectively. Six cats with persistent hyperthyroidism (4 with uni‐/ bilateral adenoma, 2 with carcinoma) and one cat with missing values that only became euthyroid after 8 months were excluded. Eight cats were started on levothyroxine at 1 month, 7/57 at 3 months, 4/57 at 6 months and 1/57 at 12 months post RAIT. Neither disease duration nor the degree of thyroid pathology had an effect on T4, TSH and creatinine post RAIT. When compared to enrolment, T4 decreased at week‐1 (*P* < 0.001) and further decreased at month‐1 (*P* < 0.001); TSH increased at month‐1 (*P* = 0.001), and creatinine was higher at month‐1 (*P* < 0.001); but none of the parameters significantly changed afterwards.

Treatment success can be generally evaluated at 1 month post RAIT. As indicated by increase in TSH concentration, thyroid‐pituitary axis needs a minimum of 1 month post RAIT to recover from hyperthyroidism‐induced suppression, but hypothyroidism necessitating levothyroxine supplementation might not be diagnosed before 6 or rarely even 12 months post RAIT. Although creatinine did not increase significantly after 1 month post RAIT in this cohort, individual cats developed azotaemia as late as 6 months post treatment.

**Disclosures**

Katarina Hazuchova has consultancy agreement with Boheringer Ingelheim.

## ESVE‐O‐7

### ESVE—European Society of Veterinary Endocrinology

#### Survival times of radioiodine treated hyperthyroid cats with and without iatrogenic hypothyroidism and investigation of effect of levothyroxine supplementation on survival time

##### TL Williams^1^, T Hall^2^, J Wakeling^3^

###### ^1^University of Cambridge, Cambridge, UK; ^2^North West Nuclear Medicine, British Columbia, Canada; ^3^Douglas College, British Columbia, Canada

Hyperthyroid cats that are hypothyroid and azotaemic after medical and/or surgical treatment have a poorer prognosis than hypothyroid non‐azotaemic cats, and levothyroxine (LT4) supplementation improves survival times of I131 treated hypothyroid azotaemic cats. Here, we report the survival times of I131 treated hyperthyroid cats with and without iatrogenic hypothyroidism and/or azotaemia, and investigate the association between LT4 supplementation and survival time in hypothyroid cats.

I131 treated cats were diagnosed as euthyroid or hypothyroid using the TSH stimulation test. Cats were classified as azotaemic at time of TSH stimulation testing if serum creatinine concentrations were >221 μmol/L with urine specific gravity (USG) <1.035. LT4 therapy was offered to owners of hypothyroid cats regardless of azotaemic status, and decision to treat was based on client preference. LT4 dose was increased until TSH normalized (where possible). Survival time was calculated from time of TSH stimulation test to time of death (all‐cause mortality). The log rank test was used to compare survival times between groups and data are presented as median [range].

I131 treatment was 423 [86–1281] days prior to TSH stimulation testing. 25/117 cats (21%) were classified as hypothyroid, and 13 of these (4 of which were azotaemic) were given LT4 supplementation whereas 12 (6 of which were azotaemic) did not receive LT4. Post‐LT4 supplementation serum TSH and total thyroxine concentrations were 0.29 [<0.03–11.2] ng/mL (*n* = 10) and 23.5 [9.2–43.3] nmol/L (*n* = 12) respectively. Euthyroid non‐azotaemic cats had longer survival times than euthyroid azotaemic cats (1140 [185–2484], *n* = 78 vs. 471 [64–1289] days, *n* = 14; *P* < 0.001) and non‐supplemented hypothyroid cats (595 [34–1852] days; *P* = 0.004). Euthyroid azotaemic cats had shorter survival times than supplemented hypothyroid cats (969 [300–2401] days; *P* = 0.013). There was no statistically significant difference in the survival times of LT4 supplemented hypothyroid cats and non‐supplemented hypothyroid cats (*P* = 0.115), however in the non‐azotaemic hypothyroid group alone, LT4 supplementation improved survival times (LT4 supplemented: 1037 [300–2401] days, *n* = 9 vs. non‐supplemented: 768 [34–1014] days, *n* = 6; *P* = 0.027).

Non supplemented hypothyroid cats had shorter survival than euthyroid non‐azotaemic cats, although this may partly reflect an increased prevalence and/or severity of concurrent chronic kidney disease in the non‐supplemented hypothyroid group. Although survival time was not significantly increased by LT4 supplementation in I131 treated cats with iatrogenic hypothyroidism as a whole, LT4 supplementation did increase survival times in the non‐azotaemic hypothyroid cats. Randomized trials investigating the effect of LT4 supplementation on survival of azotaemic and non‐azotaemic hypothyroid cats are warranted.

**Disclosures**

No disclosures to report

**Source of Funding**

NECK (Natural Sciences and Engineering Research Council of Canada) Applied Research and Development Grant (part of the College and Community Innovation Program). Additional financial support was provided by Douglas College Institutional Research office through the Research and Scholarly Activity Fund. CONFLICT OF INTEREST DECLARATION In‐kind support of this study was provided by IDEXX Laboratories in the form of no‐charge laboratory testing: all blood and urine samples collected during the course of the study were analyzed at IDEXX laboratories at no charge. IDEXX Laboratories staff were not involved in the study design, data collection, interpretation of data, or the writing of the report (other than laboratory methods section). OFF‐LABEL ANTIMICROBIAL DECLARATION Authors declare no off‐label use of antimicrobials. INSTITUTIONAL ANIMAL CARE AND USE COMMITTEE (IACUC) OR OTHER APPROVAL DECLARATION This work involved the use of non‐experimental (owned) animals only. Ethical approval for the study was granted by the Douglas College Institutional Animal Care Committee (accredited by the Canadian Council on Animal Care).

## ESVE‐O‐8

### ESVE—European Society of Veterinary Endocrinology

#### Serum parathyroid hormone concentration as a predictor of post‐operative hypocalcemia in dogs diagnosed of primary hyperparathyroidism and treated with parathyroidectomy

##### V Victoria^2^, C Lea^1^, D Kelly^1^, C Motta^1^, D Pollard^3^, A Salas‐Garcia^4^, K Clarke^4^, S Lombardo^5^, A Paul^6^, CA Blazquez^7^, M Seth^7^, M Walton‐Clark^8^, LG Roldan^8^, RF Geddes^9^, S Keyte^10^, D Hadju^10^

###### ^1^Southern Counties Veterinary Specialists, Ringwood, UK; ^2^Hampshire, UK; ^3^Self employed, UK; ^4^Davies Veterinary specialists, Hitchin, UK; ^5^The Ralph veterinary referral centre, Marlow, UK; ^6^Anderson Moores, Winchester, UK; ^7^Dick white Referrals, Cambridgeshire, UK; ^8^Eascott Veterinary Hospital, Swindon, UK; ^9^Queen Mother Hospital for Animals, The Royal Veterinary College, Hertfordshire, UK; ^10^Lumbry Park Veterinary Specialists, Alton, UK

Parathyroidectomy is commonly performed to treat primary hyperparathyroidism; however, post‐treatment severe hypocalcemia is a recognised and common complication in approximately 35 to 70% of dogs.

The aim of this study was to retrospectively determine whether in dogs with primary hyperparathyroidism, pre‐treatment serum PTH concentration can be used to predict development and/or severity of post‐surgical treatment hypocalcemia.

One hundred and three dogs diagnosed with primary hyperparathyroidism and treated by parathyroidectomy in seven referral hospitals were included in this retrospective study.

The computerized databases of seven referral hospitals were searched for dogs diagnosed with primary hyperparathyroidism and treated with parathyroidectomy between January 1, 2010 and December 31, 2021. Data was collected retrospectively based on the patient records, including signalment, physical examination findings, concurrent illnesses, ongoing medications, recent clinicopathologic tests results (including ionized calcium (iCa), ALP, phosphorus and PTH serum concentrations) prior to surgery and iCa concentrations after surgery.

No obvious relationship was identified between the ALP, ionized calcium, and phosphorus serum concentrations before parathyroidectomy and the development of hypocalcaemia post‐surgery. The median PTH serum concentration prior to surgery in dogs who developed hypocalcaemia post‐surgery was 232 (IQR 108–421) compared to a median of 81.5 (IQR 58.5–145.0) for eucalcaemic or hypercalcaemic dogs post‐surgery. Serum PTH concentrations of <75 pg./mL were highly unlikely to result in hypocalcaemia post‐parathyroidectomy with a sensitivity of 96.6% and specificity of 40.4% (ROC curve was 0.79 (95% CI 0.68–0.89)).

PTH serum concentrations could be used as a predictive factor of risk of development of post‐surgical hypocalcemia in dogs with primary hyperparathyroidism.

**Disclosures**

No disclosures to report

## ESVE‐O‐9

### ESVE—European Society of Veterinary Endocrinology

#### Metabolite profiling in canine pheochromocytomas, cortisol‐secreting adrenocortical tumors, and normal adrenals

##### MF Berg^1^, N Bechmann^2^, HS Kooistra^1^, ME Wolferen^1^, EPM Timmermans‐Sprang^1^, M Peitzsch^2^, S Galac^1^

###### ^1^Faculty of Veterinary Medicine, Utrecht University Utrecht Netherlands; ^2^Medical Faculty Carl Gustav Carus, Technische Universität Dresden Dresden Germany

Metabolite profiling is a powerful tool to assess adrenal physiology and disease. In humans with pheochromocytomas (PCCs), targeted metabolomics can be used to uncover underlying pathogenic variants in Krebs cycle genes such as succinate dehydrogenase subunits (SDHx) or to determine the catecholamine phenotype. In addition, steroid profiling can be applied to differentiate human adrenocortical carcinomas from adenomas. To date, there have been no metabolomic studies on adrenal tissue specimens in dogs. To explore the metabolomic profile of normal and neoplastic adrenals, we analysed different classes of metabolites (i.e., catecholamines, Krebs cycle metabolites, and adrenal steroid hormones) in canine PCCs, cortisol‐secreting adrenocortical tumors (csACTs), and normal adrenals (NAs).

Tumor tissue was obtained from client‐owned dogs with PCCs (*n* = 15) and csACTs (*n* = 10), while NAs (*n* = 10) were obtained from healthy dogs euthanized for reasons unrelated to the present study. Metabolites were measured by liquid chromatography‐tandem mass spectrometry (LC‐MS/MS) or high‐pressure liquid chromatography with electrochemical detection (HPLC‐ECD). Differences in results between two groups were assessed using the independent samples t‐test or Mann–Whitney *U*‐test, while the Kruskal‐Wallis test with Bonferroni correction was used to compare results among multiple groups.

PCCs showed significantly higher relative tissue concentrations of epinephrine than NAs (64% vs. 15%; *P* =.003), while relative tissue concentrations of norepinephrine were significantly lower in PCCs than NAs (36% versus 84%; *P* =.002). Compared to NAs, PCCs had significantly lower succinate (*P* =.007), fumarate (*P* <.001), and malate (*P* <.001) concentrations, while there were no significant differences in citrate, cis‐aconitate, isocitrate, a‐ketoglutarate, and lactate concentrations. One patient in the PCC group had an aberrant succinate:fumarate ratio, suggestive of an SDHx mutation.

The analysis of steroid hormone contents revealed a significantly higher cortisol concentration (*P* < .001) in csACTs than NAs. In addition, csACTs had significantly higher concentrations of and rostenedione (*P* = .005) and testosterone (*P* < .001). Although the end‐product of the mineralocorticoid pathway, aldosterone, was not significantly different between csACTs and NAs, csACTs had significantly higher concentrations of its precursors 11‐deoxycorticosterone (*P* = .015), corticosterone (*P* < .001), and 18‐OH‐corticosterone (*P* = .015).

Ours is the first study to report on tissue metabolomics in normal and neoplastic canine adrenal tissues. This study has shed light on the metabolic profile of adrenal tissues from healthy dogs and dogs with PCCs and csACTs, which can aid to better understanding of the pathophysiological processes involved and improvement in the diagnosis of these diseases.

**Disclosures**

Marit van den Berg: no disclosures. Nicole Bechmann: no disclosures. Hans Kooistra: member of the Global Diabetes Mellitus Advisory Board of Boehringer Ingelheim. Monique van Wolferen: no disclosures. Elpetra Timmermans‐Sprang: no disclosures. Mirko Peitzsch: no disclosures. Sara Galac: no disclosures.

## ESVE‐O‐10

### ESVE—European Society of Veterinary Endocrinology

#### Addition of cabergoline to trilostane treatment for dogs with pituitary‐dependent hypercortisolism

##### S Golinelli^1^, F Fracassi^1^, A Pöppl^2^, V De Marco^3^, D Miceli^4^, K Seo^5^, E Feldman^6^

###### ^1^University of Bologna, Ozzano dell'Emilia, Italy; ^2^Department of Animal Medicine, School of Veterinary Medicine, Federal Universit, Rio Grande do Sul, Brazil; ^3^Naya Especialidades, Sao Paulo, Brazil; ^4^Endocrinology Unit, School of Veterinary Medicine, University of Buenos Aires, F Buenos Aires, Argentina; ^5^Department of Veterinary Internal Medicine, College of Veterinary Medicine, Seoul, South Korea; ^6^Department of Medicine and Epidemiology, School of Veterinary Medicine, Davis, USA

Trilostane (T) is usually effective in controlling the hypercortisolemic state in canine pituitary‐dependent hypercortisolism (PDH), however, its effect on pituitary tumor (PT) function and growth has not been reported. Cabergoline (C), a dopamine agonist, is a potential “pituitary‐targeting” drug. This study aimed to evaluate the addition of cabergoline to trilostane in controlling PDH's clinical signs and/or blocking growth or even reducing the size of PTs.

This prospective, controlled, multicenter study included 25 dogs with PDH (PT height [PTh] ≤12 mm). Thirteen dogs were treated with T [median 0.5 mg/kg (minimum 0.3‐maximum 3.2)] and C (23 mcg/kg q48h) (TC group, TCg) and 12 dogs with only T (T group, Tg) for at least 6 months. Each dog underwent a pituitary CT scan at the beginning (T0) and the end of the study (T180‐T365); pituitary/brain ratio (PBr) was calculated from each scan. Each dog was monitored at T30 (days), T60, T120, T180, and T365 with a clinical evaluation (standardized questionnaire, higher scores were associated with worst PDH clinical control), urine specific gravity (USG), cortisol (prepill or ACTH stimulation test) and endogenous ACTH (eACTH) measurement.

Results of the questionnaire, USG, eACTH, and PBr were not significantly different between TCg and Tg at any time point. At T0 PTh was significantly higher (*P* = 0.0290) in the TCg versus the Tg. Questionnaire scores were significantly higher (*P* = 0.0101) at T30 versus T365 in the Tg. In the Tg the PTh and the PBr were significantly higher (*P* = 0.0469 and *P* = 0.0445, respectively) at T365 vs. T0. In the TCg, PTh was smaller in 4/12 dogs [1.2 mm (0.7–4.7)]; PTh did not show any change in 2/12 dogs; PTh increased in 6/12 dogs [1.7 mm (1–4.2)]; and one dog died before the end of the study. In the Tg, PTh was smaller in 5/12 [0.17 mm (0.03–0.3)], was not visualized at either T0 or T365 in one, and it increased in 6/12 dogs [2 mm (1–5.7)]. In TCg the PBr reduced in 4/12 dogs [0.07 (0.01–0.13)] and increased in 8/12 dogs [0.08 (0.06–0.43)]. In the Tg, the PBr reduced in 3/12 dogs [0.02 (0.01–0.03)], did not show any change in 3/12 dogs, and increased in 6/12 dogs [0.15 (0.06–0.35)].

In conclusion, the combination of trilostane and cabergoline treatment does not improve the control of PDH's clinical signs in comparison with trilostane treatment alone. However, cabergoline, potentially, plays a role in controlling the PT growth in PDH dogs.

**Disclosures**

Stefania Golinelli, Financial support: Ph.D. scholarship funded by Dechra, Speaking & consultancies: Dechra. Federico Fracassi, Financial support: Dechra, MSD, Monge. Speaking & consultancies: Boehringer Ingelheim, Dechra, MSD, Royal Canin, Hill's, Nestlé Purina, MYLAV Veterinary Laboratory.

## ESVE‐O‐11

### ESVE—European Society of Veterinary Endocrinology

#### Electrophoretic patterns of proteinuria in dogs with Cushing's syndrome and glomerular disease

##### J Jankovic, T Francey, A Schweighauser, J Howard, S Kittl, M Campos

###### Vetsuisse Faculty University of Bern, Bern, Switzerland

Dogs with Cushing's syndrome and dogs with glomerular disease can have similar clinical signs and electrophoretic patterns of proteinuria could help differentiating these diseases. The aim of this study was to characterize and compare the electrophoretic patterns of urine proteins between dogs with Cushing's syndrome and dogs with glomerular disease. We also aimed to evaluate changes in the electrophoretic pattern of proteinuria before and 4–6 months after trilostane treatment in dogs recently diagnosed with spontaneous Cushing's syndrome.

Dogs with spontaneous Cushing's syndrome (*n* = 18), dogs with glomerular disease (*n* = 20) and healthy dogs (*n* = 10) were included in this prospective study. Complete blood count, biochemistry, screening for infectious diseases, urinalysis with culture and urine protein‐to‐creatinine ratio (UPC) were performed. Urine proteins were evaluated using sodium dodecyl sulfate agarose gel (SDS‐AGE) electrophoresis.

Dogs with Cushing's syndrome presented mostly high molecular weight (HMW) bands while dogs with glomerular disease presented both low molecular weight (LMW) and HMW bands. Compared to dogs with glomerular disease, dogs with Cushing's syndrome had a significant lower number of LMW (*P* < *0.001)* and HMW (*P* = *0.0017)* bands and greater % of albumin (*P* < *0.001)* in the urine electrophoresis. Compared to dogs with Cushing's syndrome, dogs with glomerular disease had significantly greater % of tubular (*P* < *0.001*) and % glomerular (*P* < *0.001*) proteins, greater absolute concentrations (g/L) of albumin (*P* < *0.001*), tubular (*P* < *0.001)* and glomerular (*P* < *0.001)* proteins in the urine. The parameters with the highest discriminating power were the absolute concentrations of tubular (g/L) and glomerular (g/L) proteins in the urine. Dogs with glomerular disease also had significantly greater UPC comparing to dogs with Cushing's syndrome and healthy dogs (*P* < 0.001). In dogs with Cushing's syndrome, there were no significant differences for any of the evaluated parameters of proteinuria and urine electrophoresis before and 4–6 months after trilostane treatment.

Urine protein electrophoresis can be useful in differentiating Cushing's syndrome from glomerular disease in dogs. Dogs with Cushing's syndrome have predominantly glomerular proteinuria and this is persistent 4–6 months after starting trilostane treatment.

**Disclosures**

No disclosures to report

**Source of Funding**

SPEZKO (Die Spezialisierungskommission der Vetsuisse‐Fakultät Bern) and the Small Animal Clinic of the Vetsuisse Faculty, University of Bern.

## ESVE‐O‐12

### ESVE—European Society of Veterinary Endocrinology

#### Comparison of urinary cortisol and basal serum cortisol as a screening test for hypoadrenocorticism in dogs

##### F Fracassi^1^, A Tirolo^2^, M Galeotti^1^, C Corsini^2^, A Bertolazzi^3^, AM Tardo^1^, S Golinelli^1^, W Bertazzolo^4^, U Bonfanti^4^, F Del Baldo^1^

###### ^1^Department of Veterinary Medical Sciences, University of Bologna, Ozzano dell'Emilia, Italy; ^2^Department of Veterinary Medical Sciences, University of Parma, Parma, Italy; ^3^Anicura Clinica veterinaria Tibaldi, Milan, Italy; ^4^MYLAV Veterinary Laboratory, Passirana di Rho, Milan

The commonly recommended screening test to rule out hypoadrenocorticism (HA) is basal serum cortisol concentration (BSC). However, this test has low specificity and up to 33% of dogs with chronic gastrointestinal signs (CGS), without HA, have low BSC. This study investigates whether urinary cortisol (UC), used as a screening test, performs better than BSC in identifying dogs with HA.

The UC was determined on urine samples from dogs with HA, healthy dogs (HD) and dogs with CGS without HA. Dogs were included in the HA group if the post‐ACTH serum cortisol was <2 μg/dL and a clinical diagnosis of naturally occurring HA was made. Dogs were defined as healthy if no clinical signs were present. The CGS group included dogs for which HA was suspected based on clinical signs (e.g., vomiting, diarrhea, weakness, lethargy) but was subsequently excluded based on the BSC>2 μg/dL or ACTH stimulation test results (post‐ACTH serum cortisol >5 μg/dL). Dogs were excluded from the study if glucocorticoids were administered in the 90 days before testing. BSC and UC concentrations were measured with a chemiluminescent enzyme immunoassay (IMMULITE 2000 Veterinary Cortisol using IMMULITE 2000 XPi). The lower limit of quantification of the assay was 1 μg/dL.

One‐hundred‐and‐twenty dogs were enrolled, including 20 HA, 42 HD, 58 CGS. Six dogs of the HA group had the eukaliemic‐eunatriemic HA. Median (min‐max) UC (μg/dL) in HA dogs was 1 (1.0–1.6) and was significantly lower (*P* < 0.001) compared with HD (8.7, 1.0–58.5) and CGS (10.5, 1.0–293.0). Dogs with UC <2 were 20/20 (100%), 1/42 (2.3%) and 2/58 (3.4%) in HA, HD and CGS, respectively. Median BSC (μg/dL) in HA dogs was 1.0 (1.0–1.0) and was significantly lower (*P* < 0.001) compared with HD (7.9, 1.0–78.1) and CGS (2.1, 1.0–16.3). Dogs with BSC <2 μg/dL were 20/20 (100%), 1/42 (2.3%) and 14/58 (24.1%) in HA, HD and CGS, respectively. The area under the ROC curve to discriminate dogs with HA from CGS was 0.98 (95% CI: 0.95–1.00) and 0.96 (95% CI: 0.91–1.00) for UC and BSC, respectively.

A cut‐off value of UC <2 μg/dL revealed 100% sensitivity (95% CI: 83.2–100) and 90.0% specificity (95% CI: 79.2–96.2) in diagnosing HA. A cut‐off value of BSC <2 μg/dL revealed 100% sensitivity (95% CI: 83.2–100) and 51.7% specificity (95% CI: 38.2–65.0) in diagnosing HA. In conclusion, UC showed better performances compared with BSC in identifying dogs with HA and should be considered a possible screening test.

**Disclosures**

Andrea Corsini Speaking & consultancies: MYLAV Veterinary Laboratory, Boheringer Ingelheim Antonio Maria Tardo Speaking & consultancies: Dechra. Francesca Del Baldo Speaking & consultancies: MYLAV Veterinary Laboratory, Boheringer Ingelheim Federico Fracassi Financial support: Dechra, MSD, Monge. Speaking & consultancies: Boehringer Ingelheim, Dechra, MSD, Royal Canin, Hill's, Nestlé Purina, MYLAV Veterinary Laboratory. Stefania Golinelli Financial support: Ph.D. scholarship funded by Dechra Speaking & consultancies: Dechra Antonio Maria Tardo Financial support: Ph.D. scholarship funded by Monge. Speaking & consultancies: Dechra, MYLAV Veterinary Laboratory. Francesca Del Baldo Speaking & consultancies: MYLAV Veterinary Laboratory, Boheringer Ingelheim Walter Bertazzolo Is employed at MYLAV Veterinary Laboratory Ugo Bonfanti Is employed at MYLAV Veterinary Laboratory

**Source of Funding**

The study was supported by the ESVE Research Award

## ESVE‐O‐13

### ESVE—European Society of Veterinary Endocrinology

#### Clinical findings, treatment and outcomes in cats with spontaneous hypoadrenocorticism: 41 cases

##### E Roberts^1^, IK Ramsey^2^, R Gostelow^3^, A Latysheva^4^, L Battaglia^5^, P Silvestrini^6^, G Benchekroun^7^, K Brenner^8^, B Conversy^9^, R Ferriani^10^, A Kortum^11^, L Stammeleer^12^, N Van den Steen^13^, F Tavares^14^, J Lieser^15^, A Hibbert^16^, A Duclos^17^, T Bunn^18^, AM Boag^19^, C Arenas^20^, K Roe^21^, FK Zeugswetter^22^, Y Cui^23^, I Schofield^24^, F Fracassi^25^

###### ^1^Bristol Vet Specialists, Bristol, UK; ^2^University of Glasgow, Glasgow, UK; ^3^Royal Veterinary College, Potters Bar, UK; ^4^VetPlus Center Dubai Marina, Dubai, United Arab Emirates; ^5^Clinica Veterinaria M.E. Miller, Reggio Emilia, Italy; ^6^Ryan Veterinary Hospital, Philadelphia, USA; ^7^École Nationale Vétérinaire d'Alfort, Maisons‐Alfort, France; ^8^Centre for Animal Referral and Emergency, Melbourne, Australia; ^9^University of Montréal, Montréal, Canada; ^10^Ospedale Veterinario San Francesco, Milan, Italy; ^11^The Queen's Veterinary School Hospital, Cambridge, UK; ^12^University of Ghent, Merelbeke, Belgium; ^13^Cave Veterinary Specialists, Wellington, UK; ^14^Centro de endocrinologia Brazil, Brazil; ^15^Anicura Kleintierspezialisten Clinic, Augsburg, Germany; ^16^The Feline Centre, Langford Vets, Langford, UK; ^17^University College Dublin, Dublin, Ireland; ^18^Western Australian Veterinary Emergency and Speciality, Perth, Australia; ^19^The Royal (Dick) School of Veterinary Studies, Edinburgh, UK; ^20^Anicura Valencia Sur Hospital Veterinario, Valencia, Spain; ^21^Willows Veterinary Centre and Referral Service, Shirley, UK; ^22^University of Veterinary Medicine, Vienna, Austria; ^23^University of Bern, Bern, Switzerland; ^24^CVS (UK) Ltd, Diss, UK; ^25^University of Bologna, Bologna, Italy

This study describes the clinical findings and outcomes of a group of cats with hypoadrenocorticism, including cases without electrolyte abnormalities.

Cases of feline spontaneous hypoadrenocorticism diagnosed between 2000 and 2021 were retrospectively recruited from 14 countries and entered into an online data‐capture platform. Case inclusion required a post‐ACTH serum cortisol <55 nmol/L. Case exclusion included prior steroid administration, except for cases that had a documented increased endogenous ACTH.

Forty‐one cases were included from 24 referral centres. Median age was 5.7 years (range 0.2 to 13.8 years). There were 23 male cats and 18 female cats. The most common breed was the domestic shorthair (*n* = 25). There were 36 (87.8%) hyponatraemic and/or hyperkalaemic cases (group H) and 5 (12.2%) eunatraemic/eukalaemic cases (group E).

Median duration of clinical signs before diagnosis was 23 days (range 0 to 365 days) and was not significantly different between groups E and H. A history of vomiting was more commonly reported in group E (*p* = 0.006). On clinical examination, hypothermia (*p* = 0.034), dehydration (*p* = 0.043) and weakness (*p* = 0.043), were more commonly reported in group H.

Biochemical alterations, when assessed, included azotaemia (73.2%), increased CK (66.7%), hyperkalaemia (65.9%), hyponatraemia (65.9%), hypochloraemia (65.8%), hyperphosphataemia (55%), increased AST (40.7%) and ALT (36.6%), and hypoalbuminaemia (30%). Hyperglycaemia (35.1%) and hypoglycaemia (25.6%) were both documented. Except for hyponatraemia and hyperkalaemia, no significant differences in these biochemical alterations were detected between groups E and H. Ionized calcium was only assessed in group H and was increased in 8/14 cats. Exocrine pancreatic insufficiency (EPI) was diagnosed in four cases.

Survival to discharge occurred in 35/41 cases. Median follow‐up time for all cats was 287 days (IQR 28–775 days). Electrolyte abnormalities did not develop in group E during follow‐up (median 164 days; IQR 16–370 days). For cats that died or were euthanized, 12/20 (60%) were linked to hypoadrenocorticism. Median survival time (MST) for all‐cause mortality was 2035 days (95% CI 294–4380 days) and MST for disease‐specific mortality was not reached. Disease‐specific mortality was not significantly different between groups E and H or between cats treated with desoxycorticosterone pivalate and prednisolone/methylprednisolone (35.3%), fludrocortisone ± prednisolone/prednisone (52.9%) or prednisolone alone (11.8%).

This study provides survival data for the largest group of cats thus far reported with hypoadrenocorticism and presents information on eunatraemic/eukalaemic cases. Testing for concurrent EPI may be warranted in these cases.

**Disclosures**

Federico Fracassi: Financial support: Dechra, MSD, Monge. Speaking & consultancies: Boehringer Ingelheim, Dechra, MSD, Royal Canin, Hill's, Nestlé Purina, MYLAV Veterinary Laboratory. Ian Ramsey Speaking & consultancies: IDEXX and Dechra Imogen Schofield: Consultancy: Dechra Carolina Arenas: Consultancy: Dechra Ruth Gostelow: Funding: Royal Canin and Boehringer Ingelheim Angie Hibbert: Honaria: CEVA, BSAVA, Zoetis, ECVIM, CPD solutions, Improved CPD, London Vet Show, ISFM, WVDC, Protexin, Vetstream Grant funding: BSAVA, the Langford Trust, SAMSoc and TOSOH

**Source of Funding**

The online data‐capture platform used for the study was funded by SAMSoc (small animal medicine society in the UK)

## ESVE‐O‐14

### ESVE—European Society of Veterinary Endocrinology

#### Identification of somatic mutations in feline adrenal tumours causing primary hyperaldosteronism

##### A H Watson^1^, H M Syme^1^, M J Brown^2^

###### ^1^Royal Veterinary College, London, UK; ^2^Queen Mary University of London, London, UK

Primary Hyperaldosteronism (PA) is caused by constitutive aldosterone production by the adrenal gland. Somatic mutations have been identified in adenomas causing PA in humans; commonly affected genes include *KCNJ5*, *CACNA1D*, *ATP1A1* and *ATP2B3*. It is hypothesized that analogous somatic mutations arise in feline adrenal tumours.

DNA was extracted from adrenal tumours and non‐tumor tissue from ten cats diagnosed with primary hyperaldosteronism. Eight cases underwent whole genome sequencing (WGS), five of these cases had paired DNA available. Variant calling was used to identify somatic single‐nucleotide substitutions arising within the tumours, these were filtered to prioritise variants with predicted significant effect on protein function using SIFT. Genes with expected involvement in aldosterone secretion were selected. Variants of interest were confirmed using Sanger sequencing, and the two cases that did not undergo WGS were screened for these mutations.

Somatic mutations with predicted significant effect on protein function were identified in *CACNA1C*, *CYP21A1*, *CTNNB1* and *GNAQ*. No mutations were identified in genes commonly mutated in human aldosterone producing adenomas.

*Although CACNA1C* mutations have not been identified in humans with hyperaldosteronism, but analogous somatic mutations arise in a similar voltage gated calcium channel (*CACNA1D)* in human PA. Homozygous germ line mutations in *CYP21A2* cause congenital adrenal hyperplasia in humans. Mutations in *CTNNB1* and *GNAQ* have been identified in human adrenal tumours but are infrequent and located at different residues to those affected in the cats.

**Disclosures**

Alice Watson's PhD is funded by Biotechnology and Biological Sciences Research Council and Boehringer Ingelheim. No funding has been obtained that is directly related to the material presented in this abstract. However, general support for the feline research group that Harriet Syme is part of, and consultancies and speakers honoraria have been received from Royal Canin, Mars Petcare, Hill's, Idexx, Boehringer Ingelheim, Vetoquinol, MSD, Pfizer, Elanco and PetPlan Charitable Trust. Morris Brown has nothing to disclose.

**Source of Funding**

BBSRC and Boehringer Ingelheim (PhD funding)

## ESVE‐O‐15

### ESVE—European Society of Veterinary Endocrinology

#### Evaluation of serum steroid profile in healthy cats, cats with chronic kidney disease and cats with primary hyperaldosteronism

##### A Chamagne^2^, M Kurtz^1^, G Benchekroun^2^, S Baron^3^

###### ^1^Ecole Nationale Vétérinaire d'Alfort, Maisons‐Alfort, France; ^2^ Ecole Nationale Vétérinaire d'Alfort, Maisons‐Alfort, France; ^3^Faculty of Medicine‐AP‐HP‐Hôpital Européen Georges Pompidou, Paris, France

In humans, liquid chromatography–tandem mass spectrometry (LC–MS/MS) measurement of steroids is used in primary hyperaldosteronism (PHA) diagnosis. It may help discrimination between bilateral hyperplasia and adrenal tumor, and provides information of the underlying genetic mutation. LC‐MS/MS quantification of aldosterone concentration seems more reliable than radioimmunoassay techniques.

The aim of this study was to describe the steroid profile of healthy cats, cats with chronic kidney disease (CKD) and cats with primary hyperaldosteronism (PHA).

Ten healthy cats over 8 years old and ten cats with CKD were prospectively recruited. For PHA cats, leftover samples from another study were used. A panel of 19 steroids were measured by LC‐MS/MS and aldosterone concentration was measured using a commercially available RIA kit in healthy cats (*n* = 10), cats with CKD (*n* = 10) and PHA (*n* = 5). Kruskal‐Wallis tests were used to compare steroid concentrations between groups and Mann‐Whitney *U* tests were used for pairwise comparison when relevant.

Aldosterone measurement using LC‐MS/MS was strongly correlated with the measurement using RIA (*P* < 0.001; rho= 0.886).

All mineralocorticoids (11‐deoxycorticosterone, corticosterone, 18‐hydroxy‐11‐deoxycorticosterone, 18‐hydroxycorticosterone, and aldosterone) were significantly increased in the PHA group compared to CKD and healthy groups: *P* = 0.003, *P* = 0.002, *P* = 0.002, *P* = 0.002 and *P* = 0.002 respectively. Progesterone concentration was also significantly higher in the PHA group (*P* = 0.003). Mineralocorticoid concentrations were not significantly different between CKD and healthy groups. Cortisol concentration was significantly lower in PHA cats than in the other two (*P* = 0.017).

This study identified increase of all the mineralocorticoid hormones as well as progesterone in cats with PHA. The RIA kit used in this study seems to be well correlated to LC‐MS/MS measurement.

**Disclosures**

No disclosures to report

## ESVIM‐O‐1

### ESVIM—European Society of Veterinary Internal Medicine

#### Do dogs with either immune‐mediated polyarthritis or steroid‐responsive meningitis arteritis differ in their presentation and response to treatment compared to dogs with both diseases?

##### A Bailey^1^, G Nye^2^, S Keyte^3^, A Vicente‐Gimenez^4^, I Schofield^4^, E Roberts^2^

###### ^1^CVS (UK) Ltd, Alton, UK; ^2^Bristol Vet Specialists, CVS (UK) Ltd, Bristol, UK; ^3^Lumbry Park Veterinary Specialists CVS (UK) Ltd, Alton, UK; ^4^CVS (UK) Ltd, Diss, UK

Immune‐mediated polyarthritis (IMPA) and steroid‐responsive meningitis arteritis (SRMA) are common causes of pyrexia in young dogs. These diseases can have similar presentations and occur together. The study aims were to compare clinical presentation, clinicopathologic data and treatment response between dogs with primary IMPA, SRMA or both IMPA and SRMA.

Records of dogs that were presented to two referral centres between January 2016 and January 2023 and diagnosed with IMPA, SRMA or IMPA and SRMA, were retrospectively reviewed. Inclusion criteria included the presence of pyrexia (>39.2°C), synovial fluid analysis from ≥2 joints and cisterna magna and/or lumbar cerebrospinal fluid (CSF) analysis. Cases were classified as IMPA if neutrophilic inflammation was demonstrated in ≥2 joints and SRMA, if CSF analysis documented neutrophilic pleocytosis. Cases were excluded if a causative disease was identified, or steroids were administered in the two weeks prior to diagnosis.

Fifty dogs were included; 18 (36%) with IMPA, 19 (38%) with SRMA and 13 (26%) with both. Median age was significantly different between dogs with IMPA (64 months; IQR 30–84) versus SRMA (12 months; IQR 8–22) and between dogs with IMPA versus both (10 months; IQR 9–12) (both *P <* 0.001). The presence of spinal pain occurred less frequently in dogs with IMPA (61.1%) versus dogs with SRMA (100%) (*p* = 0.003) or both (100%) (*p* = 0.025). Articular pain occurred less frequently in dogs with SRMA (5.3%) vs. IMPA (55.6%) (*p* = 0.001). The presence of combined spinal and articular pain was not significantly different between groups. Median neutrophil‐to‐lymphocyte ratio was significantly higher for dogs with IMPA (14.9; IQR 8.8–17.5) versus SRMA (6.7; IQR 5.8–10) (*p* = 0.017) but did not differ between dogs with IMPA or SRMA and both (9.6; IQR 7–11.7).

Following diagnosis, 46 (92%) dogs initially received corticosteroid monotherapy and the median prednisolone starting dose was 2 mg/kg/day in all three groups. Initial treatment duration (separated into ≤4 and >4 months), was not significantly different between groups. Disease relapse occurred in 27.8%, 44.4% and 46.2% of dogs with IMPA, SRMA or both (*p* = 0.461).

This study demonstrates that IMPA and SRMA frequently occur together, and the presence of both diseases should be considered in dogs’ ≤12 months with pyrexia and spinal pain. Dogs with IMPA and SRMA do not appear to have a different response to treatment or relapse rate compared to dogs with IMPA or SRMA alone.

**Disclosures**

Imogen Schofield‐ Consultancy: Dechra

## ESVIM‐O‐2

### ESVIM—European Society of Veterinary Internal Medicine

#### Utility of screening diagnostic imaging in identifying potential triggers of associative immune‐mediated polyarthritis (IMPA) in dogs

##### K Bergum Hjellegjerde, L Kirby, A. Mas, A Paul

###### Anderson Moores Veterinary Specialists, Winchester, UK

Immune‐mediated polyarthritis (IMPA) is an inflammatory polyarthopathy commonly accompanied by systemic signs of illness, usually lethargy, joint pain, and pyrexia. IMPA can be classified into erosive or non‐erosive, with the latter being the most prevalent. Non‐erosive IMPA is further subdivided as idiopathic (type I) or associative (type II‐IV). Associative IMPA includes type II (reactive), type III (enteropathic) and type IV (neoplasia‐related), thus systemic investigations are commonly indicated in cases of suspected IMPA. This retrospective study aimed to evaluate the utility of screening diagnostic imaging in identifying potential causes of associative IMPA.

Medical records of 158 dogs diagnosed with IMPA at a specialist hospital between April 2015 to August 2022 were reviewed. Eight dogs were excluded due to no imaging being undertaken. The original diagnosis, and classification, were revised according to previously published literature. In those cases where typing was uncertain, a diagnosis of IMPA of unclear type was given.

Nine dogs were diagnosed with associative IMPA (6%). Primary IMPA (type 1) was diagnosed in 116 dogs (77.3%). Twenty‐five dogs (16.7%) had IMPA of unclear type. Abdominal ultrasound was performed in 75 dogs and identified a potential cause in four (4/75). Thoracic radiographs were performed in 63 dogs with no potential cause identified (0/63). Sixty‐nine dogs had abdominal CT and a potential cause was diagnosed in one (1/69). Thoracic CT was performed in 70 dogs, without a potential cause being diagnosed (0/70). Eight dogs had MRI performed with no potential cause identified (0/8). Forty‐three dogs underwent echocardiography, and a potential cause was diagnosed in one (1/43). Of the nine dogs diagnosed with associative IMPA five (55.6%) were diagnosed with type 2, three (33.3%) were diagnosed with type 3 and one (11.1%) was diagnosed with type 4.

Diagnostic imaging was helpful in diagnosing a potential cause in 6/9 (66.7%) cases diagnosed with associative IMPA; four of the dogs with type 2, one of the dogs with type 3 and one of the dogs with type 4. The prevalence of associative IMPA was low (9/150 dogs) and routine screening diagnostic imaging had a low yield in identifying a potential cause of associative IMPA in this population of dogs, however diagnostic imaging had a high yield in identifying a cause of associative IMPA in the dogs where associative IMPA was present.

**Disclosures**

No disclosures to report

## ESVIM‐O‐3

### ESVIM—European Society of Veterinary Internal Medicine

#### Evaluation of the utility of haematological ratios as biomarkers in dogs with forebrain disease

##### E Shalvey, H Vandenberghe, I Schofield, E Roberts

###### Highcroft Veterinary Referrals, Bristol, UK

The monocyte‐to‐lymphocyte ratio (MLR), neutrophil‐to‐lymphocyte ratio (NLR) and platelet‐to‐lymphocyte ratio (PLR) have been assessed as biomarkers in neoplastic and inflammatory diseases in human and veterinary medicine. In dogs with intracranial disease, only the NLR has been previously assessed and has shown promise as a biomarker in meningoencephalitis of unknown aetiology (MUA). The study aim was to evaluate additional haematological ratios in dogs with forebrain disease to assess their utility as biomarkers to differentiate cases with structural forebrain disease (SFD) from non‐structural forebrain disease (NSFD).

Records of dogs that were presented to a referral centre with neurological signs localized to the forebrain between June 2018 and January 2023 were retrospectively reviewed. Dogs with MUA or brain neoplasia were used to represent the SFD group and dogs with idiopathic epilepsy (IE) to represent the NSFD group. Inclusion criteria included a complete blood count and an MRI scan to have been performed and additionally for MUA and IE cases, cerebrospinal fluid analysis. Dogs with concurrent inflammatory disease, and/or treated with anti‐inflammatory medication or antibiotics in the 30 days preceding diagnosis were excluded. The discriminatory ability of leucocyte ratios to indicate SFD compared to NSFD were assessed with receiver operating characteristic (ROC) curves.

Eighty‐three dogs were included, comprising 30 (36.1%) with SFD and 53 (63.9%) with NSFD. Median (and IQR) MLR, NLR and PLR in dogs with SFD were 0.37 (0.28–0.53), 4.55 (2.60–6.14) and 190.07 (147.53–390.00), respectively and in dogs with NSFD were 0.25 (0.17–0.36), 2.76 (2.11–3.48) and 137.69 (94.80–188.67), respectively. There were significant differences in the MLR (*p* = 0.001), NLR (*p* = 0.003) and PLR (*P <* 0.001) between dogs with SFD and NSFD. Median age of dogs with SFD (85.5 months; IQR 72.0–108.0) was significantly higher than dogs with NSFD (46.5 months; IQR 23.0–81.0) (*P <* 0.001). The area under the ROCs for the MLR, NLR and PLR in their ability to differentiate SFD from NSFD were 0.71 (95% CI 0.60–0.83), 0.69 (95% CI 0.57–0.82), and 0.74 (95% CI 0.62–0.85), respectively. Discriminatory ability of the haematological ratios was improved when additionally accounting for age.

The study results suggest that either the MLR, NLR, or PLR can be of clinical utility as diagnostic biomarkers in dogs with forebrain disease, to help differentiate dogs with SFD from NSFD. Haematological ratios can aid decision‐making by primary care practitioners when investigating these cases.

**Disclosures**

Imogen Schofield: Consultancy for Dechra

## ESVIM‐O‐4

### ESVIM—European Society of Veterinary Internal Medicine

#### Epidemiological and hematological variables are useful to specify the underlying disease processes associated with feline non‐regenerative anemia: a retrospective study of 440 cases (2018–2022)

##### V Leynaud^1^, FA Oggiano^1^, R Lavoué^2^

###### ^1^National Veterinary School of Toulouse, Toulouse, France; ^2^InTheRes, Université de Toulouse, INRAE, ENVT, Toulouse, France

Feline non‐regenerative anemia (NRA) is a frequent condition. Little is published about their underlying processes or association with epidemiological and hematological variables.

Aims of this retrospective study were to describe a population of cats with NRA and evaluate if epidemiological or hematological characteristics enabled prediction of the underlying cause.

Epidemiological and hematological variables were reviewed for cats presented to a referral institution with confirmed NRA. Thirteen different causes of anemia were considered. Epidemiological and hematological variables were categorized. Associations between the five most relevant underlying mechanisms (chronic kidney disease (CKD), inflammation, neoplasia, retrovirus, immune‐mediated) and categorical variables were analyzed through logistic multivariate analysis, and odds ratios [95%CI] were calculated.

Four‐hundred‐and‐forty cats (median age of 10.0y [0.16–20.3], 60.4% males and 39.6% females), were included. One, 2, 3 or 4 underlying causes were found in 44.8%, 42.1%, 11.1% and 2.0% of cases, respectively. Inflammation was the most frequent cause (60.0%), followed by CKD (29.1%) and neoplasia (25.4%). Inflammatory disease was more likely (*P* < 0.01; OR = 5.5[2.412.6]), while neoplasia (*P* < 0.05; OR = 0.3[0.1–0.7]) or CKD (*P* < 0.01; OR = 0.4[0.2–0.7]) were less likely diagnosed in cats <5y. Males had a higher risk for retrovirus (*P* < 0.0001; OR = 4.7[2.2–10.1]). Hematocrit <10% was associated with retrovirus (*P* < 0.01; OR = 4.7[1.5–14.7]) or immune‐mediated disease (*P* < 0.001; OR = 133.8 [13.4–1341.1]), and decreased the risk for inflammation (*P* < 0.01; OR = 0.1[0–0.3]). Neutrophilia (*P* < 0.001; OR = 3.9[2.1–7.3]) and lymphopenia (*P* < 0.001; OR = 17.0 [3.2–90.1]) increased the risk for inflammation and lymphocytosis for neoplasia (*P* < 0.001; OR = 16.0 [3.9–65.5]). Confirmed thrombocytopenia was associated with retrovirus (*P* < 0.01; OR=4.3[1.6–11.8]).

Epidemiological and hematological variables might help to prioritize differentials and further define diagnostic procedure of feline NRA.

**Disclosures**

No disclosures to report

## ESVIM‐O‐5

### ESVIM—European Society of Veterinary Internal Medicine

#### Pharmacokinetics of orally administered immunosuppressive dosage of cyclosporine A over 10 days in healthy cats

##### S Rösch^1^, A Frommeyer^1^, J Schulte Bocholt^1^, D Grote‐Koska^2^, R Mischke^1^

###### ^1^University of Veterinary Medicine Hannover, Foundation, Hannover, Germany; ^2^Hannover Medical School (MHH), Hannover, Germany

Immunosuppressive Cyclosporine A (CsA) is used in treatment of cats with immune‐mediated diseases such as immune‐haemolytic anemia. Daily dosages of 5–20 mg/kg have been described. The aim of this study was to evaluate the development of CsA blood levels during administration of an oral dosage of 7 mg/kg q 12 h to assess when steady state conditions were achieved, and thereby the earliest time point to perform CsA measurements for drug monitoring in course of immunosuppressive therapy.

In this prospective study, six healthy domestic shorthair cats were orally administered 7 mg/kg CsA q 12 h over 10 days under well‐defined experimental conditions. On day 0 (before administration CsA) and on days 1, 2, 3, 5, 7 and 10 (12 h after the first CsA administration and immediately before the second daily CsA dose) venous EDTA blood was collected for measurements of trough CsA levels by high‐performance liquid chromatography coupled to mass spectrometry. After testing for normality, repeated‐measure ANOVA with Tukey's multiple comparisons test was used for statistical comparison of CsA values of days 1, 2, 3, 5, 7 and 10.

Statistical analysis revealed a significant increase of CsA blood levels until day 5 from day 1 (Tukey test: *P <* 0.05; ANOVA: *P* = 0.0021), whereas values on days 5 and 7 did not differ significantly from CsA concentrations on day 10 (2316 ± 894 ng/ml; mean ± standard deviation). CsA concentrations showed marked interindividual variability.

In conclusion, CsA blood levels reached steady state already after 5 days following administration of high dosages of CsA q 12 h indicating that that early time point is suitable to monitor blood levels in clinical patients. Results confirmed the well‐known, remarkable interindividual variability of CsA blood levels indicating necessity of treatment monitoring. The assessed treatment schedule resulted in significantly higher mean CsA trough values than the target range for immunosuppressive therapy (200–600 ng/mL).

**Disclosures**

No disclosures to report

## ESVIM‐O‐6

### ESVIM—European Society of Veterinary Internal Medicine

#### Prevalence of persistent hypertension and situational hypertension in a population of elderly cats in The Netherlands

##### M Knies^1^, HS Kooistra^2^, E Teske^2^

###### ^1^AniCura Diergeneeskundig Verwijscentrum Dordrecht, Dordrecht, Netherlands; ^2^Faculty of Veterinary Medicine, Utrecht University, Utrecht, Netherlands

Systemic arterial hypertension is increasingly recognized and can have serious adverse consequences in cats. Unfortunately, the act of measuring blood pressure itself may cause an increase in blood pressure, known as situational hypertension. It is currently unknown how often this phenomenon occurs. The aim of this study was to evaluate the prevalence of persistent hypertension and situational hypertension in an elderly population of cats in a first‐opinion clinic and to assess which factors were associated with systolic hypertension.

In this prospective study, systolic blood pressure was measured in 185 cats ≥10 years of age using the Doppler sphygmomanometry method according to the recommendations of the ACVIM Consensus Statement. Age, sex, body weight, body condition score, position during blood pressure measurement and apparent stress level were assessed. If a systolic blood pressure >160 mmHg was found, measurements were repeated to evaluate if persistent hypertension or situational hypertension was present.

Median systolic blood pressure for this population was 140 mmHg. The prevalence of persistent hypertension was at least 14.6% and situational hypertension at least 5.4%. Factors significantly associated with hypertension were age, higher apparent stress levels, and a sitting position during measuring. Sex, body weight or body condition score did not significantly influence systolic blood pressure.

Both persistent hypertension and situational hypertension are common in elderly cats. There are no reliable parameters to distinguish between the two, underlining the importance of a standard protocol and follow up with multiple serial measurements when hypertension is found. Age, demeanour and body position during blood pressure measurement influenced blood pressure in this population of elderly cats.

**Disclosures**

This study was supported by funding from AST Pharma B.V.

**Source of Funding**

AST Pharma B.V.

## ESVIM‐O‐7

### ESVIM—European Society of Veterinary Internal Medicine

#### A non‐invasive model of preclinical metabolic syndrome to study the effects of sodium glucose transporter‐2 inhibitors in dogs

##### JP Mochel^1^, CA Iennarella‐Servantez^1^, A Bourgois‐Mochel^1^, C Zemirline^2^, A Deflandre^2^, T Blondel^2^, A Blong^1^, D Kundu^1^, E Guillot^2^, JL Ward^1^, K Allenspach^1^

###### ^1^Iowa State University, Ames, USA; ^2^Ceva Sante Animale, Libourne, France

Data from several large placebo‐controlled studies in humans suggest that sodium glucose transporter 2 (SGLT‐2) inhibitors may be beneficial in the treatment of cardiovascular diseases regardless of the patient's diabetic status. Besides their effects on glucose excretion, SGLT2‐inhibitors positively impact systemic metabolism by reducing inflammation and oxidative stress, shifting metabolism towards ketone body production, and suppressing glycation end‐product signaling. Because of their effect on cell metabolism, SGLT‐2 inhibitors may have a therapeutic benefit in a wide array of canine diseases, including but not limited to congestive heart failure, hypertension, obesity, and chronic kidney disease. This multi‐systemic effect provides an opportunity to utilize a preclinical model that recapitulates features of preclinical metabolic syndrome (*MetS*). The objective of this research was to establish a non‐invasive and reversible model of preclinical *MetS* to study the effects of SGLT‐2 inhibitors in dogs.

Eighteen healthy adult beagle dogs were fed a high‐fat, high‐monosaccharide, low‐fiber western diet (WD) formulated based on parameters from the *National Health and Nutrition Examination Survey* for 12 weeks. Dogs were fed isocalorically based on individually calculated metabolizable energy. Blood samples were collected at baseline (BAS1) when dogs were fed their regular diet, and then again after 12 weeks of WD feeding (BAS2) for measurement of blood pressure, complete blood count, serum chemistry panel, lipid profiling, fasting blood glucose, glucagon, insulin, N‐terminal pro‐B‐type natriuretic peptide (NT‐proBNP), renin‐angiotensin‐aldosterone system peptides, and oxidative stress biomarkers. Differences between BAS1 and BAS2 were analyzed using Kruskall‐Wallis statistics followed by a post‐hoc Dunn or Wilcoxon signed rank test with continuity correction, as appropriate. *P*‐values < 0.1 were considered as statistically significant.

The WD model induced significant changes in all three markers of *MetS*, including (1) elevated blood pressure (*P* = 0.01), (2) increased fasting glucose levels (*P <* 0.001), and (3) elevated low‐density lipoprotein (*P* < 0.001) and total cholesterol (*P <* 0.001). Our results further showed a significant reduction of circulating glucagon (*P* = 0.09) and insulin (*P* = 0.02), together with an elevation of circulating NT‐proBNP levels (*P <* 0.001) and a decrease of serum bicarbonate (*P <* 0.001).

Short‐term feeding with a WD in dogs recapitulates key biological features of *MetS* as defined by the *National Institute of Health*, while inducing low‐grade metabolic acidosis and elevation of natriuretic peptides. These results support the use of the WD model to study the pharmacodynamic effects of SGLT‐2 inhibitors in dogs.

**Disclosures**

Claudine Zemirline, Audrey Deflandre, Thomas Blondel, and Emilie Guillot are employees of Ceva Sante Animale.

**Source of Funding**

Ceva Sante Animale

## ESVIM‐O‐8

### ESVIM—European Society of Veterinary Internal Medicine

#### Assessment of risk factors for sleep‐disordered breathing in dogs

##### I Niinikoski^1^, SL Himanen^2^, M Tenhunen^2^, L. Lilja‐Maula^1^, M Rajamäki^1^

###### ^1^University of Helsinki, Helsinki, Finland; ^2^University of Tampere, Tampere, Finland

Sleep‐disordered breathing (SDB) has recently been shown to occur in brachycephalic dogs using a portable neckband system. The neckband system measures the number of obstructive respiratory events per hour (Obstructive Respiratory Event Index, OREI) and snore percentage. However, excluding brachycephaly, the risk factors for SDB remain unknown. The aim of this study was to identify risk factors for high OREI value, indicative of SDB.

Forty‐six dogs were included in this prospective, observational cross‐sectional study, based on an owner questionnaire regarding the dogs’ sleeping habits. The 21 brachycephalic and 25 normocephalic dogs were selected based on their skull conformation and owner‐perceived signs of SDB. Physical examinations and grading of brachycephalic obstructive airway syndrome (BOAS) signs was performed. The neckband system was used for one night at the dogs’ homes. Univariable analysis was performed for possible risk factors, comprising brachycephaly, gender, age, body condition score, and for brachycephalic dogs, neck‐girth ratio, craniofacial ratio, and the severity of BOAS signs. The risk factors significant in univariable analysis were modeled together in multivariable regression analyses.

For all dogs, brachycephaly (*P* < 0.0001) and a body condition score of above five (*P* = 0.0007) were identified as risk factors for high OREI value. For brachycephalic dogs, the presence of moderate or severe BOAS signs (*P* = 0.007) was a risk factor for high OREI value.

**Disclosures**

Sari‐Leena Himanen acts as medical advisor for Nukute Ltd, the company making the neckband systems. The authors have no conflicts of interest to declare; none of the authors has a financial relationship with Nukute Ltd. Nukute Ltd. provided the portable neckband systems used in the study. Nukute Ltd. had no influence on study design, sample collection, interpretation of results, or preparation of the abstract.

**Source of Funding**

This study was supported by grants from Agria Djurförsäkring/Svenska Kennelklubben Forskningsfond, the Finnish Canine Health Research Fund, the Finnish Foundation of Veterinary Research, and the Finnish Veterinary Foundation. These sources had no influence on study design, sample collection, interpretation of results, or preparation of the abstract.

## ESVIM‐O‐9

### ESVIM—European Society of Veterinary Internal Medicine

#### Thecomparison of sinonasal clotrimazole distribution using trephination or catheterization in a cadaver study

##### SK Langton, CKJ Chan, RK Burchell

###### North Coast Veterinary Specialists, Sippy Downs, Australia

Canine sinonasal aspergillosis (SNA) leads to destructive rhinitis in dogs. The current treatment is topical antifungal cream. Two techniques for cream delivery in literature are frontal sinus trephination (FST) and frontal sinus catheterisation using the Seldinger technique (ST) with both showing high success rates. A previous study used contrast labelled clotrimazole cream (CLCC) to assess the distribution of cream throughout the sinuses with trephination, but there are no studies that directly compare the two techniques particularly their distribution or dosage required to provide adequate filling of the sinonasal passage (SNP). We hypothesised, based on our clinical experience, that trephination would provide better frontal sinus filling and nasal distribution than sinus catheterisation. We conducted a cadaver study using 10 dogs donated for scientific reasons that were euthanised for causes unrelated to nasal disease. Clotrimazole 1% cream was mixed with iohexol to achieve a 10% solution. Frontal sinus trephination was performed as previously described. Catheterisation of the frontal sinus was achieved using an over‐the‐wire technique. All patients had a pre and post‐contrast tomography (CT) performed to evaluate the distribution of the CLCC throughout the SNP. The SNP were subdivided into 4 regions; the frontal sinus, the cribriform plate to premolar 4, premolar 4 to canine tooth and canine tooth to nose. Filling of the SNP was calculated using the medical image platform “3D Slicer” and expressed as a percentage of sinonasal filling. Filling of the frontal sinus was 64% for the FST and 69% for the ST, which was not statistically significant (*P* = 0.73). There were no statistically significant differences for the other regions, but the percentage filling was lower for the rostral passages and the lowest filling percentages were seen from premolar 4 to the canine tooth. In conclusion, both techniques achieved similar filling percentages, but the ST was more technically challenging and took longer to perform. However, these procedures were performed in dogs without destructive nasal disease and the ST might be easier to achieve in dogs with destructive SNA.

**Disclosures**

No disclosures to report

## ESVIM‐O‐10

### ESVIM—European Society of Veterinary Internal Medicine

#### Lung microbiota assessment in dogs with bronchomalacia

##### A Fastrès, E Vangrinsven, F Billen, B Taminiau, G Daube, C Clercx

###### University of Liège, Liège, Belgium

The lungs are now recognized to harbor a low biomass and a diverse microbial community which is altered in lung diseases. However, whether lung microbiota (LM) alteration is a cause or a consequence of lung pathologies is still a subject for debate. Bronchomalacia (BM), that is defined as a regional to diffuse dynamic airway collapse of bronchi causing airflow limitation, has been inconsistently associated with bacterial infection and it is unclear whether bacteria contribute to the disease pathogenesis and progression or not.

In this study, we assessed the LM in dogs with reported bronchomalacia at bronchoscopy (BM‐dogs; *n* = 25) in comparison with healthy dogs (H‐dogs; *n* = 37). Dogs with primary or secondary bacterial and parasitic infection were excluded based on Baerman test and bronchoalveolar lavage fluid (BALF) cytology, culture and *Bordetella bronchiseptica*, *Mycoplasma cynos*, *Angiostrongylus vasorum* and *Crenosoma vulpis* qPCR*s*; as were dogs receiving antimicrobial drug in the week before sampling. LM was characterized using DNA extracted from naïve BALF subjected to 16S rRNA amplicon sequencing. Bacterial load was measured in duplicate using qPCR targeting the V2‐V3 region of the 16S rRNA gene. Bacterial composition, load and ecological parameters were calculated and compared between BM‐dogs and H‐dogs using MOTHUR v3, STAMP v2.1.3, XLSTAT v4.1 and R vegan package v 3.6.2.

Among the 25 BM‐dogs, based on BALF analysis, 3 had concomitant eosinophilic and 15 neutrophilic infiltrations, while others had a normal analysis. No significant differences between bacterial loads (*P* = 0.075) were found between groups. In BM‐dogs, the LM was dominated by *Conchiformibius*, *Streptococcus*, *Staphylococcus*, *Chryseobacterium* and *Serratia* genera and some genera of the *Pasteurellaceae* family. Linear discriminant analysis effect size revealed that the LM in BM‐dogs was enriched in 19 genera present in lower proportion in H‐dogs and commonly found in upper airways or digestive microbiota. Permutational multivariate analysis of variance detected a significant difference in LM between BM‐dogs and H‐dogs (*P* = 0.001). Finally, decreased bacterial alpha‐diversity (median and interquartile range of 8.03 (2.81–10.72) vs. 9.14 (5.51–15.08); *P* = 0.037) and evenness (0.06 (0.03–0.10) versus 0.09 (0.06–0.18); *P* = 0.011) were observed in BM‐dogs compared with H‐dogs.

This comparative study provides evidence for alteration of the LM in BM‐dogs. Because of the increase in genera from upper airways and digestive microbiota, altered LM might be secondary to an increase in the micro‐aspiration rate which is suggested in that disease. Further studies are warranted to determine if such bacteria have a role in disease onset or progression.

**Disclosures**

No disclosures to report

## ESVIM‐O‐11

### ESVIM—European Society of Veterinary Internal Medicine

#### Clinical performance of a point‐of‐care Coccidioides antibody test to diagnose pulmonary coccidioidomycosis in dogs

##### JA Jaffey^1^, AS Hanzlicek^2^, C Bolch^3^, NB Bradley‐Siemens^1^, M Thompson^4^, M Bemmerl^4^, L Chittick^1^

###### ^1^Midwestern University College of Veterinary Medicine, Glendale, USA; ^2^MiraVista Diagnostics, Indianapolis, USA; ^3^Midwestern University College of Graduate Studies, Glendale, USA; ^4^Arizona Humane Society, Phoenix, USA

Detection of anti‐*Coccidioides* spp. antibodies is an integral component in making a diagnosis of coccidioidomycosis and is most commonly achieved with agar gel immunodiffusion (AGID) or enzyme immunoassay (EIA), but turnaround time can be delayed. The sona *Coccidioides* antibody lateral flow assay (LFA) is a point‐of‐care test that has had mixed results in humans. Limited research in dogs has shown it to have moderate agreement with AGID IgG results. However, no prospective studies have evaluated its performance to diagnose pulmonary coccidioidomycosis (PC) in dogs. Therefore, the aims of this study were to determine the sensitivity and specificity of this LFA to diagnose PC and to assess agreement between results from LFA and standard AGID and EIA tests.

Client‐owned dogs with an unknown cause for clinical signs associated with respiratory tract disease and available thoracic radiographs were eligible for inclusion in this prospective cohort study. Serum coccidioidal antibodies were measured via AGID (IgM and IgG), EIA (IgG), and sona *Coccidioides* antibody LFA tests. A single reference laboratory was used for AGID and EIA testing. The LFA test was performed according to manufacturer's instructions. A diagnosis of PC was made based on positive AGID or EIA results, thoracic radiographic findings, and response to antifungal treatment. Dogs with an alternative diagnosis were categorized as other respiratory tract disease (ORTD). A receiver‐operating characteristic (ROC) curve was used to assess performance of the LFA test to diagnose PC and Cohen's kappa statistic was used for agreement tests. Significance was *P* < 0.05.

Sixty‐eight dogs (PC, *n* = 39; ORTD, *n* = 29) were included. Dogs with PC had the following distribution of positive antibody test results: LFA (87%, 34/39), AGID IgM (46%, 18/39), AGID IgG (79%, 31/39), AGID IgM or IgG (95%, 37/39), and EIA IgG (77%, 30/39). No dogs with ORTD had positive LFA results. The AUC for LFA test to predict PC was 0.94 (95% CI, 0.87–1.00) with a good sensitivity (0.87; 95% CI, 0.73–0.94) and excellent specificity (1.00; 95% CI, 0.88–1.00). Cohen's kappa statistic between LFA and AGID IgM, AGID IgG, and EIA IgG was 0.41 (95% CI, 0.20–0.63), 0.74 (95% CI, 0.57–0.90), and 0.65 (95% CI, 0.47–0.83), respectively.

The LFA is a viable point‐of‐care test to initially screen dogs with respiratory tract signs in which there is a clinical suspicion for PC. Standard coccidioidal antibody tests (AGID ± EIA) should still be performed for confirmation of diagnosis and treatment monitoring.

**Disclosures**

Andrew Hanzlicek is a co‐investigator of this study and is employed by MiraVista Diagnostics. This reference laboratory partially funded the study and performed the AGID and EIA IgG testing free of charge. The point‐of‐care tests were performed on‐site at MWU (not at MiraVista). The reference laboratory did not influence reporting of results in any way. Jared Jaffey once served on the Scientific Advisory Board at MiraVista, but no longer actively has this position.

**Source of Funding**

This study was partially funded by MiraVista Diagnostics.

## ESVIM‐O‐12

### ESVIM—European Society of Veterinary Internal Medicine

#### Generation of a comprehensive molecular cell atlas of the healthy canine lung

##### ER Rizzoli^1^, AF Fastrès^1^, LF Fiévez^2^, TM Marichal^2^, CC Clercx^1^

###### ^1^FARAH, Faculty of Veterinary Medicine, University of Liège Liège Belgium; ^2^GIGA Institute and Faculty of Veterinary Medicine, University of Liège, Liège, Belgium

Single cell RNA sequencing (scRNA‐seq) is a powerful transcriptomic technique to analyse cell expression profiles across various tissues or conditions, and to identify new cell subpopulations. It has been extensively used in human and mouse studies, and more recently for identification of cellular subpopulations in the bronchoalveolar lavage fluid of dogs. To date, the molecular state of all cell types in canine lung tissue has not been profiled. Such study will help to determine specific cell markers, often lacking in the canine species, and will also provide the foundation for further comparisons with specific lung diseases at single‐cell level, such as canine pulmonary fibrosis or neoplasia. In this context, we had a particular interest in fibroblast subpopulations and their expression profiles. Indeed, molecules expressed by fibroblasts (and by cancer‐associated fibroblasts) are of potential interest for further development of early markers of disease and of novel molecular fibroblast‐targeting therapies.

We performed droplet‐based scRNA‐seq on fresh healthy lung biopsies from three dogs. Two biopsies were collected from dogs euthanized for unrelated reasons, and one was collected from the tumour‐free area of a lung lobe resected for primary adenocarcinoma. Biopsies were systematically collected at the peripheral part of the right caudal lobe. Tissues were dissociated to obtain single‐cell suspensions, which were loaded into the Chromium Controller (10x Genomics). Clustering, visualization and gene profiling was achieved using the Seurat package in R. Distinct cell populations were identified based on canonical or literature‐described cell markers.

A total of 22,424 cells were sequenced. Four main cell compartments were identified and individually investigated: epithelial cells (EPCAM+, 5 subpopulations), immune cells (PTPRC+, 17 subpopulations), endothelial cells (PECAM1+, 5 subpopulations) and mesenchymal cells (EPCAM‐/PTPRC‐/PECAM1‐, 10 subpopulations). Clustering resolution was high enough to consistently discriminate different cell subpopulations within classical cell types such as fibroblasts, smooth muscle cells, lymphocytes or macrophages, for example. Among fibroblasts, cluster analysis highlighted subpopulations already identified in humans such as alveolar or adventitial fibroblasts. Differential gene expression profiles were defined for all 37 cell subpopulations, thus identifying new specific cell markers for all cells of the canine lung.

This is the first report of scRNA‐seq analysis of canine lung tissue, expanding our knowledge of canine distal lung cell subpopulations. This study provides the foundation for comparisons with specific lung diseases at single‐cell level, such as canine pulmonary fibrosis or canine pulmonary neoplasia, for which development of emerging therapies are cruelly required.

**Disclosures**

No disclosures to report

**Source of Funding**

Fonds de la Recherche Scientifique – FNRS (National Fund for Scientific Research) for my PhD fellowship grant. Fonds Spéciaux de la Recherche (Research Special Funds) from the faculty of veterinary medicine of the University of Liège for the project.

## ESVNU‐O‐1

### ESVNU—European Society of Veterinary Nephrology and Urology

#### Risk factors and short‐term implications associated with macroscopic nephrocalcinosis in cats with chronic kidney disease

##### PK Tang^1^, DHN Van den Broek^2^, RF Geddes^1^, RE Jepson^1^, YM Chang^1^, J Elliott^1^

###### ^1^Royal Veterinary College, London, UK; ^2^Utrecht University, Utrecht, Netherlands

Histopathological nephrocalcinosis is highly prevalent in feline chronic kidney disease (CKD). Nephrocalcinosis onset and its detection by diagnostic imaging is unclear. This prospective study assessed the prevalence of macroscopic nephrocalcinosis ultrasonographically, evaluated associated risk factors and determined its longitudinal implications.

Cats with CKD were recruited and evaluated ultrasonographically by a residency‐trained radiologist. Cats were re‐examined after ≥6 months to assess their renal function. Risk factors for nephrocalcinosis and the influence of nephrocalcinosis on CKD progression, were explored by logistic regression and linear mixed model respectively.

Thirty‐seven cats with CKD (IRIS stages 1 [*n* = 1], 2 [*n* = 26], 3 [*n* = 9] and 4 [*n* = 1]) were recruited.

At inclusion, nephrocalcinosis was deemed present (*n* = 23) or absent (*n* = 14) on renal ultrasound. Baseline plasma ionized calcium [iCa] (OR = 2.57 [95% CI: 1.20–8.91] per 0.1 mmol/L increase; *P* = 0.012), phosphate (OR = 1.59 [95%CI: 1.00–3.04] per 0.1 mmol/L increase; *P* = 0.049), creatinine (OR = 1.33 [95% CI: 1.03–2.01] per 10 μmol/L increase; *P* = 0.015) and ALT activity (OR = 2.08 [95% CI: 1.04–5.61] per 10 U/L increase; *P* = 0.036) were independent risk factors for nephrocalcinosis. Twenty‐seven cats had repeat assessments median 235 [range 175–371] days later. Plasma [phosphate] increased (*β* = 0.028 ± 0.007 mmol/L/month; *P* < 0.001) while plasma [albumin] (*β* = −0.15 ± 0.06 g/L/month; *P* = 0.02) and body weight (*β* = −0.027 ± 0.008 kg/month; *P* = 0.002) decreased over time when nephrocalcinosis was present, but not when absent. Rate of change of plasma [albumin] differed significantly between groups (*P* = 0.018).

Higher plasma [iCa], [phosphate], [creatinine] and [ALT] are independent risk factors for ultrasound‐diagnosed nephrocalcinosis. Phosphate retention was associated with nephrocalcinosis. Longer follow‐up is required to assess its effect on CKD progression.

**Disclosures**

P. K. Tang received PhD studentship funded by Royal Canin SAS. D. H. N. van den Broek received PhD studentship funded by Royal Canin SAS. R. Geddes has received funding from Petplan, Royal Canin, an RVC Internal Grant, The Academy of Medical Sciences and The Everycat Foundation; has previously had a consultancy agreement with Boehringer Ingelheim; has received speaking honoraria from Boehringer Ingelheim, Idexx and Royal Canin. R. Jepson received funding from PetPlan, Feline Foundation for Renal Research, RVC Internal Grant, PetSavers, and consultancy agreements: Boehringer Ingelheim, Merial, CEVA. Speaking honoraria: Boehringer Ingelheim, Hills Pet Nutrition, CEVA. Y. M. Chang had no conflicts of interest to declare. J. Elliott has Consultancy agreements with: Elanco Ltd, CEVA Animal Health Ltd, Boehringer Ingelheim Ltd, MSD Animal Health Ltd., Orion Incorp, Idexx Ltd, Waltham Petcare Science Institute, Invetx Inc and Zoetis Ltd. received grant funding from Elanco Ltd, Waltham Centre for Pet Nutrition, Royal Canin SAS, Idexx Ltd., CEVA Animal Health. He is a member of the International Renal Interest Society which receives sponsorship from Zoetis.

## ESVNU‐O‐2

### ESVNU—European Society of Veterinary Nephrology and Urology

#### Pilot study evaluating the detection of nephrocalcinosis using ultrasonography in cats with chronic kidney disease

##### PK Tang^1^, DHN Van den Broek^2^, M Hopkinsin^1^, RE Jepson^1^, RF Geddes^1^, YM Chang^1^, J Elliott^1^

###### ^1^Royal Veterinary College, London, UK; ^2^Utrecht University, Utrecht, Netherlands

The identification of nephrocalcinosis in cats with chronic kidney disease (CKD) is of clinical interest but the ability of ultrasound to detect nephrocalcinosis is uncertain. This pilot study compares ultrasound, micro‐computed tomography (mCT) and histopathology for identification of nephrocalcinosis.

Cats with azotaemic CKD were recruited and renal ultrasonographical cineloops were evaluated by a residency‐trained radiologist. Kidneys were grouped on the basis of nephrocalcinosis: present, suspected, or absent. When cats died, post‐mortem examination was performed. Renal tissue was evaluated using mCT for macroscopic nephrocalcinosis, and nephrocalcinosis volume‐to‐kidney tissue ratio (macro‐VN:KT) and sagittal nephrocalcinosis area‐to‐kidney tissue (macro‐AN:KT) calculated. Each kidney was subsequently bisected longitudinally, formalin fixed and paraffin embedded for microscopic nephrocalcinosis assessment using von‐Kossa staining with AN:KT (micro‐AN:KT) quantified using ImageJ. Data are presented as median [range]. Relationship between macroscopic (mCT) and microscopic (von‐Kossa) AN:KT was assessed using Spearman's correlation.

Nine kidneys, obtained from five CKD cats, were evaluated. Nephrocalcinosis by ultrasound was considered to be present in 4 kidneys, suspected in 2 and absent in 3. The macro‐VN:KT was 0.021% [0.016–0.186], 0.007% [0.007–0.008] and 0.001% [0.0003–0.008] and the macro‐AN:KT was 1.51% [0.56–2.35], 0.21% [0.15–0.28] and 0.08% [0.02–0.48], respectively. Histologically, micro‐AN:KT was 4.92% [0.40–8.49], 0.17% [0.11–0.22] and 0.21% [0.05–0.57], respectively. A strong correlation was identified between macroscopic and microscopic nephrocalcinosis (*r*_s_ = 0.77; *P* = 0.021).

This initial descriptive study has many limitations but helps to start to inform the sensitivity and specificity of renal ultrasound in detecting nephrocalcinosis.

**Disclosures**

P. K. Tang received PhD studentship funded by Royal Canin SAS. D. H. N. van den Broek received PhD studentship funded by Royal Canin SAS. M. Hopkinson had no conflicts of interest to declare. R. Jepson received funding from PetPlan, Feline Foundation for Renal Research, RVC Internal Grant, PetSavers, and consultancy agreements: Boehringer Ingelheim, Merial, CEVA. Speaking honoraria: Boehringer Ingelheim, Hills Pet Nutrition, CEVA. R. Geddes has received funding from Petplan, Royal Canin, an RVC Internal Grant, The Academy of Medical Sciences and The Everycat Foundation; has previously had a consultancy agreement with Boehringer Ingelheim; has received speaking honoraria from Boehringer Ingelheim, Idexx and Royal Canin. Y. M. Chang had no conflicts of interest to declare. J. Elliott has Consultancy agreements with: Elanco Ltd, CEVA Animal Health Ltd, Boehringer Ingelheim Ltd, MSD Animal Health Ltd., Orion Incorp, Idexx Ltd, Waltham Petcare Science Institute, Invetx Inc and Zoetis Ltd. received grant funding from Elanco Ltd, Waltham Centre for Pet Nutrition, Royal Canin SAS, Idexx Ltd., CEVA Animal Health. He is a member of the International Renal Interest Society which receives sponsorship from Zoetis.

## ESVNU‐O‐3

### ESVNU—European Society of Veterinary Nephrology and Urology

#### Evaluation of urinary podocin and nephrin as markers of podocyturia in dogs with leishmaniosis

##### V Pantaleo^1,4^, T Furlanello^2^, E Carli ^2,3^, L Solano‐Gallego^4^

###### ^1^San Marco Veterinary Clinic, Veggiano, Padua, Italy; ^2^Laboratorio di Analisi San Marco, Veggiano, Padua, Italy; ^3^Unit of Biostatistics, Epidemiology, and Public Health, Department of Cardiac, Thoracic, Vascular Sciences and Public Health, University of Padova, Padova, Italy; ^4^Departament de Medicina i Cirurgia Animals, Universitat Autònoma de Barcelona, Bellaterra, Barcelona, Spain

Renal disease is the main cause of death in canine leishmaniosis. Detection of active glomerular injury is important to identify early renal damage and prevent the development of chronic kidney disease. Active renal injury can be characterized by podocytes loss and podocyturia. Podocin and nephrin are two main proteins of podocytes that can be used as markers of podocyturia. The aim of this study was to assess and compare urinary podocin and nephrin in healthy dogs and dogs with clinical leishmaniosis.

Seventy‐two dogs were included in this case control study. Thirty‐five were healthy dogs and 37 were dogs with leishmaniosis. The diagnosis of leishmaniosis was based on clinical signs, clinical pathologic alterations compatible with this parasitic infection, positive *Leishmania* serology and positive *Leishmania* real‐time PCR in the bone marrow aspirates. Dogs with leishmaniosis were classified as Leishvet clinical stages IIa (*n* = 11), IIb (*n* = 8), III (*n* = 8) and IV (*n* = 10) and as International Renal Interest Society (IRIS) clinical stages I (*n* = 30), II (*n* = 4), III (*n* = 1) and IV (*n* = 2) and healthy dogs. Urinary podocin and nephrin concentrations were measured from urine collected via free‐catch in all dogs with a validated ELISA tests and normalized to creatinine (uPoC and uNeC, respectively).

Compared to healthy dogs, lower urinary podocin [(median values (IQR): 15.06 ng/ml (6.13) vs. 8.63 ng/ml (6.48); *P* < 0.01] and nephrin [(median values (IQR): 3.2 ng/ml (2.81) vs. 2.67 ng/ml (1.38); *P* < 0.01] were likely in infected dogs. No significant differences were observed in the median values of uPoC and uNeC between the two groups.

The dogs in stage IIa and IIb had a median concentration of urinary podocin and nephrin similar to that of healthy dogs. Dogs in Leishvet stage IV had a lower urinary podocin and nephrin than dogs in stage IIa (both *P* < 0.01), IIb (both *P* < 0.05) and healthy dogs (both *P* < 0.01). Urinary nephrin and podocin were higher in healthy dogs (both *P* < 0.01) and in dogs in IRIS stage I (*P* < 0.01 and *P* = 0.03 respectively) compared to dogs in IRIS stages II+III+IV. Urinary podocin was also higher in healthy dogs compared to dogs in IRIS stage I (*P* = 0.02). No significant differences were found for uPoC and uNeC between healthy dogs and sick dogs in different Leishvet and IRIS clinical stages.

This study showed physiological podocyturia in healthy dogs, while dogs with leishmaniosis appear to have a low concentration of podocin and nephrin in more advanced clinical stages where chronic kidney disease is more severe.

**Disclosures**

No disclosures to report

## ESVNU‐O‐4

### ESVNU—European Society of Veterinary Nephrology and Urology

#### Evaluation of urinary amylase to creatinine ratio as a marker of renal damage in dogs with leishmaniosis undergoing conventional anti‐Leishmania treatment

##### V Pantaleo^1,4^, T Furlanello^2^, L Ventura^3^, L Solano‐Gallego^4^

###### ^1^San Marco Veterinary Clinic, Veggiano, Padua, Italy; ^2^Laboratorio di analisi San Marco, Veggiano, Padua, Italy; ^3^Department of Statistical Sciences, University of Padova, Italy; ^4^Departament de Medicina i Cirurgia Animals, Universitat Autònoma de Barcelona, Bellaterra, Barcelona, Spain

Canine leishmaniosis (CanL) can cause glomerular and tubular lesions and conventional anti‐*Leishmania* treatment can potentially improve the renal damage. Plasma amylase is freely filtrated by the glomerulus and partially reabsorbed by the tubules. The aim of this study was to evaluate urinary amylase to creatinine ratio (uAm/cr) alone and in combination with urinary electrophoresis as a marker of renal damage in dogs with leishmaniosis at the time of diagnosis and post‐treatment with meglumine antimoniate and allopurinol or miltefosine and allopurinol.

Thirty‐one dogs with leishmaniosis fitted inclusion criteria and were enrolled in this retrospective cohort study. Diagnosis was based on clinical signs, clinicopathological findings compatible with this infection, a positive *Leishmania* serology, eventual visualization of amastigotes by microscope in lymph nodes or in bone marrow and a positive *Leishmania* real time PCR in bone marrow aspirates. Dogs were divided into three groups based on their initial serum creatinine (sCr) and urinary protein to creatinine ratio (UPC) values: group 1 included dogs with sCr<1.4 mg/dl and UPC ≤0.5 (*n* = 14; median sCr 0.9 mg/dl, range 0.5–1.3 mg/dL; median UPC 0.3; range 0.1–0.5), group 2 dogs with sCr<1.4 mg/dL and UPC>0.5 (*n* = 11; median sCr 0.8 mg/dl, range 0.3–0.9 mg/dL; median UPC 1.4, range 0.7–5.6) and group 3 dogs with sCr>1.4 mg/dL and a UPC>0.5 (*n* = 6; median sCr 2.9 mg/dl, range 1.7–5.8 mg/dl, median UPC 11.8, range 2.5–27.7).

At diagnosis, according to sodium dodecylsulphate‐agarose gel electrophoresis dogs had albuminuria (*n* = 3), glomerular (*n* = 0), tubular (*n* = 4) or mixed (glomerular and tubular) proteinuria (*n* = 20). After treatment dogs had albuminuria (*n* = 4), glomerular (*n* = 5), tubular (*n* = 5) or mixed proteinuria (*n* = 9). At diagnosis the median uAm/cr was 4, 270, and 1568 in groups 1, 2 and 3 respectively with a significant difference between all groups (*P* < 0.03). After treatment only dogs in group 2 had a significant reduction in uAm/cr (270 versus 8; *P* < 0.01) as well in median UPC (1.4 *versus* 0.3; *P <*0.01). At diagnosis the median values of uAm/cr were 387, 5 and 3 in dogs with mixed proteinuria, albuminuria and tubular proteinuria respectively. Post‐treatment median uAm/cr were 193, 9, 155 and 3 in dogs with mixed proteinuria, albuminuria, glomerular and tubular proteinuria, respectively.

This study suggests that uAm/cr could be a useful marker to evaluate the presence of a diffuse renal damage at the time of diagnosis and eventual recovery after anti‐*Leishmania* treatment, especially in dogs with proteinuria without azotemia.

**Disclosures**

No disclosures to report

## ESVNU‐O‐5

### ESVNU—European Society of Veterinary Nephrology and Urology

#### Analysis of serum proteomic in cats with polycystic kidney disease‐1 gene mutation

##### Ms Jiwaganont^2^, Dr. Petchdee^1^

###### ^1^Faculty of veterinary medicine, Kasetsart University Kamphaeng, Saen Thailand; ^2^Faculty of Veterinary Medicine, Kasetsart University Kamphaeng, Saen, Thailand

Feline autosomal dominant polycystic kidney disease (PKD) is common in Persian and Persian‐cross cats. Ultrasound is a reliable method for diagnosing and monitoring PKD. In the human case, the study of proteomics is essential for disease investigation and monitoring kidney function. This study aims to investigate the serum proteomic of cats with PKD1 heterozygous gene mutation and compare it with chronic kidney disease cats and Normal wild‐type cats using MALDI‐TOF MS (Matrix‐Assisted Laser Desorption/Ionization Time‐of‐Flight Mass Spectrometer). A total of 30 cats of variable breeds aged an average of 50.84 ± 31.06 months were included in this study. The study shows the protein upregulation in PKD1 mutation cats compared to wild type without chronic kidney disease signs, which is Protein phosphatase regulatory subunit, Vomeronasal type receptor, G protein coupling receptor, Phosphatidylinositol biphosphate binding and some other uncharacterized protein.

Moreover, there are proteins downregulated in PKD1 mutation cats which is a growth factor independent/cellular response to lipopolysaccharide. Different proteins exist between the PKD1 mutation gene cat and cat with wild‐type gene. However, further study needs to be done to confirm specific proteins for diagnostic or disease monitoring tools in clinical use.

**Disclosures**

No disclosures to report

**Source of Funding**

Faculty of Veterinary Medicine, Kasetsart University

## ESVNU‐O‐6

### ESVNU—European Society of Veterinary Nephrology and Urology

#### Progression of chronic kidney disease in cats following subcutaneous ureteral bypass device placement for ureteral obstruction compared to cats with idiopathic chronic kidney disease

##### Z Bennett, JS Lawson, YM Chang, J Elliott, H Syme, R Geddes

###### Royal Veterinary College, Hatfield, UK

Chronic kidney disease (CKD) is considered a sequela of ureteral obstruction and acute kidney injury managed with subcutaneous ureteral bypass (SUB) placement. Multiple clinicopathological factors are associated with CKD progression in cats with idiopathic CKD (iCKD) but this has not been explored post SUB placement. This study compared CKD progression between cats post SUB placement (SUB cats) and cats with iCKD, and explored variables associated with CKD progression in SUB cats.

Azotaemic (creatinine >177 μmol/L with concurrent urine specific gravity <1.035 or creatinine >177 μmol/L on 2 consecutive visits) and non‐azotaemic iCKD cats (SDMA >14 μg/dl) (*n* = 89), and SUB cats (*n* = 42) were retrospectively identified. Baseline clinicopathological data and CKD progression (>25% increase in creatinine) within 1 year were compared between groups. Cats with SUB blockage were censored. Baseline was 3–6 months post SUB placement or at diagnosis for iCKD cats. Multivariable logistic regression was performed to identify variables associated with CKD progression in SUB cats.

At baseline, median creatinine (SUB 176 μmol/L vs. iCKD 203 μmol/L; *P* = 0.04) and IRIS stage (SUB cats: stage 1; *n* = 9, 2; *n* = 30, 3; *n* = 3, 4; *n* = 0 vs. CKD cats: stage 1; *n* = 5, 2; *n* = 66, 3; *n* = 16, 4, *n* = 2, *P* = 0.019) was greater in the iCKD group. The proportion of cats demonstrating CKD progression within one year did not differ (SUB 16.7% vs. iCKD 29.2%; *P* = 0.137). No clinicopathological factors were independently associated with CKD progression in SUB cats.

Following SUB placement, cats are at risk of CKD progression which occurs with similar frequency to cats with iCKD.

**Disclosures**

J. Elliott disclosures—consultant for: Boehringer Ingelheim, IDEXX, Royal Canin, Ceva Animal Health, Invetx Ltd., Elanco, Dechra Ltd., Merck Animal Health, Orion Ltd., Nestle Purina, Waltham Petcare Science Institute and Zoetis R. Geddes has received funding from Petplan, Royal Canin, an RVC Internal Grant, The Academy of Medical Sciences and The Everycat Foundation; has previously had a consultancy agreement with Boehringer Ingelheim; has received speaking honoraria from Boehringer Ingelheim, Idexx and Royal Canin.

## ESVNU‐O‐7

### ESVNU—European Society of Veterinary Nephrology and Urology

#### Subcutaneous ureteral bypass placement is associated with a high complication rate but a prolonged survival in cats: a retrospective study of 94 cases (2014–2021)

##### K Mourou^1^, J Acobas^2^, JL Cadoré^1^, B Reynolds^2^, R Lavoué^2^, E Krafft^1^

###### ^1^Université de Lyon, VetAgro Sup, Campus vétérinaire de Lyon, Marcy‐l'Etoile, France; ^2^ENVT, Toulouse, France

Subcutaneous ureteral bypass (SUB) placement appears to be the treatment of choice for feline ureteral obstruction, but various complications rates and survival times have been reported.

The aims of this study were to describe complications and long‐term outcomes associated with SUB placement and to identify potential survival factors.

Medical records of cats that underwent SUB placement between 2014 and 2021 at two veterinary hospitals were retrospectively reviewed. Signalment, history, clinicopathologic and diagnostic imaging findings, duration of hospitalization, complications and outcomes were recorded. Complications were defined as minor or major (requiring surgery and/or leading to death) and classified as intraoperative, perioperative (i.e. during hospitalization), short‐ (<3 months) or long‐term (≥3 months). Survival analysis was conducted using Kaplan‐Meier curves and log‐rank tests.

Ninety‐four cats were included. In‐hospital mortality rate was 12%. Median follow‐up time was 540 days (range 11–2046). Seventy‐eight cats developed minor (73%) and/or major (41%) complications. Twelve out of 90, 25/89, 43/77 and 51/66 cats had intraoperative, perioperative, short‐term and long‐term complications, respectively. Long‐term complications included catheter mineralization and significant bacteriuria (41% each); sterile cystitis (33%); kinking, catheter failure, transmural digestive migration of SUB and perirenal abscess (8% each). Median survival time was 1482 days (range 1–2458). Three‐year survival rate was 54%. Creatinine concentration ≥200 μmol/L at discharge (*P* = 0.045) and being a purebred cat (*P* = 0.036) significantly decreased survival time.

In this large cohort, SUB placement was associated with a high long‐term complication rate. However, most complications were manageable and overall survival was longer than previously described, highlighting favorable long‐term outcomes.

**Disclosures**

No disclosures to report

## ESVNU‐O‐8

### ESVNU—European Society of Veterinary Nephrology and Urology

#### Interpretating discordant symmetric dimethylarginine and creatinine concentrations in relation to glomerular filtration rate

##### L Hardy^1^, S Tasker^2^, N Finch^1^, A Hibbert^1^

###### ^1^Langford Small Animal Hospital, Langford, UK; ^2^Linnaeus Veterinary Limited, Shirley, UK

Serum creatinine and symmetric dimethylarginine (SDMA) are widely used markers of glomerular filtration rate (GFR). Discordant creatinine and SDMA can be encountered when measuring these markers in cats, contributing to uncertainty in clinical diagnosis. The study aim was to evaluate SDMA, creatinine and GFR to understand which marker more closely reflects GFR when results are discordant.

Twenty‐seven client‐owned cats receiving radioiodine treatment (111MBq) for management of hyperthyroidism were included in this prospective longitudinal cohort study. Serum creatinine and SDMA were measured at the time of presentation (baseline) and at one‐, six‐ and 12‐months post‐treatment. GFR was determined by a slope‐intercept iohexol clearance method. Discordant SDMA and creatinine was defined as being when one of these markers was above the laboratory reference interval (>15 and >175 μmol/L, respectively) whilst the other was within or below the reference interval. Discordancy of these markers with GFR was subsequently determined. Decreased GFR was defined as <0.92 ml/min/kg. Correlation between each marker and GFR at individual timepoints was evaluated using Pearson's correlation coefficient and significance set at *P* < 0.05. Sensitivity, specificity, negative predictive value (NPV) and positive predictive value (PPV) of SDMA and creatinine for detecting decreased GFR was determined at 12 months in euthyroid cats.

At baseline all cats were hyperthyroid (TT4 >60 nmol/L) and 37% (*n* = 10) had discordant SDMA and creatinine. Of these 10 cats, the discordancy of SDMA and GFR was 100% and creatinine and GFR was 0%. At one‐, six‐ and 12‐months, 71%, 54% and 32% of cats, respectively, had discordant SDMA and creatinine. Discordancy of SDMA and GFR was greater than that of creatinine and GFR at all time‐points; SDMA versus creatinine; at one‐month 88% versus 12%, at six‐months 82% versus 18%, at twelve‐months 67% versus 33%. There was a significant inverse correlation between creatinine and GFR across all time‐points (baseline *r* = −0.745, one‐month *r* = −0.828, six‐months *r* = −0.831, 12‐months *r* = 0.748; *P* < 0.001). SDMA was correlated with GFR only at six‐ (*r* = −0.507, *P* = 0.012) and 12‐months (*r* = −0.499, *P* = 0.025). At 12‐months, the sensitivity, specificity, NPV and PPV of SDMA for detecting decreased GFR was 80%, 50%, 83% and 44%, and for creatinine was 40%, 90%, 75% and 67% respectively.

SDMA did not demonstrate superiority over creatinine as a biomarker of GFR using the reference intervals defined above. Greatest discordancy between SDMA and GFR was found when cats were hyperthyroid. SDMA was more sensitive in detecting decreased GFR in senior euthyroid cats whilst creatinine was more specific.

**Disclosures**

Lorna Hardy: IDEXX supported the project and performed the analysis of the SDMA free of charge. Petsavers funded this project. Other disclosures: Healthy Pets NZ (funding for another current research project). Severine Tasker: Previous financial support for infectious disease research from BSAVA PetSavers, European Society of Veterinary Internal Medicine Clinical Studies Fund grant, Bayer Animal Health, Journal of Comparative Pathology Educational Trust, The Langford Trust, Langford Vets Clinical Research Fund, Morris Animal Foundation, NERC/BBSRC/MRC, Petplan Charitable Trust, South West Biosciences DTP, Vétoquinol, The Wellcome Trust and Zoetis Animal Health. Member of Expertise Parasitology, as a specialist in feline medicine, supported by Vétoquinol. Member of the World Forum for Companion Animal Vector Borne diseases, supported by Elanco, and of the European Advisory Board on Cat Diseases, supported by Boehringer Ingelheim, the founding sponsor of the ABCD and Virbac and Idexx. Chief Medical Officer for Linnaeus Veterinary Limited, a company of Mars Veterinary Health, and Honorary professor of Feline Medicine, University of Bristol. Angie Hibber: Previously received honaria from CEVA, BSAVA, Zoetis, ECVIM, CPD solutions, Improve CPD, London Vet Show, ISFM, WVDC, Protexin, Vetstream. Previous grant funding from BSAVA, the Langford trust, SAMSoc and TOSOH Natalie Finch: Natalie has received direct financial support from PetSavers, Boehringer Animal Health, Zoetis, The Wellcome Trust, The Langford Trust and indirect research funding support from IDEXX.

**Source of Funding**

Petsavers

## ESVNU‐O‐9

### ESVNU—European Society of Veterinary Nephrology and Urology

#### The neutrophil‐to‐lymphocyte ratio (NLR) in cats with chronic kidney disease (CKD)

##### C Hawes^1^, G Edmunds^2^, A Hibbert^3^, S Tasker^4^, N Finch^2^

###### ^1^Royal Veterinary College, Hatfield, UK; ^2^Langford Vets, Bristol, UK; ^3^Feline Centre Langford Vets, Bristol, UK; ^4^Linnaeus Group, Solihull, UK

The neutrophil‐to‐lymphocyte ratio (NLR) is a biomarker reflecting the balance between the innate/acute (neutrophil count) and adaptive/chronic (lymphocyte count) immune response. A chronic inflammatory state is both a result of, and contributes to, progression of chronic kidney disease (CKD) in people, with NLR increasing with disease stage. NLR has not been studied in cats with CKD. The study objectives were to explore NLR in a population of cats with changing glomerular filtration rate (GFR) following resolution of hyperthyroidism and compare the NLR of cats with and without CKD.

Initial investigations involved principal component (PC) analysis on a population of cats (*n* = 27) that had received radioiodine treatment (111MBq) in which euthyroidism (TT4 ≤60 nmol/L, TSH <0.3 ng/ml) was restored over 12 months. Change in NLR (determined from the absolute neutrophil and lymphocyte counts) over 12 months was studied in cats defined as either euthyroid with normal renal function (*n* = 13) or euthyroid with persistently decreased renal function (GFR <0.92 mL/min/kg and/or increased creatinine concentration >175 μmol/l; *n* = 9). Subsequent investigations involved a case‐control study including cats with CKD (*n* = 99) defined as evidence of chronic structural or functional (creatinine concentration >175 μmol/l) change, and controls (*n* = 59) with no evidence of CKD.

Principal component analysis was performed in RStudio using the factomineR package. PC 1 and 2 were considered in the final analysis as they had eigenvalues >1, and Cos2 was used to determine the importance of each variable's contribution to the PC. Statistical analysis of change in NLR over time was performed using mixed effects model analysis and Mann‐Whitney for the case‐control population. Significance was set at *P* < 0.05.

In PC analysis, populations of cats separated along PC 1; time, renal and thyroid function were drivers of this separation (*P* < 0.001 for all three variables along PC 1). Along PC 2, cats also separated into clusters, and the NLR variable drove separation between cats within the same time point (*P* < 0.001). In the mixed effects model, NLR increased over time in euthyroid cats developing decreased renal function but remained static in euthyroid cats with normal renal function; however, this did not achieve statistical significance. NLR was significantly increased in cats with CKD compared to controls (*P* < 0.001). Median (range) NLR in cats with CKD and controls were 4.45 (0.73–22.36) and 2.42 (0.63–10.13), respectively

The study findings suggest that NLR increases in cats developing CKD indicating a shift in the balance of the innate and adaptive immune responses in this disease.

**Disclosures**

Severine Tasker disclosures Has received in the past financial support for infectious disease research from BSAVA PetSavers, European Society of Veterinary Internal Medicine Clinical Studies Fund grant, Bayer Animal Health, Journal of Comparative Pathology Educational Trust, The Langford Trust, Langford Vets Clinical Research Fund, Morris Animal Foundation, NERC/BBSRC/MRC, Petplan Charitable Trust, South West Biosciences DTP, Vétoquinol, The Wellcome Trust and Zoetis Animal Health. ST is a member of Expertise Parasitology, as a specialist in feline medicine, supported by Vétoquinol. ST is a member of the World Forum for Companion Animal Vector Borne diseases, supported by Elanco, and of the European Advisory Board on Cat Diseases, supported by Boehringer Ingelheim, the founding sponsor of the ABCD and Virbac and Idexx. ST is also Chief Medical Officer for Linnaeus Veterinary Limited, a company of Mars Veterinary Health, and Honorary Professor of Feline Medicine, University of Bristol. Natalie Finch Disclosures Natalie has received direct financial support from PetSavers, Boehringer Animal Health, Zoetis, The Wellcome Trust, The Langford Trust and indirect research funding support from IDEXX.

**Source of Funding**

The study was funded by PetSavers

## ESVNU‐O‐10

### ESVNU—European Society of Veterinary Nephrology and Urology

#### Diagnostic value of cell cycle arrest biomarkers, tissue inhibitor of metalloproteinase‐2 (TIMP‐2) and insulin‐like growth factor binding protein 7 (IGFBP7), to identify dogs with acute kidney injury

##### A Biscop, D Castelain, E Stock, K Demeyere, E Meyer, N Devriendt, E Dorn, N De Laet, D Paepe

###### Ghent University, Ghent, Belgium

The cell cycle arrest biomarkers tissue inhibitor of metalloproteinase‐2 (TIMP‐2) and insulin like growth factor binding protein 7 (IGFBP7) have shown to be valuable biomarkers for early detection of acute kidney injury (AKI) in people. This prospective observational study aimed to validate and measure TIMP‐2 and IGFBP7 in urine of dogs and to compare these values between healthy dogs, dogs with AKI, dogs with chronic kidney disease (CKD) and critically ill (CI) dogs. Forty‐two client‐owned dogs (healthy *n* = 10, AKI *n* = 11, CKD *n* = 11, CI *n* = 10) were included. Urine was sampled and stored at −80 °C within 12 h. TIMP‐2 and IGFBP7 were measured using respectively a Canine TIMP2 enzyme‐linked immunoassay (ELISA) Kit and a Canine IGFBP7 ELISA Kit. Validation for serum was performed by the companies but for urine, the intra‐assay coefficient of variation, sensitivity and matrix interference were assessed. Kruskal‐Wallis tests with Bonferroni corrections for multiple comparisons were used to compare values between groups. Urinary TIMP‐2 was significantly higher in the AKI group, when compared to healthy dogs (*P* < 0.001), dogs with CKD (*P* = 0.029) and CI dogs (*P* = 0.022). Urinary IGFBP7 could separate healthy dogs from dogs with AKI (*P* < 0.001) and CKD (*P* = 0.022), but could not differentiate the diseased groups. The product of TIMP‐2 and IGFBP7 was significantly higher in the AKI group, compared to healthy dogs (*P* < 0.001), dogs with CKD (*P* = 0.044) and CI dogs (*P* = 0.014). The product of TIMP‐2 and IGFBP7 and TIMP‐2 could differentiate dogs with AKI from other patient groups and may be valuable biomarkers for AKI in dogs.

**Disclosures**

Disclosures to report. Routine blood examinations necessary for patient recruitment was supported by IDEXX Laboratories Inc. Routine urine examination necessary for patient recruitment was partially supported by MEDVET‐AML (Algemeen Medisch Laboratorium, 2020 Antwerpen, Belgium).

## ESVNU‐O‐11

### ESVNU—European Society of Veterinary Nephrology and Urology

#### Diagnostic value of urinary to serum neutrophil gelatinase‐associated lipocalin (NGAL) ratio and fractional excretion of NGAL to differentiate dogs with acute kidney injury from healthy dogs, dogs with chronic kidney disease and critically ill dogs

##### A Biscop, D Castelain, E Stock, K Demeyere, E Meyer, N Devriendt, E Dorn, N De Laet, D Paepe

###### Ghent University, Ghent, Belgium

Urinary neutrophil gelatinase‐associated lipocalin (uNGAL) is a promising biomarker of acute kidney injury (AKI), but does not differentiate between acute and chronic kidney disease (CKD) in dogs. In people, the urinary to serum NGAL ratio (u/sNGAL) and fractional excretion of NGAL (FeNGAL) have proven to be able to differentiate AKI from CKD. This prospective observational study aimed to compare the u/sNGAL and FeNGAL between healthy dogs, critically ill (CI) dogs and dogs with AKI and CKD. Forty‐two client‐owned dogs (healthy *n* = 10, AKI *n* = 11, CKD *n* = 11, CI *n* = 10) were included. Blood and urine were sampled and stored at −80°C within 12 hours of collection. Urinary and serum NGAL were measured with a previously validated Canine NGAL enzyme‐linked immunoassays Kit. Subsequently u/sNGAL and FeNGAL were calculated. Kruskal‐Wallis tests with Bonferroni corrections for multiple comparisons were used to compare values between groups. uNGAL and u/sNGAL could differentiate the healthy group from the other groups, but could not distinguish the diseased groups. Serum NGAL was higher in the AKI group than in the healthy (*P* < 0.001) and CI group (*P* = 0.021), but did not differ from the CKD group (*P* = 1.000). The FeNGAL was higher in the AKI group, compared to the healthy (*P* < 0.001) and CI dogs (*P* = 0.018), but failed to reach significance compared to the CKD group (*P* = 0.112). In conclusion, the u/sNGAL ratio and FeNGAL were not helpful to distinguish AKI from CKD. Serum NGAL and FeNGAL could differentiate AKI from CI dogs and may have value to predict AKI in hospitalized or CI dogs.

**Disclosures**

Disclosures to report. Routine blood examinations necessary for patient recruitment was supported by IDEXX Laboratories Inc. Routine urine examination necessary for patient recruitment was partially supported by MEDVET‐AML (Algemeen Medisch Laboratorium, 2020 Antwerpen, Belgium).

## ESVNU‐O‐12

### ESVNU—European Society of Veterinary Nephrology and Urology

#### Urinary neutrophil gelatinase‐associated lipocalin (NGAL): A rapid lateral flow test in canine practice

##### F Tagliasacchi^1^, A Facchin^1^, J Zambarbieri^1^, M Cozzi^2^, S Camelliti^2^, S Lauzi^1^, S Paltrinieri^1^, A Giordano^1^, P Scarpa^1^

###### ^1^University of Milan, Lodi, Italy; ^2^PRIMA Lab SA, Balerna, Switzerland

Urinary Neutrophil Gelatinase‐Associated Lipocalin (uNGAL) has been identified as an early marker of acute kidney injury (AKI) in dogs, although its assessment, in clinical practice, is hardly feasible given the need to use ELISA assays. Furthermore, although higher levels of uNGAL are reported in AKI, similar findings are detected in other diseases. The aim of this study was to evaluate the usefulness of a canine specific point‐of‐care (POC) lateral flow immunoassay for semiquantitative uNGAL measurement in clinical practice. Measurement of uNGAL was performed on 147 canine urinary supernatants using “Dog NGAL ELISA Kit” (Bioporto) and “PRIMA Veterinary—AKI Rapid Test canine NGAL detection” (PRIMA Lab). The analyses were performed on left‐over samples previously collected for diagnostic purposes. Based on history, clinical and laboratory data, dogs were grouped as follows: controls; urinary tract infections (UTI); urolithiasis; chronic kidney disease (CKD); acute kidney injury (AKI); AKI on CKD; extrarenal inflammatory diseases. Data were analyzed by MedCalc 20.218. Kruskal Wallis and post hoc tests showed significantly higher uNGAL levels in AKI, AKI on CKD, and extrarenal inflammatory groups when compared to CKD and UTI (*P* < 0.05). In the control group, the level of uNGAL was significantly lower than all others. A further grouping, based on the presence (*n* = 30) or absence (*n* = 117) of AKI, was applied on the canine population to perform a ROC curve using ELISA results, which are generated on a continuous scale. The cut‐off of 29.7 ng/mL on the ROC curve determined a sensitivity of 96.7% and a specificity of 74.3%. Considering the POC device, the results were classified as negative (0 ng/mL), low (4 ng/mL), moderate (20 ng/mL) and high risk of AKI (90 ng/mL), based on the color chart provided by the manufacturer. A sensitivity of 97.6% and specificity of 55.6% were obtained using the cut‐off of 20 ng/mL to discriminate between AKI or non‐AKI dogs. Finally, Cohen's Kappa coefficient (*K*) of 0.72 showed good agreement between POC and ELISA results. In conclusion, good agreement was found between the POC test and the reference method in classifying patients with AKI. The relatively low specificity of both methods is due to the inherent characteristics of uNGAL, whose increase can be found in different disorders. However, based on its good sensitivity, the POC device may be a clinically relevant diagnostic tool in monitoring all those patients and/or clinical settings in which AKI may occur.

**Disclosures**

PRIMAlab Sa (manufacturer of the rapid kit) financially supported the study. Two people between the authors are currently receiving a salary from the company and one of the authors is currently receiving a doctoral fellowship, partially funded by the company.

**Source of Funding**

PRIMA Lab sa, for financial support and providing both tests for the study

## ESVNU‐O‐13

### ESVNU—European Society of Veterinary Nephrology and Urology

#### Validation of a three‐dimensional ultrasound device for non‐invasive bladder volume measurement in dogs and cats

##### D Pichard, H Kaufmann, G Benchekroun, M Canonne, L Desquilbet, C Maurey

###### Ecole Nationale Vétérinaire d'Alfort, Maisons‐Alfort, France

Evaluation of urinary bladder volume (UBV) in veterinary medicine is largely under‐used while it could be of great clinical importance for diagnosis of micturition disorders. The current reference method for measuring UBV in dogs and cats is based on invasive urethral catheterization. The evolution of medical technologies has allowed the emergence of 3D bladder ultrasound scanners of which one model has been validated in dogs only (BladderScan Prime Plus, Verathon).

The aim of this study was to validate a new non‐invasive three‐dimensional bladder ultrasound device for the measurement of UBV (Portascan 3D (Laborie)) in dogs and cats.

Between December 2021 and March 2023, dogs and cats experiencing urinary catherization for diagnostic or therapeutic issues were prospectively enrolled. The study was approved by local ethical committee. For each animal, signalment, urinalysis, and bladder content abnormalities visualized on conventional 2D ultrasound were collected. Animals were excluded if bladder presented major wall structure alterations (cystitis, intraluminal mass). Agreement between measurement of UBV estimated using the 3D ultrasound device and measurement of UBV obtained by catheterization (UBV reference) was quantified by using Bland‐Altman analysis.

Eighteen dogs and 13 cats were enrolled, including 10 male dogs, 8 female dogs, 11 male cats and 2 female cats. One male dog and one female cat were excluded because of severe bladder damage (following cystotomy and encrusted cystitis respectively). Bland‐Altman analysis revealed that 3D ultrasound device underestimated the real bladder volume by 4.9% [−14.7%; +4.8%]_95%_in dogs and overestimated the real bladder volume by 11.1% [−0.8%; +22.9%]_95%_ in cats. The error of the device (in 90% of the cases) was between −36% and +26% in dogs and between −20% and +42% in cats compared to the real bladder volume (90% limits of agreement). Analysis also revealed an excellent concordance and reproducibility between both methods with a Lyn's concordant correlation coefficient of 0.99 [0.96–1]_95%_ for dogs and 0.91 [0.59–0.97]_95%_ for cats.

This new 3‐dimensional ultrasound device is anon‐invasive, fast, and easy‐to‐use procedure and seems to provide accurate values of UBV in dogs and cats according to present results. This is the first study to assess the use of non‐invasive measurement of UBV in cats.

**Disclosures**

No disclosures to report

## ESVNU‐O‐14

### ESVNU—European Society of Veterinary Nephrology and Urology

#### Genitourinary dysplasia in dogs and cats: A case series

##### D Pichard, F Da Riz, M Kurtz, J Mortier, M Manassero, C Maurey

###### Ecole Nationale Vétérinaire d'Alfort, Maisons‐Alfort, France

Genitourinary dysplasia is a very rare condition only described in cats. This term represents several anatomical defects of both the urinary and genital tract (vaginal aplasia, impairment of uterine horns insertions…).

The aims of this case series were to describe signalment, clinical presentation, paraclinical findings and treatments. Dogs and cats diagnosed with genitourinary dysplasia between 2010 and 2022 were retrospectively included. Continuous data were presented as median [interquartile range].

Eight cats (domestic shorthair (*n* = 6), siamese (*n* = 1), main coon (*n* = 1)) and 2 dogs (Siberian Husky, long‐hair daschund) were included. The median age at diagnosis was 1.75 years [1.0–2.0]. All were female. Six cats and both dogs were neutered. Clinical signs at presentation were evolving for 9.6 months [3–15]. Six cats and both dogs were presented with storage disorder incontinence. Recurrent bacterial cystitis were reported before diagnosis (*n* = 6) and urinary tract infections were present at diagnosis (*n* = 8). All diagnosis were based on ultrasonographic and endoscopic features combined to vagino‐urethrocystography, apart from 2 cats (only by endoscopy). Genital abnormalities were vaginal aplasia (*n* = 6), abnormal insertions of uterine horns in 5 cats (insertion into the uretra (*n* = 2) or fused to the dorsal wall of urinary bladder (*n* = 3)), insertion of the vagina into the uretra (*n* = 2), vesico‐uterine fistula (*n* = 1). Urinary abnormalities were the absence of external urethral meatus (*n* = 5), bladder neck hypoplasia (*n* = 2), wide and short urethra (*n* = 5). Ureteral papillae were orthotopic, except for one dog in which a unilateral ectopic ureter was previously diagnosed and surgically treated. Urodynamic testing provided a diagnosis of urethral sphincter mechanism incompetence (USMI) in one cat and bladder instability (*n* = 1). Three cats were surgically treated (artificial hydraulic sphincter, urethrovesical plasty and hysterectomy) and one dog had hysterectomy with caudal pelvic osteotomy with good improvement. Other cases were medically managed (phenylpropanolamine, antibiotics) with mild to moderate improvement over time.

This case series describes a rare condition in cats, and is the first description in dogs. Uro‐endoscopic findings are key features for the diagnosis. Affected dogs and cats are usually young, presented with urinary incontinence and recurrent cystitis. Mechanisms of incontinence in this context need to be characterized but USMI is probably involved. In a young queen or bitch with recurrent cystitis and/or urinary incontinence, a hypothesis of genitourinary dysplasia needs to be mentioned.

**Disclosures**

No disclosures to report

## ESVONC‐O‐1

### ESVONC—European Society of Veterinary Oncology

#### Biologic behaviour of canine preputial, scrotal and vulvar cutaneous mast cell tumours: a single‐centre retrospective analysis of 102 dogs (2002–2022)

##### Ž Žagar, S Kuehnel‐Lawatsch, J Schmidt, T Buehler, M Kessler

###### Small Animal Clinic Hofheim, Hofheim, Germany

Anecdotally, mast cell tumours (MCTs) of the genital region have been associated with a poorer prognosis. However, no larger study has focussed exclusively on MCTs in this region. The aim of this retrospective study was to describe clinicopathologic aspects in dogs with cutaneous MCTs originating from the preputial (pMCT), scrotal (sMCT) and vulvar (vMCT) region.

Dogs (*n* = 102) with surgically resected solitary pMCT (*n* = 32), sMCT (*n* = 43) and vMCT (*n* = 27) diagnosed and treated between 2002 and 2022 were included. Median age was 8 years (range, 2–14.3 years) with no significant difference between tumour locations. Median maximal tumour diameter was 1.5 cm (range, 0.5–4.0 cm). Tumours were graded according to Patnaik (pMCT: 62.5% grade I, 31.2% grade II, 6.3% grade III; sMCT: 44.2% grade I, 41.9% grade II, 13.9% grade III; vMCT: 37% grade I, 51.9% grade II, 11.1% grade III) and Kiupel (pMCT: low‐grade/high‐grade 90.6%/9.4%; sMCT: low‐grade/high‐grade 83.7%/13.9%, undetermined *n* = 1; vMCT: low‐grade/high‐grade 88.9%/11.1%). Grade was prognostic (Patnaik grades I‐II versus grade III mST of 30.5 versus 9.5 months, respectively (*P* = 0.0025); Kiupel low‐ versus high‐grade mST of 31.5 versus 9.5 months, respectively (*P* = 0.0006)). Surgical margins were histologically clean in 91 of the 98 cases evaluated (pMCTs 26/29 (89.7%), sMCTs 41/42 (97.6%), vMCTs 24/27 (88.9%)). There was no significant difference in mSTs in dogs with clean as opposed to unclean margins (25 versus 18 months; *P* = 0.8338). All but one incompletely resected tumours were Kiupel low‐grade. At time of presentation, 30/102 dogs (29.4%; pMCT 37.5%, sMCT 25.6%, vMCT 25.9%) including 24/91 (26.4%) with Patnaik grades I or II tumours and 24/89 (27.0%) with Kiupel low‐grade tumours had histologically or cytologically confirmed regional (superficial inguinal) lymph node (iLN) metastases. Three dogs also had confirmed or highly suspicious sublumbar LN metastases. Surgical removal of the regional LN was performed in 49 dogs. Irrespective of LN resection, dogs with and without LN metastases had an mST of 15 and 30.5 months, respectively (*P* = 0.0147). Dogs with normal iLNs on palpation and without iLN removal had an mST of 30.5 months. There was no significant difference in mSTs in dogs with microscopic (HN1‐2) compared to macroscopic (HN3) iLN metastasis (30 versus 12.5 months; *P* = 0.1).

In conclusion, cutaneous MCTs in these regions do not seem to be inherently more aggressive or associated with a worse prognosis compared to similar grade and stage cutaneous MCTs in other locations. Patnaik/Kiupel grades and LN status are prognostic.

**Disclosures**

No disclosures to report

## ESVONC‐O‐2

### ESVONC—European Society of Veterinary Oncology

#### Canine large granular lymphocyte (LGL) lymphoma: A retrospective study of 42 cases

##### P Loddo, L Schiavo, N Habic, R Finotello, J Dobson, C Hare

###### Queen's Veterinary School Hospital, Cambridge, UK

In humans, disorders of large granular lymphocytes (LGL) comprise a number of conditions, ranging from benign expansion to aggressive malignant proliferation. Whilst LGL lymphoma is recognised as an aggressive entity in cats, only scattered case reports and small cases series have so far defined this disease in dogs. Use of the term LGL in the context of canine chronic lymphocytic leukaemia (CLL) of granular lymphocytes creates an overlap in terminology between diseases.

The aim of this retrospective study was to describe the presentation, clinicopathological findings, anatomical distribution and treatment responses in a larger cohort of dogs with LGL lymphoma.

The databases of three UK veterinary referral hospitals were searched for cytological diagnoses of canine LGL lymphoma from 2008 to 2022. Cases were included if there was involvement of solid tissues and cells were described as having a large nucleus with pink, perinuclear cytoplasmic granules. A total of 42 cases were eligible for inclusion. Dogs suspected of having CLL were excluded.

Patients had a mean age of 7 years and were mostly presented with gastro‐intestinal or non‐specific symptoms. Anaemia was present in 22/40 dogs (55%), and was mostly mild or moderate. Thrombocytopenia was found in 16/34 dogs (47%, excluding those with clumped platelets) and often moderate or severe. Neutropenia was uncommon (4/41, 9.8%), though in two cases it was profound. Nine patients had bi/pancytopenia at presentation and circulating atypical cells were found in 8/41 (19.5%).

Hypoalbuminemia was recorded in 24/39 (61.5%) patients, and in 87.5% of these was moderate or severe. Elevated hepatic parameters were common, alanine‐aminotransferase and alkaline‐phosphatase both being raised in 26/40 dogs (65%). Total bilirubin was increased in 13/39 (33.3%) and cholesterol decreased in 11/36 cases (30.6%).

The most commonly affected anatomical locations were the gastro‐intestinal tract, liver and spleen, followed by the central nervous system, urogenital tract and mesenteric lymph nodes.

19/42 patients (45.2%) were treated with a variety of chemotherapy agents or L‐asparaginase, with or without steroids. The overall response rate was 73.3%, clinical results lasting a median of 45 days. However, overall survival time was poor (median of 6.5 days), with many patients euthanised at or soon after diagnosis.

In conclusion, canine LGL lymphoma, similarly to its feline counterpart, seems to represent an aggressive condition, often associated with poor treatment response and prognosis. However, few dogs registered longer control of their disease after chemotherapy treatment.

**Disclosures**

No disclosures to report

## ESVONC‐O‐3

### ESVONC—European Society of Veterinary Oncology

#### Treatment and outcome of myeloma‐related disorders in cats: a multicentric retrospective study of 50 cases

##### L Lecot, I Desmas‐Bazelle, S Benjamin, P De Fornel, G Chamel, F Ponce, M Kornya, L Desquilbet, M Canonne, C Beaudu‐Lange, C Ibisch, D Sayag, G Benchekroun, J Béguin

###### Marcy L'étoile, France

Myeloma‐related disorders (MRD) are poorly documented feline neoplasms. Our aims were to describe their clinical, clinicopathologic, imaging findings, response to treatment, survival and to identify factors associated with shorter survival times.

Cats with hyperglobulinemia confirmed by serum protein electrophoresis, and either: intramedullary plasmacytosis above 10%, marked cyto‐nuclear atypia when intramedullary plasmacytosis between 5% and 10%; or cytologically/histologically confirmed visceral infiltration, were retrospectively included.

Fifty cats were included. Castrated males and domestic shorthair were overrepresented. Staging consisted of abdominal ultrasound (*n* = 42), thoracic radiographs (*n* = 26), and/or full skeletal survey (*n* = 38). Osteolytic lesions were documented in 11/38 cats (29 %). Bone marrow plasmacytosis, splenic, or hepatic involvement were present in 17/27 cats (63%), 36/42 (86%), and 27/38 cats (71%), respectively. Anemia was reported in 33/49 cats (67%), hypoalbuminemia in 29/49 cats (59%), and thrombocytopenia in 16/47 cats (34%). Cats received melphalan/prednisolone (*n* = 19), cyclophosphamide/prednisolone (*n* = 10), chlorambucil/prednisolone (*n* = 4), prednisolone (*n* = 4), or other (*n* = 4). Overall response rates to melphalan, cyclophosphamide, and chlorambucil were 88%, 91%, and 100%, respectively. Adverse events to melphalan or cyclophosphamide occurred in 65% and 23% of cases. Median survival time (MST) was 122 days (range: 0–1403). MST was not significantly associated with chemotherapy protocol. MST was significantly shorter for cats with anemia (89 vs. 356 days; *P* = 0.014), thrombocytopenia (22 vs. 156 days; *P* = 0.004), hypoalbuminemia (78 vs. 367 days; *P* = 0.033) and low albumin‐to‐globulin ratio (<0.2) (67 vs. 322 days; *P* = 0.024).

This study confirmed the guarded prognosis of feline MRD and identified factors associated with shorter survival times.

**Disclosures**

No disclosures to report

## ESVONC‐O‐4

### ESVONC—European Society of Veterinary Oncology

#### Flow cytometry for detection and quantification of nodal metastasis in dogs with treatment‐naïve firstly occurring cutaneous mast cell tumour

##### C Chalfon ^1^, R Riondato ^2^, S Sabattini ^3^, S Iussich ^2^, A Renzi ^3^, G Iamone ^2^, E Morello ^2^, M Martano ^4^, L Ciammaichella ^3^, G Ghisoni ^3^, C Agnoli ^3^, L Marconato ^3^

###### ^1^ Veterinary Center of Turin, Turin, Italy; ^2^ University of Turin, Turin, Italy; ^3^ University of Bologna, Bologna, Italy; ^4^ University of Parma, Parma, Italy

An accurate staging including the evaluation of sentinel lymph node (SLN) status by means of cytology and/or histopathology is of utmost importance to guide treatment recommendations and predict prognosis in dogs with cutaneous mast cell tumours (cMCTs). Flow cytometry (FC) is a minimally invasive technique that is helpful in the identification and quantification of mast cells (MCs) in LNs of dogs with cMCTs. Accordingly, the use of FC may improve the accuracy of staging.

The aim of this multi‐institutional prospective study was to assess the impact of the level of SLN infiltration, detected by FC, on time to progression (TTP) and tumor‐specific survival (TSS), and to establish a cut‐off value of prognostic significance.

Dogs with newly‐diagnosed, treatment‐naïve, histologically confirmed cMCT were eligible for enrolment; all dogs underwent complete staging, including SLN mapping, cytology and FC of SLN, and surgical excision of the cMCT along with sentinel lymphadenectomy. If indicated, adjuvant medical treatment was administered. The influence of potential prognostic variables (e.g., stage, substage, histologic grade) and the impact of the levels of SLN infiltration detected by FC on TTP and TSS were investigated.

Forty‐one dogs were included. Overall, 79 SLNs were surgically removed.

The two FC cut‐offs that best associated with Weishaar HN2 and HN3 classification (0.05% and 0.1%, respectively) were used for the percentage of nodal infiltration in multivariable analysis. On multivariable analysis, Kiupel high‐grade was the only variable associated with an increased risk of tumor progression (HR: 10.8; *P* = 0.02); no variable was associated with an increased risk of tumor‐related death. The level of SLN infiltration in dogs experiencing disease progression was significantly higher than that of dogs without progression (median, 9.60 vs. 0.03; *P* < 0.001). However, no cut‐off with prognostic significance was identified. Median follow‐up was 235 days (range, 20–516). Median TTP and median TSS were not reached.

No evident association between the degree of SLN infiltration detected by FC and outcome was identified. Different thresholds should be investigated; it may be possible that a longer follow‐up could bring out events (disease progression, death), thereby establishing a prognostic cut‐off.

**Disclosures**

Form of financial support for the study

**Source of Funding**

Clinical Studies Fund‐ Purina ECVIM

**ECVIM CSF Funding**

Yes

## ESVONC‐O‐5

### ESVONC—European Society of Veterinary Oncology

#### Evaluation of programmed death‐ligand 1 expression in canine lymphomas using flow cytometry

##### A Ubiali^1^, P Moretti^1^, F Riondato^2^, F Sini^2^, R De Maria^2^, D Stefanello^1^, S Comazzi^1^, L Aresu^2^, L Marconato^3^, V Martini^1^

###### ^1^Department of Veterinary Medicine and Animal Sciences, University of Milan, Lodi, Italy; ^2^Department of Veterinary Sciences, University of Turin, Torino, Italy; ^3^Department of Veterinary Medical Sciences, University of Bologna, Bologna, Italy

Immune checkpoint molecules, including Programmed Death‐Ligand 1 (PD‐L1) play a key role in cancer immunotherapy. PD‐L1 protein has been retrieved on the surface and within several different canine cancer cells. Increased amounts of mRNA encoding for PD‐L1 by RNA‐scope have been associated with worse prognosis in dogs with Diffuse Large B‐Cell Lymphoma. Flow cytometry (FC) may represent an advantageous technique to detect PD‐L1 expression in dogs with lymphoma compared to other techniques, because it can be performed at the same time and session of diagnosis analysis without any further sampling needed than those of diagnostic purpose. The aim of this study was to assess the membrane expression of PD‐L1 of different canine lymphoma subtypes by means of FC. Surface expression of PD‐L1 on neoplastic cells was assessed on surplus material from nodal samples obtained for diagnostic purpose from 46 dogs with diagnosis of lymphoma based on cytology and FC. Additional data recorded included: patient signalment, FC phenotype, cytologic (updated Kiel) and WHO histologic subtype. PD‐L1 expression was calculated as the ratio between median fluorescence intensity (MFI) of neoplastic cells stained with anti‐PD‐L1 antibody and an isotypic control. MFI‐ratio was arbitrarily considered negative if ≤1, low‐positive if >1 and <1.2, positive if ≥1.2 and <1.7 and highly‐positive if ≥1.7.

Thirty‐three (71.7%) dogs had B‐cell lymphoma (BCL), while 13 (28.3%) had T‐cell lymphoma (TCL). Seventeen (37%) dogs were negative (11 BCL and 6 TCL), 11 (23.9%) low‐positive (6 BCL and 5 TCL), 12 (26.1%) positive (10 BCL and 2 TCL) and 6 (13%) highly‐positive (all BCL). Median MFI‐ratio of positive samples was 1.41 (1.01–2.69), 1.12 (1.02–1.49) for TCL and 1.47 (1.01–2.68) for BCL. No difference in the prevalence of PD‐L1 positivity was detected between neither FC phenotypes nor cytologic and histologic subtypes. However, positive BCL had significantly higher levels of protein expression compared to TCL. This is in line with the literature, thereby confirming FC as a reliable technique for the assessment of PD‐L1 expression in canine lymphomas. Statistical power of analysis resulted >80% when comparing expression of PD‐L1 between positive BCL and TCL, but dropped when PD‐L1 negative samples were included. Considering that, the lack of significance might also depend on the limited number of samples evaluated. Lastly, the significant difference of PD‐L1 expression between B and T phenotype was quantitative rather than qualitative, suggesting that TCL might express PD‐L1 as well but with a lower intensity.

**Disclosures**

No disclosures to report

## ESVONC‐O‐6

### ESVONC—European Society of Veterinary Oncology

#### Flow cytometry expression in canine T cell lymphoma; presentation, prognosis and response to lomustine‐based protocols used in the naïve setting

##### F Lyseight, E Villiers, C Pittaway

###### Dick White Referrals, Cambridge, UK

Canine T cell lymphoma is usually considered an aggressive disease, often characterised by poor response to chemotherapy and short progression free times. Flow cytometry expression in canine T‐cell lymphoma (loss of CD3, co‐expression of CD79 and a combined patten of CD4+/CD8−/MHCII−) have previously been reported to be prognostic. Although lomustine based chemotherapy is often used as first line treatment, no studies have assessed expression of flow cytometry markers and treatment response. This is a retrospective study looking at canine T‐cell lymphoma diagnosed on flow cytometry and treated in the naive setting with protocols containing lomustine.

A total of twenty‐nine dogs were included with multicentric (*n* = 20), mediastinal (*n* = 4), hepatosplenic (*n* = 4), and cardiac (*n* = 1) presentation. The majority of the population were stage III, IV or V. Dogs were treated with LOPP protocol (*n* = 17), LOP (*n* = 9), LP (*n* = 2), and LAP (*n* = 1). The overall response after first treatment was 86% with complete remission 52%, partial remission 31% and progressive disease 14%. Of responders, 67% relapsed and response lasted on average 80 days (mean follow‐up 135 days). At the time of analysis 83% were dead and mainly due to lymphoma (96%).

All cases were positive for CD3 and CD45. The pattern most repeated amongst complete responders was CD4+/CD8−/CD5+. Most of the partial responders were CD4+/CD5+/CD79+. The non‐responders expressed CD8+/MHC II+. The only patient achieving stable disease expressed only CD3 and CD45.

In the current study, most responders expressed CD4 contrary to non‐responders that all expressed CD8. Co‐expression of CD79 was higher in partial responders than complete or non‐responders. No overt differences in flow cytometry patterns could be associated with response to lomustine. This study suggests expression of CD8 and CD79 may be associated with a worst response.

**Disclosures**

No disclosures to report

## ESVONC‐O‐7

### ESVONC—European Society of Veterinary Oncology

#### Peripheral blood and bone marrow involvement do not worsen outcome in 50 dogs with nodal peripheral t‐cell lymphoma receiving alkylating‐rich chemotherapy

##### C Agnoli ^1^, S Comazzi ^2^, S Sabattini ^3^, V Martini ^2^, F Riondato ^4^, L Aresu ^4^, D Stefanello ^2^, L Gamberini ^3^, L Marconato ^3^

###### ^1^ Department of Veterinary Medical Sciences, University of Bologna, Ozzano dell'Emilia, Italy; ^2^ Department of Veterinary Medicine and Animal Sciences, University of Milano, Lodi, Italy; ^3^ Department of Veterinary Medical Sciences, University of Bologna, Ozzano Dell'Emilia, Italy; ^4^ Department of Veterinary Sciences, University of Torino, Grugliasco, Italy

The prevalence and prognostic role of peripheral blood (PB) and bone marrow (BM) involvement vary between the different WHO lymphoma subtypes. Peripheral T‐cell lymphoma (PTCL) is among the most aggressive canine lymphomas, with poor response to conventional chemotherapy and short survival time. Prevalence and prognostic role of PB and BM infiltration in dogs with PTCL are currently unknown.

Aims of the present study were to retrospectively investigate the prevalence of PB and BM infiltration in dogs with PTCL, assess their influence on outcome and establish cut‐off values of prognostic significance.

Medical records of 2 Oncology Centers were reviewed to identify dogs with treatment‐naïve and histologically confirmed PTCL. To be included, dogs had to undergo complete staging, including flow cytometry (FC) on lymph nodes, PB and BM samples. Dogs had to be treated with an alkylating‐rich protocol and have a complete follow‐up. Response was classified based on RECIST criteria at each chemotherapy session. End‐staging was carried out at the end of treatment, and PB and BM were re‐evaluated in all cases by FC. Endpoints were time to progression (TTP) and lymphoma‐specific survival (LSS). The relationship between TTP/LSS and extent (>1%, >3%, >10%) of PB and BM infiltration was assessed.

Fifty dogs were included: 39 (78.0%) had stage V disease, 7 (14.0%) stage IV, 3 (6.0%) stage III and 1 (2.0%) stage I.

Malignant cells were identified in PB (PB>1%) in 33 cases (66%), with a median percentage of 2.6% (range 0.0—31.0%). BM infiltration (BM>1%) was identified in 30 (60%) dogs, with a median percentage of 1.3 % (range 0.1–66.5%).

Forty‐six (92.0%) dogs achieved complete (*n* = 32) or partial (*n* = 14) response, 4 (8.0%) progressed. Median TTP was 90 days (95% CI 66–114). At data analysis closure, 42 (84.0%) dogs had died because of lymphoma. Median LSS was 154 days (95% CI 129–179). In multivariable analysis, lack of complete response (HR = 10.68, *P* = .003 for TTP; HR = 2.86, *P* = .018 for LSS) and extra‐nodal site other than rate of PB and BM involvement (HR = 6.58, *P* = .025 for TTP) were significantly associated with an increased risk of progression and death. The extent of PB and BM infiltration was not significantly different between dogs experiencing disease progression before or after 6 months from the beginning of chemotherapy.

Even though PB and BM involvement occurs frequently in dogs with PTCL, they do not appear to influence outcome. Thus, PB and BM evaluation by FC may not be always mandatory in these patients.

**Disclosures**

No disclosures to report

## ESVONC‐O‐8

### ESVONC—European Society of Veterinary Oncology

#### Comparison of first‐line CHOP‐19 and CHOP‐25 in the treatment of canine aggressive peripheral nodal B‐cell lymphomas: a European multicentric retrospective cohort study

##### CIK Hawkes^1^, J Morris^2^, S Bavcar^3^, C Auquier^4^, S Benjamin^5^, J Borrego^6^, S Bottin^7^, O Davies^8^, I Desmas‐Bazelle^9^, A Einhorn^10^, CM Figueroa‐Gonzalez^9^, K Holenova^11^, E Kritsotalaki^12^, K Peak^13^, K Smallwood^14^, E Treggiari^15^, P Valenti^16^, M Virgen^17^, S Ray^2^, Q Fournier^7^

###### ^1^University of Edinburgh, Edinburgh, UK; ^2^University of Glasgow, Glasgow, UK; ^3^Small Animal Specialist Hospital, Sydney, Australia; ^4^University of Liege, Liege, Belgium; ^5^Davies Veterinary Specialists, Hitchin, UK; ^6^Clinico Hospital Auna Especialidades Veterinarias, Valencia, Spain; ^7^Aura Veterinary, Guildford, UK; ^8^Highcroft Veterinary Referrals, Bristol, UK; ^9^Royal Veterinary College, London, UK; ^10^Koret School of Veterinary Medicine, Rehovot, Israel; ^11^Wear Referrals, Bradbury, UK; ^12^Southfields Veterinary Specialists, Basildon, UK; ^13^Anderson Moores, Winchester, UK; ^14^North Downs Specialist Referrals, Bletchingley, UK; ^15^Centro Specialistico Veterinario, Milan, Italy; ^16^Clinica Veterinaria Malpensa, Milan, Italy; ^17^Auna Especialidades Veterinarias, Valencia, Spain

Aggressive peripheral nodal B‐cell lymphomas (APNBCL) are the most common presentations of lymphoproliferative disease in dogs. Over the last 20 years, the multiagent CHOP‐based chemotherapy protocol has been widely accepted as the gold standard first‐line treatment. The two versions CHOP‐25 and CHOP‐19 are the most commonly prescribed, but have never been directly compared. Both protocols are designed as four identical cycles with alternating weekly chemotherapy administrations of vincristine, cyclophosphamide and doxorubicin, which are spaced to biweekly administrations in the last two cycles of CHOP‐25, thereby decreasing the dose‐intensity and increasing the duration of the protocol. The primary aim of this European multicentric retrospective study was to compare the outcome of dogs diagnosed with APNBCL and treated with a first‐line CHOP‐19 or CHOP‐25 protocols.

Five hundred and four dogs from 16 oncology referral centres, diagnosed on cytology or histology with APNBCL between 2014 and 2021, were included. One hundred and fifty‐five dogs were treated with CHOP‐19 and 349 dogs with CHOP‐25. Age, body‐weight, breed, substage, prevalence of anaemia, and protocol completion rate were not significantly different between the two groups.

The 6‐month, 1‐year, and median progression‐free survival (PFS) were 54.7% (95% CI: 46.4–62.9), 12.8% (95% CI: 7.9–19.3), and 193 days (95% CI: 138–235) with CHOP‐19; 53.4% (95% CI: 47.8%–58.9%), 13% (95% CI: 9.5–17.1), and 196 days (95% CI: 160–222) with CHOP‐25.

The 1‐year, 2‐year and median overall survival (OS) were 28.8% (95% CI: 21.1–37.6), 5.6% (95% CI: 2.3–11.2), and 252 days (95% CI: 195–314) with CHOP‐19; 34.4% (95% CI: 29.0–40.3), 8.4% (95% CI: 5.4–12.2), and 281 days (95% CI: 232–308) with CHOP‐25

There was no statistically significant difference (*P* > .05) in any of the PFS and OS variables between the two protocols.

This study confirmed a similar outcome between dogs with APNBCL treated with CHOP‐25, or with the shorter version CHOP‐19.

**Disclosures**

No disclosures to report

## ESVONC‐O‐9

### ESVONC—European Society of Veterinary Oncology

#### Incidence of gastrointestinal toxicity and treatment outcome in dogs with multicentric lymphoma receiving doxorubicin or epirubicin as part of a multi‐agent chemotherapy protocol

##### E Treggiari ^1^, E Catania ^1^, I Forzisi ^1^, K Boyd ^2^, P Valenti ^3^, R Finotello ^2^

###### ^1^ Centro Specialistico Veterinario, Milan, Italy; ^2^ University of Liverpool, Neston, UK; ^3^ Clinica Veterinaria Malpensa AniCura, Samarate, Italy

Canine non‐Hodgkin's lymphoma (cNHL) is usually treated with multidrug protocols that include an anthracycline (AC), namely doxorubicin (DOX) or epirubicin (EPI). Very few reports have focused on comparing the gastrointestinal (GI) toxicity of these two drugs.

The aim of this study was to analyse the frequency and severity of GI adverse events (AEs) in dogs with multicentric B or T‐cell cNHL receiving a multi‐agent protocol including either DOX (*DOX‐Group*) or EPI (*EPI‐Group*), and to compare outcomes.

The medical database of four institutions was searched for dogs with a confirmed diagnosis of cNHL, undergoing chemotherapy with a multidrug protocol, between 2010 and 2022. Patients with known GI involvement, GI lymphoma or pre‐existent chronic GI conditions were excluded. Other exclusion criteria were lack of any staging procedures, lack of immunophenotype and dose reductions at the time of the first AC administration.

Variables evaluated included signalment, stage, substage, immunophenotype, concurrent use of steroids, type of AC used, dose and remission status following the first administration of AC. Gastrointestinal toxicity was graded according to the Veterinary Cooperative Oncology Group (VCOG) criteria for AEs. Outcome measures including duration of first remission (DFR) and overall survival time (OST) were evaluated for both groups.

A total of 142 dogs were included: 79 received EPI and 63 received DOX. Median age was 8 years (range 6 months‐15 years) and median body weight was 23 kg (range 5–60 kg). In the *DOX‐Group*, 54% of dogs experienced GI AEs, versus 33% in the *EPI‐Group*. The difference was statistically significant (*P* = 0.012); however, significance was lost when looking at VCOG AEs ≥ 3. None of the variables analysed influenced the development of GI AEs.

The median DFR in the *EPI‐group* was 124 days (range 22–2188 days) and median survival time (MST) was 217 days (range 29–2188 days); the median DFR in the *DOX‐Group* was 185 days (range 36–1488 days) and MST was 263 days (range 36–1488 days). The difference between groups was not statistically significant and none of the variables, including stage, substage and immunophenotype, influenced DFR and OST.

In conclusion, DOX may be associated with a higher incidence of GI AEs compared to EPI, although there was no significant difference in the occurrence of severe AEs (VCOG events ≥ 3) between the two groups. No significant difference in DFR or OST was detected, possibly suggesting a similar efficacy when one of these AC is used within a multi‐agent protocol.

**Disclosures**

Dr. Valenti and Dr. Treggiari are consultants for WizzVet, Dr. Finotello is a consultant for Vallonea Lab.

## ESVONC‐O‐10

### ESVONC—European Society of Veterinary Oncology

#### Optimization of radioactive iodine uptake in canine thyroid carcinomas using recombinant human thyroid stimulating hormone

##### S Scheemaeker, K Peremans, E Vandermeulen, L Duchateau, T Roggeman, S Daminet

###### University of Ghent, Merelbeke, Belgium

Only around 50% of the dog population with thyroid carcinoma (TC) are good candidates for radioactive iodine (^131^I) therapy. Further, high doses of ^131^I are required to effectively treat canine TCs. Hence, optimization of ^131^I therapy in dogs is warranted. In people, recombinant human thyroid stimulating hormone (rhTSH) increases radioactive iodine uptake (RAIU) in differentiated TCs optimizing the efficacy of ^131^I treatment. A previous study could not clearly demonstrate such an effect of rhTSH on RAIU in canine TCs. Therefore, the influence of a revised rhTSH protocol on tumour RAIU in dogs with TC was evaluated in a prospective cross‐over study design.

Nine dogs, diagnosed with TC based on cytology and medical imaging, were included. One dog was excluded because histopathology did not confirm TC. Thyroid tumours were unilateral in 6/9 dogs and bilateral in 3/9 dogs, whereas 6/9, 1/9 and 2/9 dogs were euthyroid, hypothyroid and hyperthyroid, respectively. The study lasted two weeks with one week wash‐out in between. Treatment order was randomly assigned to each dog. Treatment consisted of 250 μg of rhTSH administered IM 24h and 12h prior to IV administration of 37 MBq ^123^I, followed by an 8h and 24h static thyroid scintigraphy scan. Blood samples were taken before and 6h, 12h, 24h and 48h after rhTSH administration to determine serum total thyroxine (TT4) and TSH concentrations. The procedures were identical if no treatment was administered, except for blood collection. The 8h‐ and 24h‐RAIU, with and without rhTSH, were calculated and compared using a mixed model with dog as random effects, and period and treatment as fixed effects.

Overall, rhTSH administration resulted in a significant increase of tumour 24h‐RAIU (*P* = 0.03) but failed to cause a significant increase of tumour 8h‐RAIU (*P* = 0.052). Recombinant human TSH also caused a significant change of serum TT4 and TSH concentrations over time (*P* < 0.001). The area under the curve (AUC) of TSH was negatively correlated with the body weight of the dogs (*r*= −0.704, *P* = 0.01). No significant correlation existed between the AUC of TT4 and TSH, and tumors’ 8 h‐ and 24 h‐RAIU (*P* > 0.30).

In conclusion, IM administration of 250 μg rhTSH 24 and 12 h prior to radioactive iodine causes a significant increase in tumour 24h‐RAIU. The latter could lead to ^131^I treatment optimization in canine TCs as is the case in human differentiated TC.

**Disclosures**

No disclosures to report

**Source of Funding**

ECVIM Clinical Studies Fund; Bijzonder Onderzoeks Fonds (BOF) UGent; MEDVET laboratorium

**ECVIM CSF Funding**

Yes

## ESVONC‐O‐11

### ESVONC—European Society of Veterinary Oncology

#### Serum lactate dehydrogenase acts as a potential prognostic biomarker of canine appendicular osteosarcoma

##### D Guerra ^1^, L Marconato ^1^, S Sabattini ^1^, E Faroni ^1^, C Chalfon ^2^, A Renzi ^1^, R Zaccone ^1^, S Zanardi ^1^, I Maga ^1^

###### ^1^ University of Bologna, Bologna, Italy; ^2^ Centro Veterinario Torinese, Turin, Italy

Dogs with appendicular osteosarcoma undergoing limb amputation and adjuvant chemotherapy have a median survival time of 10–14 months; unfortunately, the greatest majority of patients faces the development of metastatic disease. Therefore, identifying valuable prognostic factors is important for anticipating outcome. High serum alkaline phosphatase (ALP) and proximal humerus location are well‐documented negative prognostic factors in dogs with osteosarcoma. In human patients with osteosarcoma, increased serum lactate dehydrogenase (LDH) is strongly associated with poor prognosis.

The aim of this retrospective study was to investigate whether elevated serum LDH at diagnosis resulted in shorter time to progression (TTP) and overall survival (OS) in dogs with appendicular osteosarcoma undergoing limb amputation and adjuvant chemotherapy.

Medical records were retrospectively searched for dogs with completely staged appendicular osteosarcoma, that had been tested for serum LDH at diagnosis and underwent amputation and adjuvant carboplatin chemotherapy. Possible prognostic factors (i.e., signalment, tumor anatomic location, ALP, lymph node metastasis, distant metastasis, presence of pathologic fracture, histologic subtype, histologic grade) and elevated serum LDH activity were tested for their possible influence on TTP and OS.

A total of 30 dogs were included in the analysis. At diagnosis, 15 (50%) had high LDH and 15 had normal LDH. The two groups were well balanced in terms of the aforementioned prognostic factors.

On univariable analysis, high LDH level and high ALP level were significantly associated with an increased risk of tumor progression and death. On multivariable analysis, elevated ALP was significantly associated with an increased risk of tumor progression (HR: 6.21; 95% CI: 1.42–27.17; *P* = 0.015), while high LDH activity was associated with an increased risk of death (HR: 4.15; 95% CI: 1.23–14.09; *P* = 0.022).

Median TTP and OS of dogs with normal LDH (300 days, 95%CI: 105–495, and 387 days, 95% CI: 172–602, respectively) were significantly longer than those having high LDH (134 days, 95% CI: 108–160, *P* = 0.038, and 169 days, 95% CI: 73–265, *P* = 0.010, respectively). One‐year survival rates were 50% for dogs with normal LDH and 9.1% for those with increased LDH.

In the current study, serum LDH activity at diagnosis was significantly associated with OS in dogs with appendicular osteosarcoma. In view of its low cost and reproducibility, LDH could be introduced as a prognostic biomarker in clinical practice. These data need to be confirmed in prospective studies.

**Disclosures**

No disclosures to report

## ESVONC‐O‐12

### ESVONC—European Society of Veterinary Oncology

#### Investigation and description of circulating MicroRNAs in healthy and T‐cell lymphoma‐bearing dogs: a prospective, two‐arm study

##### C Capuano ^1^, V Moccia ^2^, S Ferraresso ^2^, A Molinari ^2^, F Torrigiani ^2^, V Zappulli ^2^, C Leo ^1^

###### ^1^ AniCura Istituto Veterinario Novara, Granozzo con Monticello, Italy; ^2^ University of Padua, Padua, Italy

MicroRNAs (miRNAs) are a family of highly conserved, small, non‐coding RNAs that play an important role in many biological processes, including tumorigenesis. Among others, they have been largely investigated as tumor biomarkers in human lymphomas. Recent studies have described their potential role as biomarkers also in canine B‐cell lymphoma.

This study aims to identify expression patterns of specific circulating miRNAs in canine T‐cell lymphoma, specifically miRNAs associated with circulating extracellular vesicles (EVs) were studied.

Canine patients were enrolled and divided into healthy control group (CG, *n* = 8) and lymphoma‐bearing group (LG, *n* = 8). Inclusion criteria for the CG comprised no abnormalities at clinical examination, complete blood count, serum biochemistry, and lymph node cytology. Inclusion criteria for the LG included newly diagnosed T cell lymphoma, free of concurrent disease and naïve to corticosteroid therapy.

Leftover plasma samples of both groups were collected. EVs were isolated with ultracentrifugation and circulating EV‐associated miRNAs extracted. Quantitative Real‐Time polymerase chain reaction (qPCR) assays were performed to quantify the expression of specific canine target miRNAs. Cycle threshold (CT) values were analyzed via data analysis web portal at http://www.qiagen.com/geneglobe using the ∆∆CT method, and normalization was based on the NormFinder method. The Fold Change (FC) was then calculated using 2^ (‐∆∆CT) formula and *P* values were calculated based on a Student's *t*‐test, with *P <* 0.05 were considered significant.

Nine EV‐associated miRNAs were found significantly differently expressed between CG and LG, specifically hsa‐miR‐103a‐3p, cfa‐miR‐191, hsa‐miR‐192‐5p, hsa‐miR‐222‐3p, rno‐miR‐223‐3p, bta‐miR‐27a‐3p, hsa‐miR‐30b‐5p, cfa‐miR‐30d, hsa‐miR‐378a‐3p. Rno‐miR‐223‐3p expression has been reported in human hematopoietic neoplasia too. One additional miRNA was found marginally significant (has‐miR‐130a‐3p) (*P* = 0.0506).

T‐cell canine lymphomas express a different pattern of specific circulating miRNAs compared to healthy dogs from the circulating EVs. These miRNAs could be evaluated in the future as possible circulating biomarkers for helping in early detection of T‐cell lymphoma in canine patients

**Disclosures**

This study has been funded by the AniCura scientific grant (2020–2021) which has been used to cover the laboratory costs. None of the authors has been paid by using this fund. Drs. Capuano and Leo are paid clinicians for Mars AniCura Inc.

**Source of Funding**

AniCura scientific grant (10.000 €)

## ISCAID‐O‐1

### ISCAID—International Society for Companion Animal Infectious Diseases

#### Update on the distribution and seasonal occurrence of vector‐borne diseases in dogs in France

##### K Museux^1^, C Boucraut^2^

###### ^1^Cerba Vet Massy, France; ^2^Scanelis Colomiers, France

France is already known for being endemic for several canine vector‐borne diseases, including zoonotic ones. However, recent research showed that the distribution and seasonality of vector‐borne diseases are changing. The aim of this retrospective study was to evaluate the clinical occurrence in order to update the distribution of these diseases in France.

Between January 1, 2021 and December 31, 2022, biological samples (blood, synovia, CSF, tissue) of dogs suspected of vector‐borne diseases were submitted for a total of 9462 PCRs and 8541 serologies to the laboratory. All the positive cases were implemented in an online and public map.

*Babesia* infections were diagnosed by PCR in 547/3794 blood samples. In 78 samples, the *Babesia* species could be identified by Amplified Fragment Length Polymorphism. 70 were positive for *Babesia canis*, 5 for *Babesia vogeli*, 2 for *Babesia vulpes* and 1 for *Babesia gibsoni*. The seasonal distribution showed a year round occurrence, with the highest occurrence rates from March to May and in October. *Ehrlichia* sp. was detected in 25/3423 samples by PCR. Serology was positive in 115/1766 samples. *Anaplasma* sp. was detected in 17/ 2087 samples by PCR while 41/1081 samples were positive at serology. *Mycoplasma* PCR was positive in 11/879 samples for *Mycoplasma haematoparvum* and 4/879 samples for *Mycoplasma haemocanis. Hepatozoon* sp. was detected by PCR in 3/879 samples. Neither *Rickettsia* sp., *Neorickettsia* sp., *Bartonella* sp. nor *Borrelia burgdorferi sl* (0/879) were detected in any of the samples by PCR. However, serology (ELISA) was positive to *Borrelia burgdorferi sl* in 102/1335 cases, with 10 out of 111 immunoblots positive for Variable Lipoprotein Surface‐Exposed protein (VlsE). Two out of 18 samples were positive for *Dirofilaria immitis* and 5 for *Dirofilaria repens* by PCR, 13 out of 198 by a filtration test for microfilaria and 43 out of 355 cases for *Dirofilaria immitis* antigen. *Leishmania* PCR was positive in 146 cases out of 1898. Serology was positive in 1312 out of 3893 samples for *Leishmania infantum*.

This study is the first study to combine PCR and serology results for a countrywide survey. This dataset underlines the rise of vector‐borne diseases in new territories as well as occurrence all around the year due to climate changes and travelling. This data may increase the awareness of veterinarians about vector‐borne disease agents when investigating a travel history case in order to better select the appropriate diagnostic and prophylactic measures.

**Disclosures**

We declare that the two authors (Kristina Museux and Corine Boucraut) are employees of Cerba Vet, which performs diagnostic testing on a commercial basis.

**Source of Funding**

No funding was received for this retrospective study

## ISCAID‐O‐2

### ISCAID—International Society for Companion Animal Infectious Diseases

#### Seropositivity to the louping ill flavivirus in dogs in the UK

##### I. Elgueta^1^, K. Allen^2^, T. Liatis^3^, V. Gonzalo‐Nadal^4^, E. Laming^2^, M.P. Dagleish^4^, P.M. Jamieson^5^, G. Innocent^6^, M. Rocchi^2^

###### ^1^Internal Medicine Service, Vets Now Referral Hospital, Glasgow, UK; ^2^Moredun Research Institute, Edinburgh, UK; ^3^Royal Veterinary College, London, UK; ^4^University of Glasgow, Glasgow, UK; ^5^Vets Now Referral Hospital, Glasgow, UK; ^6^Biomathematics and Statistics Scotland, Edinburgh, UK

Neurotropic flaviviruses, including louping ill virus (LIV) and tick‐borne encephalitis virus (TBEV), cause disease with neurological complications in various species. Both LIV and TBEV are transmitted primarily by *Ixodes ricinus* or *Dermacentor* spp. ticks, but while TBEV is endemic to central Europe, where infection causes encephalitis in several species including dogs and humans, the closely related LIV is endemic to the UK and affects primarily sheep and grouse. LIV has been reported to cause clinical meningoencephalitis in only three dogs in the last 50 years, most recently in 2018, however underreporting is suspected.

The aim was to investigate the prevalence of exposure of dogs in the UK to LIV. Surplus serum or plasma from 220 dogs sampled for clinical purposes by Neurology or Internal Medicine Referral Services or General Practice Clinics between September 2021 and October 2022 was analysed with informed owner consent. An owner questionnaire gathered information on the dogs’ lifestyles and geographical environments, known tick exposure, acaricide use and travel history. Serological analysis for LIV was performed by hemagglutination inhibition for total immunoglobulins. A titre of greater than 1:10 was considered positive and was the outcome of interest in statistical univariate logistic regression analysis.

Eighteen samples were invalid due to non‐specific inhibition of haemagglutination. A further 22 dogs had equivocal titres of 1:10, which could represent historical LIV exposure or an unexplained reactivity in the assay observed previously in occasional naive animals. Nine of 202 dogs (4.5%) had a positive titre. In dogs without neurological signs this was 4 of 146 (2.6%), whereas in dogs under investigation for neurological signs (2 seizures, one pelvic limb paraparesis, one acute progressive painful T3‐L3 myelopathy, one blindness) this was 5 of 46 (10.9%). No seropositive dog had known tick exposure in the previous 12 months, and only one had received acaricide in the owner's recollection. None had travelled outside the UK.

No signalment or lifestyle covariate was statistically significant at the 5% level. However, low numbers of seropositive animals meant the statistical power to detect differences truly present was low (high Type II error probability). Acaricide use approached significance (*P* = 0.065) for being protective against LIV seropositivity.

This study suggests that exposure of dogs to LIV in the UK, as denoted by an immunological response, is more common than previously thought, and LIV should be considered as a potential diagnosis in dogs presenting with unexplained acute or subacute progressive neurological signs.

**Disclosures**

M. P. Dagleish: Is in receipt of funding from the University of Glasgow Jerry and Joanne Haigh Fund for work unrelated to LIV. P. M. Jamieson: Receives salary for External Examiner for the British Small Animal Veterinary Association. M. Rocchi: Is in receipt of research funding from the European Union, Defra and The Scottish Government for work unrelated to LIV in dogs. M. Rocchi, K. Allen, E Laming: The Moredun Research Institute runs a laboratory service which includes LIV testing for Scottish Government surveillance purposes and for commercial clients. I. Elgueta Gil, T. Liatis, V. Gonzalo‐Nadal, G. Innocent: no disclosures

**Source of Funding**

This study was funded by a Dogs Trust grant: “Risk factors affecting the exposure to louping ill in dogs”. The Authors acknowledge the support of the Clinical Investigation Centre of the Royal Veterinary College.

## ISCAID‐O‐3

### ISCAID—International Society for Companion Animal Infectious Diseases

#### Comparison of prognostic factors in feline panleukopenia in juvenile and adult cats

##### A Strobl, I Burgener, S Schery, M Leschnik

###### University of Veterinary Medicine Vienna, Vienna, Austria

Feline panleukopenia (FPL) is a highly contagious and often fatal disease, with diarrhoea and vomiting being the leading symptoms. Kittens are most affected, but cats of all ages can suffer from the disease. Prognostic factors for survival have been evaluated but no studies have investigated differences in prognostic factors in cats of different ages.In this retrospective study we compared serum total protein, serum albumin, total leucocyte and neutrophil count, age, and the lowest and highest recorded body temperature between juvenile (<1 year) and adult (≥1 year) cats with FPL. Correlations between alterations in the parameters and survival probability were calculated by binary logistic regression analysis. Mann‐Whitney U test was performed to compare the survival probabilities between both groups. *X* ^2^ test was used to examine the association between both ages and survival.The study included 266 cats, whereof 225 cats were allocated to the juvenile group and 41 cats to the adult group. Overall lethality in our population was 35.7%. Results showed survival probability dropping below 50% if albumin fell below 1.96 g/dl in the adults and 1.84 g/dl in the juvenile cats. No significant difference was found between both groups (*P* = 0.428). Total protein values below 4.56 g/dl in the juvenile cats and 5.67 g/dl in the adult cats were associated with a lethality rate of more than 50%—which represented a significant difference between both groups (*P* = 0.012). Leucocyte and neutrophil count were not associated with a survival probability below 50%. Results were similar between both groups (leucocytes: *P* = 0.544; neutrophils: *P* = 0.913). Within the juvenile cats, survival increased significantly with higher age (*P* = 0.025), while there was no association between age and survival in the adult cats (*P* = 0.832). Further, no significant difference was found among both age groups and survival (*P* = 0.63). Interestingly, we found significant differences when evaluating the lowest recorded body temperature. In juvenile cats the risk of death increased above 50% if temperature fell below 36.1°C, while in adult cats this happened already at 36.8°C (*P* = 0.033). In contrast, an elevated body temperature was not associated with lethality, with no differences between both groups (*P* = 0.985).

This study showed that total protein and lowest body temperature as prognostic factors are significantly different when looking at survival probability in juvenile and adult cats. Furthermore, it is crucial to monitor vital signs for early recognition and prevention of hypothermia as well as secure continuous protein intake to prevent hypoproteinaemia—independent of the cat's age.

**Disclosures**

No disclosures to report

## ISCAID‐O‐4

### ISCAID—International Society for Companion Animal Infectious Diseases

#### Comparison of antibody response after feline panleukopenia virus vaccination in kittens with and without gastrointestinal parasitic infection

##### A Weidinger^1^, M Bergmann^1^, D Barutzki^2^, U Truyen^3^, Y Zablotski^1^, K Hartmann^1^

###### ^1^Clinic of Small Animal Medicine, LMU Munich, Munich, Germany; ^2^Veterinary Laboratory Freiburg, Freiburg in Breisgau, Germany; ^3^Institute of Animal Hygiene and Veterinary Public Health, Veterinary Faculty, Leipzig, Germany

Deworming before vaccination is generally recommended, but so far, no study investigated the influence of intestinal parasitic infection on the immune response in cats. The aim of the study was to compare the antibody response after feline panleukopenia virus (FPV) vaccination in kittens with and without intestinal parasites.

In this prospective study, 74 healthy kittens were included. Of these, 17 had intestinal parasites (12/17 *Toxocara cati*, 6/17 *Cystoisospora felis*, 1/17 *Capillaria* spp.). Both, cats with and without (*n* = 57) parasites received 2 primary kitten vaccinations with modified live FPV starting at the age of 8–12 weeks and in a 4‐week interval. Anti‐FPV antibodies were determined on week 0 (before vaccination) and week 8 (4 weeks after 2nd vaccination) by haemagglutination inhibition (HI). A titer increase ≥4‐fold titer (week 0 to week 8) was defined as vaccination response. Comparison of the immune response of the two groups was performed using Fisher's exact test.

Pre‐vaccination anti‐FPV antibodies were present in 23.5% (4/17) of kittens with intestinal parasites and in 42.1% (24/57) without parasites. A ≥4‐fold titer increase was seen in 76.5% (13/17) of infected kittens compared to 56.1% (32/57) of non‐infected kittens (56.1%). There was neither a significant difference in pre‐vaccination antibody titers (*P* = 0.255), nor in vaccination response (*P* = 0.164) between kittens with and without parasites.

Results indicate that asymptomatic intestinal infections with endoparasites does not impair the immune response to kitten vaccination series. Parasitic infection (at least with *Toxocara cati*, *Cystoisospora felis*, *Capillaria* spp.) is therefore not a reason to postpone important vaccinations.

**Disclosures**

Research was partially funded by Boehringer Ingelheim. Anna‐Karina Weidinger is in a residency program (sponsored by Virbac). Prof. Dr. Katrin Hartmann is member of the ABCD (sponsored by Boehringer Ingelheim). Katrin Hartmann has given talks for MSD, Boehringer Ingelheim, IDEXX; Michèle Bergmann for Hill's and Boehringer. Katrin Hartmann and Michèle Bergmann participated in research funded by or using products from MSD, Boehringer, Virbac, Zoetis, Megacor, Biogal, Scil. Boehringer and Virbac played no role in the collection and interpretation of data. There is no commercial conflict of interest of the authors as the information generated here is solely for scientific dissemination.

## ISCAID‐O‐5

### ISCAID—International Society for Companion Animal Infectious Diseases

#### Performance of a molecular diagnostic as compared to routine centrifugal‐flotation for fecal gastrointestinal parasite identification

##### D Evason, M Leutenegger, M Wilcox, Rochani, Meeks, D Mitchell, M Andrews

###### Antech Diagnostics, part of Mars PetCare, Fountain Valley, USA

Routine gastrointestinal (GI) parasite screening in veterinary medicine has long incorporated zinc sulfate centrifugal flotation (ZCF) for ova and parasite (O&P) identification as the primary methodology. While ZCF remains standard in many veterinary hospitals, this test has known limitations in sensitivity, high potential for subjectivity, and only identifies what can be visualized microscopically. Other ZCF limitations include the inability to detect potential zoonotic *Giardia duodenalis* assemblages and hookworm benzimidazole (BZ) treatment resistance markers. The aim of this study was to evaluate the performance of a broad (20 GI parasites), rapid, molecular diagnostic (qPCR) panel for GI parasite screening as compared to routine ZCF (as performed at a reference laboratory).

A selection of dog and cat fecal samples (*n* = 931) submitted from the Northeast USA (US) in November 2022 for ZCF, were subsequently evaluated by qPCR following retention release. Eight parasites were selected for evaluation, and frequency of detection was calculated for both methods. Sensitivity and specificity of detection was determined for each test (qPCR and ZCF) and then compared.

Overall, qPCR detected 2.6 times more parasites (*n* = 977 vs. 372) and 4.1 times more coinfections (*n* = 374 vs. 90), as compared to ZCF. The *Ancylostoma* spp. detection frequency was 3.3% (*n* = 31) of fecal samples by qPCR, as compared to 2.4% (*n* = 22) with ZCF, with a sensitivity of 95.5% (qPCR) and 67.7% (ZCF), respectively. Five *A. caninum* detected samples were F167Y marker detected (16.1%), indicating BZ resistance. *Giardia duodenalis* qPCR detection frequency was 26.1% (*n* = 243) with 95.2% sensitivity, as compared to ZCF frequency 13.4% (*n* = 125) and 49% sensitivity. Zoonotic potential *Giardia* assemblages were detected in 9.1% of samples (*n* = 22). *Toxocara* spp. was detected by qPCR with a frequency of 32.2% (*n* = 300), with a sensitivity of 95.1%. Four of the eight parasites were detected by qPCR, *Tritrichomonas blagburni* (*n* = 16), *Toxoplasma gondii* (*n* = 1), *Cryptosporidium canis* (*n* = 71), and *C. felis* (*n* = 18), and unidentified by ZCF.

These data describe the utility and sensitivity of performance of a rapid qPCR method for routine GI parasite screening in veterinary practice. Additionally, this fecal qPCR panel offers benefits beyond ZCF, with information on *Giardia* assemblages with zoonotic potential and hookworm treatment (BZ) resistance. This study highlights the importance of parasite detection molecular diagnostic advancement allowing improved GI parasite identification that informs clinical decision‐making and incorporates One Health and antimicrobial stewardship.

**Disclosures**

All authors are employed by Antech Diagnostics, Inc., a part of the Mars PetCare family of companies

**Source of Funding**

Antech Diagnostics, Inc.

## ISCAID‐O‐7

### ISCAID—International Society for Companion Animal Infectious Diseases

#### Leishmania infantum‐specific production of IL‐2 in stimulated blood in dogs with different states of infection

##### Dr. Murillo, Ms. Jiménez, Ms. Del Collado, Dr. Solano

###### Universitat Autònoma de Barcelona Bellaterra, Cerdanyola del Vallès, Spain

Canine leishmaniosis (CanL) caused by *Leishmania infantum* (Li) is a frequent sandfly‐borne disease in endemic areas. The host's immune system plays an important role in determining the disease's severity. Th1‐like immune response usually determines a resistant profile, leading to subclinical infection or mild to moderate disease, involving cytokines such as IFN‐γ, IL‐2, and TNF‐ α. On the contrary, the predominance of Th2‐like immune response, with IL‐10, IL‐4, IL‐5, and TGF‐ β cytokines, leads to more severe illness. Quantification of parasite specific IFN‐γ production in canine whole blood assay (WBA) is commonly used to evaluate immune response in CanL. However, scarce information is available regarding *Li* specific IL‐2 production in WBA in different states of canine infection. The present study aimed to assess the *Li* specific production of IL‐2 in dogs in different states of infection and correlate these findings with humoral immune response. Sixty‐one dogs were recruited based on physical examination, blood work, cytology of lesions, antibody levels against *Li* ELISA and IFN‐γ concentration against *Li* soluble antigen (LSA) in a WBA. Dogs were classified as healthy seronegative and non‐IFN‐γ producers (group 1, *n* = 16), healthy seronegative or seropositive and IFN‐γ producers (group 2, *n* = 15), sick dogs with leishmaniosis and IFN‐γ producers, belonging to LeishVet I and II stages (group 3, *n* = 15) and sick dogs with leishmaniosis and non‐IFN‐γ producers, LeishVet stages II and III (group 4, *n* = 15). WBA were stimulated with medium alone, LSA and concanavalin‐A (ConA). Supernatants were harvested for measurement of canine IL‐2 concentration by ELISA. Differences in IL‐2 concentrations in LSA‐stimulated WBA were observed when comparing groups 1 and 2 (*P* < 0.005), 1 and 3 (*P* < 0.001), 2 and 4 (*P* < 0.005), 3 and 4 (*P* < 0.005), being highest in groups 2 and 3. IL‐2 concentrations were higher in group 2 (median 245.0, IQR 245.1 pg/mL) when compared with group 4 (median 116.2, IQR 97.2 pg/mL) (*P* < 0.05) in ConA‐stimulated WBA. Parasite specific IL‐2 concentrations were positively correlated with IFN‐γ concentrations (*r* = 0.7, *P* < 0.001). No correlation was found with antibody levels. Lower percentages of IL‐2 producers (66.7%) were encountered when compared with all dogs IFN‐γ producers (groups 2–3, 100%) (*P* < 0.005). In conclusion, parasite specific IL‐2 production showed a similar pattern of IFN‐γ immune response in all groups studied. However, IFN‐γ appears to be a best marker of subclinical infection and mild to moderate disease in CanL.

**Disclosures**

No disclosures to report

**Source of Funding**

Spanish Ministry grant. Ministerio de Ciencia e Innovación: PID2020‐113267RB‐I00

## ISCAID‐O‐9

### ISCAID—International Society for Companion Animal Infectious Diseases

#### Severe babesiosis in recently splenectomised dogs due to suspected or confirmed Babesia vulpes infection

##### Y Fernández^1^, MC Luna^2^, R Checa^3^, G Miró^3^, A Rodríguez^4^, M Suárez^5^

###### ^1^AniCura Imavet Referencia Veterinaria, Santiago de Compostela, Spain; ^2^AniCura Navia Clínica Veterinaria, Vigo, Spain; ^3^Universidad Complutense, Madrid, Spain; ^4^AniCura Vetsia Hospital Veterinario, Madrid, Spain; ^5^Rof Codina Veterinary Teaching Hospital, Lugo, Spain

In both humans and dogs, splenectomy is a risk factor for increased disease severity in patients with babesiosis.

The aim of this study was to describe the clinical presentation, treatment and outcome of recently splenectomised dogs with suspected or confirmed *Babesia vulpes* infection.

Retrospective case series of dogs with suspected or confirmed *Babesia vulpes* infection <2 months following splenectomy. Diagnosis of babesiosis was based on the direct identification of intraerythrocytic parasites consistent with small *Babesia* spp. in dogs that lived in an endemic region for *Babesia vulpes* (Galicia, Spain).

Fourteen dogs were included. Five dogs had no clinical signs, the remaining dogs presented with lethargy and weight loss (4 dogs), abdominal pain (2 dogs), gastrointestinal signs, lameness and PU/PD (1 dog each). Prior to surgery, laboratory abnormalities included concurrent (11 dogs) or isolated anaemia and thrombocytopenia (1 dog each), hyperglobulinemia (3 dogs) and azotaemia (2 dogs). *Babesia* spp. nested PCR was negative in 4 dogs. Reasons for splenectomy included the presence of splenic nodules (5/14), a heterogenous mass that distorted the splenic capsule (4/14), severe splenomegaly with mottled appearance (3/14) and splenic torsion (2/14). Splenic histopathology identified benign changes in all cases. Median time from splenectomy to diagnosis of babesiosis was 30 days (range: 6–50 days). All dogs presented with a progressive anaemia, thrombocytopenia and a high parasitological burden on blood smears. *Babesia vulpes* infection was confirmed by nested PCR in 10/14 dogs. All dogs received antiprotozoal treatment, 12/14 dogs received ≥1 packed red blood cell transfusions (range: 1–6) and 11/14 dogs received glucocorticoids concurrently. Antiprotozoal treatment included one (8 dogs), two (4 dogs), three (1 dog) and six (1 dog) courses of atovaquone or buparvaquone and azithromycin ± clindamycin from 1 to 46 days (median: 10 days). Seven dogs received at least one dose of imidocarb. Three dogs also received a course of metronidazole, clindamycin and doxycycline from 3 weeks to 4 months and achieved a clinical cure. Eight dogs died due to progressive/refractory disease a median of 45 days (range: 1–510 days) after diagnosis. Five dogs are still alive at the time of writing (median follow‐up: 114 days; range: 60–729 days) and one dog died 19 months after splenectomy due to an unrelated cause.

Dogs diagnosed with *Babesia vulpes* infection following splenectomy appear to develop a severe disease with a greater than 50% mortality rate. This risk merits awareness, particularly in endemic areas of this piroplasm.

**Disclosures**

No disclosures to report

## ISCAID‐O‐10

### ISCAID—International Society for Companion Animal Infectious Diseases

#### Molecular detection of Babesia spp. in community and privately‐owned cats in Hong Kong

##### A Almendros^1^, YR Choi^1^, T Leung^1^, WY Tam^1^, DH Muguiro^2^, FM Woodhouse^3^, JJ Gray^3^, JA Beatty^1^, VR Barrs^1^

###### ^1^Jockey Club College of Veterinary Medicine and Life Sciences, City University of Hong Kong, Kowloon Tong, Hong Kong SAR, China; ^2^Veterinary Diagnostic Laboratory, City University of Hong Kong, Kowloon Tong, Hong Kong SAR, China; ^3^The Society for the Prevention of Cruelty to Animals, Wan Chai, Hong Kong SAR, China

Domestic cats are susceptible to infection with at least 11 *Babesia* spp. A few species are associated with babesiosis, particularly in South Africa, but most feline *Babesia* infections are subclinical. In Hong Kong, where dogs are commonly infected with *B. gibsoni*, a single infection in a cat with novel species *B. hongkongensis* is reported.

The aim of this study was to investigate the frequency of detection of *Babesia* spp. in cats in Hong Kong. Residual whole‐blood DNA from free‐roaming healthy community‐owned cats (*n* = 239), privately‐owned cats undergoing diagnostic investigations (*n* = 112) collected between January 2021 and March 2023 was used. Signalment and clinical data, where available, were recorded. DNA samples testing positive for housekeeping gene GAPDH on cPCR were tested for *Babesia* DNA using a pan‐*Babesia* mitochondrial *Cytochrome B* cPCR, and a *B. hongkongensis*‐specific 18S rRNA cPCR. Positive samples were confirmed by sequencing and comparative sequence analysis using the GenBank nucleotide database.

*B. hongkongensis* was detected in 4/239 (1.67%) community cats, and 0/112 privately‐owned cats. *B gibsoni* was detected in 0/239 community cats and 1/112 (0.89%) owned cats. The *B. gibsoni*‐infected cat had with signs consistent with babesiosis (anaemia, thrombocytopenia), although parasitaemia was subsequently cleared while signs persisted.

Cats in Hong Kong can be infected with *B. hongkongensis* or *B. gibsoni*, albeit at low frequency. Whether or not *B. gibsoni* contributed to clinical signs in the infected cat is unclear. Molecular surveillance of cats with and without risk factors for *Babesia* infection in the region is warranted.

**Disclosures**

No disclosures to report

**Source of Funding**

The study was supported by grants from City University, HK to VRB (Project No 9380113) and AA (No. 9610596).

## ISCAID‐O‐11

### ISCAID—International Society for Companion Animal Infectious Diseases

#### Detection of pathogenic Leptospira spp. serogroups in Europe between 2017 and 2020 applying a gene‐based molecular approach

##### J Wenderlein^1^, T Zitzl^1^, D Dufay‐Simon^2^, N Cachet^2^, M Le Guyader^3^, JP Tronel^2^, L Cupillard^2^, RK Straubinger^1^

###### ^1^LMU Munich, Institute for Infectious Diseases and Zoonoses, München, Germany; ^2^Boehringer Ingelheim Animal Health, R&D Centre Lyon Porte des Alpes, Saint‐Priest, France; ^3^Pôle d'analyse, VetAgroSup, Université de Lyon, Marcy L’étoile, France

Leptospirosis is a neglected but re‐emerging worldwide zoonotic disease. Climate change and urbanization elevate the risk of acquiring *Leptospira* (*L*.) spp. A wide spectrum of mammals, including dogs, can acquire leptospirosis resulting in the shedding of leptospiral organisms with urine. Current canine vaccines in Europe contain from two to four serogroups. The lack of cross‐protection between leptospiral serogroups makes the continuous evaluation of leptospiral epidemiology necessary to assess the suitability of current vaccines and identify shortcomings to protect both dogs and humans.

Dogs’ blood and urine samples sent to a commercial laboratory with suspected *L*. spp. infection and tested positive with the *lipL32*‐PCR were collected when residual DNA was available (*n* = 239). Residual DNA was further analyzed using a novel molecular serogroup typing approach. First, a 16S rRNA endpoint PCR was conducted, followed by 16S rRNA gene amplicon sequencing to identify the leptospiral DNA and the respective genospecies (either *L. interrogans*, *L. kirschneri*, or *L. borgpetersenii*). According to the species identified, a PCR protocol with serogroup‐specific primers was conducted. Tests for *L*. interrogans‐serogroups Icterohaemorrhagiae (ICT), Australis (AUS), Pomona (POM), Canicola (CAN), Autumnalis (AUT), and Pyrogenes (PYR) were performed consecutively; for *L. kirschneri*‐serogroups ICT, POM, Grippotyphosa (GRI), and AUT, and for *L. borgpetersenii*‐serogroups Sejroe (SEJ). If neither of the PCRs displayed a positive signal, the sample was classified as unknown.

The newly developed PCR was able to type the serogroup of 81% of samples containing leptospiral DNA. As only the most prevalent canine serogroups throughout Europe were tested for, samples classified as unknown might originate from serogroups not tested. Nevertheless, this limitation is common with MAT, MLST, and VNTR. In comparison to MAT, a test widely used in veterinary studies, this novel approach *via* PCR allows higher standardization and compatibility. The most prevalent *L*. spp.‐serogroup identified in Europe was ICT (53%), followed by serogroups detected in some countries but not all like AUS (13%). The less prevalent *L*. spp.‐serogroups detected in some countries were POM (5%), AUT (4%), SEJ (2%), CAN (1%), GRI (1%), and PYR (1%). In the past, the most seroprevalent groups were ICT, GRI, AUS, and CAN. As rodents show a high seroprevalence for ICT, AUS, and GRI, a continuous vaccination approach is needed, while the inclusion of the host‐adapted serogroup CAN is recommended. This work shows that current L4 vaccines are relevant and should confer a high efficacy profile at least against ICT and AUS serogroups.

**Disclosures**

No disclosures to report

## ISCAID‐O‐12

### ISCAID—International Society for Companion Animal Infectious Diseases

#### Serological evidence of exposure to Leptospira in dogs in Sydney, New South Wales, Australia

##### C Griebsch, N Kirkwood, M Ward, J Norris

###### Sydney School of Veterinary Science, The University of Sydney, Sydney, Australia

In 2019, highly fatal canine leptospirosis emerged in Sydney, New South Wales (NSW), Australia. Based on results of microscopic agglutination testing (MAT), serovar Copenhageni appeared to be the causative serovar in most clinical cases. Prior to this, there had been no cases reported since 1976. In a serosurvey of healthy dogs in Australian shelters in 2004, seroprevalence of 2.4% was estimated in 431 NSW dogs and Copenhageni was the most prevalent serovar.

The aim of the study was to determine the prevalence of *Leptospira* exposure and the associated serovars in healthy Sydney dogs, previously unvaccinated against *Leptospira*.

Serum samples from 411 healthy dogs in leptospirosis hotspots and in neighboring suburbs were collected prior to vaccination. Microscopic agglutination testing (MAT) for 23 serovars was performed at the WHO Leptospirosis Reference Laboratory in Queensland, Australia.

The overall seroprevalence was 4.1% (17/411) and titres were low (1/50 to 1/200). Eleven dogs were from leptospirosis hotspots with previously confirmed cases. Eight dogs were known to hunt rodents. One dog had been in contact with a leptospirosis positive dog one year prior. Serovar Topaz was the the most prevalent serovar (*n* = 5) followed by serovars Australis (*n* = 4), Copenhageni (*n* = 4), Djasiman (*n* = 2), Cynopteri (*n* = 1), Javanica (*n* = 1), Medanensis (*n* = 1) and Pomona (*n* = 1).

In conclusion, serological evidence of exposure to Leptospira in dogs in Sydney is low however has increased since 2004. Positive titres to serovars not previously reported to cause disease in dogs could be due to low virulence of those serovars or cross‐reactivity with other serovars. Low seroprevalence with emergence of canine leptospirosis suggests a high virulence of infecting serovars.

**Disclosures**

The authors have the following disclosures related to their abstract: Employee/salary: N/A Grants/research: The study has been funded by MSD Animal Health and philantropic funding from the Sydney School of Veterinary Science (Betty & Keith Cook Canine Research Fund, Schnakenberg Bequest) Speaking & consultancies: N/A Investments/commercial interests: N/A Gifts, hospitality, travel support: The author will be applying for Travel Funds for Academics from the Sydney School of Veterinary Science

**Source of Funding**

The study has been funded by MSD Animal Health and philantropic funding from the Sydney School of Veterinary Science (Betty & Keith Cook Canine Research Fund, Schnakenberg Bequest)

## ISCAID‐O‐13

### ISCAID—International Society for Companion Animal Infectious Diseases

#### Leptospira in community and privately‐owned cats in Hong Kong: serology and urinary shedding

##### WYJ Tam^1^, O Nekouei^2^, F Rizzo^3^, LST Cheng^1^, YR Choi^4^, M Staples^5^, S Hobi^1^, YF Chai^1^, J Gray^6^, FM Woodhouse^6^, JA Beatty^1^, VR Barrs^3^

###### ^1^ Department of Veterinary Clinical Sciences, Jockey Club College of Veterinary Medicine and Life Sciences, City University of Hong Kong, Kowloon Tong, Hong Kong Special Administrative Region of China; ^2^ Department of Infectious Diseases and Public Health, Jockey Club College of Veterinary Medicine and Life Sciences, City University of Hong Kong, Kowloon Tong, Hong Kong Special Administrative Region of China; ^3^ Jockey Club College of Veterinary Medicine and Life Sciences, City University of Hong Kong, Kowloon Tong, Hong Kong Special Administrative Region of China; ^4^Centre for Animal Health and Welfare, Jockey Club College of Veterinary Medicine and Life Sciences, City University of Hong Kong, Kowloon Tong, Hong Kong Special Administrative Region of China; ^5^ World Health Organisation Collaborating Centre for Reference and Research on Leptospirosis, at Queensland Health Forensic and Scientific Services, Brisbane, Australia; ^6^ Society for the Prevention of Cruelty to Animals (Hong Kong), Wan Chai, Hong Kong Special Administrative Region of China

Leptospirosis is an important zoonotic disease. Subclinically infected cats can act as reservoirs shedding leptospires into the environment. This study aimed to determine *Leptospira* serogroups, seroprevalence, prevalence of urinary shedding and risk factors for infection in Hong Kong cats.

Microagglutination testing (MAT) of 22 serogroups was performed on 739 serum samples from free‐roaming community (*n* = 392) and privately‐owned (*n* = 347) cats using a cut‐off of 1:100. Urine from 269 community cats underwent *LipL32* quantitative polymerase chain reaction (qPCR) for pathogenic leptospire DNA. Risk factors were analysed by logistic regression.

Seroprevalence was 9.34% (69/739) and 4.46% (12/269) of cats were shedding leptospires in urine (median load 10.41 copies/ng DNA, range 0.07–3344). Sixteen serogroups were detected at titres up to 1:6400, most frequently Javanica (4.33%), Djasiman (2.30%) and Australis (1.35%). Seroprevalence was higher in community (13.20%) than owned (4.90%) cats (OR 2.97 (95% CI 1.68–5.24), *P <*0.001). Community cats were more frequently exposed to serogroup Javanica (7.65%) than privately‐owned cats (0.58%) (*P <* 0.001). Among 233 community cats with paired samples, 1 in 3 seropositive cats had leptospiruria. After adjusting for provenance, none of breed, sex, neuter status, age, district rodent infestation levels, alanine transaminase or creatinine values were associated with seropositivity.

Cats in Hong Kong are exposed to a wide range of *Leptospira* serogroups. Greater exposure in community cats compared with privately‐owned cats likely reflects greater interaction with reservoir species or their urine. Although urinary *Leptospira* loads in cats were low, gloves should be worn when handling urine to reduce zoonotic risk.

**Disclosures**

No disclosures to report

**Source of Funding**

The study was supported by grants from CityU, HK to VRB (Project No 9380113) and JB (No. 9380111).

## ISCAID‐O‐14

### ISCAID—International Society for Companion Animal Infectious Diseases

#### Plasma procalcitonin and C‐reactive protein in dogs with suspected bacterial pneumonia or non‐bacterial pulmonary diseases

##### B Lutz^1^, J Rompf^1^, E Marti^1^, J Mirkovitch^1^, L Peters^1^, K Adamik^1^, B Willi^2^, S Schuller^1^

###### ^1^Vetsuisse‐Fakultät, Bern, Switzerland; ^2^Vetsuisse Fakultät, Zürich, Switzerland

Procalcitonin (PCT) is a biomarker for human bacterial infections. It is used to differentiate bacterial pneumonias from other causes of respiratory inflammation and to personalize the duration of antimicrobial treatments. It is also of prognostic value. C‐reactive protein (CRP) is a biomarker commonly used in canine bacterial pneumonia to support diagnosis and monitoring.

The aim of this prospective observational study was to determine the clinical utility of PCT and CRP as biomarkers for canine bacterial pneumonia.

Client‐owned dogs were categorized in three groups: healthy, suspected bacterial pneumonia (BP) and non‐bacterial pulmonary diseases (non‐BP). Dogs were classified into two disease groups depending on history, clinical signs, results of further examinations (compatible thoracic radiographic changes and/or cytologic evidence of septic neutrophilic inflammation) and response to treatment with antimicrobials. Blood was collected from diseased patients for hematology, PCT and CRP measurements on Days 1, 2, 3, 4 and 10 of hospitalization, and where available, on Day 21. Plasma concentrations of PCT and CRP were measured batch wise with an ELISA (Biovendor, Nashville, USA) and an automated immunoturbidimetric assay (Randox Reagents, London, UK), respectively, and compared between groups at admission and all further time points.

Thirty‐eight dogs were included: 15 healthy, 9 non‐BP and 14 BP dogs. At admission, CRP and band neutrophil concentration were increased in non‐BP and BP dogs compared to healthy (*P* < 0.0001). There was no significant difference in median PCT between groups at admission (healthy 119 pg/dL (range, 82–147), BP 82 pg/dL (range, 68–230), non‐BP 62 pg/dL (range, 44–79; *P* = 0.4). There was no difference in decrease in median PCT concentrations over the first 24 hours (*P* = 0.61). Conversely, the median decrease in CRP over the first 24 hours was significantly greater in dogs with PB compared to non‐BP (*P* = 0.002). The evolution of PCT over the first 4 days was not different between healthy, non‐BP and BP dogs (*P* > 0.05). BP The evolution of CRP and PCT over the first 4 days was not significantly different between survivors and non‐survivors (*P* = 0.09 and *P* = 0.22). The survival rate was not significantly different between BP and non‐BP (*P* = 0.83) but non‐BP dogs had a shorter hospitalization (*P* = 0.03).

This study suggests that PCT has limited clinical utility for the diagnosis and prognosis of canine bacterial pneumonia. On the other hand, CRP, particularly its decrease in the early management, shows more promise.

**Disclosures**

No disclosures to report

**Source of Funding**

The project was generously supported by a grant by the Swiss Federal Food Safety and Veterinary Office (FSVO Grant No 1.20.03).

## ISCAID‐O‐15

### ISCAID—International Society for Companion Animal Infectious Diseases

#### Antimicrobial use in 6270 European dogs: a retrospective cohort study (2019–2021)

##### K Scahill^1^, E MacLeod^1^, E O'Reilly^1^, U Grönlund^2^

###### ^1^University of Edinburgh, Edinburgh, UK; ^2^AniCura Group, Stockholm, Sweden

Antimicrobial stewardship programs (ASP) have been successful in reducing and optimizing antimicrobial use in human medicine and are being used increasingly in companion animal medicine. There is, however, a paucity of clinical information of antimicrobial use (AMU) in companion animals. The aim of the present study was to identify areas where antimicrobial stewardship interventions could have the highest impact. All canine systemic antimicrobial treatments were recorded during one week annually for three consecutive years (2019–2021) in 198 European first opinion practices and referral hospitals. One person in each location was responsible for data collection from their respective clinic's journal software. Information about treatment indication, duration and substance were recorded in an online questionnaire.

Information from 6270 canine antimicrobial treatments were available for analysis. The five most common indications of treatment were surgical prophylaxis (SAP) (27%), dermatological disease (11%), gastrointestinal disease (GID) (10%), wounds (10%) and lower urinary tract infections (UTI) (8%). Median duration of treatment (DOT) and the most frequently prescribed substance was SAP (5 days; amoxicillin‐clavulanic acid (ACA)), dermatology (10 days; first generation cephalosporins), GID (6 days; metronidazole), wounds (7 days, ACA) and UTI (7 days; ACA). Most dogs that received surgical prophylaxis (67%) were treated for >24 hours indicating a high use of postoperative prophylaxis. There is no indication of treatment for GID in the absence of sepsis. Dermatological disease had the highest DOT and can often be controlled by topical treatments and by focusing on the underlying etiology. Dogs with UTI were mostly treated with a broader spectrum (75%) than recommended and 10% were treated with high priority critically important antimicrobials (HPCIA). Also, DOT for UTI was longer than recommended, 78% were treated >5 days. More detailed information is needed to draw any conclusions on the appropriateness of wound treatment. DOT is likely to be unnecessarily long in most conditions, which could be due to a clinical research gap in optimal treatment durations.

The results of the present study suggest that antimicrobial stewardship interventions directed towards reducing AMU in postoperative prophylaxis (>24 h) and gastrointestinal disease could have high impact. Efforts should also be made to use a narrower antimicrobial spectrum and reduce DOT in UTI. Moreover, educational efforts in the treatment of dermatological conditions such as atopic dermatitis and other antibacterial topical treatments could also have a substantial impact on AMU in dogs.

**Disclosures**

The data was collected in a quality improvement program for a large veterinary company. It was part of one of the author's work duties for which salary was received.

**Source of Funding**

No funding was provided except salary for one of the authors (see disclosures)

## SCH‐O‐1

### SCH—Society of Comparative Hepatology

#### Cobalamin status derangements in dogs with portosystemic shunt

##### AL Proksch^1^, M Schneider^1^, J Langenstein^2^, N Bauer^1^, A Moritz^1^

###### ^1^Clinic for Small Animals, Internal Medicine, Justus Liebig University, Giessen, Germany; ^2^SYNLAB.vet Augsburg, Augsburg, Germany;

In dogs with congenital portosystemic shunt (cPSS), hepatic bypass results in malnutrition. Hypothesis was that cPSS induces hepatocellular cobalamin deficiency that normalizes after shunt closure.

Aims of this prospective study were (1) to compare serum cobalamin status (cobalamin, homocysteine, methylmalonic acid [sMMA]) and urinary MMA‐to‐creatinine‐ratio [uMMA:crea]) between cPSS‐dogs and matched (age and body weight) healthy dogs and (2) to compare cobalamin status before and three months after shunt closure.

Between July 2021 and June 2022, 34 healthy and 44 cPSS‐dogs were included. After 3 months, 36/44 cPSS‐dogs with complete or partial shunt closure were available.

There was no significant difference in cobalamin concentrations (*P* = 0.097) between healthy and cPSS‐dogs (median 464.2 pmol/L (range 222.1–834.7) vs. 601.1 pmol/L [133.6–2145]). However, sMMA in healthy dogs (median 83.8 μg/L [48.5–164.0] vs. 175.5 μg/L [26.5–815.9]) and uMMA:crea concentrations (median 2.6 [1.1–16.0] vs. 22.4 [2.8–156.9]) were significantly lower (*P* < 0.0001), and homocysteine concentration significantly higher (median 10.8 μmol/L [6.1–24.6; *P* < 0.0001] vs. 5.9 [2.5–18.3]) in healthy dogs vs. cPSS‐dogs, respectively. Despite normal (35/44 dogs) to increased (7/44 dogs) plasma cobalamin concentrations, behaviour of cobalamin intermediate products in cPSS‐dogs reflects cellular cobalamin deficiency. After shunt closure, cobalamin concentrations trended to normalize with significantly lower uMMA:crea concentrations (*P* = 0.003) after shunt closure. Comparing dogs with complete vs. incomplete shunt closure, sMMA (*P* = 0.0003) and uMMA:crea (*P* = 0.006) were significantly lower in dogs with complete shunt closure.

Cobalamin status in cPSS‐dogs suggests cellular cobalamin deficiency that normalizes after shunt closure. Whether cPSS‐dogs would profit from early cobalamin substitution needs to be further elucidated.

**Disclosures**

Study partially funded by SYNLAB.vet Augsburg; 2 authors work(ed) as consultant for Bayer Animal Health GmbH Leverkusen. Two authors have/had cooperations with Scil Animal Care Company GmbH and Sysmex Deutschland GmbH; two authors work as partial consultants for MSD Germany and one author for IDEXX laboratories

**Source of Funding**

Costs für cobalamin status measurements were given at reduced costs in patients and no costs in healthy dogs

## SCH‐O‐2

### SCH—Society of Comparative Hepatology

#### Association between hyperlipidaemia and selected cholestatic markers in 75 dogs with suspect acute pancreatitis

##### A J Da Silva^1^, A Hope^2^, E M Stavroulaki^1^, C T Mooney^1^

###### ^1^UCD Veterinary Hospital, Dublin, Ireland; ^2^VetsNow Referrals, Glasgow, UK

Hypercholesterolaemia and hypertrigyceridaemia occur in <25% of dogs with pancreatitis when concurrent diseases or drugs are eliminated as inciting factors. The underlying mechanisms are not well defined. The aim of this study was to investigate the association of hyperlipidaemia with other markers of cholestasis in dogs presenting with suspect acute pancreatitis.

Case records of dogs between January 2014 and June 2022 that had a point‐of‐care canine pancreatic lipase (cPL) (SNAP cPL, IDEXX Laboratories) measured were retrospectively reviewed and a diagnosis of acute pancreatitis made if there were ultrasonographic changes suggesting acute pancreatitis or the quantitative cPL was ≥400 μ/L and the clinician‐stated diagnosis was pancreatitis and treatment qualified the disease as acute.

In total, 889 dogs were identified, but only 213 cases fulfilled the criteria of suspect acute pancreatitis. In total, 138 cases were excluded because of pre‐existing disorders or drug therapies associated with hyperlipidaemia (*n* = 114), hypocholesterolaemia (*n* = 22) and incomplete biochemical data (*n* = 2). The remaining 75 dogs included 57 purebreds of 28 different breeds and 18 crossbreds ranging in age from 2 to 14 years.

There were 33 (44%) dogs with hypercholesterolaemia (HC) (median 8.35; range 6.6–21.79 mmol/L) and 42 (56%) without (NC) (median 5.02; range 3.23–6.47 mmol/L). The ALP activity was significantly (P < 0.001) higher in the HC group (median 932; range 140–24990 IU/L) compared to NC group (median 353; range 59–12150 IU/L). There was a strong positive correlation between cholesterol concentration and ALP activity (r = 0.516, P <0.001) and a moderate positive correlation between cholesterol concentration and gamma‐glutamyl transpeptidase (GGT) activity (r = 0.317, *P* = 0.006). There was no significant correlation between cholesterol concentration and alanine aminotransferase activity or bilirubin concentration. Triglyceride concentrations were only increased above the reference interval (0.11–1.69 mmol/L) in 17 (22.7%) cases and no value exceeded 5 mmol/L.

In this study, the prevalence of hypercholesterolaemia was higher compared to previous reports. Cholesterol concentration was strongly correlated with other cholestatic markers suggesting an association between cholestasis and hypercholesterolaemia in dogs with acute pancreatitis. Triglyceride concentrations did not increase to any clinically significant extent. If marked hypertriglyceridaemia is present with acute pancreatitis, alternative concurrent disorders should be investigated.

**Disclosures**

No disclosures to report

## SCH‐O‐3

### SCH—Society of Comparative Hepatology

#### Hepatic AA amyloidosis in shelter cats: clinico‐pathological data and light microscopic findings

##### C Palizzotto^1^, S Ferro^2^, G Buzzi^1^, F Ferri^1^, G Gerardi^3^, S Ricagno^4^, V Zappulli^2^, E Zini^1^

###### ^1^Anicura Istituto veterinario di Novara, Granozzo con Monticello, Italy; ^2^Department of Comparative Biomedicine and Food Sciences, Legnaro (PD), Italy; ^3^Department of Animal Medicine, Production and Health, University of Padova, Legnaro (PD), Italy; ^4^Institute of Molecular and Translational Cardiology, Milan, Italy

Amyloid‐A (AA) amyloidosis is a systemic disease characterized by tissue deposition of misfolded serum AA. The disease is reported as familial in Abyssinian and Siamese breeds. Recently, a prevalence between 52% and 73% was reported in domestic shorthair cats from three different Italian shelters; among affected cats, 79% had liver involvement. Whether liver deposition of AA is associated to clinical or laboratory findings is unclear. Hence, the aim of this study was to retrospectively explore medical records of shelter cats with AA amyloidosis and liver involvement, and perform hepatic histology.

Shelters cats were included if clinical and laboratory data performed within 30 days before death were available and if liver, spleen and kidney had been collected within 24 h from death. To confirm AA amyloidosis, Congo red staining and immunohistochemistry for AA were used. Optic microscopy was employed to characterize liver damage and score amyloid deposits.

Twenty‐two domestic shorthair cats were included. Nine were male and 13 were female, with a median age of 8 years (1–14). Twelve cats were affected by hepatic AA amyloidosis; 10 matched‐cats without AA amyloidosis in liver, kidney and spleen were included as negative control group. Among cats with hepatic AA amyloidosis, the disease was scored mild or moderate/severe in 6 cases each. Hepatic amyloidosis was associated with lobular diffuse atrophy (*P* = 0.033), hepatic cords disarrangement (*P* = 0.050) and sinusoid dilation (*P* = 0.050). Deposits scored as moderate were associated with centrilobular fibrils (*P* = 0.015). Cats with hepatic amyloidosis had erythrocytes with lower mean corpuscular volume [media*n* = 41 fL (range = 29–46) vs. 56.0 (38–47); *P* = 0.003] and mean cell hemoglobin [media*n* = 13 pg (range = 11–15) vs. 16 (13–21); *P* < 0.001], as well as higher leukocyte counts [media*n* = 16.5 k/mL (range = 10.1–31.9) vs. 10 (0.6–20.6); *P* = 0.015] and platelet counts [media*n* = 279 k/mL (range = 76–616) vs. 125 (42–437); *P* = 0.038], compared to those without deposits. Circulating hepatic enzymes and function parameters did not differ between affected and unaffected cats. Among cats with hepatic amyloidosis, the main cause of death was chronic kidney disease (41.7%), with only one cat (8.3%) dying of liver disease (spontaneous hepatic rupture).

In conclusion, shelter cats with hepatic AA amyloidosis have impaired liver architecture but hepatocellular damage does not seem clinically relevant. Routine blood works are not sufficient to identify affected cats, that are usually asymptomatic except for the most advanced cases. Microcytosis, hypochromia, leukocytosis and thrombocytosis might reflect the permissive role of concurrent inflammatory disorders on AA amyloidosis.

**Disclosures**

No disclosures to report

## SCH‐O‐4

### SCH—Society of Comparative Hepatology

#### Prevalence of canine cholangitis/cholangiohepatitis, a retrospective study based on 263 liver biopsy cases (2013–2021)

##### FB BEDEL^1^, MC CHEVALLIER^2^, MG GIROD^1^, PL LECOINDRE^1^, AL LECOINDRE^1^

###### ^1^ONLYVET, Saint Priest, France; ^2^Eurofins Biomnis, Lyon, France

Cholangitis is a well‐documented entity in cats, but the disease is poorly described and probably underestimated in dogs. The aim of this retrospective study was to describe the histological biliary lesions observed in dogs, to define their prevalence and to assess hepatic fibrosis to provide prognostic information. Indeed, cholangitis in humans often leads to fibrosis and irreversible liver cirrhosis.

Two hundred and sixty‐three dogs that underwent liver biopsy between 2011 and 2021 were included. They were presented with elevated liver enzymes or hepatobiliary abnormalities on ultrasound. Liver biopsies were performed either by US guided sampling (Tru‐cut), laparoscopy, laparotomy or a combination of techniques. All biopsies were analyzed by three board‐certified veterinary pathologists and one human pathologist specializing in liver disease, in accordance with the recommendations of the World Small Animal Veterinary Association's Liver Standardization Group. According to the histological diagnosis, the lesions were classified as neutrophilic cholangitis, lymphocytic cholangitis, destructive cholangitis and chronic cholangitis associated with liver fluke infestation. Masson's trichrome was used to identify the presence of fibrous connective tissue. The degree of hepatic fibrosis was assessed by a 3‐level score (absent to mild/moderate/severe).

Of 263 liver biopsies, 59 dogs had biliary lesions (22%). 47/59 (80%) were neutrophilic cholangitis/cholangiohepatitis, 10/59 (17%) were lymphocytic cholangitis/cholangiohepatitis. Two dogs had an unclassified cholangitis (eosinophilic cholangitis and presumed congenital biliary cyst). No dog had destructive cholangitis/cholangiohepatitis or chronic cholangitis/cholangiohepatitis associated with liver fluke. 83% (49/59) of the dogs had mild to absent fibrosis, 15% (9/59) had moderate fibrotic lesions and only one dog (2%) had severe liver fibrosis associated with biliary lesions.

Finally, this descriptive study suggests that canine cholangitis or cholangiohepatitis should be suspected more often than originally described. Histopathological lesions of the bile ducts are most often consistent with a neutrophilic inflammation. In our canine population, hepatic fibrosis was rarely associated with biliary inflammation, perhaps because lymphocytic cholangitis was less frequent in this cohort. The classification of canine cholangitis or cholangiohepatitis appears to differ from cats, due to the absence of parasitic and destructive cholangitis on histopathological analysis. The presence of neutrophilic inflammation on histopathology should prompt the clinician to look for gallbladder involvement and identify a bacterial strain associated with canine neutrophilic cholangitis/cholangiohepatitis, as previously described.

**Disclosures**

No disclosures to report

## SCH‐O‐5

### SCH—Society of Comparative Hepatology

#### Point‐of‐care viscoelastometric evaluation of dogs with chronic hepatitis

##### FC Dröes^1^, JP Cavasin^1^, PM Manino^2^, JM Steiner^1^, CR Rutter^3^, JA Lidbury^1^

###### ^1^Texas A&M University, Gastrointestinal laboratory, College Station, USA; ^2^GulfCoast Veterinary Specialists, Houston, USA; ^3^Texas A&M University‐Small Animal Veterinary Teaching Hospital, College Station, USA

The liver plays an essential role in hemostasis. Dogs with chronic hepatitis (CH) can therefore develop diverse coagulopathies (e.g., hypo‐, hypercoagulability, and hyperfibrinolysis). Plasma‐based coagulation tests might not identify the whole range of abnormalities in hemostasis in these patients.

The aim of this study was to evaluate hemostasis in dogs with CH using a point‐of‐care viscoelastic coagulometer (VCM Vet) that uses fresh whole blood and compare the findings to traditional plasma‐based coagulation tests.

Forty‐two healthy dogs (He) and 24 dogs with a histopathological diagnosis of CH were enrolled.

Prothrombin time (PT), activated partial thromboplastin time (PTT), concentrations of fibrinogen and D‐dimers, antithrombin (AT) activity, platelet count, and viscoelastic coagulometer parameters were concurrently evaluated at the time of histopathological diagnosis. Results were compared between He and CH dogs using Mann‐Whitney U‐test or t‐tests as appropriate with correction for multiple comparisons. Dogs were classified as hypocoagulable if clotting time (CT) or clot formation time (CFT) were prolonged, and alpha angle and maximum clot formation (MCF) were decreased, hypercoagulable if CT or CFT were shortened, and alpha angle and MCF were increased, and hyperfibrinolytic if clot lysis parameters were decreased.

In dogs with CH, PT and/or PTT were outside of the reference interval in 2 of 24 (8.3%) dogs, while the point‐of‐care viscoelastic coagulometer identified at least one abnormal value in 14 of 26 (53.8%) dogs. Dogs with CH had significantly increased CT (CH: median 395 sec [interquartile range {IQR} 331–426] vs. He: 334 [301–357]; p = 0.0020), and significantly decreased AT (CH: mean 116% [s.d. ± 4] vs. He: 136% [± 3]; *P* = 0.0002) compared to healthy dogs. Clot retraction or collapse was suspected in 2 of 24 dogs with CH (8.3%) and 8 of 42 healthy dogs (19.1%). After exclusion of these test results, 2 of 22 (9.1%) dogs with CH were classified as hypocoagulable, 19 of 22 (86.4%) normocoagulable, 1 of 22 (4.5%) hypercoagulable, and 4 of 22 (18.2%) hyperfibrinolytic. Four dogs classified as hyperfibrinolytic were also classified as normocoagulable.

In dogs with CH, this point‐of‐care viscoelastic coagulometer using fresh whole blood showed more abnormalities in hemostasis than traditional clotting times. Based on its results, 9% of dogs with CH were hypocoagulable, 5% were hypercoagulable, and 18% hyperfibrinolytic. Some dogs classified as normocoagulable also showed signs of hyperfibrinolysis. Suspected clot retraction or collapse might pose a challenge in evaluation of fibrinolysis using this method.

**Disclosures**

Entegrion (producer of VCM Vet cartridges) has supported this study by providing VCM Vet cartridges for this study. •At the time of this study:‐ Drs. Dröes, Cavasin, Steiner, and Lidbury are employed by the Gastrointestinal Laboratory at Texas A&M University, which offers laboratory testing, including hepatic histopathology services, on a fee‐for‐service basis.

**Source of Funding**

Entegrion (producer of VCM Vet cartridges) has supported this study by providing VCM Vet™ cartridges for this study.

## ESCG‐P‐1

### ESCG—European Society of Comparative Gastroenterology

#### Serial evaluation of the effects of antibiotic use on the intestinal microbiome in kittens using the feline dysbiosis index

##### E Chrysovergi^1^, EM Stavroulaki^1^, GM Steiner^2^, J Suchodolski^2^, PG Xenoulis^2,3^, R Pillar^2^, JA Lidbury^2^

###### ^1^University College Dublin Veterinary Hospital, Dublin, Ireland; ^2^Gastroenterology Laboratory, College of Veterinary Sciences, Texas A&M University, College Station, Texas, USA; ^3^Clinic of Medicine, Faculty of Veterinary Sciences, University of Thessaly, Karditsa, Greece

Studies assessing sequencing have shown that cats treated with antibiotics have significant changes of the intestinal microbiome. The feline dysbiosis index (DI) is a PCR‐based tool that was recently developed for use in cats to evaluate fecal microbiota. The aim of this study was to serially evaluate the DI in kittens receiving antibiotic treatment (AT) during early life.

Naturally passed feces were collected from 15 healthy control kittens (CON group), and 30 kittens receiving antibiotics. AT kittens were presented for treatment of upper respiratory tract disease and were randomly assigned to receive either amoxicillin/clavulanic acid (AMC; *n* = 15) for 20 days or doxycycline for 28 days (DOX; *n* = 15) as part of their standard treatment. Feces were collected on days 0 (baseline), 20 (last day of AT for the AMC group) or 28 (last day of AT for the DOX and control groups), 60, 120, and 300. Kittens were approximately 2 months of age at enrollment and received the same diet and antiparasitic treatment for the duration of the study. DNA was extracted from each fecal sample and qPCR assays were used for evaluating abundances of *Bacteroides*, *Bifidobacterium*, *Clostridium hiranonis*, *Escherichia coli*, *Faecalibacterium*, *Streptococcus*, and *Turicibacter*) to calculate the DI (reference interval <0). Data were assessed for normality and appropriate statistical analyses were used for independent and dependent measurements. Data are presented as means ± standard deviation. Statistical significance was set at *P <* 0.05.

No significant differences in the DI were observed among groups on day 0. Interestingly, the mean DI was abnormal in all groups on day 0 (CON 1.0 ± 1.3; AMC 1.1 ± 1.7; DOX 1.2 ± 1.5). The mean DI of the CON cats normalized by day 28 (−0.5 ± 1.8) but remained abnormal in both AT groups (AMC 1.8 ± 1.6; DOX 0.2 ± 1.7), with the AMC group having a significantly higher DI compared to CON cats (*P* < 0.01). For the remaining timepoints, no significant differences were observed, and the mean DI normalized in all three groups.

This study showed an increased mean DI in 2‐month‐old cats, which normalized by the age of 3 months in control cats. This process was disrupted during AT in both groups, and this was more prominent in cats receiving amoxicillin/clavulanic acid, which showed an increase in the DI compatible with dysbiosis. One month after discontinuation of AT, the DI normalized in both AT groups. The clinical significance and long‐term consequences of these findings require further research.

**Disclosures**

Dr. Steiner, Dr. Suchodolski, Dr Pillar and Dr. Lidbury work at the GI Lab at Texas A&M University which offers the assessment of the dysbiosis index (DI) for a fee of service.

## ESCG‐P‐2

### ESCG—European Society of Comparative Gastroenterology

#### Serial measurement of serum specific pancreatic lipase and trypsin‐like immunoreactivity concentrations in dogs and cats with diabetes mellitus

##### KT Moraiti^1^, F Del Baldo^2^, F Fracassi^2^, MN Pitropaki^1^, DA Karra^1^, VD Vaggeli^1^, JM Steiner^3^, PG Xenoulis^1^

###### ^1^University of Thessaly, Karditsa, Greece; ^2^University of Bologna, Bologna, Italy; ^3^Texas A&M University, Texas, USA

Diabetes mellitus (DM) is the most common disorder of the endocrine pancreas, while pancreatitis and exocrine pancreatic insufficiency (EPI) are the most common disorders of the exocrine pancreas in dogs and cats. While DM and pancreatitis commonly coexist in dogs and cats, the actual prevalence of pancreatitis and EPI in dogs and cats with DM is unknown. In addition, longitudinal studies on serum specific pancreatic lipase (Spec PL) and trypsin‐like immunoreactivity (TLI) concentrations in dogs and cats with DM have not yet been reported. The aim of this study was to longitudinally evaluate serum Spec PL and TLI concentrations in dogs and cats with DM.

Leftover serum samples from 51 dogs and 20 cats diagnosed with DM were used in this study. Samples were available from the initial presentation and from reexaminations (ranging from 1 to 13 for each animal). Serum Spec PL and TLI concentrations were measured in all samples. The reference interval for Spec fPL is currently being updated; the updated reference interval is used in the present study.

Serum Spec cPL and cTLI concentrations were indicative of pancreatitis (>400 μg/L) in 22/51 (43%) dogs with DM and of EPI (<2.5 μg/L) in 3/51 (6%) dogs, in at least 1 timepoint, respectively. Serum Spec fPL and fTLI concentrations were indicative of pancreatitis (≥8.8 μg/L) in 7/20 (35%) cats with DM and of EPI (≤8 μg/L) in 1/20 (5%) cats in at least 1 timepoint, respectively. Sixteen of 51 (31%) dogs and 5/20 (25%) cats had serum Spec PL concentration indicative of pancreatitis at the initial presentation. Two of 51 (4%) dogs and 1/20 (5%) cats had serum TLI concentration indicative of EPI at the initial presentation. Thirteen of 51 (25%) dogs and 4/20 (20%) of cats had serum Spec PL concentrations that were fluctuating within or above the reference interval during the study. One dog and 2 cats had persistently increased serum Spec fPL concentrations in all timepoints. Two of 3 (67%) dogs and the 1 cat with EPI had constantly low serum TLI concentrations.

In conclusion, the prevalence of increased Spec PL concentrations indicating pancreatitis in dogs and cats with DM is high, while a smaller percentage develop EPI. It might be necessary to retest dogs and cats with DM throughout their disease for pancreatitis and/or EPI as these diseases might not be present at the time of initial diagnosis of DM.

**Disclosures**

Dr. Steiner J.M. is the director of the GI Lab, which offers diagnostic testing on a fee‐for‐service basis and also serve as a paid consultant for IDEXX Laboratories, the manufacturer of the Spec fPL assay.

## ESCG‐P‐3

### ESCG—European Society of Comparative Gastroenterology

#### Comparative study of video capsule endoscopy findings in dogs with chronic enteropathy and in healthy dogs

##### J Holmberg^1^, I Ljungvall^2^, L Pelander^2^, A Defarges^3^, J Stiller^4^, J Ingman^5^, C Harlos^6^, T Spillmann^7^, J Häggström^2^

###### ^1^Swedish University of Agricultural Studies, Uppsala, Sweden; ^2^Swedish University of Agricultural Sciences, Uppsala, Sweden; ^3^Ontario Veterinary College, Guelph, Canada; ^4^Lakeshore Animal Health Partners, Mississauga, Canada; ^5^University Animal Hospital, Uppsala, Sweden; ^6^Anicura Albano Animal Hospital, Stockholm, Sweden; ^7^University of Helsinki, Helsinki, Finland

The aim of this prospective study was to investigate safety, and feasibility of the video capsule ALICAM® for evaluating and comparing macroscopic gastrointestinal morphology in dogs with chronic enteropathy (CE) and in healthy controls (HC). Fifteen dogs with CE and 15 breed, body weight and age matched HCs were included. The dogs were fasted 16 hours before and eight hours after they orally received an ALICAM. All recordings were masked, quality assessed, and evaluated by two trained observers. Macroscopic abnormalities were recorded when identified by both observers. The median body weight for all dogs was 27.4 kg, interquartile range (IQR) 20.1–30.6 kg, and the median Canine CE Activity Index was 6 (IQR 4–8) in CE dogs and 1 (IQR 0–1) in HC. All video capsules reached the colon within the recording time, without any complications, and complete recordings of adequate quality were obtained from all dogs. In the recordings, gastric‐ and small intestinal abnormalities were detected such as irregular mucosa (CE: 3/15, HC: 6/15), erythema (CE: 6/15, HC: 8/15), non‐bleeding erosions (CE: 1/15, HC: 0/15), bleeding erosions (CE: 1/15, HC: 3/15), and dilated lacteals (CE: 5/15, HC: 5/15). The proportions and extent of macroscopic abnormalities were comparable between the groups. In conclusion, the use of ALICAM® for evaluation of CE and HC dogs seems safe and feasible regarding gastrointestinal transit and macroscopic morphology assessment. However, abnormalities were found in similar proportions in CE and HC dogs. Further validation studies are required to reveal the causes for such unexpected findings.

**Disclosures**

No disclosures to report

## ESCG‐P‐4

### ESCG—European Society of Comparative Gastroenterology

#### Characterization of the intestinal microbiome of dogs with inflammatory brain disease compared to dogs with non‐inflammatory brain disease and healthy control dogs

##### KD Mathews^1^, CF Li^2^, LI Yoon^1^, ND Jeffrey^3^, KM Vernau^2^, R Pilla^3^, JS Suchodolski^3^, S Marsilio^1^

###### ^1^UC Davis School of Veterinary Medicine, Department of Veterinary Medicine and Epidemiology, Davis, CA USA.; ^2^UC Davis School of Veterinary Medicine, Department of Surgical and Radiological Sciences, Davis, CA USA.; ^3^Texas A&M School of Veterinary Medicine & Biomedical Sciences, Department of Small Animal Clinical Sciences, College Station, TX, USA.

The gut‐brain‐axis (GBA) describes an intricate, dynamic network between the central and enteric nervous system. Previous studies have revealed the importance of the intestinal microbiome as a key component of this axis, now known as the microbiota‐gut‐brain‐axis, with bidirectional communication between the microbiome and the central nervous system through neural, endocrine, immune, and humoral mediators. Perturbances of the microbiota‐gut‐brain‐axis have been associated with various diseases in people, including multiple sclerosis, Parkinson's disease, Alzheimer's disease, depression, anxiety, and Autism. One previous study investigated the role of two specific constituents of the fecal microbiota, *Faecalibacterium prausnitzii* and *Prevotellaceae* in dogs with inflammatory brain disease. This study aimed to characterize community shifts of the fecal microbiome in dogs with inflammatory brain disease (meningoencephalitis of undetermined origin, MUO) compared to dogs with non‐inflammatory brain disease (NIBD) and healthy control dogs.

Fecal samples from 20 dogs with a diagnosis of MUO, 23 dogs with NIBD and 21 healthy control dogs were collected. Healthy control dogs were age and weight matched to MUO dogs. Samples were batch analyzed using a quantitative PCR panel for total bacteria, *Faecalibacterium*, *Turicibacter*, *Escherichia coli (E.coli)*, *Streptococcus*, *Blautia*, *Fusobacterium* and *Clostridium hiranonis* to calculate a dysbiosis index (DI) as previously described.

Results showed that dogs with MUO had a significantly increased DI (median [range]: 0.6 [−3 to 7.9]) compared to healthy control dogs (median [range]: −3.5 [−6.7–2.8]) (*P* = .001). The DI of dogs with NIBD (median [range]: −1.3 [−6.1 to 8.4]) was not significantly different from healthy controls or dogs with MUO. Dogs with MUO showed a significantly higher abundance (*P* < .0001) of *E.coli* (log DNA, median [range]: 6.7 [2.2–8.4]) compared to healthy controls dogs (log DNA, median [range]: 3.1 [1.4–6.3]). Abundances of *Faecalibacterium* and *Blautia* was significantly lower (*P* = .003 and .025, respectively) in dogs with MUO (log DNA, median [range]: 3.7 [2.1–3.8] and 9.9 [6.8–10.7], respectively) compared to healthy control dogs (log DNA, median [range]: 5.6 [3.0–7.1] and 10.2 [9.8–10.5], respectively). In conclusion, dogs with MUO show a significant intestinal dysbiosis driven by an increased abundance of *E.coli* and decreased abundance of *Faecalibacterium* and *Blautia*.

**Disclosures**

No disclosures to report

**Source of Funding**

ACVIM resident research grant, Center for Companion Animal Health, UC Davis research grant

## ESCG‐P‐5

### ESCG—European Society of Comparative Gastroenterology

#### Interrelation between microbiota and intraepithelial and lamina propria duodenal T‐lymphocytes in dogs with inflammatory bowel disease

##### D Díaz‐Regañón^1^, B Agulla^2^, A Villaescusa^1^, F Rodríguez‐Franco^1^, A Sainz^1^, C Alba^1^, P Olmeda^1^, M García‐Sancho^1^

###### ^1^Veterinary College, Complutense University of Madrid, Madrid, Spain; ^2^Veterinary College, Autonomous University of Barcelona, Barcelona, Spain

The pathogenesis of canine inflammatory bowel disease (IBD) is multifactorial, but immunological dysregulation and gut microbiota shifts appear to play a central role. Thus, we aimed to characterize the immunophenotype of intraepithelial (IEL) and lamina propria (LPL) T‐lymphocytes of the duodenum as well as the fecal microbiota in dogs with IBD at the time of the diagnosis in order to find possible interrelations between these two pillars of the disease.

Twenty‐six dogs were included in the study (14 IBD dogs and 12 healthy dogs). T‐lymphocytes were isolated from duodenal biopsies collected during upper GI endoscopy. Phenotypic characterization of IELs and LPLs was performed by flow cytometry using a specific panel of monoclonal antibodies for the detection of different T‐lymphocyte populations. Bacterial DNA from fecal samples was amplified using universal primers targeting gene 16S rRNA and sequenced using the Illumina MiSeq platform. Taxonomy of amplicon sequence variants was assigned using the Naïve Bayesian classifier integrated in QIIME2 (v. 2022.2) plugins using SILVA 138.1 reference database. α–Diversity analysis were utilized to estimate the samples' diversity and richness. β–Diversity analyses were graphically explored by Principal Coordinates Analysis (PCoA).

The percentage of epithelial T‐helper (Th, CD3+CD4+) lymphocytes and CD4/CD8 ratio were significantly lower in IBD dogs (*P* = 0.021 and *P* = 0.008, respectively). Moreover, the percentage of T‐regulatory (T‐reg, CD3+CD4+CD25+FoxP3+) LPLs was higher in dogs with IBD (*P* = 0.014). In addition, IBD dogs presented lower α–diversity (Shannon index; *P* = 0.019), and differences in β–diversity (Bray‐Courtis; *P* = 0.036). When analyzing the interrelation between immunophenotype and taxonomy, dogs presenting lower percentage of Th IELs and CD4/CD8 ratio in the epithelium also showed lower relative abundance of *Blautia* genus (*P* = 0.030 and *P* = 0.004, respectively). Dogs with higher CD4/CD8 ratio in the epithelium presented higher α–diversity (Simpson index; *P* = 0.023). Furthermore, those with a lower expression of T‐reg LPLs also presented higher α–diversity (Simpson index; *P* = 0.037).

Our study describes for the first time the interrelation of intestinal immune system and microbiota during canine IBD, suggesting that these factors are closely interrelated and could be considered as potential biomarkers and targets in canine IBD.

**Disclosures**

No disclosures to report

**Source of Funding**

This study was supported by Proyecto Santander of the Complutense University of Madrid, Spain (references: PR26/16‐20319, PR41/17–21023, and PR 75/18–21623).

## ESCG‐P‐6

### ESCG—European Society of Comparative Gastroenterology

#### Evaluation of fecal microbiota transplantation as adjunct management of cats with chronic enteropathies in a controlled, blinded, randomized clinical trial

##### D Karra^1^, JA Lidbury^2^, J Suchodolski^2^, JM Steiner^2^, PG Xenoulis^3^

###### ^1^University of Thessaly, Karditsa, Greece; ^2^Texas A&M University, Texas, USA; ^3^University of Thessaly, Karditsa, Greece

Fecal microbiota transplantation (FMT) may play an important role in the management of cats with chronic enteropathies (CE). The aim of this controlled, blinded, randomized clinical trial was to evaluate the efficacy of FMT as an adjunct treatment for cats with CE.

Nineteen cats with CE that underwent upper and lower gastrointestinal endoscopy were included in the study. They were diagnosed with inflammatory bowel disease (*n* = 11), food‐responsive enteropathy (*n* = 3) or small‐cell gastrointestinal lymphoma (*n* = 5). Of these, 9 cats were randomly selected to receive one FMT via enema (FMT‐group) on the day of the endoscopy (T0) and the remaining 10 cats were used as controls. Clinical activity was determined using the Feline Chronic Enteropathy Activity Index (FCEAI) and intestinal dysbiosis was determined using the feline dysbiosis index (DI) at T0 and 30 days after endoscopy (T1). All cats received hydroxocobalamin supplementation, 5 cats in each group consumed a hydrolyzed‐protein diet, 2 cats in FMT‐group and 4 controls received prednisolone, and 1 cat in each group received prednisolone plus chlorambucil. Data are reported as mean±standard deviation and significance was set at *P* < 0.05.

FCEAI significantly decreased from T0 (8.7±2.8) to T1 (4.2±0.9; *P* = 0.0039) in the FMT‐group and in the control group from T0 (9.3±2.7) to T1 (5.1±2.1; *P* = 0.0002). No significant difference was found in FCEAI between the FMT and control groups at T0 or T1. No significant difference was found in the DI from T0 to T1 in either the FMT or the control group. Also, no significant difference in DI was found between FMT and the control group at T0 or T1. In the FMT‐group, 5/9 cats had an increased DI (>0) at T0 and remained increased in 4 of those at T1, while 4/9 cats had a normal DI (<0) at T0 and in 2 of those DI became abnormal at T1. In the control group, 6/10 cats had increased DI at T0 and remained increased in all at T1, while 4/10 had a normal DI at T0 and in 2 of those it became abnormal at T1.

In this study, one FMT enema as adjunctive treatment did not lead to a significant improvement in DI 30 days after the administration of FMT in cats with CE compared to controls. No difference in clinical activity score was found in cats that received FMT compared to controls. Further studies are needed to investigate the efficacy of FMT in cats with CE.

**Disclosures**

Joerg Steiner is the director of the Gastrointestinal Laboratory at Texas A&M University (GI lab), which offers microbiome testing including the Dysbiosis Index on a fee‐for‐service basis. Jan Suchodolski is an employee of the GI lab, and the Purina Petcare Endowed Chair for Microbiome Research, and has received consulting and/or speaking fees from IDEXX Laboratories, Purina Petcare, Royal Canin, Hill's Pet Nutrition, Blue Buffalo, Exegi Pharma, and Nutramax Laboratories. Jonathan Lidbury is an employee of the GI lab.

**Source of Funding**

No funding

## ESCG‐P‐7

### ESCG—European Society of Comparative Gastroenterology

#### Chronic enteropathy and emotional health in dogs—a prospective comparative pilot study

##### U Ludvigsson^1^, S Heath^2^, S Heath^3^, CT Tooley^2^, LT Toresson^1^, L Toresson^4^

###### ^1^Evidensia Specialist Animal Hospital, Helsingborg, Sweden; ^2^Behavioural Referrals Veterinary Practice, Chester, UK; ^3^University of Liverpool School of Veterinary Science, Liverpool, UK; ^4^Helsinki University, Helsink, Finland

The gastrointestinal tract and centres for cognition and emotions in the brain are connected through the gut‐brain axis. The correlation between emotional health and gastrointestinal disorders is evidenced in humans, but less studied in dogs. Emotional health in dogs can be evaluated from several aspects. The Heath model emphasises the equal importance of emotional valence and emotional arousal. The Positive and Negative Activation Scale (PANAS) is a validated questionnaire to evaluate valence. Valence relates to whether emotions are related to positive or negative behavioural responses. Besides valence, it is important to consider total emotional arousal. Displacement behaviours are behaviours performed out of context, when in a high arousal state. The authors’ clinical impression is that several dogs with chronic enteropathy (CE) also suffer from compromised emotional health. However, data from dogs is lacking.

A questionnaire‐based case‐control study was performed at a referral animal hospital from June 2022 to February 2023. Dogs with CE, between 1 and 10 years of age, visiting the gastroenterology service were included after informed owner consent. Control dogs were sampled from dogs visiting the hospital for preventive services and by advertising on social media. The control dogs were matched to the CE dogs based on age and Kennel club breed group. Exclusion criteria for control dogs were CIBDAI >3, a diagnosis of CE and/or chronic extraintestinal diseases. Participating dog owners completed an online questionnaire, including questions regarding CIBDAI and PANAS. The questionnaire also included a separate, non‐validated, section with questions on frequency of displacement behaviours.

Thirty‐five CE dogs with corresponding matched controls were included. Wilcoxon matched‐pair signed rank test was used for statistical comparisons. Dogs with CE scored higher on negative activation than control dogs (*P* = 0.0002). Compared to control dogs, CE dogs had significantly lower scores for positive activation regarding energy and interest (*P* = 0.038), and higher frequency of displacement behaviours when interacting with a familiar dog (*P* = 0.046), interacting with a familiar (*P* = 0.0024) or unfamiliar person (*P* = 0.048) and during a walk (*P* = 0.0004).

Positive and negative emotional activation affects an individuals’ behaviour tendencies. High negative activation is associated with fear‐anxiety and frustration related behaviours. A high frequency of displacement behaviours indicates high levels of emotional arousal, which, if excessive in duration or intensity, can negatively impact emotional health. This pilot study suggests that there is a link between CE and compromised emotional health in dogs. Emotional assessment in general practice may therefore be beneficial. Further studies are warranted.

**Disclosures**

No disclosures to report

**Source of Funding**

The Ulla Yard Foundation The Swedish Veterinary Care Foundation.

## ESCG‐P‐8

### ESCG—European Society of Comparative Gastroenterology

#### Overview of faecal pathogens isolated from faeces of French young cats and dogs using PCR technique over a year (2022)

##### C Peyron,

###### Idexx Laboratories, Saint‐Denis, France

Infectious diarrhoea is commonly encountered in young cats and dogs, but the incidence of the numerous pathogens encountered across different countries is poorly described. The aim of this study was to describe the pathogenic agents isolated using PCR on faeces from French young cats and dogs, according to their age.

Data from all the faecal PCR performed in 2022 in an international reference laboratory were retrospectively collected. Cats and dogs were divided into age groups: ≤6 months (group 1) and >6 but <24 months (group 2).

428 cats, 237 in group 1 (C1) and 191 in group 2 (C2) and 309 dogs, 169 in group 1 (D1) and 140 in group 2 (D2) were included. In cats, pathogens were detected with quite similar orders of frequency and proportions for *Clostridium perfringens* alphatoxin (73.3% in C1 and 72.8% in C2), feline Coronavirus (68.7 and 64.9%), Giardia (24 and 20.4%) and Cryptosporidium (21 and 18.3%). *Tritrichomonas* was more frequently detected in cats older than 6 months (11.4% in C1 versus 19.9% in C2), unlike feline Parvovirus (7.6 versus 3.3%). The alphatoxin was found as a co‐positive result in more than 70% of feline Coronavirus positive cases. In dogs, more differences were noted between D1 and D2 regarding frequencies, particularly for *Giardia* (53 versus 34.3% respectively), *Cryptosporidium* (33 vs. 16.4%), canine Coronavirus (19.5 vs. 5%) and canine Parvovirus (10.6 vs. 1.4%). The alphatoxin was a co‐positive result in 100% of enterotoxin positive cases, more than 80% of *Cryptosporidium* positive cases, and more than 80% of *Giardia* positive cases. In the same way, *Giardia* was co‐detected in more than 60% of *Cryptosporidium* positive cases. In both species, alphatoxin was detected in a very high proportion of cases (72.8% in C2 to 85% in D2) and co‐positive rates were similar across age groups.

This study suggests that age can have an impact on the pathogens isolated in faeces from young dogs, as PCR positive results were more common in samples from dogs <6 months than in the older group, whereas cats didn't show a strong age effect. Alphatoxin was the most common finding in both cats and dogs, and it was a common co‐positive in cats positive for Coronavirus and dogs positive for *Giardia* and *Cryptosporidium*. To our knowledge, it is the first time such a study was conducted in France.

**Disclosures**

I work as a medical consultant for Idexx Laboratories. The data I use in my study are results from PCRs performed at Idexx.

## ESCG‐P‐9

### ESCG—European Society of Comparative Gastroenterology

#### Serial measurement of serum‐specific pancreatic lipase and trypsin‐like immunoreactivity concentrations in cats during treatment for chronic enteropathy

##### DA Karra^1^, KT Moraiti^2^, JA Lidbury^3^, JS Suchodolski^3^, JM Steiner^3^, PG Xenoulis^2^

###### ^1^University of Thessaly, Karditsa, Greece; ^2^University of Thessaly, Karditsa, Greece; ^3^Texas A&M University, Texas, USA

Chronic enteropathies (CE) are common in cats and are often associated with pancreatic diseases, mainly pancreatitis and, less commonly, exocrine pancreatic insufficiency (EPI). The actual incidence of pancreatitis and EPI in cats with CE at initial presentation and during treatment is unknown. The aim of this study was to serially evaluate serum‐specific pancreatic lipase (Spec fPL) and trypsin‐like immunoreactivity (fTLI) concentrations in cats with CE before and during treatment for CE.

A total of 91 samples from 28 cats with CE (13 with idiopathic inflammatory bowel disease (IBD), 3 with food‐responsive enteropathy and 12 with small cell gastrointestinal lymphoma) were used. All cats were treated with SC cobalamin supplementation, 11 cats received a hydrolyzed‐protein diet, 11 cats were treated with prednisolone and 9 with prednisolone plus chlorambucil. Serum samples were available from the initial presentation as well as from reexaminations (1–8 reexaminations per cat during 1–22 months after initiation of treatment). Serum Spec fPL and fTLI concentrations were measured in all samples. The reference interval for Spec fPL is currently being updated; the updated reference interval is used in the present study.

Serum Spec fPL was indicative of pancreatitis (≥8.8 μg/L) in 15/92 (16.3%) samples. Serum Spec fPL concentrations indicative of pancreatitis were present in only 1/28 (3.5%) cats at initial presentation and in 4/28 (14.3%) cats during treatment. Two of these had initially normal Spec fPL and 2 had initial values within the equivocal zone (4.4–8.8 μg/L). Serum fTLI concentrations were indicative of EPI (≤8 μg/L) in 2/86 (2.3%) samples and in 2/28 (7.1%) cats. In 1 of those, fTLI was indicative of EPI at the initial presentation but was within the equivocal zone (8–12 μg/L) at 4 subsequent measurements. No additional measurements were available in the other cat with a fTLI concentration indicative of EPI.

Serum Spec fPL concentrations indicative of pancreatitis are relatively common in cats with CE, while serum fTLI concentrations indicative of EPI are less common but were still observed. Serum Spec fPL concentrations can be normal at initial presentation and increase later during treatment. Repeated testing for concurrent pancreatic disease during treatment in cats with CE may be indicated. Further studies are needed to determine the clinical importance of these findings.

**Disclosures**

Joerg Steiner is the director of the Gastrointestinal Laboratory at Texas A&M University, which offers diagnostic testing on a fee‐for‐service basis including fPL and TLI and also serves as a paid consultant for idexx laboratories, the manufacturer of the Spec fPL assay. Jonathan Lidbury is an employee of the Gastrointestinal Laboratory at Texas A&M University, which offers diagnostic testing on a fee‐for‐service basis including fPL and fTLI.

**Source of Funding**

No funding

## ESCG‐P‐10

### ESCG—European Society of Comparative Gastroenterology

#### Relationship between 1,2‐O‐dilauryl‐rac‐glycero glutaric acid‐(6’‐methylresorufin) ester (DGGR) lipase activity and clinical signs in dogs with chronic gastrointestinal signs over time

##### J Tang^1^, M Seth^2^

###### ^1^Dick White Referrals Six Mile Bottom, Cambridge, UK; ^2^KGS Veterinary Services Limited Saffron Walden, Essex, UK

Serum 1,2‐O‐dilauryl‐rac‐glycero glutaric acid‐(6’‐methylresorufin) ester (DGGR) lipase activity is widely used to identify pancreatic inflammation in dogs. A recently published study described serial monitoring of DGGR lipase activity in dogs with chronic pancreatitis, without comorbidities, concluding that elevations in activity were associated with clinic readmissions. Chronic pancreatitis is often identified alongside other states recognised to alter DGGR lipase activity, including chronic enteropathies, hepatobiliary disease, endocrinopathies, immune‐mediated diseases and subsequent corticosteroid therapy.

The primary aim of this study was to investigate for any correlations between serial DGGR lipase activity and chronic gastrointestinal signs in a population of dog with heterogeneous diseases. A secondary aim was to describe if corticosteroid therapy and diet manipulation were associated with serial DGGR lipase activity alterations in this population.

The records of a single referral hospital were searched retrospectively for dogs with elevated DGGR lipase activity between April 2015 and April 2021. Diagnosis of chronic pancreatitis and episodes of relapse were based on clinical signs, history, DGGR lipase activity, and sonographic findings where available. Fifty‐two dogs were included in this descriptive study and follow‐up time ranged from 15 to 1558 days. Twenty‐two dogs received corticosteroid therapy during the study period, and seventeen dogs received dietary manipulation.

Results identified a positive association between DGGR lipase activity and severity of clinical signs over time. Of the dogs who received corticosteroid therapy, five dogs had an initial increase in lipase activity when corticosteroid therapy was initiated with subsequent decrease in lipase activity as corticosteroid therapy was tapered. Another five dogs had progressive decrease in their lipase activities following initiation and subsequent tapering of corticosteroid therapy. No association was found between dietary manipulation, using various diets, and serial DGGR lipase activity.

This descriptive study suggests that serial monitoring of DGGR lipase activity appears to be a useful tool to help monitor the clinical course of chronic gastrointestinal signs in a heterogeneous population of dogs. Additionally, DGGR lipase activity generally decreases alongside dose reduction of corticosteroid therapy. However, it is uncertain if this observation reflects improvement in the underlying disease, a reduced direct effect of the corticosteroid, or a combination of both.

**Disclosures**

No disclosures to report

## ESCG‐P‐11

### ESCG—European Society of Comparative Gastroenterology

#### Prevalence of duodenal lymphangiectasia in French Bulldogs with obstructive airway syndrome undergoing endoscopic examination of the respiratory tract and upper digestive tract: retrospective study in 29 cases (2021–2022)

##### A Cocci^1^, V Greci^2^, S Monti^3^

###### ^1^Clinica Veterinaria San Siro, Anicura, Milano, Italy; ^2^Ospedale Veterinario Gregorio VII BluVet, Rome, Italy; ^3^Clinica Veterinaria Valdostana, Aosta, Italy

Intestinal lymphangiectasia is a disorder characterized by dilation, obstruction and dysfunction of the lymphatic vessels within the small intestine. The prevalence of gastro‐oesophageal lesions in brachycephalic dogs is reported to be very high in several studies while endoscopic duodenal mucosal lesions like “rice grain appearance” attributed to dilated lacteals are sometimes reported.

The aim of this study is to assess the prevalence of duodenal lymphangiectasia (DL) in French Bulldogs affected with brachycephalic obstructive airway syndrome with or without gastrointestinal clinical signs.

Inclusion criteria were availability of complete medical records including signalment, clinical signs, blood work, endoscopic records of the respiratory and upper digestive tract, and histopathology reports of gastroduodenal biopsies.

Endoscopic DL was graded on a scale of 0–3 (0 absence, 1 mild, 2 moderate, 3 severe); histopathological DL was graded on a scale of 0–3 according to the WSAVA guidelines.

Twenty‐nine dogs met the inclusion criteria, 20 male (19 intact, 1 neutered) and 9 female (6 intact, 3 spayed), mean age was 25.6 months and mean body weight 12.2 kg.

Sixteen dogs had no gastrointestinal clinical signs, 6 regurgitation, 2 vomiting, 2 small bowel diarrhea and 3 dogs concurrent vomiting and diarrhea.

Endoscopically DL was absent in 6/29 dogs, mild in 18/29, moderate in 4/29 and severe in 1/29.

On histopathology DL was absent in 4/29 dogs, mild in 19/29, moderate in 5/29 and severe in 1/29.

Endoscopic and histopathological agreement was present in 23/29 dogs, 21/23 with both endoscopy and histopathology and 2/23 without endoscopy and histopathology; 4/29 dogs without endoscopic DL, had DL on histopathology and 2/29 dogs with endoscopic DL, had no evidence of DL on histopathology.

All dogs with vomiting and/or diarrhea (5/29) had endoscopic and histopathological diagnosis of DL, of the 16 dogs without clinical signs 10 dogs had endoscopic and histopathological diagnosis of DL, 4/16 without endoscopic DL had DL on histopathology and 2/16 with endoscopic DL had absence of DL on histopathology.

Sensibility and specificity of endoscopy for DL was 91.3 % and 88.4% respectively.

This study showed a higher agreement 79.3% (23/29) between endoscopic and histopathological DL than those previously reported. Interestingly 12/16 (75%) of the French Bulldogs without gastrointestinal signs showed histological DL. The pathological significance of DL should be further investigated in this breed.

**Disclosures**

No disclosures to report

## ESCG‐P‐12

### ESCG—European Society of Comparative Gastroenterology

#### Holter analysis in dogs with acute and chronic diarrhea

##### I Spalla^1^, Y Nebel^1^, M Caccia^1^, V Caroli^1^, A Galizzi^1^, R Huober^1^, M Falco^1^, P Gianella^2^, R Ferriani^1^, R Toschi Corneliani^1^

###### ^1^Ospedale Veterinario San Francesco, Gallarate, Italy; ^2^Università degli Studi di Torino, Torino, Italy

Diarrhoea is a frequent presenting complaint in internal medicine and is often a primary sign of gastrointestinal disease, which can affect cardiovascular function via several mechanisms including autonomic dystonia, acid‐base disturbances, electrolyte abnormalities, and circulating inflammatory cytokines and gut microbial metabolites. Heart rate variability may differ depending on the underlying disease process and the duration of clinical signs. The aim of the present study was to assess the heart rhythm in dogs with a normal cardiovascular system presenting for acute (signs lasting less 7 days) or chronic diarrhoea (more than 14–21 days). This prospective study included 32 dogs with acute (*n* = 17) or chronic diarrhoea (*N* = 15). Dogs with acute diarrhoea included acute haemorrhagic diarrhea syndrome (9), acute idiopathic diarrhoea (5), parvoviral enteritis (3), whereas dogs with chronic diarrhoea had chronic inflammatory enteropathy. Dogs underwent complete physical examination, echocardiogram, bloodwork (haematology, biochemistry, blood venous gas analysis) and others diagnostics if required. A 24‐hour Holter monitor was fitted within 12 hours from admission; heart rate and time domain indexes of heart rate variability were investigated with statistical analysis. Most dogs were cross‐breeds (15); mean age was 7 years (3 months to 17 years), with no major sex differences (18M/14F). Mean heart rate was 93 bpm (61–136), minimum heart rate was 52 bpm (31–101), maximum heart rate was 224 bpm(148–283). Dogs with acute diarrhea had statistically higher minimum HR and medium HR (*P* < 0.001, *P* = 0.006 respectively). No statistically significant difference was noted within groups for maximum heart rate. Nine dogs did not show atrial or ventricular arrhythmias, whereas 17 dogs had ventricular (range 1–14235) and 13 dogs had supraventricular arrhythmias (1–300), with 7 dogs displaying both supraventricular and ventricular arrhythmias. Dogs in the chronic diarrhoea group had mean longest RR intervals during the recordings (*P* = 0.003). Dogs with acute diarrhoea had shorter SDNN (155 vs. 204, *P* = 0.009), SDNN index (119 vs. 150, *P* = 0.039) and SDANN (90 vs. 122, *P* = 0.005). In conclusion, dogs with acute diarrhoea do not tend to display a typical parasympathetic activation, likely due to the hemodynamic changes associated with acute volume losses. In contrast, dogs with chronic enteropathy are more likely to show an increase in parasympathetic system activation.

**Disclosures**

No disclosures to report

## ESCG‐P‐13

### ESCG—European Society of Comparative Gastroenterology

#### Psyllium husk powder increases defecation frequency and fecal score, bulk and moisture in healthy cats

##### E Keller^1^, IMJ Van Hoek^1^, J Laxalde^1^, N Tranier^1^, A Jackson^2^

###### ^1^Royal Canin SAS, Aimargues, France; ^2^TSG consulting, Knaresborough, UK

Psyllium is a low‐fermentable soluble fiber with water holding properties, that exudes mucilaginous gel upon lubrication. This study evaluated the effect of a diet containing psyllium in healthy cats on defecation frequency, fecal score, bulk, and moisture.

Nine healthy adult neutered cats (age (median (range) 3.7 years (4.4–3.3), 6 females, 3 males) were consecutively fed a dry extruded diet containing either 6% psyllium‐ (Test) or 6% powdered cellulose (Control) over a 10‐day digestibility trial. Feces were collected the last 3 days. The number of bowel movements and fecal score (1=hard and dry, 2.5 = optimum, 5=liquid diarrhea) were recorded, and fecal wet weight and fecal moisture were measured daily over those 3 days. Statistical analysis (*P* < 0.05) between the diets for these endpoints was performed with Paired *t*‐test or Wilcoxon signed rank test depending on normal distribution. Results are expressed as median (min–max) unless stated otherwise.

Test diet led to a significant increase of bowl movements over 3 days (*P* = 0.026, Test 5 (2–8), Control 3 (2–5)) and on day 3 (*P* = 0.0353, Test 2 (1–2), Control 1 (1–2)), mean fecal score over 3 days (*P* = 0.0213, Test 3.24 (2.3–4.06, Control 2.6 (1.4–3.1)) and on day 3 (*P* = 0.0066, Test 3.4 (2.6–4), control 2.6 (1.4–3.4)), total wet weight (kg) (*P* = 0.0003, Test 0.25 (0.06–0.430), Control 0.15 (0.03–0.21)) and total moisture (%) (*P* = 0.0426, Test 72.9 (66.11–81.5, Control 66.45 (41.45–72.9). Description of feces with Test diet was often noted as unusual with an exterior dry appearing shell and soft interior, leading to a higher score.

Psyllium promotes bowel movements, fecal moisture, and fecal softer consistency, all considered properties of benefits in the management of feline constipation. Moreover, its propensity to form gel, as observed in this study, will promote the transit of feces. Those observations are in agreement with an uncontrolled clinical trial supporting the use of psyllium in the management of severe constipation in cats.

**Disclosures**

Emeline Keller, Jeremy Laxalde, Nelly Tranier and Ingrid van Hoek are associates of Royal Canin SAS.

**Source of Funding**

Research was funded by Royal Canin SAS.

## ESCG‐P‐14

### ESCG—European Society of Comparative Gastroenterology

#### Fecal microbiota transplantation in dogs with tylosin responsive enteropathy—A proof of concept study

##### M Hanifeh^1^, M Huhtinen^2^, T. Laine^2^, E Scarsella^3^, H Ganz^3^, T Spillmann^1^

###### ^1^University of Helsinki, Helsinki, Finland; ^2^Orion Corporation, Espoo, Finland; ^3^AnimalBiome, Oakland, USA

Eighty‐six percent of dogs with tylosin responsive enteropathy (TRE) relapse within one month after tylosin discontinuation. This can lead to long‐term use of the antibiotic. Fecal microbiota transplantation (FMT) is considered an alternative to long‐term antibiotic therapy. This proof‐of‐concept study was a prospectively designed, double‐blinded, placebo controlled clinical trial and aimed at assessing the efficacy of oral freeze‐dried FMT capsules from one healthy dog donor to treat TRE in pet dogs.

The trial consisted of recruitment, inclusion, and treatment phases with a treatment and a post‐treatment period. Primary efficacy variable was the canine chronic enteropathy clinical activity index (CCECAI). Of 55 dogs from owners volunteering to the trial, 29 dogs were suspect to TRE and screened. Inclusion criteria were met by 14 dogs. They were randomized to either the FMT (*n* = 7) or placebo group (*n* = 7) and entered the treatment phase. All 14 dogs had CCECAI scores >3 when being without tylosin. All dogs received tylosin and when CCECAI was ≤ 3, tylosin was discontinued and FMT/placebo capsules were given daily for four weeks (treatment period). In the post‐treatment follow‐up period (4 weeks), the dogs did not receive any treatment.

Based on CCECAI, 5/7 (71.4%) dogs of the FMT group responded in the treatment period and 2/6 (33.3%) dogs remained without clinical signs in the post‐treatment period. One dog was excluded due to receiving corticosteroids. In the placebo group, one dog developed pyometra and was excluded. Three of six remaining dogs (50%) relapsed during the treatment period, but three dogs had no clinical signs throughout both periods. There was no significant difference between FMT and placebo groups in the primary efficacy variable at different time points. Mild to moderate adverse events (AE) without causal relationship to the study treatment were recorded in 2/7 FMT dogs and 5/6 placebo dogs. However, in two dogs of the FMT group unexpected severe AEs were recorded during the post‐treatment period (acute pancreatitis, bacterial fibro‐necrotizing cholangitis). Both dogs were responders in the FMT treatment period and clinical signs of relapse occurred > 10 days after stopping FMT.

In conclusion, the FMT efficacy was slightly higher than placebo, but the difference was statistically not significant since the study was underpowered. A causal relationship between the unexpected severe AEs and FMT seems unlikely but cannot be ruled out. In humans, delivery‐ or microbiota‐related severe AEs have been reported in 1.4% of patients receiving FMT.

**Disclosures**

Mirja Huhtinen and Tarmo Laine are employed by Orion Corporation, Espoo, Finland; Elisa Scarsella and Holly Ganz are employed by AnimalBiome, Oakland, USA.

**Source of Funding**

This research was funded by Orion Corporation, Espoo, Finland.

## ESCG‐P‐15

### ESCG—European Society of Comparative Gastroenterology

#### Characterization of underlying causes, treatment approaches, short and long‐term outcome in young cats with chronic recurrent gastrointestinal signs

##### F Signorelli^1^, M Carvalho^2^, R Oliveira Leal^3^, F Procoli^1^

###### ^1^Anicura Ospedale Veterinario I Portoni Rossi Zola, Predosa, Italy; ^2^Veterinary Teaching Hospital, Fac. Vet. Med. ULisboa, Lisbon, Portugal; ^3^Centre for Interdisciplinary Research in Animal Health, Fac.Vet.Med., ULisboa, Lisbon, Portugal

In young cats, endoparasitoses and food‐related disorders are believed to be the most common causes of chronic gastrointestinal (GI) disease. Studies specifically investigating underlying aetiologies, treatment, and long‐term outcome in this population are lacking.

This study aims to characterize signalment, clinical presentation, presumptive diagnoses, treatment approaches, short and long‐term outcome in young cats (<2 years old) with chronic (>3 weeks duration) GI signs.

A retrospective descriptive multicentric study was conducted. Medical records of young cats referred for chronic GI signs presented to two referral hospitals in southwestern Europe between September 2018‐ December 2022 were reviewed.

Thirty‐six cats were included. There were 22 males (61.1%) and 14 females (38.9%), with a median age of 370 days (range 56–721). Twenty‐one were purebred (58.3%). The most prevalent clinical sign was diarrhoea, present in 28 cases (77.7%).

At the time of presentation, 24 cats (66.6%) were already on a therapeutic diet. Fifteen cats (40.5%) had been treated with at least one antibiotic, being metronidazole the most frequently used (14/15; 93.3%). Faecal diagnostics (coprology and/or ELISA or PCR panels) were performed in 26/36 cats (72.2%) being positive in 11 cases (42.3%) for at least one parasite.

A sequential therapeutic approach was performed in 28/36 cats (77.7%) with 20 of them (55.5%) undergoing a dietary change (hydrolyzed 9, novel protein 5, fiber‐enriched 6). In eight cats, the prescription diet was not changed. Cats that did not respond to dietary change received deworming (2), antibiotics (3), faecal microbiota transplantation (3) or glucocorticoids (2) treatment.

Of the 28 cats that underwent a sequential therapeutic approach, short‐term follow‐up (1–3 months) was available for 20/28 (71.4%). Of them, 14/20 (70%) were considered food‐responsive, 4/20 (20%) were microbiome modulation responsive and 2/20 (10%) responded to deworming treatment. Long term follow‐up (5 months to 2 years from presentation) was available for 26/28 (92.9%) cats: 19/26 (73%) were in complete remission, while the remaining still showed recurrent GI signs.

Our study emphasizes the role of diet in the management of chronic GI signs in young cats, with most cases responding to dietary change. A relevant percentage of these cases were receiving chronic antimicrobial treatment at the time of presentation, despite a lack of clear indication and the persistence of clinical signs. Although endopararasitoses should always be ruled out in young cats presented with chronic GI signs, their role was minimal in this cohort according to the observed therapeutic response.

**Disclosures**

Fabio Procoli Speaking & consultancies: Nestle Purina, Hills, Royal Canin Waltham, Idexx laboratories, NBF lanes; Rodolfo Oliveira Leal Speaking and consultancies: Veterinary Information Network (USA), Vet2Vet, Boehringer Ingelheim, Dechra

## ESCG‐P‐16

### ESCG—European Society of Comparative Gastroenterology

#### Effect of various storage conditions on bacteria viability in fecal microbiota transplantation preparations

##### B Correa Lopes, M Tolbert, P Giaretta, J Suchodolski, R Pilla

###### Gastrointestinal Laboratory, Texas A&M University College Station, TX, USA

Chronic enteropathy (CE) is a multifactorial disease leading to changes in gastrointestinal microbiota. Alterations in intestinal environment homeostasis in dogs with CE can cause reduction or loss of *Clostridium hiranonis*, *Faecalibacterium*, *Turicibacter*, *Blautia*, and *Fusobacterium*. Fecal Microbiota Transplantation (FMT) can be useful as adjunct treatment for CE and improve bacterial diversity. Different protocols are used to prepare and store FMT preparations, but the effect of these methods on microbial viability is unknown. *C. hiranonis* is a strict anaerobe and a functional biomarker for gastrointestinal diseases, which may serve as proxy for the survival of other anaerobes. We aimed to assess the viability of different bacteria using a quantitative PCR that assesses viability, as well as the viability of *C. hiranonis* by culture to compare methods for conservation of bacteria in FMT preparations.

Abundances of *C. hiranonis*, *Faecalibacterium*, *Turicibacter*, *Escherichia coli*, *Streptococcus*, *Blautia*, and *Fusobacterium* were assessed in feces of six healthy dogs by qPCR following treatment with propidium monoazide (PMA‐qPCR). Conservation methods included lyophilization stored at 4 and −20°C, and freezing with and without glycerol stored at −20°C. In addition, *C. hiranonis* abundance was quantified by culture using Blood Agar at 36°C in anaerobiosis. Repeated measures one‐way ANOVA was used to compare bacterial abundances across conservation methods.

Using PMA‐qPCR, *Faecalibacterium*, *Blautia*, *Fusobacterium*, and *C. hiranonis* were reduced in lyophilized samples kept at 4°C and in frozen samples without glycerol (*P* < .05), as well as *Faecalibacterium* and *Blautia* in lyophilized samples kept at −20°C (*P* < .05). Decreased *C. hiranonis* abundance was identified by culture for all tested conditions (*P* ≤ .0025). After three months, *C. hiranonis* abundance was lower in lyophilized samples kept at 4°C (median[range]: 0 [0–4.48] Log CFU/g) than in samples kept at −20°C (3.81 [2.78–4.91] Log CFU/g). Moreover, its abundance was lower in frozen samples without glycerol (0 [0–3.30] Log CFU/g) than in samples conserved with glycerol (5.65 [4.3–7.2] Log CFU/g).

Although all preservation methods led to a lower *C. hiranonis* abundance compared to fresh samples, the highest bacterial abundance on PMA‐qPCR and culture was observed when freezing samples with glycerol. No differences were observed for glycerol‐preserved samples in PMA‐qPCR for any of the bacteria assessed. While the lyophilization procedure reduced *C. hiranonis* viability, it remained stable in storage at −20°C for three months. Frozen samples without glycerol had decreased abundance of bacteria by PMA‐qPCR, and *C. hiranonis* was undetectable by culture in 5/6 samples after three months.

**Disclosures**

Bruna Correa Lopes, M Katherine Tolbert, Paula R Giaretta, Jan S Suchodolski, and Rachel Pilla are employed by the Gastrointestinal Laboratory at Texas A&M University, which provides assays for intestinal function and microbiota analysis on a fee‐for‐service basis.

## ESCG‐P‐17

### ESCG—European Society of Comparative Gastroenterology

#### Fecal proteolytic activities in dogs with chronic inflammatory enteropathies

##### T Méric^1^, A Kriaa^2^, V Mariaule^2^, A Jablaoui^2^, A Drut^3^, O Sénécat^3^, M Rhimi^2^, J Hernandez^3^

###### ^1^Oniris, Nantes, France; ^2^Microbiota Interaction with Human and Animal team (MIHA), Gif‐sur‐Yvette, France; ^3^ Oniris, Nantes, France

There is increasing evidence for the involvement of host‐microbiota interaction imbalances in canine chronic inflammatory enteropathies (CCIE) pathogenesis. In humans, recent studies have highlighted the role of the host proteases and their respective counterparts from the gut microbiota in the pathogenesis of Inflammatory Bowel Disease (IBD). Indeed, intestinal and fecal samples from patients with IBD have shown significantly increased protease activity compared to healthy controls. Such hyperproteolytic activity is likely to play key roles in a variety of signaling pathways, inflict tissue damage and exacerbate gut inflammation. Over one‐third of known proteases belong to the serine proteases (SPs) family. SPs are physiologically secreted by intestinal cells, immune cells in the lamina propria and the gut microbiota. Fecal proteolytic activity observed in human patients with IBD is mainly due to SPs overactivity (namely, trypsin, neutrophil elastase, proteinase 3 and cathepsin G). We aimed to assess the activity of SPs within faeces and to analyze their distribution in CCIE.

Fifty client‐owned dogs with CCIE and 50 client‐owned healthy dogs participated in the study with owner consent. Diagnosis of CCIE was based on gastro‐intestinal clinical signs of at least 3 weeks’ duration, exclusion of extra‐intestinal, infectious and neoplastic causes, and clinical response to food, antibiotics and/or steroid trials. The healthy dogs were matched in terms of diet and age with the sick animals to limit the impact of these confounding factors. Protease activity was measured in feces by estimating the number of chromogenic/fluorogenic compounds released after proteolytic cleavage. Total proteolytic activity was determined using azocasein as substrate. For protease activity profiling, we used different substrates specific to each targeted protease family. Differences in fecal protease activity between groups were assessed using Kruskal‐Wallis test followed by Dunn's test for multiple comparison.

Total fecal proteolytic activities were significantly (5‐fold) higher in CCIEs compared to healthy controls. Analysis of the increased activity demonstrated that the upregulated proteases belonged to SPs family. Such results were confirmed by the use of specific inhibitors. Functional profiling of the SPs showed that the most increased activities were: elastase (8‐fold, *P* < 0.001), chymase (3‐fold, *P* < 0.001) and trypsin (3.5‐fold, *P* < 0.001).

Upregulation of fecal serine proteolytic activity is confirmed in dogs with CCIE compared to healthy dogs. Such results highlight the key role of the proteolytic imbalance in dogs with CIE, currently poorly studied, and will open new future prospects to validate these results in large multicenter cohorts.

**Disclosures**

Royal Canin funded the study.

**Source of Funding**

Royal Canin funded the study.

## ESCG‐P‐18

### ESCG—European Society of Comparative Gastroenterology

#### Protein‐losing enteropathy in Pugs: a different form of disease?

##### AJ Smith^1^, J Aguiar^2^

###### ^1^Queen Veterinary School Hopsital, Cambridge, UK; ^2^Dick White Referrals, Six Mile Bottom, UK

Protein losing enteropathy (PLE) is a term that refers to diseases of the intestine that lead to protein loss from the gastrointestinal tract. Breed predisposition to PLE has been established in the past with soft coated wheaten terriers suffering a type of familial PLE or German Shepherd dogs having a predisposition to developing inflammatory bowel disease. Pugs have also been identified to respond poorer to treatment of PLE compared to other breeds. In addition, it is authors's anecdotal experience that dogs of this breed, suffering from this condition, have lower serum albumin concentrations than dogs of other breeds. As preliminary work, the aim of this study was to establish if Pugs have significantly lower serum albumin concentrations than dogs of other breeds.

Two hundred Pugs that presented to a referral veterinary centre, for varying reasons and to all specialities, between January 2015 and January 2022, were included in this retrospective study. Pugs were included if they had serum total protein, albumin and globulin concentrations measured on their first visit to the hospital. Two‐hundred dogs of other breeds matched for age, sex and neuter status of the Pugs, presenting to the same hospital departments, were also included. Serum total protein, albumin and globulin concentrations were compared between groups. These measurements were performed in the same commercial laboratory and for the dogs of other breeds run in the same week as the measurements of the matched Pug.Mann‐Whitney tests were used to compare serum total protein, albumin and globulin concentrations between groups. Statistical significance was determined as *P* < 0.05. Results are reported as median [25th, 75th percentiles).

Serum total protein concentrations were significantly different between the two groups (*P* < 0.001), with Pugs having significantly lower concentrations (61 g/L [56, 64]) than other dog breeds (64.5 g/L [61, 68]). Similarly, serum albumin concentrations were significantly lower in the Pug group (*P* < 0.001; 28 g/L [26,30]) compared to other breeds (33 g/L [30, 35]). There was no significant difference in serum globulin concentrations between groups (*P* = 0.2158).

This study suggests that Pugs have a tendency towards lower serum albumin and total protein concentrations when compared to other breeds and this could be associated with a more severe form of disease should they develop protein‐losing enteropathy.

**Disclosures**

No disclosures to report

## ESCG‐P‐19

### ESCG—European Society of Comparative Gastroenterology

#### Mortality rate and associated characteristics prior to hospital discharge in dogs with protein‐losing enteropathy

##### C Hawes, A Kathrani

###### Royal Veterinary College, Hatfield, UK

Protein‐losing enteropathy (PLE) results in death in 50% of affected dogs. However, the rate of mortality in dogs that do not survive to hospital discharge is not well reported. Our objectives were to report the percentage of dogs with PLE that did not survive to hospital discharge, the causes of mortality, and to identify any associated risk factors.

Dogs diagnosed with PLE based on compatible clinical signs, hypoalbuminemia (<26.3 g/L), exclusion of other causes, and inflammatory enteritis, intestinal lymphangiectasia, or both on intestinal histopathology were included. Clinicopathological data, treatment and duration of hospitalization, and cause of death were recorded when available. Statistical analyses were performed using parametric testing and data reported as mean ± standard deviation.

The mortality rate for dogs with PLE prior to hospital discharge was 19.4% (21/108). The most commonly recorded causes of death were aspiration pneumonia (*n* = 5), failure to improve (*n* = 5) and hemorrhagic diarrhoea resulting in anaemia or hypovolaemia (*n* = 3). Twenty‐six dogs had C‐reactive protein (CRP) recorded at least twice during hospitalization; the mortality rate of this cohort was 46% (*n* = 12). A change in CRP concentration between admission to the hospital and 1 to 3 days following initiation of treatment was significantly different between survivors and non‐survivors (−11 ± 40 mg/l, *n* = 8 vs. +28 ± 39.1 mg/l, *n* = 8; *P* value 0.02). Twelve of the 108 dogs included in the study were Pugs and 50% (*n* = 6) of these did not survive to hospital discharge. The cause of death in 5 of these 6 Pugs was recorded as aspiration pneumonia. Pugs that did not survive to hospital discharge were older (9.4 ± 1.5 vs. 5.8 ± 2.2 years, p‐value 0.004) and had higher neutrophil concentrations (23.5 ± 9.4 vs. 14.3± 5.2 × 10e9/l, *P* value 0.032), neutrophil to lymphocyte ratios (24.4± 10.6 vs. 15.2± 6.2, *P* value 0.049), and CRP (50.5±22.6 vs. 17.7±9.3 mg/L, *P* value 0.026) at admission compared to Pugs that did survive to hospital discharge.

Repeat CRP following 1 to 3 days of initiating treatment can help to predict survival to hospital discharge. Pugs were less likely to survive to hospital discharge and this was most commonly due to aspiration pneumonia. Pugs that did not survive to hospital discharge had higher CRP at admission suggesting that subclinical aspiration pneumonia may already be present prior to initiating treatment. Further studies will help to confirm this possibility and therefore whether postponement of glucocorticoids in these dogs could increase survival.

**Disclosures**

Connor Hawes has no disclosures Aarti Kathrani Currently receiving funding from Royal Canin. Previously received funding from Petsavers, PetPlan Charitable Trust, Purina, Waltham and American Association of Veterinary Nutrition.

## ESVC‐P‐1

### ESVC—European Society of Veterinary Cardiology

#### Cilostazol as medical treatment of bradycardia of cardiac origin in dogs

##### G Kiss, S Mørk, F Manczur

###### University of Veterinary Medicine, Budapest, Hungary

In humans and dogs, common cardiac pathological causes for bradycardia are sinus node dysfunction and atrioventricular (AV) blocks. These conditions usually require pacemaker implantation. This is a surgery requiring high competence, expensive equipment and canine patients are often of old age and the summation of these factors causes a smaller motivation for the owner to proceed with the implantation.

Cilostazol‐a phosphodiesterase type 3 inhibitor‐, is a drug originally developed for human patients with symptoms of intermittent claudation in peripheral vascular disease. As a side effect it produces tachycardia. In some studies, it has shown promising effects in patients presenting with pathological bradycardia, both in humans and in dogs, where the use of the drug has increased the heart rate. Despite the clinical success of cilostazol in human studies, there are individual cases only in veterinary literature about it's medical use in bradycardia of cardiac origin.

The aim of this prospective clinical study was to determine the safety, tolerability and clinical effects of cilostazol in severely bradycardic dogs due to cardiac disease. Six dogs ranging from 12 to 15 years was included in the study with clinical signs of bradycardia, all six showed signs of weakness, four was presented with syncope. Three dogs were diagnosed with sick sinus syndrome, one with 2nd degree AV block Mobitz II and two with 3rd degree AV block. Cilostazol was suggested as an alternative treatment option for the owners who refused pacemaker implantation for their dogs. The dogs were given 10 mg/kg cilostazol b.i.d orally. Follow up were made in frame of regular cardiological examinations. Data were evaluated using descriptive statistics and Student's paired *t*‐test. All dogs showed increase in heart rate after administration of the drug. We observed an average of 62% significant increase of the baseline heart rate (*P* = 0.03) and all dogs became asymptomatic with no side effects. The average symptom free survival was 14 months (ranging 5–22 months). However, our study population size is limited, we conclude that cilostazol is clinically effective, safe and well tolerated in bradycardia of cardiac origin in dogs with symptomatic AV block and sick sinus syndrome. Cilostazol may provide an alternative medical treatment when pacemaker implantation is not an affordable option or even to the preoperative hemodynamic stabilization in severe cases before pacemaker implantation.

**Disclosures**

No disclosures to report

## ESVC‐P‐2

### ESVC—European Society of Veterinary Cardiology

#### Smartphone‐based 6‐lead ECG: a new device for electrocardiographic recording in dogs

##### L Alibrandi^1^, T Vezzosi^2^, O Domenech^3^, M Giuntoli^2^, M Croce^3^, G Grosso^2^, R Tognetti^2^

###### ^1^Unit of Translational Critical Care Medicine, Scuola Superiore Sant'Anna, Pisa, Italy; ^2^Department of Veterinary Sciences, University of Pisa, Pisa, Italy; ^3^Department of Cardiology, Anicura Istituto Veterinario Novara, Novara, Italy

Growing research on smartphone‐based technology for electrocardiographic recording has been developed and has become part of the new concept of mobile health both in human and veterinary medicine. Different studies showed the clinical reliability of smartphone‐based ECG for electrocardiographic screening in dogs, but only 1‐lead devices were previously evaluated. Recent studies in humans have begun to evaluate the reliability of smartphone‐based 6‐lead ECG in pediatric settings, on athletes, and in a general cardiology outpatient population. This prospective study assessed the feasibility and the diagnostic reliability of the eKuore 6‐leads ECG, a new smartphone‐based 6‐lead electrocardiograph (smECG), in comparison to standard 6‐lead ECG (stECG) in dogs.

The study included 108 dogs owned by clients. All included subjects underwent simultaneous electrocardiographic recording with both methods (stECG and smECG) in right lateral recumbency for at least 30 seconds. All ECG traces were reviewed blindly by an experienced operator, who judged whether the traces were acceptable for interpretation, performed electrocardiographic measurements, and assigned a diagnosis. Agreement in electrocardiographic interpretation and diagnosis between smECG and stECG was assessed using the Bland‐Altman test and the Cohen's k test.

Electrocardiographic tracings obtained with smECG were interpretable in 100% of cases. Perfect agreement between smECG and stECG was found in the detection of sinus rhythm, atrial fibrillation, ventricular arrhythmias, atrioventricular blocks, and branch blocks (*k* = 1). No clinically relevant differences were found in the assessment of heart rate (bias, 2 bpm), P wave duration (bias, −2 ms), PQ interval duration (bias, −3 ms), QRS complex duration (bias, −3 ms) and QT interval duration (bias, −7 ms). Differently, the smECG showed an underestimation of the amplitude of P waves (bias, 0.1 mV) and R waves (bias, 1 mV) of possible clinical relevance. However, P waves were clearly visible on all smECG tracings with sinus rhythm. The underestimation could result from a technical difference in the fixed low‐pass filter of the smECG device (40 Hz) compared with that of the stECG used in the present study (100 Hz). Our study suggests that the tested smECG is a clinically reliable device for assessing heart rate and rhythm in dogs but may underestimate the amplitude of electrocardiographic waves. The device could be a new diagnostic tool for arrhythmia detection in dogs, particularly useful for telemedicine and mobile health thanks to the easy‐to‐use smartphone‐based system.

**Disclosures**

No disclosures to report

## ESVC‐P‐3

### ESVC—European Society of Veterinary Cardiology

#### Correlation of serum N‐terminal pro B‐type natriuretic peptide with pulmonary hypertension in heartworm‐infected dogs during adulticide treatment

##### N Costa‐Rodríguez, Y Falcón Cordón, S Garcia Rodríguez, S Falcón Cordón, J Matos, J Montoya Alonso, E Carretón Gómez

###### Research Institute of Biomedical and Health Sciences (IUIBS), Las Palmas de Gran Canaria, Spain;

In veterinary medicine, natriuretic peptides, including NT‐proBNP, are important tools in the diagnosis and therapeutic monitoring of cardiac diseases. Increased concentrations of NT‐proBNP have been described in dogs with post‐capillary pulmonary hypertension (PH) due to left heart failure. Moreover, NT‐proBNP concentrations are increased in humans and dogs with precapillary PH, and has been validated to determine severity, monitor response to treatment and as a prognostic indicator. PH of precapillary origin is a frequent and severe consequence of heartworm disease (*Dirofilaria immitis)*. Therefore, the aim was to evaluate NT‐proBNP concentrations in 50 dogs with heartworm throughout adulticide treatment following the recommended protocol and 6 months after finishing (270 days). Parasite load and presence/absence of PH were echocardiographically assessed, following the ACVIM consensus statement guidelines; presence/absence of microfilariae was also determined. NT‐proBNP levels were measured by using the VCHECK V200 Veterinary Immunoassay Analyzer (Bionote, USA). The cut‐off value for healthy dogs was established by the manufacturer as 900 pmol/L. At the beginning of the adulticide treatment (day 0), 40% of the dogs showed PH, 50% showed high parasite burden and 44% were microfilaremic. Mean NT‐proBNP concentrations in dogs with PH were 3 times higher than in normotense dogs, being within reference values in the latter (2004.4 ± 467.6 pmol/L vs. 689.9 ± 257.1 pmol/L). No significant differences were observed in NT‐proBNP results regarding parasite load and microfilaremia. Most dogs without PH maintained NT‐proBNP concentrations within the reference ranges throughout the treatment, showing significant increases during the death of the adult parasites on days 60 and 90 (*P* < 0.05) (day 30: 683.9 ± 105.9 pmol/L; day 60: 851.8±168.6 pmol/L; day 90: 936.1 ± 189.2 pmol/L, day 270: 699.9 ± 96.0 pmol/L). Dogs with PH showed a significant reduction in NT‐proBNP concentrations on day 30 (947.2±388.3 pmol/L) (*P* = 0.022), while the values remain on day 60 (1402.4 ± 360.0 pmol/L) (*P* = 0.212) and on day 90 (939.6 ± 215.5 pmol/L) (*P* > 0.05). On day 270, the values were within physiological ranges (592.8 ± 102.4 pmol/L), significantly lower than on day 0 (*P* = 0.001). The measurement of NT‐proBNP concentrations seems to be useful in the determination of presence of HP in dogs with heartworm, being consistent with the results reported by other authors in dogs with precapillary HP caused by other pathologies of diverse origin. Moreover, NT‐proBNP seems to be useful in the determination and monitoring of heartworm‐infected dogs undergoing adulticide treatment.

**Disclosures**

No disclosures to report

## ESVC‐P‐4

### ESVC—European Society of Veterinary Cardiology

#### The good, the bad and the breeder—Development of genetic HCM‐variants in cat populations from routine testing in the last 10 years

##### L Kempker^1^, AUP Aupperle‐Lellbach^1^, POC Pocknell^1^, AV Van de Weyer^1^, KK Kempker^2^, CB Beitzinger^1^

###### ^1^Laboklin GmbH & Co. KG, Bad Kissingen, Germany; ^2^Tierärztliche Gemeinschaftspraxis Dres. Fischer & Dr. Kempker, Niederwerrn, Germany

Primary hypertrophic cardiomyopathy (HCM) is the most common heart disease in cats, characterized by left ventricular and/or septal hypertrophy in the absence of systemic or other cardiac diseases. Feline HCM is a multifactorial disease with a genetic component. An autosomal dominant single nucleotide variation (SNV) of the MYBPC3 gene (HCM1) in Maine Coons, an autosomal dominant SNV in the MYBPC3 gene (HCM3) in Ragdolls, and an autosomal dominant SNV in the ALMS1 gene (HCM4) in Sphynx have been identified.

The aim of this study was to investigate the allele frequency of the genetic variants HCM1, HCM3 and HCM4 in routine diagnostic samples from cats with a special view on dynamic development over the years.

From January 2012 to June 2022, blood samples or cheek swabs from 28,441 cats (female: 16,078, male: 12,363) were submitted to Laboklin for genetic testing (HCM1, 3, 4). Clinical findings were not available. Genetic analyses included PCR, direct sequencing and/or real‐time PCR. The results were three possible genotypes (N/N; N/mut; mut/mut). The genotype frequency of the variant was calculated retrospectively and the development over the years 2012 to 2022 was analyzed.

In Maine Coons (*n* = 23,309), the causative genetic variant HCM1 was distributed as follows: 79.8% free (N/N); 19.5% carrier (N/HCM1); 0.7% affected (HCM1/HCM1). The genotype frequency decreased from 39.7% to 12.4% during the years 2012 to 2022. Samples from 4660 Ragdoll cats showed the following genotypes: 93.9% HCM3‐free (N/N); 5.9% carrier (N/HCM3); 0.2% affected (HCM3/HCM3). Genotype frequency decreased from 28.6% to 3.1% (2012 to 2022). Between 2021 and 2022, samples from 242 Sphynx were examined. Genotype distribution: 33.8% free (N/N); 44.2% carrier (N/HCM4); 21.9% affected (HCM4/ HCM4); genotype frequency of HCM4: 44%.

In summary, this is the first study describing the genotype frequencies of genetic variants for feline HCM1 and HCM3 over an extended time period. In Maine Coon and Ragdoll cats, breeding successes in eliminating the risk alleles for primary HCM could be demonstrated. The genetic test for HCM4 in Sphynx has been available for only 2 years, thus development is not obvious at the moment, but it is hoped that similar effects will be observed in the future. Nevertheless, primary HCM is a multifactorial process, depending on the breed and other (unknown) factors, which results in a high variability of the clinical expression of the disease.

**Disclosures**

Lena Kempker, Heike Aupperle‐Lellbach, Annabella Pocknell, Anna‐Lena Van de Weyer, and Christoph Beitzinger are employed by Laboklin GmbH & Co. KG. The test evaluated in the study is commercially offered by Laboklin GmbH & Co. KG. Karsten Kempker is the husband of the author.

## ESVC‐P‐6

### ESVC—European Society of Veterinary Cardiology

#### Assessment of precordial R‐peak time in dogs with left sided volume overload secondary to myxomatous mitral valve disease and dilated cardiomyopathy

##### RA Baisan, CA Turcu, LM Bilboc, MC Maftei, V Vulpe

###### Iasi University of Life Sciences “Ion Ionescu de la Brad”, Iasi, Romania

R peak‐time (RPT) is an electrocardiographic index measuring the time of wave spread from the ventricular endocardium to epicardium. In human medicine, RPT is used as a diagnostic criterion in both left and right ventricle volume overload and hypertrophy as well as for assessing the interventricular dyssynchrony. Recently, reference ranges of RPT in healthy dogs were published, based on their morphometric body type. However, to date, there is no data regarding the utility of RPT in dogs with left ventricle (LV) volume overload secondary to myxomatous mitral valve disease (MMVD) or dilated cardiomyopathy (DCM). We hypothesized that dogs with LV volume overload would have delayed depolarization time on left precordial leads compared to healthy dogs. Therefore, the aim of the study was to compare RPT in healthy dogs and dogs diagnosed with clinical MMVD and DCM.

This retrospective study included dogs between October 2020 and December 2022, subjected to a complete cardiologic examination consisting of clinical examination, 5 min 12‐lead electrocardiography, echocardiography and thoracic radiography. Dogs were grouped according to their diagnosis into MMVD and DCM. Healthy dogs examined for annual cardiologic screening or prior to anesthesia were included and divided into healthy large (HL) and healthy small (HS). RPT was measured in precordial leads (V1–V6) and averaged from five consecutive complexes as the time from the QRS onset to the time of R or R' peak.

Sixty‐six dogs were included in the study and were divided into DCM Group (*n* = 25), MMVD Group (*n* = 19), HL Group (*n* = 11) and HS Group (*n* = 11). There were no statistically differences in BW or age between HL and DCM, while dogs in HS were younger compared to MMVD Group (*P* < 0.05). There was no statistically difference in V1 lead RPT between HL and DCM (*P* = 0.73) while dogs in DCM had significantly larger RPT in V2‐V5 compared to HL (*P* < 0.01). Similarly, no difference was found in lead V1 RPT between HS and MMVD (*P* = 0.51), while RPT in V2‐V6 was significantly larger in dogs with MMVD compared to HS Group (*P* < 0.05). Moreover, RPT in all leads revealed significantly larger values in DCM Group compared to MMVD Group (*P* < 0.01).

This study showed that left ventricle depolarization time is prolonged in dogs with left ventricle volume overload secondary to DCM or MMVD. Moreover, DCM dogs revealed even longer depolarization time compared to MMVD dogs. These results may be useful as an electrocardiographic diagnostic criterion for LV volume overload in dogs.

**Disclosures**

No disclosures to report

## ESVC‐P‐7

### ESVC—European Society of Veterinary Cardiology

#### Inflammatory and immune variables as predictors of survival in dogs with myxomatous mitral valve disease

##### M Cimerman^1^, N Druzhaeva^2^, A Nemec Svete^1^, M Hajdinjak^3^, K Pohar^4^, A Ihan^4^, A Domanjko Petrič^1^

###### ^1^Small Animal Clinic, Veterinary faculty, University of Ljubljana, Slovenia; ^2^Olympus Cove Veterinary Clinic, Salt Lake City, Utah, USA; ^3^Laboratory of Applied Mathematics and Statistics, Faculty of Electrical Engineering, Slovenia; ^4^ Institute of Microbiology and Immunology, Faculty of Medicine, University of Ljubljana, Slovenia

Human and canine studies have shown that peripheral blood inflammatory and immune markers are elevated in heart failure. Human studies suggest that elevated levels of inflammatory cells are associated with poor outcome in patients with heart failure.

The aim of our study was to investigate the association of inflammatory and immune variables with prognosis and survival in dogs at different stages of myxomatous mitral valve disease (MMVD).

Sixty‐two client‐owned dogs with MMVD were included in the study and divided into three groups according to the American College of Veterinary Internal Medicine (ACVIM) classification. Nineteen dogs were asymptomatic (ACVIM B2), twenty‐four with stable heart failure (ACVIM C), and nineteen with unstable heart failure (ACVIM C and D). For each of the three groups and for all patients we performed univariate Cox proportional hazards regression analysis to test the following covariates: peripheral white blood cell count, C‐reactive protein (CRP) and cardiac troponin I (cTnI) concentrations, as well as concentrations and percentages of T lymphocytes (CD3+) and their subtypes (T helper cells (CD3+CD4+), cytotoxic T cells (CD3+CD8+), double positive T cells (DPT; CD3+CD4+CD8+), and double negative T cells (DNT; CD3+CD4‐CD8‐)), and B lymphocytes (CD45+CD21+). P values <0.1 in individual groups and *P* values <0.05 in the group of all patients were considered significant.

In the ACVIM B2 group higher DPT percentage was negatively associated with survival (HR 2.5455; *P* = 0.0512), in stable ACVIM C group higher cTnI concentration (HR 9.497; *P* = 0.0609) and in unstable ACVIM C and D group, higher concentrations of cTnI (HR 16.428; *P* = 0.0691), CD3+ (HR 2.4515; *P* = 0.0849), CD3+CD8+ (HR 2.9347; *P* = 0.0484), DPT (HR 1.021e+27; *P* = 0.0419) and DNT (HR 26.580; *P* = 0.0963) were all negatively associated with survival. Whereas higher percentage of CD3+CD4+ (HR 0.96044; *P* = 0.0527) in unstable ACVIM C and D had a positive impact on survival. In the group of all patients, higher concentrations of cTnI (HR 19.6638; *P* < 0.0001) and CD3+CD8+ (HR 3.9313; *P* = 0.0073) were negative predictors of survival and higher percentage of CD3+CD4+ (HR 0.97331; *P* = 0.0437) was a positive predictor of survival.

In conclusion, the study showed that selected inflammatory and immune variables may be predictors of survival in MMVD, especially in the unstable ACVIM C and D group and when all patients are considered as one group.

**Disclosures**

No disclosures to report

**Source of Funding**

Slovenian Research Agency ARRS (P4‐0053)

## ESVC‐P‐8

### ESVC—European Society of Veterinary Cardiology

#### Clinical findings and outcome in 27 cats with transient myocardial thickening

##### G Romito^1^, C Guglielmini^2^, H Poser^2^, C Valente^2^, C Mazzoldi^1^, P Castagna^3^, M Cipone^1^

###### ^1^University of Bologna, Ozzano dell'Emilia, Italy; ^2^University of Padova, Legnaro (PD), Italy; ^3^Freelance Veterinary Cardiologist, Bologna, Italy

Transient myocardial thickening (TMT) is a reversible left ventricular wall (LVW) thickening mimicking primary hypertrophic cardiomyopathy in cats. A correct assessment of TMT cases is essential to avoid misdiagnosis and improper medical decisions. To date, only few papers described the clinical features of feline TMT. Therefore, the aim of this study was to provide further demographic and clinical data on feline TMT.

For the purposes of this retrospective, multicenter cohort study, cats diagnosed with TMT were searched in the medical databases of three veterinary hospitals. Transient myocardial thickening was defined for cats with at least two echocardiographic examinations showing an increase in end‐diastolic LVW thickness (i.e., ≥6 mm) at presentation and subsequent echocardiographic normalization (i.e., end‐diastolic LVW thickness <5.5 mm). Signalment, history, clinical, laboratory, radiographic, electrocardiographic, echocardiographic, treatment and outcome data were retrieved for each cat.

Twenty‐seven cats were included, 16 males (59.3%) and 11 females (40.7%). The median age and body weight were 3 (range: 0.5–14) years and 4.4 (2.3–9) kg, respectively. In nine cats (33.3%) an antecedent event was documented (spaying/neutering [*n* = 7]; abscess [*n* = 2]) occurring within 7 days before presentation. Twenty‐five cats (92.6%) had congestive heart failure (CHF), including: lung edema (LE; *n* = 10); LE and pleural effusion (Pl‐Ef; *n* = 8); pericardial effusion (PE‐Ef; *n* = 3); LE, Pl‐Ef and PE‐Ef (*n* = 2); LE and PE‐Ef (*n* = 1); and LE and abdominal effusion (*n* = 1). Additional signs at admission included hypothermia (*n* = 13), hypotension (*n* = 8) and bradycardia (*n* = 7). At admission, cardiac troponin I was measured in all cats; in each case it was increased (median value 3.1 ng/mL [0.21–25ng/mL]). Seven cats (25.9%) showed electrocardiographic abnormalities. On echocardiography, the median value of the thickest end‐diastolic LVW segment was 6.7 (6–8.8) mm. One cat also showed cardiac thrombosis. The cause of myocardial injury was identified in 14 cats (51.9%): spaying/neutering complications (*n* = 7), toxpoplasmosis (*n* = 3), bartonellosis (*n* = 2), and sepsis (*n* = 2). All cats with CHF were hospitalized (median duration 4 [2–9] days) and received cardiac treatment; cats with toxoplasmosis, bartonellosis and sepsis also received antimicrobial treatment. Four cats showed relapses of CHF. All cats survived; the median time from diagnosis to echocardiographic normalization was 41 days (5–260 days).

Cats affected by TMT are usually young animals with acute myocardial injury, CHF and clinical signs of cardiogenic shock at admission. An antecedent predisposing event or an ongoing infectious disease can be diagnosed in many cats. The prognosis is good, and echocardiographic normalization usually occurs shortly after the prescription of appropriate treatment.

**Disclosures**

No disclosures to report

## ESVC‐P‐9

### ESVC—European Society of Veterinary Cardiology

#### Associations between thoracic radiographic changes and severity of pulmonary hypertension diagnosed via Doppler echocardiography in dogs with heartworm disease (Dirofilaria immitis)

##### S Falcón Cordón^1^, Y Falcón Cordón^1^, A Caro Vadillo^2^, M Montoya Alonso^1^, E Carretón Gomez^1^

###### ^1^Internal Medicine, Faculty of Veterinary Medicine, Research Institute of Biomedical and Health Science (IUIBS), University of Las Palmas de Gran Canaria, Las Palmas de Gran Canaria, Spain.; ^2^Internal Medicine and Animal Surgery, Faculty of Veterinary Medicine, University Complutense of Madrid, Madrid, Spain.

Pulmonary hypertension (PH) is a common finding in dogs with heartworm (*Dirofilaria immitis*), usually diagnosed by using transthoracic Doppler echocardiography. However, this method is not always available for veterinary clinicians or economically available to animal owners. Radiographic findings are very useful in determining the severity of this pathology, but the correlation of these changes with the presence or absence of PH has never been evaluated. The objective was to determine if specific radiographic findings are associated with the presence and severity of PH in 62 dogs with heartworm. To this aim, the studied animals underwent an echocardiographic exam to determine the presence/absence of PH following the ACVIM consensus statement guidelines, and parasite load was also determined. Thoracic radiographs (right laterolateral and dorso‐ventral views) were taken, and vertebral heart score (VHS), ratio of the right cranial pulmonary artery to the fourth rib (PA:4r) and ratio of the right caudal pulmonary artery at the ninth rib level (PA:9r) were calculated. Presence/absence of microfilaremia was determined as well. PH was present in 51.6% of the dogs. Furthermore, 12.9% showed high parasite burden and 40.3% showed microfilaremia. The results showed significantly increased VHS (10.41±0.81 vs. 9.72±0.81), PA:4r (1.37±0.44 vs. 1.05±0.23) and PA:9r (1.64±0.56 vs. 1.16±0.27) ratios in dogs with PH when compared with normotensive dogs (*P* < 0.001). No significant differences were found in the radiological measurements based on the parasite load, and regarding the presence/absence of MF, only significant differences were found in the PA:9r ratios (1.46±0.36 vs. 1.37±0.59, respectively) (*P* = 0.031). The results showed that cardiac enlargement, as well as enlargement and tortuosity of the pulmonary arteries, was associated with presence of PH echocardiographically determined, suggesting that specific radiographic findings can be used to help determine the presence or absence of PH in dogs with heartworm. This may help guide the determination of the severity of this pathology and guide the need to perform further specific diagnostic tests.

**Disclosures**

No disclosures to report

## ESVC‐P‐10

### ESVC—European Society of Veterinary Cardiology

#### Clinical presentation and survival in dogs with acquired atrial septal defect secondary to myxomatous mitral valve disease

##### K Georgiadis^1^, W Davis^2^, H Hodgkiss‐Geere^2^, J Dukes‐McEwan^2^, S Sudunagunta^3^, C Linney^3^, V Palermo^1^

###### ^1^Anderson Moores Veterinary Specialists, UK

^2^Small Animal Teaching Hospital, University of Liverpool, UK

^3^Willows Veterinary Centre & Referral Service, UK

Rupture of the left atrial wall is a rare consequence of advanced myxomatous mitral valve disease (MMVD) in dogs. The aetiology of spontaneous endocardial splitting secondary to mitral insufficiency in dogs is likely related to increased left atrial volume/pressure and mechanical trauma to the endocardium induced by the regurgitant jet. Most atrial tears occur in the caudal wall of the left atrium causing haemopericardium, however the fossa ovalis may represent a site of anatomical weakness. In contrast to rupture of the caudal wall, rupture of the atrial septum may offer a haemodynamic advantage to a dog with elevated left atrial pressure by providing a low pressure “sink” into which the left atrium can unload. The subsequent left‐to‐right shunting of blood causes volume overload to the right side of the heart and may lead to right‐sided congestive heart failure (R‐CHF). R‐CHF is rarely an acute life‐threatening condition and can be managed palliatively.

The aim of this study was to evaluate the clinical presentation and survival time in dogs with an atrial septal defect (ASD) secondary to MMVD. This condition has been rarely reported in the literature and outcome data are lacking.

Seven dogs with acquired ASD secondary to MMVD were included in this retrospective multicenter study. Diagnosis of acquired ASD was based on echocardiography. All seven dogs were previously diagnosed with MMVD, classified as ACVIM stage B2 (1/7), ACVIM stage C (3/7) and ACVIM stage D (3/7). Breeds included CKCS (5 dogs), Chihuahua (1) and Griffon (1). Mean age of the dogs was 9 yo and mean weight was 8.3kg. R‐CHF was a common presenting sign (5 dogs), together with left‐sided CHF (L‐CHF) (4 dogs). The mean LA major, LA:Ao and LVIDdN were 49.70 mm, 2.79 and 2.3, respectively. All dogs had evidence of pulmonary hypertension (PH). All dogs were treated with a combination of furosemide, pimobendan, benazepril and spironolactone, 2 dogs received hydrochlorothiazide and 1 received diltiazem.

Survival times varied from 1 to 340 days, with 2 dogs surviving more than 193 days and 1 dog still alive at the time of writing (63 days since diagnosis). Although the prognosis is often considered poor, the results of this study show that dogs presenting with acquired ASD secondary to MMVD might have a variable outcome which might be better when compared with dogs that present with atrial tears in the caudal wall of the left atrium.

**Disclosures**

No disclosures to report

## ESVC‐P‐11

### ESVC—European Society of Veterinary Cardiology

#### Echocardiographic classification of dogs with aortic stenosis: potential utility of a novel staging system

##### W Davis^1^, A Francis^2^, K Borgeat^3^

###### ^1^University of Liverpool, Neston, UK; ^2^South Coast Cardiology, Eastleigh, UK; ^3^Langford Vets, Bristol, UK

Severity of aortic stenosis (AS) in humans is classified using a staging system which considers the extent of global cardiac damage. Currently, classification of canine AS is based on trans‐aortic pressure gradient (PG) alone. We hypothesised that a broader system of classification would be useful in dogs with AS.

This study aimed to retrospectively classify dogs with AS based on an adapted human staging system, exploring feasibility of classification and the association between stage and clinical features such as PG and clinical signs.

Aortic stenosis was diagnosed in 183 dogs, and 87 dogs met inclusion criteria. Clinical and echocardiographic data were retrieved from computerised records. Dogs were classified into three groups, based on the human staging system (stage 0, 1, 2+). Descriptive statistics were explored. Differences in signalment, PG and clinical signs between groups were evaluated using appropriate statistical tests. Significance was set at *P* < 0.05, corrected for multiple comparisons.

No difference in age, weight, sex or breed was identified. Dogs classified as stage 2+ were more likely to have clinical signs than those in stages 0 or 1 (50% vs. 7% [*P* = 0.004] and 17% [*P* = 0.043] respectively) and had a higher PG than dogs in stage 0 (90 mmHg [35–143] vs. 25 mmHg [18–182], *P* = 0.001). No difference in clinical signs was present between stage 0 and 1, nor was a difference in PG identified between stages 1 and 2.

Results support future validation of a staging system for AS in dogs based on a broader spectrum of echocardiographic data than PG alone.

**Disclosures**

No disclosures to report

## ESVC‐P‐12

### ESVC—European Society of Veterinary Cardiology

#### Serum proteomic profiles in dogs affected with pulmonary hypertension secondary to degenerative mitral valve disease

##### S Sakarin^1^, S Surachetpong^1^, A Rungsipipat^1^, S Roytrakul^2^, J Jaresitthikunchai^2^, N Phaonakrop^2^, S Charoenlappa^2^, S Thaisakun^2^

###### ^1^Chulalongkorn University, Bangkok, Thailand; ^2^National Center for Genetic Engineering and Biotechnology (BIOTEC), National Sci Pathum Thani, Thailand

Pulmonary hypertension (PH), which refers to abnormally increased pressure in the pulmonary arteries, is frequently observed in dogs with degenerative mitral valve disease (DMVD). Differentiating between DMVD dogs with and without PH is challenging due to the nonspecific clinical signs and the need for expensive ultrasound machines. Proteomic techniques are valuable for identifying the proteins that could serve as biomarkers in several diseases.

The aim of this study was to identify serum proteins that were uniquely expressed in the DMVD dogs with PH and could be used as biomarkers for the diagnosis of PH secondary to DMVD in dogs.

Serum samples were collected from 81 elder small‐breed dogs. All dogs were classified into 3 groups, including the control (*n* = 28), DMVD (*n* = 24), and DMVD+PH (*n* = 29) groups. Liquid chromatography tandem‐mass spectrometry (LC‐MS/MS) was used for quantitative proteomic analysis.

Only 48 proteins were expressed in the DMVD+PH group. Among these, U2AF homology motif kinase 1 (UHMK1), fibroblast growth factor 13 (FGF13) and ribosomal protein L24 (RPL24) were found to associate with the pathogenesis of PH. These proteins could be potentially served as candidate biomarkers for diagnosis PH in DMVD dogs.

This study highlights the potential use of shotgun proteomic techniques for identifying candidate biomarkers in PH secondary to DMVD in dogs. The results from this study may benefit for the future research in developing alternative diagnostic or prognostic biomarkers for PH in dogs with DMVD.

**Disclosures**

No disclosures to report

**Source of Funding**

This study was supported by the 90th Anniversary of Chulalongkorn University Fund (Ratchadaphiseksomphot Endowment Fund) and the 100th Anniversary Chulalongkorn University Fund for Doctoral Scholarship.

## ESVC‐P‐13

### ESVC—European Society of Veterinary Cardiology

#### The impact of intravenous medetomidine and vatinoxan on echocardiographic evaluation of dogs with ACVIM stage B1 mitral valve disease

##### HE Välimäki^1^, H Leppänen^2^, H Turunen^3^, M Raekallio^2^, JM Honkavaara^2^

###### ^1^Evidensia Animal Hospital Tammisto, Vantaa, Finland; ^2^University of Helsinki, Helsinki, Finland; ^3^Vetcare Ltd, Mäntsälä, Finland

Alpha_2_‐adrenoceptor agonists, such as medetomidine and dexmedetomidine, are commonly used in clinical practice to induce a dose‐dependent, reliable, and reversible sedation in dogs. The peripheral effects of alpha_2_‐agonists, however, impair the function of the canine cardiovascular system by increasing vascular resistance and decreasing heart rate and cardiac output. The peripherally acting alpha_2_‐adrenoceptor antagonist, vatinoxan, is a novel solution to prevent these undesirable peripheral effects of (dex)medetomidine, while the central effects (i.e., sedation) remain unaffected.

Our aim was to investigate the sedative, hemodynamic and echocardiographic effects of intravenous medetomidine and vatinoxan in dogs with ACVIM stage B1 mitral valve disease. We hypothesized that medetomidine‐vatinoxan would provide a clinically useful level of sedation, without untoward cardiovascular effects or echocardiographic changes.

Twelve client‐owned dogs with ACVIM stage B1 mitral valve disease were included in the study. A transthoracic echocardiographic examination was performed, and vital parameters recorded before and 10 min (T10) after sedation with intravenous medetomidine (10 μg/kg) and vatinoxan (200 μg/kg). Sedation was assessed subjectively. Parametric data were analyzed with Student's *t*‐tests with an alpha‐level of <0.05.

When compared to baseline, normalized end‐systolic volume and left ventricular systolic diameter increased significantly at T10 from 0.89 ± 0.19 to 1.13 ± 0.29 mL/kg (mean ± standard deviation) and 0.96 ± 0.12 to 1.10 ± 0.10 cm, respectively. Ejection fraction (EF from 66.33 ± 4.0 to 56.23 ± 9.54 %) and fractional shortening (FS from 36.13 ± 5.42 to 27.24 ± 5.6 %) decreased significantly. No significant changes were observed in normalized end diastolic volume, left ventricular diastolic diameter or left atrial size, while aortic (from 1.34 ± 0.2 to 0.99 ± 0.14 m/s) and pulmonic (from 0.94 ± 0.16 to 0.66 ± 0.15 m/s) velocities decreased significantly. All dogs remained normotensive whereas heart rates decreased significantly from baseline (114 ± 22 to 82 ± 22 beats/minute).

Sedation was suitable for performing echocardiography without manual restraint. Echocardiographic parameters were not fully comparable with baseline values, which should be taken into consideration when evaluating echocardiographic results in dogs sedated with intravenous medetomidine‐vatinoxan. No adverse effects were observed with the dose studied.

**Disclosures**

Dr. Turunen works as an employee for Vetcare Ltd (Mäntsälä, Finland), which produces the veterinary medicinal product that was the subject of this study. Dr Honkavaara has received lecture fees from Vetcare Ltd as well as financial support for his previous studies with vatinoxan. Dr Raekallio has received financial support from Vetcare Ltd for her previous studies with vatinoxan.

**Source of Funding**

The Finnish Foundation of Veterinary Research and Evidensia Research Fund supported financially, Vetcare Ltd provided medicinal products studied as well as anesthesia monitors

## ESVC‐P‐14

### ESVC—European Society of Veterinary Cardiology

#### Percutaneous closure of patent ductus arteriosus with the Vet‐PDA Occluder device in dog

##### AJ Santana^2^, D Saavedra^2^, MJ Perdigón^2^, JI Matos^3^, SN García‐Rodríguez^1^, JA Montoya‐Alonso^1^

###### ^1^Internal Medicine, Faculty of Veterinary Medicine, Research Institute of Biomedi, Las Palmas de Gran Canaria, Spain; ^2^Anicura Albea veterinary hospital, Las Palmas de Gran Canaria, Spain, Las Palmas de Gran Canaria, Spain; ^3^Internal Medicine, Faculty of Veterinary Medicine, Research Institute of Biomedi, Arucas, Spain

The development of safe devices and protocols for the resolution of patent ductus arteriosus (PDA) is of the utmost importance in veterinary practice. The great diversity of sizes and morphologies of the PDA, moreover, the variety of body conditions in the canine species, make it difficult to determine the most appropriate occlusion device. The Vet‐PDA Occluder (VOP) (Evomed‐Vetcare SL, Madrid, Spain) device consists of a series of nitinol coils, conical in shape, occluding vascular communication when released. The use of homologous devices Nit‐Occlud PDA (PFM‐Medical AG Colonia, Germany) has previously demonstrated its utility for the adequate resolution of PDA in human medicine. The aim of this study is to describe for the first time the use of the VPO device in the successful resolution of PDA in 5 dogs.

The selected animals had <6 months of age, weighing <2 kg. The mean diameters of the ductal ampulla were 3.3 ± 0.7 mm, and those of the ductal ostium were 1.8 ± 0.2 mm. PDA morphology was previously classified as type IIB in 4/5 of the animals and type I in 1/5. No animal presented signs of congestive heart failure. The same anesthetic protocol was applied in all cases.

The conducted protocol consisted in obtaining percutaneous vascular access to the right jugular vein (5–6 Fr) by using a modified Seldinger technique and passing a guidewire and multipurpose catheter in a retrograde manner through the PDA, which allowed for manual measurement of the canal by angiography. Device sizing was based on the distal coil diameter, no more than 2 mm greater than the ampulla, and on the proximal coil diameter, no more than 3–4 mm greater than the ostium diameter. The occlusion device and delivery system were loaded onto a dedicated guide catheter (4–5 Fr) and, under fluoroscopy, the device was placed by performing continuous loops within the ductal ampulla and on the pulmonary side of the duct. The safe position of the device was determined by gentle manipulation and transthoracic echocardiography. Echocardiographic presence of residual flow was no reported. The endovascular material was removed, and hemostasis was achieved by applying digital pressure for ten minutes. No significant complications were reported during the surgical intervention and the time range for its performance was 34–64 min.

The VPO Closure Device has proven effective for ductal occlusion in all studied animals, and the protocol performed has been a safe surgical alternative in this group of small patients.

**Disclosures**

No disclosures to report

## ESVC‐P‐15

### ESVC—European Society of Veterinary Cardiology

#### How do practicing veterinarians treat stage B and C myxomatous mitral valve degeneration in dogs?

##### V Szatmári, E Muis, M van Staveren Utrecht University, Utrecht, Netherlands

###### In recent years, several clinical trials on medical management of myxomatous mitral valve degeneration were published, in addition to the newest edition of the ACVIM Consensus Guidelines in 2019. The present study aimed to investigate how first opinion veterinary practitioners integrate these study results in their daily practice.

Digital questionnaires with multiple choice and open questions were sent out to veterinary practices via newsletters of various professional organizations. Participation was voluntary. Only the 363 fully completed questionnaires were analyzed of the 524 responses.

For stage B1 dogs, 7% of the respondents would recommend life‐long medical therapy, of which 93% would be pimobendan. The remaining 93% would recommend no therapy for this stage.

For stage B2 dogs, 84% of the respondents would recommend life‐long medical therapy, of which 98% would prescribe pimobendan, 11% would add a loop diuretic, 4% an ACE‐inhibitor and 4% spironolactone. Sixteen percent would not treat dogs in stage B2.

For stage C, the respondents were asked to describe their best practice, if the owner reports no financial concerns. Seventeen different combinations of medications were listed with the most common ones: pimobendan and furosemide (35%), pimobendan and torasemide (13%), pimobendan, furosemide and torasemide (9%), an ACE‐inhibitor, pimobendan and furosemide (9%), furosemide and torasemide (4%), an ACE‐inhibitor, pimobendan, spironolacton and furosemide (3%), and pimobendan, spironolactone and furosemide (3%).

Based on the results of this survey, close to 90% of respondents successfully apply the clinical trial results for treating dogs in stage B1 and B2. However, regarding treatment of dogs in stage C, more diversity was observed. About half of the respondents prescribe the evidence‐based combination of pimobendan with a loop diuretic. An interesting finding is that almost a quart of the respondents prescribe two loop diuretics simultaneously.

**Disclosures**

No disclosures to report

## ESVC‐P‐16

### ESVC—European Society of Veterinary Cardiology

#### Ratio of tricuspid annular plane systolic excursion on systolic pulmonary arterial pressure as a prognostic factor in canine pre‐capillary pulmonary hypertension

##### E Van Renterghem, MLegrand, M Lekane, E Roels, K Gommeren, AC Merveille

###### University of Liège, Liège, Belgium

Pre‐capillary pulmonary hypertension (prePH) can cause canine right‐sided heart failure. Right‐ventricular pulmonary arterial (RVPA) coupling, measured by invasive pressures, offers prognostic information in humans. Tricuspid annular plane systolic excursion (TAPSE) is an index of right ventricular (RV) systolic function in dogs. The ratio of TAPSE and systolic pulmonary artery pressure estimated by tricuspid regurgitation (TRPG) (TAPSE/sPAP), although a validated non‐invasive surrogate of RVPA coupling in human medicine, is not described in dogs. This study describes bodyweight‐independent TAPSE/sPAP in dogs with prePH, its correlation with RV function and size indices, and prognosis.

This retrospective observational study included dogs with prePH, dividing them into three classes according to TRPG (mild: 31–50; moderate: 51–75, severe: >75 mmHg). Dogs were classified into categories based on clinical signs of heart failure (HF) or not. RV size, wall thickness and systolic function, right atrial size, left ventricular shape and left atrial size were recorded. For TAPSE/sPAP, we tested 4 different combinations of bodyweight‐independent TAPSE (nTAPSE or TAPSE/Ao) and TR measurements (mmHg or m/s). Survival data were obtained from the dog's record or by telephone interview. Cardio‐pulmonary death (CPD) was defined as death or euthanasia attributable to cardio‐pulmonary disease. One‐month and long‐term survival were calculated. Univariate and multivariate logistic regression models including HF, age, etiology, prePH severity and TAPSE/sPAP were tested as prognostic factors; results were reported as Hazard Ratios (HR) and 95% confidence intervals [CI]. Data were expressed as mean and standard deviation. Survival data were expressed as median survival time (MST).

Ninety‐five dogs with mild (*n* = 10), moderate (*n* = 31) and severe (*n* = 54) PreHP were included. Significant differences for nTAPSE/sPAP(m/s), nTAPSE/sPAP(mmHg) × 10, (TAPSE/Ao)/sPAP(m/s) × 10, (TAPSE/Ao)/sPAP(mmHg) × 100 were observed in mild (1.8 ± 0.46; 1.3 ± 0.43; 2.1 ± 0.60; 1.6 ± 0.55), moderate (1.5 ± 0.53; 0.96 ± 0.34; 1.8 ± 0.58; 1.2 ± 0.40) and severe (0.84 ± 0.35; 0.44 ± 0.19; 1.1 ± 0.40; 0.55 ± 0.24) prePH (*P* < 0.0001). The 4 ratios were significantly lower in dogs with HF (*n* = 48) (0.89±0.45; 1.16±0.54; 0.49 ± 0.34; 0.62 ± 0.43) compared to dogs without HF (*n* = 47) (1.39 ± 0.53; 1.74 ± 0.54; 0.92 ± 0.39; 1.11 ± 0.46). All variables besides LA/Ao were significantly associated with TAPSE/sPAP measurements (<0.001). Sixty‐four dogs died, of which fifty‐one from CPD. HF (OR= 2 [1.1–3.9]) was the only factor significantly associated with long‐term CPD‐mortality in the multivariate model (*P* < 0.031). nTAPSE/sPAP(m/s) (OR= 0.38 [0.15–0.97]) and (TAPSE/Ao)/sPAP(m/s) × 10 (OR = 0.33 [0.12–0.87]) were both independent predictors of 1‐month CPD as well as pulmonary thromboembolism (*P* < 0.044).

nTAPSE/sPAP(m/s) and (TAPSE/Ao)/sPAP(m/s)*10 are associated with prePH severity, correlate with other echocardiographic variables and are independent predictors of short‐term cardio‐pulmonary mortality in dogs with PrePH.

**Disclosures**

No disclosures to report

## ESVC‐P‐17

### ESVC—European Society of Veterinary Cardiology

#### The effect of anesthesia drug on cardiac function parameters in cats

##### Ms Panprom^2^, Dr. Petchdee^1^

###### ^1^Faculty of veterinary medicine, Kasetsart University Kamphaeng, Saen, Thailand; ^2^Faculty of Veterinary Medicine, Kasetsart University Kamphaeng, Saen, Thailand

Heart rate variability is an assessment of cardiovascular risk. This study aimed to assess the effect of anesthetics drug on heart rate and heart rate variability (HRV) in cats and to provide information for clinical use. Twenty healthy cats of various breeds scheduled to undergo surgery participated in this study. Cats were pre‐medicated and induced with four protocols: Protocol 1, diazepam (0.3 mg/kg) and propofol (3 mg/kg); Protocol 2, diazepam (0.3 mg/kg) and alfaxalone (3 mg/kg); Protocol 3, diazepam (0.3 mg/kg) and ketamine (5 mg/kg); and Protocol 4, xylazine (1 mg/kg) and tiletamine/zolazepam (zoletil) (5 mg/kg), heart rate and variability were collected before and after anesthesia. All cats were determined for cardiac function by echocardiography. The low‐frequency power ratio (LF) and high‐frequency (HF) increased in all protocols except for the propofol group cats. The standard deviation of normal sinus rhythm (SDNN) in the ketamine group cats was higher than in other protocols. The decrease in heart rate variability has been associated with high blood pressure and the left atrium diameter. In cats with the ketamine group, the cardiac contraction function has decreased compared to other protocols. This study demonstrates that combining diazepam and propofol is safe and maintains cardiovascular function in cats. It can be used as a protocol for anesthesia in cats.

**Disclosures**

No disclosures to report

**Source of Funding**

Faculty of Veterinary Medicine, Kasetsart University

## ESVC‐P‐18

### ESVC—European Society of Veterinary Cardiology

#### Aortic insufficiency in dogs and cats: A retrospective study of clinical characteristics, echocardiographic findings, and prognosis in non‐infectious and non‐congenital cases

##### YJ Chang^1^, WT Chang^2^, MC Lam^1^, PY Lo^1^, CH Lin^2^

###### ^1^TACS‐Alliance Research Center, Taipei, Taiwan; ^2^1.National Taiwan University, 2.TACS‐Alliance Research Center, Taipei, Taiwan

Significant aortic insufficiency (AI) is generally considered rare in small animals without infectious endocarditis or congenital disease. Functional AI secondary to systemic hypertension (SHT) or degenerative valvular disease has been uncommonly described.

This study included 53 AI cases (41 dogs and 12 cats) without clinical or echocardiographic evidence suggestive of infectious endocarditis or congenital abnormality. According to the jet width/size on color flow Doppler and the profiles on spectral Doppler, AI severity was categorized as trivial‐to‐mild (jet width ≤25% of the left ventricular outflow tract [LVOT]; nonaliased signal just beyond the aortic valve; faint density or plateau‐shaped slope) or significant (jet width >25% of the LVOT; jet extending to or beyond the mitral leaflets; dense or increased slope). Clinical assessment, echocardiographic findings, and prognosis were retrospectively reviewed. Unfavorable outcomes were defined as target organ damage associated with SHT (TOD‐SHT), congestive heart failure (CHF), and all‐cause death (AC‐death) in this study.

The median age of the dogs and cats included in this study was 12.5 (range 6.5–19) and 7 (range 1–19) years, respectively. AI severity was significant in 23 (56%) dogs and 5 (42%) cats. Evidence of SHT at diagnosis and suspicion of degenerative changes of the aortic valve were observed in 37% and 44% of study dogs, and 25% and 8% of study cats, respectively. Systolic blood pressure and left ventricular wall thickness were not statistically different between significant and trivial‐to‐mild AI cases *(*p>0.05). Subsequent follow‐up information was obtained in 83% of the cases, with a median follow‐up duration of 12 (range 1–77) months. Cases with significant AI were at a higher risk of TOD‐SHT at 2 years after diagnosis than those with trivial‐to‐mild AI (92% vs. 46%, *P* = 0.027). However, the risk was not significantly different at 6 months after diagnosis (54% vs. 19%, *P* = 0.06). Moreover, the risk of CHF (50% vs. 22%, *P* = 0.33) or AC‐death (63% vs. 22%, *P* = 0.15) was not statistically different for significant and trivial‐to‐mild AI cases during follow‐up.

In conclusion, evidence of SHT or degenerative valvular change was identified only in a minority of dogs and cats with non‐infectious, non‐congenital AI. However, significant AI was associated with an increased risk of long‐term TOD‐SHT. These results suggest that unrecognized SHT might be the underlying cause of AI in some dogs and cats. Furthermore, significant AI can lead to an unfavorable prognosis and therefore requires attention.

**Disclosures**

A part of this study was supported by National Taiwan University & the award from TACS‐Alliance Research Center

**Source of Funding**

TACS‐A 2023 Award / A part of this study was supported by National Taiwan University

## ESVC‐P‐19

### ESVC—European Society of Veterinary Cardiology

#### Respiratory rate and breathing pattern in dogs and cats admitted to the intensive care unit at two small animal hospitals in Sweden

##### S Di Grado^1^, A Rave Vestberg^2^, J Häggström^3^, A‐L Saraluoma^2^, S Sandström^2^, C Wahldén^2^, J Andersson^3^, S Lundgren^4^, P. Åhr^1^, I Ljungvall^3^

###### ^1^Evidensia Södra Animal Hospital Kungens Kurva, Stockholm, Sweden; ^2^Anicura Regional Hospital Bagarmossen, Stockholm, Sweden; ^3^The University Animal Hospital (UDS), Swedish University of Agricultural science, Uppsala, Sweden; ^4^Awake Animal Hospital, Stockholm, Sweden

Information regarding respiratory rate (RR) and breathing pattern (BP) in dogs and cats at intensive care unit (ICU) is sparse. The aims of the study were to investigate RR and BP in dogs and cats from admission to ICU until discharge and to compare RR from video camera monitoring with results from traditional ocular monitoring close to the animal's cage. Dogs and cats that needed intensive care for at least four hours were enrolled into the study at two small animal hospitals in Sweden.

In total, 62 cats and 41 dogs were enrolled. During the entire observation period, median RR was lower (3.2 breaths/min, IQR 1‐ 4.3) when measured by video camera compared to traditional monitoring (*P* < 0.001). Respiratory rate was higher in dogs with respiratory disease (with camera; *P* = 0.0024) and cardiac disease (with and without camera; *P* = 0.0034) compared to in dogs with gastrointestinal disease and in cats with heart disease (with camera; *P* = 0.0003, without camera *P* = 0.0002) and respiratory disease (with camera; *P* = 0.0005, without camera; *P* = 0.0008) compared to cats with urinary tract disease. Respiratory rates measured one hour after admission to the ICU was higher in dogs (with camera; *P* = 0.0064, without camera; *P* = 0.025) and cats (with camera; *P* = 0.034) with an abnormal BP compared to dogs and cats with a normal BP.This study suggests that RR measured via video camera monitoring was lower compared to RR measured from traditional ocular monitoring and that dogs and cats with an abnormal BP have higher RR.

**Disclosures**

Dr. Ljungvall is involved in projects with/ has received funding/grants from Nestle Purina, Boehringer Ingelheim, Stiftelsen djursjukhus i storstockholm, Agria Insurance Ltd/SKK, Forsbergs stiftelse and has received consulting fees from Boehringer Ingelheim, —Dr. Häggström is involved in projects with/has received funding/grants from Nestle Purina, Boehringer Ingelheim, Orion Pharma, Stiftelsen djursjukhus i storstockholm, Forsbergs stiftelse and has received consulting fees from Boehringer Ingelheim, Royal Canin, Orion Pharma.

**Source of Funding**

Foundation animal hospital greater Stockholm (Stiftelsen djursjukhus i storstockholm)

## ESVC‐P‐20

### ESVC—European Society of Veterinary Cardiology

#### Temporal cardiomegaly induced by non‐cardiogenic respiratory distress in dogs with myxomatous mitral valve disease

##### J Lee^1^, H Hwang^1^, Jh Lee^1^, C Ahn^1^, H Kim^1^

###### ^1^Korea Animal Speciality Medical Institute, Seongnam, South Korea;

Dyspnea resulted from non‐cardiogenic causes (NCC) requires excessive cardiac demand, leading to hemodynamic instability in patients with congestive heart failure. However, there is very few report, describing the influence of respiratory failure on the underlying cardiac disease. We aimed to evaluate geometric and hemodynamic effects of non‐cardiogenic dyspnea on the patients with concurrent myxomatous mitral valve disease (MMVD).

In this retrospective cohort study, database of 198 MMVD dogs, emergently referred with acute respiratory distress was reviewed. Among them, 29 dogs that were eventually able to discontinue oral medication for congestive heart failure (CHF) were enrolled (NCC group). The detailed description of the NCC group is as follows: acute respiratory distress syndrome (*n* = 14), post‐anesthetic dyspneic events (*n* = 6), aspiration (*n* = 6), upper airway obstruction (*n* = 3). Clinical characteristics, including medical history, lab data and diagnostic imaging of the NCC group were compared to CHF dogs (CHF group, *n* = 169) at the timing of dyspneic events. Furthermore, the clinical indices of the NCC group were compared among their baseline (the acute onset of dyspnea) and follow‐ups (the resolution of non‐cardiogenic respiratory causes) which were tracked down over a year.

Although traditional radiographic and echocardiographic indices of the NCC group were not different from those of the CHF group at the onset of dyspneic event, LA/Ao indexed to LVIDDn in the NCC group (1.36±0.16) was significantly higher than the CHF group (1.02±0.11; *P* < 0.01). Additionally, the NCC group showed significantly lower SPO2 (%; 93.1 ± 4.5 vs. 97.5 ± 2.0; *P* < 0.01) and higher lactate level (mmol/L; 3.12 ± 1.4 vs. 1.0 ± 0.2; *P* < 0.01) than the CHF group. With their serial comparison of the indices among the baseline and the follow‐ups, only the NCC group revealed significant and gradual decreases in radiographic (VHS, VLAS) and echocardiographic parameters (LVIDDn, LA/Ao, E peak velocity) (*P* < 0.01). NT‐proBNP and the lactate level in the NCC group were also significantly reduced (*P* < 0.01) during follow‐ups and some of them were fully restored to within normal range.

These results suggest that non‐cardiogenic respiratory distress can have temporal and deleterious effects on cardiac geometry and hemodynamics in dogs with concurrent MMVD. Our study could provide insight into naturally developed reverse cardiac remodeling in MMVD, which is related to non‐cardiogenic respiratory failure.

**Disclosures**

No disclosures to report

## ESVC‐P‐21

### ESVC—European Society of Veterinary Cardiology

#### Association of decreased bodyweight‐independent tricuspid annular plane systolic excursion (TAPSE) measured in M‐mode with clinical signs of heart failure and cardiac death in dogs with pre‐capillary pulmonary hypertension

##### M Legrand, E Van Renterghem, M Lekane, K Gommeren, AC Merveille

###### Liège university, Liège, Belgium;

Pre‐capillary pulmonary hypertension (PrePH) is a complication of cardiopulmonary disorders in dogs, associated with shorter survival. Several echocardiographic indices allow for non‐invasive PH assessment. TAPSE is an index of right ventricular systolic function and in dogs with severe PH, TAPSE is reduced. nTAPSE assessed by 2D echocardiography was associated with a shorter survival time in dogs with PH of different origin. This study assessed whether reduced bodyweight‐independent TAPSE measured in M‐mode in dogs with PrePH was associated with clinical signs of heart failure at presentation and with cardiac death.

In this retrospective observational study, dogs with PrePH were included based on an increased measurable tricuspid regurgitation pressure gradient and classified into 3 categories (mild: 31–50 mmHg, moderate: 51–70 mmHg and severe >75 mmHg PH). Dogs were also classified into categories of heart failure (HF) according to the clinical and/or echocardiographic signs (absence, right congestive (R‐CHF), low output (LOHF) or both). TAPSE was measured in M‐mode and bodyweight‐independent values were calculated: TAPSE:Ao and nTAPSE, which were compared between different groups. Survival analysis was performed assessing 1‐month and long term outcome of dogs with reduced TAPSE:Ao and nTAPSE and those with normal values, based on previously described cutoff values: <0,65 and <4.5 mm/kg^0.285^, respectively. Survival data were obtained from the dog's record or through telephone interview. Cardio‐pulmonary death was defined as death or euthanasia attributable to cardio‐pulmonary diseases. Data are expressed as median and range [minimum‐maximum]. Survival data were expressed as median survival time (MST) and 95% confidence interval [CI].

Ninety‐five dogs were included; 10 with mild, 31 moderate and 53 severe prePH, of which 47 without HF, 9 R‐CHF, 20 LOHF and 18 with both. nTAPSE and TAPSE:Ao were significantly lower in dogs with severe (4.25[2.21–10.15]; 0.54[0.3–1.26]) compared with mild (5.97[3.5–8.25]; 0.7[0.51–1.09]) and moderate (5.83[1.19–8.92]; 0.7[0.14–1.05]) preHP. nTAPSE and TAPSE:Ao were significantly lower in dogs with LOHF (4.27[2.26–7.65]; 0.56[0.30–0.89]) and both types of HF (3.34[1.19–8.25]; 0.45[0.14–1.09]) compared to dogs without HF (5.48[3.49–10.15]; 0.69[0.42–1.26]) whereas R‐CHF was not different from other categories (4.70[3.20–6.77]; 0.61[0.41–0.91]). Sixty‐four dogs died, of which 51 experienced cardiac death. Reduced nTAPSE was significantly associated with 1‐month (*P* < 0.008) and long term (184 days [15–716] versus 485 [309–1055]; *P* < 0.04) cardiac‐pulmonary mortality whereas reduced TAPSE:Ao was not.

Dogs showing LOHF signs had reduced normalized TAPSE which might indicate poorer right ventricular systolic function. A nTAPSE <4.5 mm/kg^0.285^, assessed in M‐Mode was associated with cardio‐pulmonary death at 1‐month and long‐term survival.

**Disclosures**

No disclosures to report

## ESVC‐P‐22

### ESVC—European Society of Veterinary Cardiology

#### Respiratory sinus arrhythmia dynamics in dogs with various classes of naturally occurring myxomatous mitral valve disease

##### RA Baisan^1^, CA Turcu^1^, P Frydrychowski^2^, M Michałek^2^, J Gach^2^, V. Vulpe^1^, GD Ohad^3^

###### ^1^Iasi University of Life Sciences "Ion Ionescu de la Brad", Iasi, Romania; ^2^Faculty of Veterinary Medicine, Wrocław University of Environmental and Life Sci, Wroclaw, Poland; ^3^Veterinary Teaching Hospital, Koret School of Veterinary Medicine, Robert H. Smi, Koret, Israel

Respiratory sinus arrhythmia (RSA) is a sign of health and implies gradual, cyclic changes in heart rate (HR) associated with respiratory phases. This is modulated by the autonomic nervous system (ANS), mostly by its parasympathetic arm, namely the vagal tone, during normal sinus rhythm. The evolution of myxomatous mitral valve disease (MMVD) involves a progressive increase in volume and pressure overload in the left atrium and pulmonary veins, which might culminate in left‐sided congestive heart failure (L‐CHF) with loss of RSA. No studies have so far measured RSA dynamics in dogs with MMVD, prior to and following L‐CHF development. The aim of this study was to assess the proportion of various RSA degrees in dogs with various severity classes of MMVD.

This prospective study included control dogs and dogs with MMVD, examined between November 2021 and January 2023. Dogs were subjected to a complete cardiac physical examination, thoracic radiography, and bloodwork. MMVD dogs were classified based on echocardiography and thoracic radiography according to the ACVIM recommendations as B1 and B2 (preclinical MMVD without or with cardiomegaly), Ca (acute onset of L‐CHF due to MMVD), and Cc (with a history of L‐CHF stabilized with therapy). Control dogs (N) were included if they demonstrated no cardiac or biochemical abnormalities.

Dual channel electrocardiography along with a respiratory curve was recorded. RSA was calculated, the magnitude of which was defined as the difference between HR‐peak and HR‐though, in percent of the average HR. A mean of three consecutive breaths was then computed.

Ninety‐five dogs were divided into N (*n* = 20), B1 (*n* = 30), B2 (*n* = 16), Ca (*n* = 19), and Cc (*n* = 10). An overall decrease in RSA was observed with increased disease severity, except for Class Cc, in which RSA values were higher compared to those in Class Ca. A significantly higher RSA was observed in N and B1 dogs compared to Ca (*P* < 0.01). Importantly, B2 dogs had a significantly lower RSA when compared to N dogs (*P* < 0.05). The 95% CI in control dogs ranged from 30.7 to 48.4%, while in MMVD dogs it ranged from 18 to 27%.

The present study shows that RSA decreases with MMVD severity. However, Cc dogs tend to regain a normal RSA when stabilized. Moreover, dogs with a preclinical disease with cardiomegaly showed lower RSA values than those with a milder disease. These findings can be useful for risk stratification and for both diagnostic and therapeutic management decisions in dogs with MMVD.

**Disclosures**

No disclosures to report

## ESVC‐P‐23

### ESVC—European Society of Veterinary Cardiology

#### Proteomics and coagulation markers in cats with asymptomatic and symptomatic hypertrophic cardiomyopathy

##### Mr. Sukumolanan^2^, Dr. Petchdee^1^

###### ^1^Faculty of veterinary medicine, Kasetsart University Kamphaeng, Saen, Thailand; ^2^Faculty of Veterinary Medicine, Kasetsart University Kamphaeng, Saen, Thailand

Hypertrophic cardiomyopathy (HCM) is a common cause of death in cats. Although cats are asymptomatic, some may experience symptoms such as heart failure, artery thromboembolism, or sudden cardiac death. Therefore, early identification of biomarkers may reduce the risk of sudden death in cats. This study compares plasma concentrations of d‐dimer, prothrombin time (PT), proteomic profiles, and echocardiographic parameters between asymptomatic cats with myosin‐binding protein C mutation and cats with symptomatic hypertrophic cardiomyopathy. Blood samples from fifteen asymptomatic cats and ten cats with HCM were obtained. All cats were determined for cardiac function by echocardiography. Proteomics profiles and coagulation markers differed in cats with hypertrophic cardiomyopathy. However, the coagulation markers were significantly lower in the asymptomatic stage of the disease. The potentially beneficial effect of proteomics profiles and coagulation markers in the asymptomatic and symptomatic stages may be used to evaluate the risk of arterial thrombosis in cats and determine the HCM treatment in cats.

**Disclosures**

No disclosures to report

**Source of Funding**

Faculty of Veterinary Medicine, Kasetsart University

## ESVC‐P‐24

### ESVC—European Society of Veterinary Cardiology

#### Clinical and echocardiographic findings in dogs with heartworm caval syndrome

##### JI Matos^2^, R Abecassis^3^, SN García‐Rodríguez^1^, AJ Santana^4^, D Saavedra^4^, N Costa‐Rodríguez^3^, E Carretón^1^, JA Montoya‐Alonso^1^

###### ^1^Internal Medicine, Faculty of Veterinary Medicine, Research Institute of Biomedi, Las Palmas de Gran Canaria, Spain; ^2^Internal Medicine, Faculty of Veterinary Medicine, Research Institute of Biomedi, Arucas, Spain; ^3^Faculty of Veterinary Medicine, University of Las Palmas de Gran Canaria, Las Pa Las Palmas de Gran Canaria, Spain; ^4^Anicura Albea veterinary hospital, Las Palmas de Gran Canaria, Spain, Las Palmas de Gran Canaria, Spain

Caval syndrome is a serious disorder caused by massive parasitism of adult *Dirofilaria immitis*, characterized by cardiogenic shock and complicated by anemia, metabolic acidosis, and disseminated intravascular coagulation. The objective of this study was to evaluate some clinical findings and echocardiographic measurements in a group of dogs with caval syndrome in order to determine their relevance in the identification of this condition.

A prospective descriptive study was carried out in 16 dogs with caval syndrome (group A), 16 animals with heartworm without caval syndrome (group B) and 16 healthy animals (group C). Data on presence of respiratory symptoms, signs of right congestive heart failure, and presence of hemoglobinuria were collected. The serum values of NT‐proBNP were determined. Furthermore, the number of adult filariae in the right chambers and vena cava was determined after surgical extraction or necropsy in group A.

The echocardiographic measurements determined were: right pulmonary artery distensibility index (RPADi), right ventricular shortening fraction (RVOT‐FS), right ventricular end‐diastolic area (RVEDAi), right atrial area (RAAi), tricuspid regurgitation pressure gradient (TRPG), tricuspid annular systolic displacement (TAPSE), right ventricular acceleration time to ejection time (AT:ET) ratio, pulmonary trunk to Aorta ratio (PT:Ao), the ratio between the pulmonary vein and the pulmonary artery (PV:PA) and the maximum right myocardial velocities measured in early diastole (E'), late diastole (A') and systole (S), calculated the ratio E'/A' and the global TDI index of myocardial function (Global‐TDI).

The results show a greater number of affected dogs with respiratory symptoms (group A 87.52%; group B 43.75%; group 0%), right congestive heart failure (group A 62.51%; group B 18.75%; and group C 0%) and hemoglobinuria (group A 68.75%; group B 0%; and group C 0%) in dogs from group A. NT‐pBNP concentrations were considerably higher in group A (group A 3769.45 ± 620.67 pmol/l; group B 878.15 ± 288.25 pmol/l; and group C 524.91 ± 260.13 pmol/l). The average number of extracted filariae in group A was 15.29 ± 8.23. Regarding the echocardiographic measurements, extremely increased (RVEDAi, RAAi, TRPG, PT:Ao, A') or decreased (RPADi, RVOT‐FS, TAPSE, AT:ET, PV:PA, E', S, E':A', Global‐TDI) values were reported in the animals of group A compared to the animals of groups B and C.

Heartworm caval syndrome is considered a clinical emergency with fatal consequences that must be resolved quickly. The echocardiographic measurements and the clinical findings determined in the present study have proven to be relevant for its effective determination.

**Disclosures**

No disclosures to report

**ECVIM CSF Funding**

Yes

## ESVC‐P‐25

### ESVC—European Society of Veterinary Cardiology

#### The prognostic value of circulating cardiac and renal biomarkers in dogs with myxomatous mitral valve disease

##### N Iwasa^1^, R Kumazawa^1^, M Shimizu^1^, T Iwasa^1^, M Hara^2^, M Kawabe^3^, M Iwata^4^, K Watanabe^2^, Y Kobatake^2^, S Takashima^2^, N Nishii^2^

###### ^1^Hashima animal hospital Hashima, Gifu, Japan; ^2^Joint Department of Veterinary Medicine, Faculty of Applied Biological Sciences, Yanagido, Gifu, Japan; ^3^Animal Medical Center, Gifu university Yanagido, Gifu, Japan; ^4^Department of Orthopaedics Surgery, Graduate School of Medical and Dental Scienc, Tokyo, Japan

Myxomatous mitral valve disease (MMVD) is the most common acquired heart disease in small‐size dog breeds. Blood circulating biomarkers that are specific for cardiac functions are used to determine the prognosis and severity of MMVD.Moreover, blood circulating biomarkers that are specific for renal functions have also proven useful for MMVD prognosis. Although the detection of such circulating biomarkers is more convenient than cardiac ultrasound or radiography, it is still not known which option is superior for the prognosis of MMVD. The aim of this study was to evaluate the prognostic value of blood circulating cardiac and renal biomarkers in dogs with MMVD.

This is a retrospective cross‐sectional study, which included thirty‐six client‐owned small‐size dogs with MMVD. The measured values of blood circulating cardiac biomarkers (N‐terminal pro‐brain natriuretic peptide [NT‐proBNP], atrial natriuretic peptide [ANP], and troponin I) and blood circulating renal biomarkers (blood urea nitrogen [BUN], creatinine, symmetric dimethylarginine [SDMA], and cystatin C [Cys‐C]) were collected from medical records. We also collected the imaging data from the same records (vertebral heart score [VHS], vertebral left atrial size [V‐LAS], left atrial‐to‐aortic ratio [LA/Ao], and left ventricular end‐diastolic internal diameter normalized for body weight [LVIDDN]). Next, we recorded the 1‐year survival rates and performed the receiver operating characteristic curve (ROC) analysis of the death prediction performance by the above‐mentioned measurements. Finally, we generated the Kaplan‐Meier survival curves for the MMVD‐specific survival in dogs that were stratified into high‐ or low‐ measurement groups based on the ROC analysis results, followed by the log‐rank test analysis.

The three parameters with the highest predictive accuracy were NT‐proBNP, with an area under the curve (AUC) of 0.85 (95% confidence interval [CI] 0.72–0.99), followed by ANP, with an AUC of 0.80 (95% CI 0.64–0.95), and serum Cys‐C, with an AUC of 0.78 (95% CI 0.61–0.96). Groups with high NT‐proBNP, ANP, and Cys‐C circulating values had significantly shorter MMVD‐specific survival rates (*P* < 0.01).

Therefore, higher NT‐proBNP, ANP, Cys‐C circulating values were associated with a worse prognosis in MMVD and were better predictors than the imaging data, such as VHS, V‐LAS, LA/AO, and LVIDDN.

**Disclosures**

No disclosures to report

## ESVE‐P‐1

### ESVE—European Society of Veterinary Endocrinology

#### Radioimmunoassay (RIA) enabled thyroid profile in healthy dogs: effect of age, sex, breed, and diet

##### G Galdhar, G Gaikwad

###### Mumbai Veterinary College (MAFSU), Mumbai, India

Hypothyroidism is considered one of the most frequently encountered endocrine disorders in dogs. The clinical signs of hypothyroidism are numerous, variable, and nonspecific. Canine thyroid function is now evaluated mainly with the measurement of serum levels of total triiodothyronine (TT3), total thyroxine (TT4), and free thyroxine (FT4). RIA is sensitive *in vitro* method for the assessment of antigens from biological fluids i.e. hormones, minerals, vitamins, and so forth. I^125^ is a commonly used radioisotope for RIA because of its long half‐life (*t*_1/2_ = 60 days). This method offers a convenient assay of large numbers of samples with good precision.

The present study was undertaken to investigate the variations in thyroid profile (TT3, TT4, and FT4) in relation to age, sex, breed, and diet. A total of 192 apparently healthy dogs were enrolled and the thyroid hormone concentration was measured by RIA.

The present study reported a significant (*P* ≤ 0.05) decrease in concentration in TT4 and FT4, as age increases. However, changes in FT3 were non‐significant (*P* ≤ 0.05). Sex‐wise variation in thyroid profile reported non‐significant (*P* ≤ 0.05) difference in TT3, TT4_,_ and fT_4_ between males and females. Breed‐wise study revealed significant (*P* ≤ 0.05) variation in TT3 and TT4 concentrations, however, fT4 values did not differ significantly (*P* ≤ 0.05). Labrador breed recorded comparatively lowest TT3 and TT4 concentrations than other breeds. Studies on alternation in thyroid profile in context to diet reported non‐significant (*P* ≤ 0.05) changes in the levels of TT3 and TT4 and significant (*P* ≤ 0.05) changes in the levels of FT4_._

Thus, the present study suggests that the thyroid profile in healthy dogs deviates in relation to their physiological states viz. age, sex, breed, and diet.

**Disclosures**

No disclosures to report

**Source of Funding**

Present research work was undertaken with funding from the host university (MAFSU RESEARCH GRANTS‐ Maharashtra Animal and Fishery Sciences University‐ Nagpur‐M.S.‐India)

## ESVE‐P‐2

### ESVE—European Society of Veterinary Endocrinology

#### Machine‐learning as a diagnostic tool for naturally occurring hypercortisolism in dogs

##### M Bennaim^1^, ME White^2^, JA Jaffey^2^, CT Mooney^3^

###### ^1^Centre Hospitalier Vétérinaire Anicura Aquivet, Eysines, France; ^2^Midwestern University College of Veterinary Medicine, Glendale, Arizona, USA of America; ^3^School of Veterinary Medicine, University College Dublin, Ireland

Diagnosing canine hypercortisolism is challenging. In the absence of a gold standard test, the diagnosis is currently based on clinical presentation and results from multiple diagnostic modalities. Endocrine tests are imperfect and results can be difficult to interpret.

The aim of this study was to construct a machine‐learning model using clinicopathological abnormalities and results of endocrine tests to aid in the diagnosis of hypercortisolism.

Data were retrospectively collected from dogs in which hypercortisolism was investigated. There were 25 laboratory features (basic clinicopathology and results of ACTH stimulation test [ACTHst] and low‐dose dexamethasone suppression test [LDDST]) collected including 20 categorical and 5 numerical. Four modeling techniques were used (logistic regression, random forest, decision tree and support vector machine). Algorithms were cross‐validated using a nested cross‐validation approach (10 outer folds, 5 inner folds), repeated 3 times. Metrics were computed as the average of the 3 × 10 outer folds metric values. The primary measure of model performance was the area under the receiver operator curve (AUROC).

Overall, 136 dogs were included: 72 dogs with hypercortisolism confirmed by response to treatment and/or post‐mortem examination and 64 control dogs suspected to have hypercortisolism, but eventually diagnosed with non‐adrenal illness. The four machine‐learning methods showed excellent to outstanding discrimination (AUROC between 0.88 and 0.96). Random forest indicated the best overall performance with an AUROC = 0.96 (95% CI 0.92–1), sensitivity = 0.89 (95% CI 0.78–0.99), specificity = 0.84 (95% CI 0.73–0.96) and positive and negative predictive values of 0.88 (95% CI 0.80–0.96) and 0.89 (95% CI 0.79–0.99), respectively. Compared to the LDDST and the ACTHst, the trained machine‐learning model (MLM) demonstrated a significantly higher AUROC (*P* < 0.001 in each case), specificity (*P* = 0.001 in each case) and positive predictive value (*P* < 0.001 and *P* = 0.003, respectively). The sensitivity and the negative predictive value of the MLM were both significantly higher compared to the ACTHst (*P* = 0.003 and *P* = 0.001, respectively) but not significantly different compared to the LDDST (*P* = 0.057 and *P* = 0.063, respectively).

In conclusion, our trained MLM outperforms historically used endocrine tests to discriminate hypercortisolism from non‐adrenal illness in a population of dogs in which hypercortisolism is clinically suspected. The use of such model may decrease misdiagnosis of hypercortisolism.

**Disclosures**

Jared Jaffey is an internal medicine consultant for IDEXX.

## ESVE‐P‐3

### ESVE—European Society of Veterinary Endocrinology

#### A prospective study to examine the natural variation of interstitial glucose levels in healthy adult dogs utilising a continuous glucose monitoring system

##### SJ Reeves^1^, A Gal^2^, CL Gordon^1^, JA Harper^1^, RK Burchell^1^

###### ^1^North Coast Veterinary Specialist and Referral Centre, Sippy Downs, Australia; ^2^College of Veterinary Medicine, University of Illinois Champagne, Ilinois, USA

Continuous interstitial glucose (IG) has become a popular method of diabetic monitoring. The variation of IG in normal dogs has not been established and inferences as to the range of IG concentrations has been derived from blood glucose. A previous study in supreme athlete working dogs demonstrated low IG events, but it is uncertain if this occurs in healthy pet dogs. Our study aimed to evaluate the natural variation of IG in adult healthy dogs, as this might inform insulin dosage change decisions made in clinical practice. Interstitial glucose measurements were collected from twenty‐four adult healthy dogs utilising the flash glucose monitoring Freestyle Libre system. Inclusion criteria were an age range of one to ten years of age and a minimum database involving bloodwork and urinalysis. Data were collected for a minimum of 7 h to a maximum of 14 days. We estimated the population‐based reference interval (RI) for IG according to ASVCP guidelines by bootstrapping the 90% confidence limit for the population reference limits (i.e., the mean ± 2SD) after removing extreme outliers using the Tukey's interquartile fences. The estimated population‐based reference interval for IG was 3.87 to 6.99 mmol/L. Of the IG readings, 864/14946 (5.78%) were outside the reference range. Fifteen dogs had at least one IG < RI (total of 489 readings) and most low IG readings happened between 24:00 and 10:00. Twenty one dogs had at least one IG > RI (375 readings) and most high IG readings happened between 10:00–20:00. The mean low IG was 3.48 ± 0.32 and mean high IG was 7.59 ± 1.41. The mean glucose of the entire cohort was 5.43 ± 0.86 mmol/L with coefficient of variation of 15%. Low glucose events occurred with no adverse clinical signs noted. Device removal or dislodgement of the sensor was a common problem. Our study found that healthy dogs have a wide variation of IG readings, with some at levels that would be judged to be hypoglycemic. This should be taken into consideration when managing diabetic patients.

**Disclosures**

No disclosures to report

## ESVE‐P‐4

### ESVE—European Society of Veterinary Endocrinology

#### Signalment, clinicopathological findings, management practices and occurrence of comorbidities in cats with diabetes mellitus in Germany: prospective study of 148 cases

##### B Guse^1^, J Langenstein^2^, N Bauer^1^, K Hazuchova^1^

###### ^1^Small Animal Clinic, Justus‐Liebig‐University Giessen, Giessen, Germany; ^2^SYNLAB.vet GmbH, Leverkusen, Germany

Diabetes mellitus (DM) is a common disease in cats, but prospective studies on laboratory findings, management and concurrent diseases in general practice (GP) are scarce. The aim of this study was to describe epidemiological and clinicopathological findings, management practices and occurrence of comorbidities in feline DM in German GP.

Data was obtained prospectively from questionnaires (signalment, body condition score [BCS], clinical signs, DM treatment and monitoring practices, comorbidities) and laboratory submissions (erythrocyte count, creatinine, alkaline phosphatase, alanine aminotransferase, bilirubin, cholesterol, triglycerides, fructosamine, total thyroxine, DGGR‐lipase, cobalamin, ß‐hydroxybutyrate, insulin‐like growth factor‐1 [IGF‐1]) to a commercial laboratory SYNLAB Vet between May 2021 and July 2022. Non‐parametric tests were used to compare laboratory results and proportion of cats with anaemia (erythrocyte count ≤4.21 × 10^12^/l), creatinine >250 μmol/l, alanine aminotransferase >510 U/l, alkaline phosphatase >330 U/l, bilirubin ≥35 μmol/l, DGGR‐lipase >26 U/l, cobalamin <295.2 pmol/l (corresponding to 400 ng/l, recommended for substitution by Texas A&M), IGF‐1 >746 ng/ml and ß‐hydroxybutyrate >2.4 mmol/l between clinically well‐controlled (WD) and poorly‐controlled diabetics (PD) (judged based on questionnaire responses by submitting veterinarians). Significant was *P* < 0.05, effect size was assessed by Cramér V or r.

Median age of the 148 included cats at DM diagnosis was 11.3 years, 65% were male‐neutered, 85% were Domestic Shorthair, 42% were currently and 63% were previously overweight (BCS>5/9). Fifteen % were not yet treated with insulin, 39% were treated for <6 months; most commonly used insulin was ProZinc (59%); 41% were fed diabetic diet; fructosamine measurement (28%) and home blood glucose curves (27%) were the most common monitoring methods. Most common comorbidities were pancreatitis (13%), chronic enteropathy (8%), heart disease (8%). Based on questionnaires, 21% cats were WD and 79% were PD. PD compared to WD had lower creatinine (median 107 vs. 135 μmol/l, *P* = 0.009, *r* = 0.23), higher bilirubin (median 3.42 vs. 3.42 μmol/l, *P* = 0.035, *r* = 0.19) and higher ß‐hydroxybutyrate (median 0.23 vs. 0.1 mmol/l, *P* = 0.003, *r* = 0.26). Twenty‐three (15.5%) cats had elevated IGF‐1, indicating hypersomatotropism. Cobalamin was below the laboratory reference range (<664pmol/l) in 46%, and <295.2 pmol/l in 23%. Eighty‐one cats had elevated DGGR‐lipase, of which 17% had pancreatitis according to their vets, and 14% had symptoms compatible with pancreatitis.

In the majority of cats in German GP, their DM is poorly‐controlled. Comorbidities (including pancreatitis, hypocobalaminaemia, and elevated IGF‐1, indicating hypersomatotropism) are common and should be tested for. Lower creatinine in PD can be explained by reduced muscle mass.

**Disclosures**

The blood tests performed as a part of this study were funded by Synlab Vet and were free of charge to the owners. Katarina Hazuchova has a consultancy agreement with Boehringer Ingelheim.

**Source of Funding**

The blood tests performed as a part of this study were funded by Synlab Vet and were free of charge to the owners.

## ESVE‐P‐5

### ESVE—European Society of Veterinary Endocrinology

#### Thyroid hormone concentrations in healthy Irish Setters and Rhodesian Ridgeback dogs: a diagnostic comparison

##### RJ Egbert^1^, AM LaClair^2^, K Refsal^2^, JM Brudvig^2^, BK Petroff^2^

###### ^1^Lansing, USA; ^2^Michigan State University, East Lansing, USA

Primary hypothyroidism is the most common canine adult‐onset endocrinopathy. The etiology in most instances is attributed to autoimmune‐related destruction of thyroid follicular tissue, with similarity to Hashimoto's thyroiditis in humans. Laboratory reference intervals for canine thyroid testing have historically been derived with samples collected from a variety of purebred and mixed‐breed individuals. Previous work has shown that euthyroid test results in some breeds (e.g., sighthounds) may deviate from reference intervals derived from a variety of breeds. The objective of this study was to compare thyroid profiles in euthyroid Irish Setters (IS) and Rhodesian Ridgebacks (RR), two canine breeds with a propensity for lymphocytic thyroiditis. Blood samples were collected with owner consent from dogs at national specialty shows providing thyroid testing for breeding animals. Testing was performed using commercially available radioimmunoassays (free T4 by dialysis, total T3, T3/T4 autoantibodies), chemiluminescent immunoassays (TSH, total T4) and ELISA (thyroglobulin autoantibody) validated for use with canine sera. Diagnosis of canine hypothyroidism is based upon clinical signs together with measurement of thyroid hormones (T3 and T4), thyroid stimulating hormone (TSH). Thyroglobulin autoantibody (TgAA) is used as a marker of autoimmune thyroiditis as well. Dogs were classified as healthy based upon history, physical examination findings, and clinical pathology data (complete blood count and biochemical profile). Exclusion criteria included evidence of autoimmune thyroiditis and/or hypothyroidism, non‐thyroidal illness, estrus, pregnancy, and administration of medications that could alter thyroid function. Reference data were prepared from 118 Irish Setters (124 collected, 6 excluded) and 101 Rhodesian Ridgebacks (125 collected, 24 excluded). Thyroid hormone ranges were mostly similar between IS and RR with the exception of TSH, which ranged lower in IS. Thyroid hormones test results were: TT4 35.0 ± 9.4 (IS) vs. 22.7 ± 8.1 (RR) nmol/L (*P <* 0.001), fT4d 24.7 ± 8.1 (IS) vs. 23.7 ± 6.1 (RR) pmol/L (*P* = 0.35), TT3 1.37 ± 0.28 (IS) vs. 1.20 ± 0.34 (RR) nmol/L (*P <* 0.001), TSH 0.08 ± 0.05 (IS) vs. 0.22 ± 0.16 (RR) ng/ml (*P <* 0.001). The current study indicates that while there were differences in mean thyroid hormone concentrations in healthy dogs of different breeds, ranges were similar with the exception of thyroid stimulating hormone.

**Disclosures**

No disclosures to report

**Source of Funding**

AKC Canine Health Foundation

## ESVE‐P‐6

### ESVE—European Society of Veterinary Endocrinology

#### Interpretation of screening tests for hypercortisolism by first‐opinion western European veterinary surgeons

##### M Carvalho^1^, R Oliveira Leal^2^, S Golinelli^3^, F Fracassi^3^, C Arenas^4^, M Pérez‐Alenza^5^, S Galac^6^, C T M Mooney^7^, M Bennaim^8^

###### ^1^Hosp. Escolar Vet.—Fac. Med. Vet. U. Lisboa, Lisbon, Portugal; ^2^Centre for Interdisciplinary Research in Animal Health, Fac.Vet.Med., ULisboa, Lisbon, Portugal; ^3^University of Bologna, Bologna, Italy; ^4^Anicura Hosp. Vet. Valencia Sur, Valencia, Spain; ^5^Complutense University, Madrid, Spain; ^6^Faculty of Veterinary Medicine, Utrecht University, Utrecht, Netherlands; ^7^School of Veterinary Medicine, University College Dublin, Dublin, Ireland; ^8^Clinique Vétérinaire Anicura Aquivet, Eysines, France

Hypercortisolism (HC) is a diagnosis made primarily in primary care practices in Western Europe. Most Western European primary care veterinarians (WEPCV) use the same initial screening test to investigate suspected cases. This study aimed to determine how these test results are interpreted in this population.

An online English survey translated into four different languages (Portuguese, Spanish, French and Italian) was developed using an electronic platform. Respondents were recruited through social network veterinary groups, mailing lists and presentations. Questions focused on testing protocols.

Overall, 1070 responses were included (Italy [*n* = 584], France [*n* = 218], Portugal [*n* = 143], Spain [*n* = 80], Belgium [*n* = 21], Netherlands [*n* = 15], Republic of Ireland [*n* = 4], Luxembourg [*n* = 3] and Switzerland [*n* = 2]). Among 498 (46.5%) respondents who perform an ACTH stimulation test as an initial screening test, 157 (31.5%) and 44 (8.8%) indicated they would not know how to interpret a result within and above reference interval (RI), respectively. Among the remaining respondents, 173 (50.7%) typically find a result within RI sufficient to exclude HC and 418 (92.1%) typically find a result above RI sufficient to confirm HC. Among 435 (40.7%) respondents who perform a low‐dose dexamethasone suppression test as an initial screening test, 140 (32.2%) and 40 (9.2%) indicated they would not know how to interpret a result within and above RI, respectively. Among the remaining respondents, 185 (62.7%) typically find a result within RI sufficient to exclude HC and 358 (90.6%) typically find a result above RI sufficient to confirm HC. Among 77 (7.2%) respondents who perform a urine corticoid:creatinine ratio (UCCR) as an initial screening test, 19 (24.7%) and 8 (10.4%) indicated they would not know how to interpret a result within and above RI, respectively. Among the remaining respondents, 50 (86.2%) find a result within RI sufficient to exclude HC and 17 (24.6%) find a result above RI sufficient to confirm HC. Among 42 (3.9%) respondents who perform a UCCR combined with an oral high‐dose dexamethasone suppression test as an initial screening test, 8 (19%) and 5 (11.9%) indicated they would not know how to interpret a result within and above RI, respectively. Among the remaining respondents, 22(64.7%) typically find a result within RI sufficient to exclude HC and 35(94.6%) typically find a result above RI sufficient to confirm HC.

These results show that WEPCV have difficulties interpreting the screening tests they commonly use to investigate HC, raising concerns for common misdiagnosis. There is room for further education of WEPCV.

**Disclosures**

F. Fracassi: Financial support: Dechra, MSD, Monge Speaking & consultancies: Boehringer Ingelheim, Dechra, MSD, Royal Canin, Hill's, Nestlé Purina, La Vallonea Stefania Golinelli: Financial support: Ph.D. scholarship funded by Dechra Speaking & consultancies: Dechra Carolina Arenas: Consultancies: Dechra R. Leal: Speaking & consultancies:Veterinary information network USA, Vet2Vet (IT), Boehringer Ingelheim, Dechra

## ESVE‐P‐8

### ESVE—European Society of Veterinary Endocrinology

#### Perceptions of first‐opinion veterinarians and owners in Switzerland about treatment of feline hyperthyroidism

##### L Stadler, R Özcelik, M Campos

###### Vetsuisse Faculty, University of Bern, Bern, Switzerland

The aim of this study was to investigate the perceptions and concerns of first‐opinion veterinarians and owners in Switzerland about the different treatments of feline hyperthyroidism. Online surveys were created for veterinarians and owners of hyperthyroid cats. Participants were contacted electronically. The surveys were also advertised in newsletters and through referring veterinarians.

A total of 171 first‐opinion veterinarians completed the survey. The most common long‐term therapy was anti‐thyroid drugs (98.2%). If costs and owner motivation were not a limiting factor, radioiodine was the preferred treatment option (47.4%). The opinion of veterinarians about the efficacy of each treatment modality and treatment preference was significantly different between veterinarians that had already referred cats for radioiodine treatment and veterinarians that had never referred for radioiodine treatment. Veterinarians consider long hospitalization, costs, traumatic experience by hospitalization and age as major concerns of owners about radioiodine therapy.

A total of 99 owners completed the survey. Anti‐thyroid drugs (65.7%) were the most frequent treatment used, followed by radioiodine (30.3%), thyroidectomy (2.0%) and low iodine diet (2.0%). Owners’ perception of their cats quality of life (QoL) after therapy was significantly higher for owners of cats treated with radioiodine compared to owners of cats treated with antithyroid drugs (*P* < *0.001*). Owners’ perception of the impact of treatment on their cats QoL (*P* = 0.006), their own QoL (*P* < *0.001*) and impact of treatment on their relationship with their cat (*P* = 0.01) were significantly more positive for owners of cats treated with radioiodine compared to owners of cats treated with antithyroid drugs. Similarly, owners’ perception of treatment efficacy (*P* < *0.001*) and owners’ satisfaction with treatment choice (*P* = *0.002)* were significantly higher for owners of cats treated with radioiodine compared to owners of cats treated with antithyroid drugs. Nearly half (47.8%) of owners of cats that didn't received radioiodine answered they had never heard of radioiodine treatment. Contrary to the expectation of veterinarians, cost was not considered a major concern by owners regarding the choice of treatment, whereas long hospitalization period and well‐being of the cat had higher impact on treatment choice.

In conclusion, having already referred patients for radioiodine treatment had a significant effect on the opinion of veterinarians regarding treatment of choice and perception of efficacy of different treatment modalities. These results should be considered during education of veterinary students and continuous education of veterinarians in Switzerland.

**Disclosures**

The authors declare that radioiodine treatment is offered at the Small Animal Clinic of the University of Bern

## ESVE‐P‐9

### ESVE—European Society of Veterinary Endocrinology

#### Urinary aldosterone to creatinine ratio and fractional excretion of sodium and potassium in healthy cats, cats with primary hyperaldosteronism and cats with secondary hyperaldosteronism

##### M Kurtz^1^, T Buronfosse^2^, G Benchekroun^3^

###### ^1^Ecole Nationale Vétérinaire d'Alfort, Maisons‐Alfort, France; ^2^VetAgroSup, Marcy l'Etoile, France; ^3^Ecole Nationale Vétérinaire d'Alfort, Maisons‐Alfort, France

Primary hyperaldosteronism (PHA) is characterized by an excessive secretion of aldosterone. Mineralocorticoid excess exacerbates tubular potassium losses as well as renal sodium and fluid retention, which results in hypokalemia and systemic hypertension. Little is known about urinary fractional excretion of sodium (FE_Na_) and potassium (FE_K_), as well as urinary aldosterone to creatinine ratio (UACR) in feline PHA.

We aimed at evaluating the diagnostic utility of urine electrolytes and aldosterone measurements in feline PHA.

Urinary aldosterone (U_aldosterone_), sodium (U_Na_) and potassium (U_K_) concentrations were measured in leftover samples from 4 groups of cats: healthy middle‐aged cats (healthy; *n* = 5); acute or chronic kidney disease (CKD, *n* = 10); hyperthyroidism (HTH; *n* = 4); and PHA (PHA; *n* = 5). UACR, FE_Na_ and FE_K_ (%) were calculated. Results were compared using Kruskal‐Wallis test, and are presented as medians [min; max]. Correlation between UACR and serum aldosterone concentration (SAC) was evaluated using the Spearman correlation test. Significance was set as *P* < 0.05.

Median age in the studied population was 14 years old [7; 17]. Median UACR (10^−3^), U_Na_, FE_Na,_ U_K_ and FE_K_ were 76 [49; 144], 155 mmol/L [91; 263]; 0.04% [0.02; 0.82], 84 mmol/L [17; 112] and 1.2% [0.6; 2.1] in healthy cats, 170 [64; 1631], 163 mmol/L [26; 302], 0.53% [0.01; 1.53], 27 mmol/L [18; 53] and 2.6% [0.47; 6.00] in CKD cats, 187 [66; 2725], 106 mmol/L [97; 283]; 0.1% [0.04; 1.20], 22 mmol/L [18; 61] and 0.9% [0.48; 2.30] in HTH cats, and 233 [75; 10476], 147 mmol/L [119; 320]; 0.16% [0.11; 24.00], 24 mmol/L [18; 58] and 3.8% [0.3; 62.0] in PHA cats.

UACR, U_Na_, FE_Na,_ U_K_ and FE_K_ were not significantly different between groups (*P* = 0.20, *P =* 0.71, *P* = 0.36, *P* = 0.24 and *P* = 0.12, respectively). UACR and SAC were moderately correlated (*r*(23) = 0.52, *P* = 0.009).

In conclusion, the UACR, FE_Na_ and FE_K_ did not discriminate cats with PHA from healthy cats or cats with SHA caused by an intercurrent disease potentially upregulating the renin‐angiotensin system in our study.

**Disclosures**

No disclosures to report

## ESVE‐P‐10

### ESVE—European Society of Veterinary Endocrinology

#### The effect of treatment modality on changes in quality of life in hyperthyroid cats

##### F Blunschi^1^, S Muthmann^1^, I Schofield^2^, N Bauer^1^, K Hazuchova^1^

###### ^1^Small Animal Clinic, Justus‐Liebig University Giessen, Giessen, Germany; ^2^CVS (UK) Ltd, Norfolk, UK

Impaired health‐related quality‐of‐life (HRQoL) has been detected in hyperthyroid cats in a cross‐sectional study. The effect of treatment of hyperthyroidism on HRQoL, and any differences between curative (e.g., radioiodine [RAI]) and palliative (e.g., antithyroid drugs [ATD]) treatment modalities are unknown. We hypothesized that HRQoL improves with treatment and that RAI‐treated cats achieve better, that is, lower total HRQoL scores than ATD‐treated. To test these hypotheses, HRQoL was evaluated in cats treated with RAI or ATD over a 6‐month period.

For this prospective study, cats medically treated for hyperthyroidism for less than 6 months were recruited between March and December 2022. Based on owners’ decisions, cats either underwent RAI treatment (RAI‐group) or continued receiving ATD (ATD‐group). HRQoL was assessed using a validated questionnaire (HyperthyroidismQoL‐cat) at enrolment and after 1, 3 and 6 months, alongside measurement of T4 and creatinine. HRQoL was compared between groups at enrolment and within groups between study timepoints by non‐parametric tests. The overall effect of treatment group and timepoint on log(HRQoL) was assessed by mixed‐effect modelling. Data is presented as median (range). Significance was set at *P* < 0.05.

Forty cats were enrolled of which 38 could be included in the analysis (questionnaires completed at ≥ 2 timepoints); RAI‐group: *n* = 23, ATD‐group: *n* = 15. HRQoL did not differ between groups at enrolment (RAI: 103.5 [27–211], ATD: 73 [22–260], *P* = 0.22). Overall, the HRQoL improved over time for all 38 cats (*P* < 0.001), but there was no difference between treatment groups (*P* = 0.35). Interestingly, total HRQoL scores decreased (improved) between enrolment and month‐1 in the RAI‐group (enrolment: 103.5 [27–211]; month‐1: 65.5 [33–167]; *P* < 0.001), but not in the ATD‐group (enrolment: 73 [22–260]; month‐1: 80 [11–161]; *P* = 0.89). In the ATD‐group, it took 3 months until HRQoL improvement was achieved (enrolment: 73 [22–260]; month‐3: 31 [11–98]; *P* = 0.02).

In conclusion, HRQoL ameliorates with treatment of hyperthyroidism irrespective of treatment modality. Although RAI was not superior to ATD in improving HRQoL considering the whole 6‐month study period, it did have a significant positive effect on HRQoL within the first month following RAI treatment, indicating faster improvement of HRQoL with curative treatment of hyperthyroidism when compared to ATD. Small sample size, lower number of ATD‐treated cats, and lack of randomization might have impacted the results of this study.

**Disclosures**

Katarina Hazuchova has a consultancy agreement with Boehringer Ingelheim

## ESVE‐P‐11

### ESVE—European Society of Veterinary Endocrinology

#### Hypercortisolism in cats in relation with diabetes mellitus: clinical and laboratory abnormalities, blood pressure and survival under trilostane treatment

##### N López‐Gómez, P García San José, D Pérez Alenza

###### Hospital Clínico Veterinario Complutense, Madrid, Spain

Hypercortisolism (HC) is a rare endocrine condition in cats with few cases described in the literature. It is caused in 80% of the cases by pituitary tumors and ~70%–80% have concurrent diabetes mellitus (DM).

The aims of this study were to describe the clinical and clinicopathological abnormalities in cats with HC and the survival time under trilostane treatment. Also, to assess the prevalence of HC in cats with DM, and to determine which clinical signs and physical examination findings might be suggestive of HC in diabetic cats.

Medical records of cats presenting at a Veterinary Teaching Hospital from January 2014 to December 2022 were retrospectively evaluated.

Six cats were diagnosed with HC 4/6 ACTH‐dependent and 2/6 ACTH‐independent. Median age at diagnosis was 12.5 years, 5/6 were domestic short‐hair and 1/6 Persian; 2/6 were neutered males and 4/6 were females, being 2/4 neutered. Five had an increased body condition score. Most common concurrent conditions were DM (5/6; 83.3%), chronic kidney disease (3/6) and periodontal diseases (3/6). Two cats had hyperthyroidism and acromegaly respectively.

Most frequent clinical abnormalities in cats with HC were abdominal enlargement (6/6), PU/PD (5/6), thin skin (5/6), polyphagia (4/6), hair loss (4/6) and weight increase (3/6). Only 1 cat presented skin fragility. Laboratorial abnormalities more prevalent were lymphopenia (3/5), hyperglycemia (5/6), hyperproteinemia (3/6), hypochloremia (3/6), hypokalemia (3/5), and moderately concentrated urine (< 1.030) (6/6). Prevalence of systemic hypertension (SH; systolic blood pressure ≥160 mmHg) was 50% (3/6). Trilostane treatment was prescribed at a median dose of 0.8 mg/kg/12 h (range 0.3–2.0 mg/kg/12 h). Survival time ranged from 95 to 2063 days (median 193 days); four cats are dead nowadays.

Clinical abnormalities of cats with HC and DM (HC‐DM; 3/5 poorly controlled, 2/5 good controlled) were compared with those of 59 cats with DM evaluated during the same period. Hair loss and weight increase were observed in 60% of cats with HC‐DM but only in 10% and 6.8% non‐hypercortisolemic diabetic cats respectively (*P* = 0.018; *P* = 0.008). Thin skin was observed in all cats with HC‐DM vs. 7.4% of non‐hypercortisolemic diabetics (*P* = 0.000)

In conclusion, prevalence of HC in cats with DM in a referral institution was 7.4%. Weight increase and SH were more common (50%) than usually reported (20%). Not all cats with HC‐DM were poorly controlled as previously described. Clinical signs such as thin skin, increased weight and hair loss might suggest the presence of a concurrent HC in diabetic cats.

**Disclosures**

1. Mª Dolores Pérez Alenza and Paula García San José have financial support of Royal Canin for research in canine Cushing's syndrome. 2. Mª Dolores Pérez Alenza, Paula García San José and Natalia López Gómez have financial support of Nestlé Purina for research in the use of supplements complementary to conventional treatment in animals with endocrine, cardiac or neurological conditions. 3. Mª Dolores Pérez Alenza receive royalties due to her contribution in several books.

## ESVE‐P‐12

### ESVE—European Society of Veterinary Endocrinology

#### Addison Detect: a machine learning predictive model web application to assess canine hypoadrenocorticism likelihood using signalment and routine bloodwork results

##### M Martineau^1^, M Kurtz^2^, M Hebert^3^, R Lavoue^4^, E Krafft^5^, G Benchekroun^2^, A Drut^3^, M Kim^5^, C Pouzot‐Nevoret^5^, E Robin^1^, K Le Boedec^1^

###### ^1^Centre Hospitalier Véterinaire Frégis, Gentilly, France; ^2^Ecole Nationale Vétérinaire d'Alfort, Maisons‐Alfort, France; ^3^Ecole Nationale Vétérinaire de Nantes—Oniris, Nantes, France; ^4^Ecole Nationale Vétérinaire de Toulouse, Toulouse, France; ^5^Ecole Nationale Vétérinaire de Lyon—Vetagrosup, Lyon, France

Canine hypoadrenocorticism (CHA) diagnosis can be challenging as it may mimic many other diseases. Definitive diagnosis requires an adrenocorticotropic hormone stimulation test (AHST) but identifying the dogs needing an AHST can be difficult. This study aimed to develop and validate a machine learning model (called Addison Detect) that predicted CHA probability from signalment and hematology, biochemistry and electrolyte profiles. Data were collected retrospectively in 5 institutions from 504 controls and 68 CHA cases. A parallel random forest algorithm and 10‐fold adaptive cross‐validation were used to develop the model on data from 438 controls and 51 cases from 4/5 institutions. Synthetic Minority Oversampling Technique was used to correct data imbalance. The model performance was assessed on the out‐of‐bag data (internal validation) and the 83 dogs from the 5th institution (external data). On the out‐of‐bag data, Addison Detect had an area under the receiver‐operating‐characteristic curve (AUC) of 0.998 (0.996–0.999) and an optimal threshold of 50% (sensitivity: 98.6% [95.9%–99.5%], specificity: 97.3% [93.6%–98.6%]). The threshold associated with 100% sensitivity was 10%. On external data, Addison Detect had an AUC of 0.942 (0.853–1), sensitivity of 94.1% (71.3%–99.9%), and specificity of 93.9% (85.2%–98.3%) using a 10% predicted probability as the decision threshold. Addison Detect failed to detect one CHA case with sepsis and improperly classified 3 dogs with chronic enteropathy and a dog with chronic kidney disease as CHA cases. Based on these results, Addison Detect appears a useful tool to assess CHA likelihood and identify dogs requiring an AHST.

**Disclosures**

I am not sure to have to report these disclosures. For this study, we obtained a financial support (research fund) of IVC Evidensia for: Obtaining archived files from CHV Frégis for missing data; financial support for congress (transport, accommodation); in the future to pay fees for publication The last author (KLB) owned a website that allows predicted canine hypoadrenocorticism calculation but the website is completely free and is used without registration (so no personal data are entered and therefore can be collected).

**Source of Funding**

Research fund from IVC Evidensia (CHV Frégis)

## ESVE‐P‐13

### ESVE—European Society of Veterinary Endocrinology

#### Longitudinal evaluation of blood pressure in cats treated for hyperthyroidism

##### PA Norman^1^, HM Syme^1^, MD Wallace^2^, LJ Davison^1^, R Chang^1^

###### ^1^Royal Veterinary College, London, UK; ^2^Wellcome Trust Centre for Human Genetics, University of Oxford, Oxford, UK

Hypertension is common in geriatric cats and is often associated with chronic kidney disease (CKD). Hyperthyroid (HTH) cats may be normotensive at diagnosis but develop hypertension with treatment. This study aimed to investigate longitudinal changes in blood pressure (BP) following treatment.

Data from normotensive cats diagnosed with HTH between January 2000 and January 2018 with ≥3 BP measurements over >90 days were compared with previously described populations of healthy‐geriatric cats and cats with CKD. Cats were classified by development of hypertension or azotaemia (creatinine >177 μmol/L). Euthyroidism was determined by a combination of total thyroxine measurement, clinical examination and bodyweight change. Risk factors for developing hypertension were investigated using logistic regression and a time‐dependent Cox regression model.

A total of 4560 visits (median [25th, 75th %] follow‐up 435 [230, 869] days) for 332 HTH cats were analysed; 69 cats became hypertensive and 110 became azotaemic. Proportions of cats developing azotaemia was similar in hypertensive and normotensive groups. Baseline systolic BP was higher in HTH cats developing hypertension than those that remained normotensive (150 [144, 164] vs. 141 [128, 153] mmHg; *P* < 0.001). HTH cats that developed azotaemia were at increased risk of becoming hypertensive (Hazard ratio: 2.74; *P* = 0.0042). HTH cats were significantly more likely to develop hypertension than healthy‐geriatric cats (*P* < 0.001), but not cats with CKD (*P* = 0.995); this risk was not associated with becoming euthyroid (*P* = 0.496).

Development of azotaemia with treatment for HTH increases the risk of developing hypertension. Careful monitoring of BP during treatment is recommended, especially pre‐hypertensive cats at diagnosis.

**Disclosures**

Prof Harriet Syme: This work was not directly funded. Funding from the Evetts‐Luff foundation to support a feline PhD student (Paul Norman) Although they have never directly funded the research we do in hyperthyroidism support for the feline research clinic comes mainly from Royal Canin (Waltham), with additional funding from Idexx, and support for other PhD students from multiple drug companies including Boehringer Ingelheim and Ceva (of note both of these companies market treatments for hypertension) as well as charities such as PetPlan Charitable Trust, PetSavers. In the past year I have been a paid speaker for ESVE, ECVIM, BSAVA modular certificate, ACVIM, Hellenic Veterinary Medical Feline conference, and Renal week as well as receiving funding to support travel for the IRIS board meeting (from Zoetis). Dr. Marsha Wallace is supported by a Clinical Research Fellowship at the Royal Veterinary College. Prof Lucy Davison: Indirect funding supporting current research of LJD (data not presented here): Medical Research Council (Clinician Scientist Fellowship), American Kennel Club Canine Health Foundation, Morris Animal Foundation, UK International Coronavirus Network, Royal Canin, BSAVA PetSavers, UK Research and Innovation, Hong Kong Jockey Club, PetPlan Charitable Trust, Dechra Veterinary Products. Speaker honoraria in past 12 months: ACVIM, Improve International, ESVE Summer School.

**Source of Funding**

Funding from the Evetts‐Luff foundation for PhD studies of Dr Paul Norman.

## ESVE‐P‐14

### ESVE—European Society of Veterinary Endocrinology

#### Feline hypersomatotropism without concurrent diabetes mellitus: 21 cases (2014–2023)

##### D Miceli^1,2^, SJM Niessen^3,4^, JP Rey Amunategui^1^, F Fracassi^5^, GA Pompili^1^, F Tavares^6^, E Molina^1^, R Leal Oliveira^7^, F Zeugswetter^8^, D Bota^9^, A Corsini^10^

###### ^1^Veterinary Science Center, Maimonides University, Buenos Aires, Argentina; ^2^Institute of Experimental Biology and Medicine—CONICET, Buenos Aires, Argentina; ^**3**^Royal Veterinary College, London, UK; ^4^Veterinary Specialist Consultations & VIN Europe, Hilversum, The Netherlands; ^5^Department of Veterinary Medical Science, University of Bologna, Bologna, Italy; ^6^E+VET Clinic, Rio de Janeiro, Brazil; ^7^CIISA—Centre for Interdisciplinary Research in Animal Health, Faculty of Veterinary Medicine, University of Lisbon, Lisbon, Portugal / Associate Laboratory for Animal and Veterinary Sciences (AL4AnimalS); ^8^Department for Companion Animals and Horses, University Clinic for Small Animals, University of Veterinary Medicine Vienna, Vienna, Austria; ^9^AniCura Restelo Veterinary Hospital, Lisbon, Portugal; ^10^Department of Veterinary Medical Sciences, University of Parma, Parma, Italy Feline

Hypersomatotropism (HST) has previously been predominantly characterized by the presence of diabetes mellitus (DM). While in humans most cases do not develop overt DM, in cats only 5 non‐diabetic HST cases have been published to date. This multicentric retrospective case series aims to describe the clinical findings and outcome of non‐diabetic cats with HST. Medical records of non‐diabetic cats with HST admitted between March 2014 and February 2023 to 8 Veterinary Hospitals were evaluated. The diagnosis of HST was based on compatible clinical signs and increased serum insulin‐like growth factor 1 (IGF‐1) concentration (>900 ng/mL). Twenty‐one cats with clinical signs compatible with growth hormone excess and an increased IGF‐1 concentration were included. Eighteen cats were Domestic Short‐Hair, 2 Domestic Long‐Hair, and 1 Norwegian forest. Twenty cats were male (of these 19 neutered) and one cat was a spayed female; median age was 10 years (range 2–15 years); median body weight was 7.3 kg (range 4.2–12 kg). Pituitary enlargement was detected in 9/12 (75%) cats on computed tomography imaging. Sixteen out of 21 (76%) cats had phenotypic changes consistent with acromegaly: prognathia inferior (10/21), broad facial features (8/21), abdominal enlargement (8/21). Additionally, 10/21 cats showed weight gain, 6/21 polyphagia, 6/21 respiratory stridor, 2/21 degenerative arthropathy and 1/21 neurological signs. The main reasons for considering HST were acromegalic phenotype and weight gain. The most relevant clinicopathological abnormalities were: hyperproteinemia (8/21), azotemia (7/21), increased liver enzymes (5/21), decreased urinary specific gravity (5/21), anemia (4/21), hypertriglyceridemia (2/21), and hypercholesterolemia (2/21). Hypertrophic cardiomyopathy phenotype was identified in 8/16 cases. The most common concurrent diseases were chronic kidney disease (7/21) and neoplasia (7/21). Systolic hypertension was reported in 7/10 cases. Seven cats received treatment with cabergoline, one cat underwent hypophysectomy and one cat radiotherapy. Cabergoline normalized IGF‐1 concentrations in 3/7 cases. One cat developed DM after corticosteroid administration post‐radiotherapy. Most non‐diabetic HST cases (76%) were diagnosed on the basis of observed phenotypic changes consistent with growth hormone excess. Cabergoline was not consistently effective to control IGF‐1 excess, though allowed normalization of IGF‐1 concentration in three cases. This study suggests that HST should also be considered in cats without DM. A focus on screening a wider range of presentation types could lead to the uncovering of a larger non‐diabetic HST population among cats.

**Disclosures**

Rodolfo Leal Speaking & Consultancies: Veterinary Information Network (USA), Vet2Vet (IT), Boehringer Ingelheim Andrea Corsini Speaking &amp; consultancies: MYLAV Veterinary Laboratory, Boehringer Ingelheim Stijn Niessen Speaking & consultancies: Boehringer Ingelheim, Dechra Pharmaceuticals, MSD Animal Health, Novartis

## ESVE‐P‐15

### ESVE—European Society of Veterinary Endocrinology

#### Stress or success—hair cortisol concentrations in relation to cat characteristics, health and reproductive status in cats

##### N Rothlin Zachrisson^1^, M Öhlund^2^, H Röcklinsberg^1^, B Ström Holst^1^

###### ^1^Swedish University of Agricultural Sciences, Uppsala, Sweden; ^2^Swedish Medical Products Agency, Uppsala, Sweden

Analysis of hair cortisol concentrations (HCC) is a non‐invasive way to measure cumulative HPA‐axis activity, and is increasingly used in evaluation of chronic stress. The aim with this study was to explore HCC in relation to different characteristics and chronic disease in cats.

Hair from 167 cats, healthy (*n* = 103) or with chronic disease (*n* = 64), was collected from ≥1 location; clipped from the front leg or the abdomen, or collected by combing (representing a full body sample). A questionnaire was sent to the owners, with questions set in retrospective and concerning the cat (e.g., age and sex; neutered females, neutered males, intact females without experience of pregnancy or a period of heat, and females, irrespective of neutering status, that had experienced pregnancy or a period of heat), health status (e.g., chronic disease) and exposure of potential stressors (e.g., house renovation) or the cat exhibiting signs of stress (e.g., house soiling). In cats with ≥1 sample location, results from the location with the lowest HCC was used for analysis. Cortisol concentrations in hair was analyzed with an ELISA (Salimetrics Salivary Cortisol Enzyme Immunoassay Kit) and data were analyzed with multiple linear regression with HCC as outcome. The final regression model was decided with a backwards elimination process combined with a lowered Akaike information criterion, and variables with p >0.05 were excluded. Expected changes in HCC were calculated with 95 % confidence intervals.

Cats with chronic disease had an expected 44% increase in HCC compared to healthy cats (*P* = 0.012, CI 9–92), with chronic disease including one or more of the following diseases; diabetes mellitus (*n* = 28), hyperthyroidism (*n* = 19), chronic kidney disease (*n* = 14) and other (*n* = 14). Further, intact females (*P* = 0.005, CI 23–209) and females that had experienced pregnancy or a period of heat (*P* = 0.036, CI 3–161), had an expected 95% and 64% increase in HCC respectively, compared to neutered females. There were no other significant associations between HCC and cat characteristics, exposure to potential stressors or signs of stress.

Recognizing that although different individual responses to stressors exist, this study presents an association between chronic disease and increased HPA axis activity in cats. Hence, analysis of HCC might have important implications in research and, by extension, in clinical practice, enabling assessment and refinement of disease interventions and management. Associations between HCC and reproductive status in cats proposes both physiological and possibly psychological alternations during different phases of female reproduction.

**Disclosures**

No disclosures to report

**Source of Funding**

The Animal Hospital Foundation in Greater Stockholm, The Fund of Maj Johnson

## ESVE‐P‐16

### ESVE—European Society of Veterinary Endocrinology

#### Evaluation of urinary corticoid‐to‐creatinine ratio using a canine‐specific chemiluminescent cortisol assay (immulite 2000) in the diagnosis of canine hypercortisolism

##### F Del Baldo^1^, A Tirolo^2^, F Dondi^1^, A Sapignoli^1^, M Galeotti^1^, S Segatore^1^, AM Tardo^1^, S Golinelli^1^, F Fracassi^1^

###### ^1^University of Bologna, Bologna, Italy; ^2^University of Parma, Parma, Italy

The urinary corticoid to creatinine ratio (UCCR) is one of the most commonly used screening tests for canine hypercortisolism (HC). Recently, there was a change in the Immulite‐2000‐antibody used for cortisol measurement and new investigations of UCCR are required. This study aimed to establish the reference interval (RI) for the UCCR measured with a chemiluminescent enzyme immunoassay (Veterinary Cortisol, Siemens, IMMULITE 2000 XPi) and to investigate the diagnostic performance of this method for UCCR in canine HC. The UCCR was determined on urine samples from healthy dogs (HD), dogs with HC and dogs with diseases mimicking HC (DMHC). The HD group included dogs with no clinical signs and normal blood tests and urinalysis. Dogs were included in the HC group if they had compatible clinical signs and clinicopathological abnormalities coupled with a positive ACTH stimulation test and/or low‐dose dexamethasone suppression test. The DMHC group included dogs for which HC was suspected based on clinical signs and/or ultrasonographic evidence of an adrenal mass but was subsequently excluded. Dogs were excluded from the study if glucocorticoids were administered in the previous 90 days before testing. The study included 40 HD, 97 dogs with HC and 35 dogs with DMHC. In all HD, urine was collected by free catch at home (AH) and in 26 dogs HD also in the hospital (IH). The RI for UCCR, established on urine collected AH, was between 3 × 10^−6^ (90% CI 2.3–3.8) and 26 × 10^−6^ (90% CI 29.7–35.0). The median (min–max) UCCR results were significantly higher for IH samples (11.7 × 10^−6^; 5.3–45.8 × 10^−6^) compared to those collected AH (8.19 × 10^−6^; 3.9–36.3 × 10^−6^; *P* = 0.03). UCCR in dogs with HC (70.9 × 10−^6^; 6.8–882.2 × 10^−6^) was significantly higher than that HD (9.1 × 10^−6^, 3.9–36.3 × 10^−6^; *P* < 0.001) and dogs with DMHC (15 × 10^−6^, 2.63–137.8 × 10^−6^;*P* < 0.001). The area under the ROC curve for UCCR to differentiate HC dogs from dogs with DMHC was 0.85 (95% CI 0.78 to 0.92). Using as cut‐off value the upper limit of the RI (UCCR > 26 × 10^−6^) the sensitivity and the specificity for the UCCR in detecting HC were 80.4% (95% CI: 71.1–87.8) and 71.4% (95% CI: 53.7–85.4), respectively. In conclusion, we established a new RI for UCCR using IMMULITE 2000 XPi in dogs and we confirmed the importance to collect urine AH to avoid the influence of stress on UCCR results. Using the upper limit of the RI, the sensitivity of this test for diagnosis of HC resulted lower than previously reported. Therefore, UCCR should not be used alone to exclude HC in dogs.

**Disclosures**

Francesca Del Baldo Speaking & consultancies: MYLAV Veterinary Laboratory, Boheringer Ingelheim Federico Fracassi Financial support: Dechra, MSD, Monge. Speaking & consultancies: Boehringer Ingelheim, Dechra, MSD, Royal Canin, Hill's, Nestlé Purina, MYLAV Veterinary Laboratory. Antonio Maria Tardo Speaking & consultancies: Dechra. Stefania Golinelli Financial support: Ph.D. scholarship funded by Dechra Speaking & consultancies: Dechra Francesco Dondi Speaking & consultancies: MYLAV Veterinary Laboratory

**Source of Funding**

European Society of Veterinary Endocrinology Pilot Research Award

**ECVIM CSF Funding**

Yes

## ESVE‐P‐17

### ESVE—European Society of Veterinary Endocrinology

#### Circulating concentrations of betatrophin in dogs with diabetes mellitus

##### A Slon, M Mazaki‐Tovi

###### Veterinary Teaching Hospital, Koret School of Veterinary Medicine, The Hebrew Un Rishon, Le Zion, Israel

Diabetes mellitus (DM) is a common endocrine disorder in dogs. Virtually all dogs are insulin dependent and serum concentrations of triglyceride and cholesterol are typically increased. Betatrophin is a hormone secreted from the liver and adipose tissue, and is involved in glucose and lipid metabolism. It inhibits lipoprotein lipase, its secretion is stimulated by insulin, and it has been associated with β‐cell hyperplasia in insulin‐resistance models. Betatrophin concentrations are increased in both type‐1 and type‐2 human DM, but has not been studied in dogs. We hypothesized that circulating betatrophin concentrations are altered in dogs with DM. Betatrophin and insulin concentrations were measured in surplus serum samples from client‐owned dogs with DM at the time of diagnosis and in healthy dogs using ELISA, and compared by Mann Whitney *U* test. Betatrophin concentrations (median, interquartile range) were lower (*P* = 0.001) in dogs with DM (*n* = 13, 33 pg/mL, 33–45) than in healthy dogs (*n* = 24, 308 pg/mL, 92–635). There was no difference in insulin concentrations between the groups (5.1 mU/L, 3.1–11.3 vs. 6.8 mU/L, 4.9–12.3, *P* = 0.2). Betatrophin concentrations were negatively correlated to glucose (*r* = −0.56, *P* = 0.046), urea (*r* = −0.59, *P* = 0.035) and creatinine (*r* = −0.74, *P* = 0.004) concentrations in dogs with DM, but not in healthy dogs. Serum betahydroxybutyric acid concentrations were elevated in 11/13 of the DM dogs (3.27 mmol/L, 0.86–7.13), but were not correlated to betatrophin or insulin concentrations. This preliminary study showed decreased betatrophin concentrations in dogs with naturally‐occurring DM prior to insulin treatment, potentially in agreement with the stimulatory effect of insulin on betatrophin.

**Disclosures**

No disclosures to report

**Source of Funding**

Department of Small Animal Medicine, Veterinary Teaching Hospital, Koret School of Veterinary Medicine, The Hebrew University of Jerusalem, Rehovot, Israel.

## ESVE‐P‐18

### ESVE—European Society of Veterinary Endocrinology

#### Comparison of symmetric dimethylarginine, plasma aldosterone concentration, renin activity and systolic blood pressure between diabetic and non‐diabetic cats with hypersomatotropism

##### JP Rey Amunategui^1^, G Pompili^2^, J Mas^2^, E Molina^2^, C Figueira Carvalho^3^, D Miceli^1–4^

###### ^1^Veterinary Science Center, Maimonides University, Buenos Aires, Argentina; ^2^Private practice, Buenos Aires, Argentina; ^3^Hospital School of Veterinary Medicine of Sao Paulo University, Brazil; ^4^ Laboratory of Molecular Endocrinology and Signal Transduction, Institute of Experimental Biology and Medicine—CONICET, Buenos Aires, Argentina

Acromegaly in humans is associated with multiple comorbidites, including kidney disease. Growth hormone (GH) and insulin‐like growth factor 1 (IGF‐1) excess may promote changes in plasma aldosterone concentration (PAC) and plasma renin activity (PRA) in acromegalic humans. The impact of hypersomatotropism (HST) in renal function in cats is unknown. The aim of this study was to evaluate symmetrical dimethylarginine (SDMA), PAC, PRA and systolic blood pressure (SBP) in cats with HST and to compare if there are differences between diabetic and non‐diabetic cats with HST. In this prospective study, twenty cats with HST were included. The diagnosis of HST was based on clinical signs and markedly increased serum IGF‐1 concentration (>1000 ng/mL). Serum biochemical analysis, urinalysis, SDMA, PAC, PRA and SBP were evaluated in all cats. Fourteen cats were diabetic and six cats were non‐diabetic. Twelve cats (60%) had concurrent chronic kidney disease (CKD) (8 diabetic and 4 non‐diabetic). Median creatinine concentration in diabetic and non‐diabetic cats with HST were 1.8 mg/dl (0.8–6.4) and 2 mg/dl (1.4–6.3), respectively. Median SDMA in diabetic and non‐diabetic cats with HST were 10.5 μg/dL (10–48.3) and 19.4 μg/dl (10–98.3), respectively. Median PAC in diabetic and non‐diabetic cats with HST were 35 pg/ml (30–399) and 115 pg/ml (30–1065), respectively. Median PRA in diabetic and non‐diabetic cats with HST were 0.6 ngAng/mL (0.1–2.5) and 1.3 ngAng/mL (0.2–3.5), respectively. Six cats showed elevated PAC (>190 pg/ml), however none of these cats met diagnostic criteria for primary hyperaldosteronism. Median SBP was 151 mmg/Hg (131–217), classifying 9 cats as prehypertensive, 4 cats as moderate hypertensive and 3 cats as severe hypertensive. No significant differences were observed comparing creatinine, PAC, PAR, SDMA and SBP between diabetic and non‐diabetic cats with HST. Two cats with CKD achieved diabetic remission (one spontaneously and one treated with cabergoline), and in both cases an increase in SDMA, without changes in creatinine concentration, was observed. This study suggests that CKD is a relatively common comorbidity in cats with HST. Likewise, SDMA seems not be a reliable renal biomarker for diabetic cats with HST, but it could be used for non‐diabetic cats (or cats that achieved diabetic remission) with GH excess. In addition, 35% and 30% of cats with HST have different degrees of hypertension and secondary hyperaldolsteronism, respectively.

**Disclosures**

No disclosures to report

## ESVE‐P‐19

### ESVE—European Society of Veterinary Endocrinology

#### Increased insulin‐like growth factor 1 concentrations in a population of non‐diabetic cats with chronic kidney disease

##### JP Rey Amunategui^1^, E Molina^2^, G Pompili^2^, J Mas^2^, O Pignataro^3^, D Miceli^1–3^

###### ^1^Veterinary Science Center, Maimonides University, Buenos Aires, Argentina; ^2^Private practice, Buenos Aires, Argentina; ^3^Laboratory of Molecular Endocrinology and Signal Transduction, Institute of Experimental Biology and Medicine—CONICET, Buenos Aires, Argentina

Hypersomatotropism (HST) is increasingly recognized as a prevalent endocrinopathy in cats, especially associated with diabetes mellitus (DM). Diabetic cats with HST have higher serum concentrations of creatinine than diabetic cats without HST. In humans, HST is also associated with different comorbidities and most cases do not develop overt DM. The aim of the study was to evaluate circulating insulin‐like growth factor 1 (IGF‐1) in non‐diabetic cats with chronic kidney disease (CKD) and to screen this population for the presence of HST. In this prospective study, one hundred cats diagnosed with CKD at referral centers between June 2021 and March 2023 were included. Serum IGF‐1 was measured as part of the routine tests for CKD. The diagnosis of CKD was based on history, physical exam, systolic blood pressure, clinicopathological findings and renal ultrasonography. The CKD staging was made according to IRIS (International Renal Interest Society) guidelines. Ninety cats were Domestic Short‐Hair, 7 Siamese, 1 Bengal, 1 Burmese and 1 Persian. Fifty‐two cats were spayed female and 48 cats were neutered male; median age was 10 years (range 4–20 years); median body weight was 3.6 kg (range 1.9–8 kg). Median serum IGF‐1 concentrations were 486 ng/mL (range 34–1456 ng/mL). Median serum IGF‐1 concentrations of cats with stage 1 (*n* = 8), 2 (*n* = 58), 3 (*n* = 24) and 4 (*n* = 10) of CKD were 257 ng/mL (range 58–918 ng/mL), 476 ng/mL (range 34–1456 ng/mL), 597 ng/mL (range 123–1055 ng/mL), 571 ng/ml (range 123–948 ng/mL), respectively. There was no correlation between serum IGF‐1 concentrations and creatinine (*r* = 0.16, *P* = 0.09). Four out of 100 cats had IGF‐1 concentrations >1000 ng/mL, resulting in a 4% (95% confidence interval 1.1%–9.9%) HST prevalence rate in non‐diabetic cats with CKD. Twelve cats (12%) had IGF‐1 concentrations between 800 and 1000 ng/mL. Intracranial imaging was performed in the four cats with IGF‐1 concentrations >1000 ng/mL and pituitary enlargement was detected in 1/4 cases on computed tomography. Three of these four cats had phenotypic changes consistent with acromegaly: prognathia inferior (3/3), broad facial features (3/3), abdominal enlargement (1/3) and broadening of paws (1/3). The most common ultrasound findings were bilateral chronic nephropathy (4/4) and adrenomegaly (2/4). A proportion of 4% of non‐diabetic cats with CKD from referral centers had serum IGF‐1 concentration compatible with HST. This study highlights the relevance of screening different sub‐populations of non‐diabetic cats to increase the recognition of HST and to determine the significance of this disease in the cat population.

**Disclosures**

No disclosures to report

## ESVE‐P‐20

### ESVE—European Society of Veterinary Endocrinology

#### Healthy canine and human derived pituitary gland organoids exhibit comparable cellular complexity and hormone secretion

##### K Allenspach^1^, J Chakrabarti^2^, C Zdyrski^3^, O Fasina^1^, A Ralston^3^, AS Little^4^, JP Mochel^1^, Y Zavros^2^

###### ^1^Iowa State University, Ames, USA; ^2^University Of Arizona, Tucson, USA; ^3^3D Health Solutions Inc, Ames, USA; ^4^Department of Neurosurgery, Barrow Neurological Institute, Phoenix, USA

Cushing's disease (CD) is a serious endocrine disorder often caused by an adrenocorticotropic hormone (ACTH)‐secreting pituitary neuroendocrine tumor (PitNET) leading to hypersecretion of cortisol from the adrenal glands in both humans and dogs. Conventional therapeutic drugs used for management of CD including steroidogenesis inhibitors (e.g., trilostane), do not target the PitNET stem cells directly, thus emphasizing that existing medical therapy for CD remains suboptimal in both species. In contrast to genetically modified mouse models, dogs spontaneously develop PitNETs leading to CD that closely recapitulates the clinical, histological and immunohistochemical phenotype of the human disease. Therefore, the objective of these studies was to develop physiologically relevant *in vitro* models of the dog and human pituitary gland.

We generated adult organoid cultures from healthy humans and dogs and compared their cellular complexity and hormone production. Normal pituitary tissues from humans were harvested during transsphenoidal pituitary surgery from patients to generate 3‐dimensional organoid cultures, referred to as human pituitary organoids (hPIT^org^). Pituitary tissues were also harvested post‐mortem from healthy dogs to generate canine pituitary organoids (cPIT^org^). Cellular complexity of hPIT^org^ and cPIT^org^ organoid lines were measured using whole mount immunofluorescence staining, Cytek*™* Aurora advanced spectral flow cytometry system. As a measurement of organoid function, secreted ACTH concentrations were measured by ELISA using collected organoid conditioned media.

Immunofluorescence staining revealed the expression of corticotroph subtype markers ACTH and CAM5.2 in both cPits^org^ and hPits^org^. Flow cytometric analysis revealed the expression of Pit1 cell lineages including cells expressing growth hormone and prolactin, and SF1 lineage expressing follicle stimulating hormone, in both normal hPit^org^ and cPit^org^ lines. ACTH secretion that was measured in the conditioned media collected from hPits^org^ and cPits^org^ were comparable between canine and human organoid lines.

In conclusion, we demonstrate that healthy canine and human derived pituitary gland organoids exhibit comparable cellular complexity and hormone secretion. Our findings support the use of cPit^org^ organoid lines as an advanced preclinical *in vitro* model that recapitulates much of the pituitary gland cell lineages for the study of CD.

**Disclosures**

Karin Allenspach and Jonathan Mochel are co‐Founders of 3D Health Solutions, Inc., a company with the goal to commercialize assays based on organoid technology.

**Source of Funding**

Iowa State University Foundation

## ESVE‐P‐21

### ESVE—European Society of Veterinary Endocrinology

#### Canine hypoadrenocorticism—evaluation of aldosterone concentration and thyroid autoantibodies in dogs

##### S Strey^1^, I Leiter^2^, R Mischke^2^, C Emming^2^, M Schmicke^2^, J Rieder^2^

###### ^1^Small Animal Clinic, University of Veterinary Medicine Hannover, Foundation, Han, Garbsen, Germany; ^2^Small Animal Clinic, University of Veterinary Medicine Hannover, Foundation, Hannover, Germany

The aim of this study was to evaluate aldosterone concentrations and thyroid hormones and antibodies in dogs with hypoadrenocorticism (HA). Dogs were included in the study if HA was confirmed by an ACTH stimulation test between 2019 and 2022. Serum aldosterone, total thyroxine (TT4), thyrotropin (TSH) and thyroglobulin autoantibodies (TgAA) were analysed from preserved samples. Twenty‐nine dogs were included in the study. Hypoaldosteronism (reference range aldosterone: <36 pg/ml) was detected in 59 % (17/29). A sodium‐potassium ratio <27 was seen in all dogs with hypoaldosteronism and in four of the remaining twelve dogs with normal aldosterone. The correlation between potassium and aldosterone concentrations was negative (*r* = −0.6025, *P* = 0.0005), whereas the correlation between sodium and aldosterone concentrations was weakly positive (*r* = 0.3726, *P* = 0.0465). An elevated TSH concentration (reference range TSH: < 5.05 mU/l) could be identified in six dogs while only three of those showed a subnormal TT4 concentration indicating primary hypothyroidism. An elevated TgAA concentration was detected in 14 % (4/29) of the study population. Two of these had concurrently decreased TT4 and increased TSH values which both improved without thyroid hormone substitution. In conclusion, thyroid parameters including TgAA should be interpreted with caution in patients with HA. Aldosterone measurement promises to be a valuable diagnostic tool in clinical practice to decide for treatment protocols or monitoring intervals. Nevertheless, well established test methods and their reference intervals are needed.

**Disclosures**

No disclosures to report

## ESVE‐P‐22

### ESVE—European Society of Veterinary Endocrinology

#### Urinary steroid profiling using liquid chromatography‐tandem mass spectrometry for the diagnosis of canine Cushing's syndrome

##### N Nagata^1^, H Sawamura^2^, K Morishita^2^, K Hosoya^2^, N Sasaki^2^, K Nakamura^2^, M Takiguchi^2^, Y Ikenaka^2^

###### ^1^ Gifu University, Gifu, Japan; ^2^ Hokkaido University, Sapporo, Japan

Although the measurement of serum cortisol by chemiluminescence enzyme immunoassay (CLEIA) is widely used for the diagnosis of hypercortisolism (HC) or Cushing's syndrome in dogs, problems including the necessity for multiple blood collection under stressful conditions and the cross‐reactivity among hormones are present. Therefore, a less invasive and more accurate diagnostic method is needed. The purposes of this study were to develop a urinary steroid profile analysis method using liquid chromatography‐tandem mass spectrometry (LC/MS/MS) and to evaluate the clinical usefulness of the urinary steroid profile.

Sixty‐five healthy dogs and 38 dogs with suspected hypercortisolism were included in this cross‐sectional and prospective study. Residual samples of the supernatant of free‐catch urine collected at home by the owners for routine urinalysis were used. Eleven steroid hormones in urine were determined by LC/MS/MS. The upper limit of reference interval for each urinary steroid to creatinine ratio was established, and the diagnostic performance of each urinary steroid was evaluated.

Five steroid hormones were significantly higher in 14 dogs with HC than in 24 dogs with mimicking HC and 65 healthy dogs. Urinary corticosterone to creatinine ratio had the highest diagnostic accuracy (area under the curve: 0.96). When concentrations of urinary cortisol were compared between LC/MS/MS and CLEIA, there was a significant correlation (*r* = 0.88, *P* < 0.001) but urinary cortisol measured by CLEIA was significantly higher than that measured by LC/MS/MS (*P* < 0.001).

The upper limit of reference intervals of urinary steroids to creatinine ratios were established in this study. The results of this study suggest that urinary steroid profile using LC/MS/MS is a promising tool for the diagnosis of canine HC.

**Disclosures**

No disclosures to report

## ESVE‐P‐23

### ESVE—European Society of Veterinary Endocrinology

#### Normal thyroid values in 3‐week and 8‐week‐old healthy kittens

##### SE Hulsebosch, KM Vernau, SL Marks, W Vernau

###### UC Davis, Davis, USA

Naturally‐occurring hypothyroidism is uncommon in cats; however, it is more commonly reported in kittens than adult cats. Congenital hypothyroidism may be an underrecognized cause of “failure to thrive” in kittens. Normal thyroid values of healthy kittens are unknown, complicating the diagnosis of congenital hypothyroidism.

Blood was collected from healthy 3‐week and 8‐week‐old random‐bred kittens. Kittens were deemed healthy based on unremarkable history, physical examination, CBC, serum chemistry panel, and negative retroviral testing. Serum total thyroxine (T4), free thyroxine (fT4), and canine thyroid‐stimulating hormone (cTSH) were also measured; feline TSH (fTSH) was measured when serum was available. T4 and cTSH were measured by chemiluminescent immunoassay, fT4 was measured by radioimmunoassay, and fTSH was measured by sandwich immunoassay utilizing bulk acoustic wave sensor technology.

Results were available for 61 3‐week‐old kittens: median T4 was 3.46 g/dL (range 1.79–5.28), median fT4 was 2.18 ng/dL (range 0.85–5.13), median cTSH was 0.09 ng/mL (range 0.02–0.38), and median fTSH was 0.12 ng/mL (range 0.05–0.56). Results were available for 76 8‐week‐old kittens: median T4 was 3.81 g/dL (range 1.86–7.07), median fT4 was 3.34 ng/dL (range 1.01–5.67), median cTSH was 0.04 ng/mL (range 0.02–0.15), and median fTSH was 0.04 ng/mL (range 0.02–0.21). Adult feline reference intervals from the same laboratories were T4 (0.69–3.50 g/dL), fT4 (0.78–4.10 ng/dL), cTSH (0–0.38 ng/mL), and fTSH (0.01–0.22 ng/mL).

These data provide useful age‐specific guidelines for assessing thyroid status in kittens.

**Disclosures**

A Truforma machine was loaned by the company for our university's use.

**Source of Funding**

UC Davis Center for Companion Animal Health and the Winn Feline Foundation

## ESVE‐P‐24

### ESVE—European Society of Veterinary Endocrinology

#### Desoxycorticosterone pivalate (DOCP) pharmacodynamics in dogs with hypoadrenocorticism

##### DK Langlois, JM Brudvig

###### Michigan State University, East Lansing, USA

The synthetic mineralocorticoid desoxycorticosterone pivalate (DOCP), which is administered as an approximately once monthly injection, is commonly used to treat aldosterone deficiency in dogs with hypoadrenocorticism. Drug costs are substantial, and recent evidence suggests that manufacturer‐recommended dosing guidelines could result in over‐treatment. Some clinicians utilize lower starting dosages whereas others will attempt interval prolongation. The adequacy of these protocols is generally based upon serum electrolyte concentrations, which are not necessarily indicative of mineralocorticoid adequacy. The primary objectives of this study were to utilize plasma renin activity (PRA) measurements to determine the pharmacodynamics of DOCP in dogs with hypoadrenocorticism.

Dogs with newly diagnosed primary glucocorticoid‐ and mineralocorticoid‐deficient hypoadrenocorticism were treated with either 1.1 or 2.2 mg/kg DOCP administered subcutaneously in randomized fashion. Dogs were re‐evaluated at days 1, 7, 10, 14, 21, and weekly thereafter until either day 63 or until serum electrolyte abnormalities recurred, at which time the study was concluded. Blood samples were collected at each evaluation for determination of serum electrolyte concentrations and PRA. Pharmacodynamic properties as determined by serum electrolyte measurements were compared to pharmacodynamic properties as determined by PRA.

Five dogs with primary glucocorticoid‐ and mineralocorticoid‐deficient hypoadrenocorticism, including 3 dogs treated with 1.1 mg/kg DOCP and 2 dogs treated with 2.2 mg/kg DOCP, have completed this ongoing clinical study. Independent of DOCP dosage, the duration of DOCP action as determined by documenting an increased PRA (reference interval, 0.4–3.7 ng/ml/h) was a median of 3 weeks shorter than when duration of action was determined by documenting hyponatremia or hyperkalemia (*P* < 0.01). A PRA > 3.7 ng/ml/h occurred by day 49 in all 5 dogs. Detailed comparisons cannot be made between the DOCP dosages at the current time, but the two dogs treated with 2.2 mg/kg DOCP had overly‐suppressed PRA (<0.4 ng/ml/h) for over 21 days of the study period despite serum sodium and potassium concentrations remaining within reference intervals.

These initial results highlight potential dangers of relying on serum electrolytes to determine DOCP duration of action as this could result in prolonged periods of mineralocorticoid deficiency during the dosing interval. Furthermore, these results suggest that DOCP dosages of 2.2 mg/kg are too high for most dogs as they result overly‐suppressed PRA for multiple weeks.

**Disclosures**

No disclosures to report

**Source of Funding**

The Stanton Foundation

## ESVIM‐P‐1

### ESVIM—European Society of Veterinary Internal Medicine

#### Characterization of brachycephalic obstructive airway syndrome in cats using barometric whole‐body plethysmography

##### Dr García‐Guasch^1^, Dr Caro‐Vadillo^2^, Dr Montoya‐Alonso^3^, Dr Lin^4^

###### ^1^IVC Evidensia Hospital Veterinari Molins & Hospital Veterinaria del Mar Sant Vicens dels Horts, Barcelona, Spain; ^2^Faculty of Veterinary Medicine, University Complutense of Madrid, Madrid, Spain; ^3^Faculty of Veterinary Medicine, Research Institute of Biomedical and Health Scie, Las Palmas de Gran Canaria, Spain; ^4^National Taiwan University—TACS‐Alliance Research Center, Taipei Taiwan

Brachycephalic obstructive airway syndrome (BOAS) refers to upper airway obstruction (UAO) caused by a combination of anatomical abnormalities that increased airway resistance during inspiration. Barometric whole‐body plethysmography (BWBP) is a non‐invasive pulmonary function test that allows a dynamic study of breathing patterns on unrestrained and unsedated cats. The aim of this study was to confirm the utility of BWBP as a clinical diagnostic test for BOAS in cats.

Forty‐three client‐owned cats were enrolled including 11 clinically affected brachycephalic cats (CABcats), 7 non‐clinically affected brachycephalic cats (NCABcats) and 25 non‐brachycephalic cats (NBcats). BOAS cats included did not have a respiratory issue other than BOAS, NBcats were respiratory disease‐free, and none of the cats had history of cardiac or systemic diseases nor exposure to cigarette smoke.

Cats were placed in the BWBP chamber, and breathing signals were obtained after an adaptation period in a quiet and silent environment. Data were expressed as means ± standard deviations. A *P* < 0.05 was considered statistically significant.

BWBP variables obtained were respiratory rate (RR;[bpm]), tidal and minute volume per kilogram (MV/kg and TV/kg;[mL/kg]), inspiratory (Ti;[s]) and expiratory (Te;[s]) intervals, airway obstruction index enhanced pause (Penh), and peak inspiratory and expiratory flows per kilogram (PIF and PEF;[mL/s/kg]).

Brachycephalic cats showed significantly lower MV/BW (CABcats: 324 ± 120; *P* = 0.028; NCABcats: 320±103; *P* = 0.039) than NBcats (467 ± 141). CABcats had significantly higher PEF/PIF ratio (1.60 ± 0.62) and Penh (6.48 ± 7.53) than NCABcats (PEF/PIF 0.76 ± 0.19; *P* = 0.007; Penh 0.63 ± 0.31; *P* = 0.002) and NBcats (PEF/PIF 0.73 ± 0.11; *P* < 0.001; Penh 0.51 ± 0.13; *P* < 0.001). There were no statistical differences among groups for RR, TV/kg, Ti, and Te.

These results suggested brachycephalic conformation is associated with impaired minute ventilation. The severity of inspiratory airflow limitation and upper airway resistance in BOAS cats could be estimated from PEF/PIF ratio and Penh. As conclusion, it can be stated that BWBP can be used as a clinical tool to characterize the severity of BOAS noninvasively and objectively in cats.

**Disclosures**

No disclosures to report

## ESVIM‐P‐2

### ESVIM—European Society of Veterinary Internal Medicine

#### Dorsomedial nasopharyngeal masses with a benign appearance in dogs: a retrospective study of 99 cases (2019–2022)

##### A Petitpre, P Deprez, A Lamoureux

###### CHV Anicura Nordvet, La Madeleine, France

Dorsomedial nasopharyngeal masses with a benign macroscopic appearance are frequently observed in dogs during rhinoscopy, especially in brachycephalic breeds. However, they are not well‐described in the veterinary literature.

The aim of this study was to characterize those masses; to verify if their frequency was higher in brachycephalic breeds; and to search for any association with anamnestic and clinical data.

Endoscopic images of all rhinoscopies performed between 2019 and 2022 in a private hospital center were retrospectively reviewed. Dogs were included if suitable nasopharynx images were available for review. The medical record of each dog was retrieved. Standard statistical analyses were performed; *P* < 0.05 was considered significant.

Two hundred and twenty‐one dogs were included with 45% of them (99/221) having a nasopharyngeal mass. Masses measured <10%, 10%–30%, and >30% of the nasopharyngeal height in 68% (67/99), 25% (25/99) and 7% (7/99) of cases, respectively. Masses were significantly more common in brachycephalic dogs (54%;51/95) compared to non‐brachycephalic dogs (38%;48/126) (*P* = 0.021). Among dog with masses, brachycephalic dogs were significantly younger (*P* < 0.001) and had more chronic clinical signs (*P* < 0.01) than non‐brachycephalic dogs. In the brachycephalic population, dogs with masses showed more grade two laryngeal collapse (*P* = 0.013) and more sleep apnea (*P* = 0.033) than non‐affected brachycephalic dogs.

This study suggests that dorsomedial nasopharyngeal masses are relatively frequent in dogs, with a possible predisposition of brachycephalic breeds. Their nature and their clinical significance are unknown, but an association with more severe respiratory lesions is suggested in brachycephalic dogs. They could represent undescribed secondary lesions of brachycephalic syndrome.

**Disclosures**

No disclosures to report

## ESVIM‐P‐3

### ESVIM—European Society of Veterinary Internal Medicine

#### Incidence and characterization of aerophagia in dogs using videofluoroscopic swallow studies

##### M Grobman^1^, C Reinero^2^, T Lee‐Fowler^3^, T Lever^3^

###### ^1^Auburn University, Auburn, USA; ^2^Columbia, USA; ^3^USA

Aerophagia, ingestion of air, is a functional aerodigestive disorder that is increasingly recognized in adults and children. Aerophagia, defined in dogs as >1/3 of bolus volume containing air or resulting in gastric distention (>1/3 of end gastric volume), is highlighted during eating/drinking. Videofluoroscopic swallow studies (VFSS) have been used to document aerophagia in dogs, however, the incidence, clinical signs (CS), and associated disorders are unknown. The objectives of this study were to identify the incidence of aerophagia, compare CS between dogs with and without aerophagia, and identify predisposing disorders for aerophagia using VFSS. Fifty‐seven sequential VFSS and associated medical records from dogs presenting to a single university teaching hospital were retrospectively reviewed. Statistical comparisons were made by Mann‐Whitney, Chi‐squared, and Fisher's exact tests, and multiple linear regression (*P* < 0.050). The incidence of aerophagia was 43%. Dogs with mixed (gastrointestinal and respiratory) CS (*P* = 0.013) were more likely to have aerophagia compared to respiratory (*P* = 0.819) or gastrointestinal CS alone (*P* = 0.05). Aerophagia was significantly more common in brachycephalic dogs (*P* = 0.012) and dogs with upper airway obstruction unrelated to brachycephaly, including laryngeal paralysis and nasal masses among others (*P* < 0.001). Both brachycephaly (*P* < 0.001) and upper airway obstruction (*P* < 0.001) were found to be independent predictors of aerophagia. No significant differences in aerophagia were detected between dysphagia groups: oral‐preparatory, pharyngeal, and esophageal (*P* > 0.05). In conclusion, aerophagia was commonly identified, particularly in those presenting with mixed clinical signs. Brachycephalic dogs and dogs with upper airway obstruction should be considered predisposed.

**Disclosures**

No disclosures to report

## ESVIM‐P‐4

### ESVIM—European Society of Veterinary Internal Medicine

#### Spectrographic analysis of lung sound for identifying visual characteristics of crackles in dogs

##### CR Chen^1^, PY Lo^2^, Y Ho^2^, PY Huang^2^, WT Chang^1^, HW Chen^3^, HD Wu^3^, CH Lin^1^

###### ^1^National Taiwan University, 2.TACS‐Alliance Research Center, Taipei, Taiwan; ^2^TACS‐Alliance Research Center, Taipei, Taiwan; ^3^National Taiwan University, Taipei, Taiwan

The detection of adventitious lung sounds is important in screening for cardiopulmonary abnormalities. Spectrographic analysis is a tool to objectively assess the visual characteristics of lung sounds. This study aimed to investigate the distinguishable spectrographic features of crackles in dogs.

Lung sounds were recorded from 33 dogs using a 3M Littmann 3200 digital stethoscope to obtain conventional amplitude‐time waveforms (A‐t‐wav). Fast Fourier transform was performed by freeware software (Kaleidoscope Lite, v.5.4.8) to generate spectrograms with a three‐dimensional perspective (frequency, intensity, and time) of lung sounds. The characteristics of crackles from 18 dogs on spectrograms and A‐t‐wav were compared with normal lung sounds from 15 dogs. Spectral variables were measured and averaged from three consecutive breaths. The accuracy of applying spectrographic and A‐t‐wav features to distinguish crackles from normal lung sounds was assessed by four observers who were blinded to the results of auscultation.

Crackles could be identified on spectrograms by the feature of high‐frequency, short‐duration narrow peaks periodically displayed in every breathing cycle. Compared to normal lung sounds, crackles showed a significantly higher peak frequency (1083 vs. 657 Hz, *P <* 0.001) and maximum intensity values (−32.83 vs. −57.02 dB referenced to background noise, *P <* 0.001) on spectrograms. The typical feature of crackles on A‐t‐wav has been described as multiple baseline crossings, with an initial sharp deflection that gradually widens and an amplitude greater than twice that of background noise in the previous study. There was no quantifiable measure that could be defined on A‐t‐wav to differentiate crackles from normal lung sounds in the study dogs; thus, the qualitative criteria of crackles from previous literature was adopted. By utilizing spectrographic characteristics, the average accuracy among observers for distinguishing crackles from normal lung sounds on spectrograms was the highest (96.9%, range: 96.9%–96.9%). Of notes, the accuracy decreased to 71.8% (range: 64.5%–77.4%) when common artifacts, such as shivering or subtle rubbing, were included in the recording. The worst accuracy of 46.0% (range: 35.5%–51.6%) was obtained when using qualitative features on conventional A‐t‐wav, even without artifacts.

In conclusion, distinguishable features of crackles can be well identified on spectrographic analysis in dogs and could be potentially used for future computerized lung sound analysis. Nevertheless, careful stethoscopic recording without artifacts is important for a successful application.

**Disclosures**

A part of this study was supported by National Taiwan University, the award from TACS‐Alliance Research Center, and the grant from National Science and Technology Council, Taiwan

**Source of Funding**

NSTC 111‐2313‐B‐002‐062‐/TACS‐A 2023 Award / Support from National Taiwan University

## ESVIM‐P‐5

### ESVIM—European Society of Veterinary Internal Medicine

#### Clinical evaluation and endoscopic classification of gastrointestinal findings in 220 dogs with brachicephalic syndrome

##### E Bottero^1^, F Raponi^2^, P Ruggero^2^, G De Cata^2^, U Ala^3^, P Giannella^3^

###### ^1^Endovet Italia, Ceva (Cn) Italy; ^2^Endovet, Roma, Italy; ^3^Università degli Studi di Torino, Torino, Italy

The pathophysiological correlation between the brachycephalic syndrome and the gastrointestinal system has been known for many years. Different grading schemes have been proposed for digestive signs (DS); however, an endoscopic systematic classification is lacking. The aims of this retrospective study were to describe DS; to classify endoscopic gastrointestinal findings (EGF); and to evaluate possible associations among clinical data, endoscopic respiratory findings (ERF) and EGF at diagnosis and after surgery. Information gleaned from the medical records included signalment, body weight, body condition score (BCS), DS, respiratory signs (RS), EGF, ERF, and DS/RS 1 and 6 months after surgery. DS and RS were classified according to the Poncet classification. Video documentation was reviewed to assign a score to each EGF (1 present/0 absent). A total EGF score (0–8) was obtained by the sum of each EGF score. The following EGF were evaluated before intubation (sternal recumbency): alterations compatible with distal esophagitis, cardial atony and sliding hiatal ernia. The following EGF were evaluated after intubation (left lateral recumbency): alterations compatible with gastric and duodenal inflammation and lymphangectasia, mucosal hyperplasia of the antrum and pylorus. In addition, esophageal dilatation/deviation, and presence of food in the stomach were recorded. A total of 220 brachycephalic dogs were included: 176 French Bouledogues, 22 English Bulldogs, 18 Pugs, 4 other brachycephalic breeds. The median age was 24 months (range 2–120), the median body weight was 12.2 kg (range 5–40), the median BCS was 3 (range 2–5). Of the 157 dogs with DS, regurgitation was the most prevalent sign (94 dogs), followed by vomiting (27 dogs), nausea (25 dogs) and diarrhea (11 dogs). All dogs but 11 showed EGF. Esophageal dilatation/deviation, distal esophagitis, cardial atony, axial hiatal ernia, gastritis, mucosal hyperplasia of the antrum and pylorus, food in the stomach, duodenitis and lymphangectasia were found in 166, 167, 62, 51, 150, 40, 77, 47, 130 and 60 dogs, respectively. Total EGF score ≥5 was assigned to 59 dogs. A tendency for dogs with lower BCS to have higher total EGF scores was found (*P* = 0.05586). Significant associations were found between total EGF scores and DS/RS both at diagnosis and follow‐up, total EGF scores and severity of ERF, severity of distal esophagitis and severity of laryngeal granuloma. Based on these results, a standardized endoscopic evaluation of the digestive system could be a useful additional tool to define severity and prognosis of brachycephalic syndrome.

**Disclosures**

No disclosures to report

## ESVIM‐P‐6

### ESVIM—European Society of Veterinary Internal Medicine

#### Heinz Body Formation in 13 Multimorbid Dogs following Metamizole Administration

##### V Geisen, K Hartmann, R Dörfelt

###### Medizinische Kleintierklinik University Munich, Munich, Germany

Heinz Body (HB) anemia, a hemolysis secondary to oxidative damage of erythrocytes, is an uncommon condition in dogs when compared to cats. Metamizole is commonly used as an NSAID in some European countries (e.g., Germany). In humans, metamizole rarely causes gastrointestinal hemorrhage, but HB formation is not described. Aim of this study was to evaluate HB formation in dogs receiving metamizole.

In this retrospective case series, 13 multimorbid dogs were included that developed HBs after receiving metamizole, and clinical features, laboratory values, concurrent diseases, and outcome were described.

In this case series, 10/13 dogs that developed HBs after metamizole administration were older and multimorbid. HBs were detected in 7/13 dogs within few days (3–10 days) after starting metamizole treatment. Highest percentage of HBs detected were 28%–95% (mean 46%) at a dose of metamizole of 38–159 mg/kg/day. There was no correlation between percentage of HBs and daily metamizole dose. All but one dog had a mild to severe anemia at the time of highest HB percentage, but amount of HBs did not correlate with hematocrit. In 8/12 dogs, no stress leukogram was present. About half of the dogs with HBs also had evidence of gastrointestinal hemorrhage, which could have masked hemolysis as cause for the regenerative anemia.

In multimorbid dogs receiving metamizole, HBs leading to hemolysis due to oxidative damage can occur. Thus, multimorbid dogs seem to be predisposed for HB anemia after metamizole application, and in those dogs, metamizole treatment should only be performed with regular CBC controls.

**Disclosures**

No disclosures to report

## ESVIM‐P‐7

### ESVIM—European Society of Veterinary Internal Medicine

#### Root cause analysis of medication‐related incidents in small animal veterinary practices: An Analysis of Incident Reports

##### L Schortz^1^, L Mossop^1^, A Bergström^2^, C Oxtoby^3^

###### ^1^University of Lincoln, Lincoln, UK; ^2^Swedish University of Agricultural Sciences, Uppsala, Sweden; ^3^Veterinary Defence Society, Cheshire, UK

Veterinary care can be complex, and like with any high‐risk organisation, there are risks of unintentional harm. Similar to human healthcare the most frequent type of incidents reported are medication related. There is a need to expand the understanding of what causes are identified to inform improvement activities.

Descriptive statistical analysis was utilized to characterize key features in medication‐related incidents with wrong dose or dosing frequency. The incidents were reported in a company specific incident handling system by 29 small animal practices in Sweden, between April 2018 and February 2023. Content analysis was used to identify groups of causes in the analysis of the incidents.

A total of 474 incident reports were analyzed, of which 65% (*n* = 311) were for dogs and 34% (*n* = 163) for cats. The majority, 77% (*n* = 366) did not result in patient harm. 108 reports were included for content analysis of the root cause. Poor communication, lack of or unclear routines, understaffing, inadequate documentation, impact of stress, lack of knowledge, and insufficient teamwork were groups identified.

These findings suggest that the causes for dosing and frequency‐related medication incidents are multifactorial and complicated.

**Disclosures**

No disclosures to report

**Source of Funding**

This research has come from a PhD study funded by AniCura.

## ESVIM‐P‐8

### ESVIM—European Society of Veterinary Internal Medicine

#### Effects of short‐term oral 25‐hydroxyvitamin D supplementation on leukocyte cytokine production in healthy dogs

##### JA Jaffey^1^, R Kreisler^1^, RC Backus^2^, D Gordon^1^, R Monasky^1^, L Chittick^1^

###### ^1^Midwestern University College of Veterinary Medicine, Glendale, USA; ^2^University of Missouri College of Veterinary Medicine, Columbia, USA

Vitamin D modulates exaggerated pro‐inflammatory cytokine responses in many species, including dogs. To date, research investigating the immunological effects of vitamin D in dogs has been limited to in vitro studies. Therefore, we aimed to determine whether oral supplementation with 25‐hydroxyvitamin (OH)D in dogs affects peripheral leukocyte cytokine production.

Eleven healthy dogs with serum 25(OH)D concentrations of ≤30 ng/mL were included in this randomized, double blinded, placebo‐controlled cross‐over study. Dogs were randomized to receive 25(OH)D at 2.3μg/kg^0.75^ (low‐dose), 25(OH)D at 4.6 μg/kg^0.75^ (high‐dose), or placebo for 7 days and crossed over to alternative treatments following 28‐day washout periods. Serum 25(OH)D was measured with a modified‐HPLC method at baseline (before any treatments), after each treatment (i.e., placebo, low‐dose 25(OH)D, and high‐dose 25(OH)D), and at the end of each washout period. Whole blood cultures were performed at baseline and after each treatment. Cultures consisted of incubating blood with lipopolysaccharide (LPS), lipoteichoic acid (LTA), or phosphate buffered solution (PBS) for 24 h. Tumor necrosis factor (TNF)‐α, interleukin (IL)‐6, and IL‐10 were measured in supernatant using a canine‐specific multiplex assay. Linear mixed models with a random intercept were used to model cytokine concentration for each treatment group while controlling for subject‐specific components of the response and carry effects from the preceding treatment. The relationship between cytokine concentration and 25(OH)D concentration, with independent and dependent variables log transformed for greater normality, was evaluated through mixed effects linear regression.

Both low‐dose (mean, SD; 60 ng/mL, 18) and high‐dose (95 ng/mL, 27) oral supplementation increased serum 25(OH)D concentrations from baseline (24 ng/mL, 7; both *P* < 0.0001). Serum 25(OH)D concentrations for placebo (*P* = 0.005), washout 1 (*P* = 0.001), and washout 2 (*P* = 0.001) were higher than baseline. However, there were no differences in serum 25(OH)D concentrations between placebo, washout 1, and washout 2. Both low‐dose (*P* = 0.005) and high‐dose (*P* = 0.009) oral 25(OH)D supplementation decreased LPS‐stimulated leukocyte production of IL‐6 compared to baseline. No differences were identified with TNF‐α or IL‐10 concentrations in any treatment. Linear regression revealed an inverse association between serum 25(OH)D concentrations and LPS‐stimulated leukocyte production of IL‐6 (*P* = 0.03). For every 20% increase in serum 25(OH)D concentration, LPS‐stimulated IL‐6 concentrations decreased by 2.6%.

Oral 25(OH)D supplementation can substantially improve vitamin D status of dogs in just 7 days while dampening stimulated production of IL‐6, a potent pro‐inflammatory cytokine.

**Disclosures**

No disclosures to report

**Source of Funding**

Intramural grant funding

## ESVIM‐P‐9

### ESVIM—European Society of Veterinary Internal Medicine

#### Serum procalcitonin as a diagnostic biomarker in dogs with bacterial respiratory diseases

##### SJ Viitanen, NM Ninna, MM Rajamäki

###### University of Helsinki, Helsinki, Finland

Procalcitonin (PCT) is a precursor of calcitonin, produced in health by parafollicular cells of thyroid gland and during bacterial infections also by parenchymal organs. In humans, increased PCT identifies patients with bacterial respiratory tract infections and is significantly connected to prolonged hospitalization and increased mortality. Purpose of this study was to investigate serum PCT in dogs with respiratory diseases and to examine, whether PCT can be used as a diagnostic biomarker for canine bacterial pneumonia.

Privately owned dogs diagnosed with bacterial pneumonia (BP, *n* = 25), bacterial tracheobronchitis caused by *Bordetella bronchiseptica* (BB, *n* = 17) and chronic bronchitis (CB, *n* = 20) as well as healthy dogs (*n* = 44) were included. Diagnosis was based on thorough clinical examinations including blood work, thoracic imaging, bronchoscopy and respiratory sample cytology and culture. PCT was measured using an ELISA previously validated for dogs (Canine procalcitonin, BioVendor, Asheville, NC). PCT concentrations between different groups were compared with an ANCOVA‐model.

Serum PCT concentrations in dogs with bacterial diseases (BP mean 51.8 ng/L ± standard deviation [SD] 40.6 and BB mean 61.4 ± SD 35.3 ng/L) were not significantly different when compared to dogs with a non‐bacterial respiratory disease (CB mean 89.7 ± SD 73.5 ng/L) or to healthy dogs (mean 51.0 ± SD 37.5 ng/L, *P* > 0.05 in all comparisons).

These results indicate that despite being a valuable diagnostic, prognostic and follow up marker in humans, serum PCT is not elevated in dogs with bacterial lower respiratory diseases and therefore cannot be used as a diagnostic biomarker in dogs.

**Disclosures**

No disclosures to report

**Source of Funding**

S. J. Viitanen has received financial support in form of salary and material grants from the Academy of Finland for this study and also for other ongoing research work.

## ESVIM‐P‐11

### ESVIM—European Society of Veterinary Internal Medicine

#### Clinical efficacy of feline recombinant interferon‐omega as an additional agent in refractory feline chronic gingivostomatitis

##### BL Su^1,2^, SC Huang^1,2^ TT Kao^3^

###### ^1^Institute of Veterinary Clinical Sciences National Taiwan, University, Taiwan; ^2^National Taiwan University Veterinary Hospital National Taiwan, University, Taiwan; ^3^Animal Technology Research Center, Agricultural Technology Research Institute, Taiwan

Feline chronic gingivostomatitis (FCGS) is a painful and inflammatory oral cavity disease. The pathogenesis of FCGS is currently not clearly understood and is thought to be due to an inappropriate immune response to multifactorial antigens, including viruses. Some cats have little or no improvement after surgical teeth extraction and need further medical treatment for the rest of their life. Several therapeutical strategies are introduced to control refractory FCGS. Steroids are one of the most used drugs. However, the side effects of prednisolone will become severe with time. This study aimed to evaluate the clinical efficacy of a new product of feline recombinant interferon‐omega (rFeIFN‐ω, ATRIron) as an additional agent in the control of FCGS.

Seventeen FCGS cats were enrolled after surgical teeth extraction and still under medical treatment for at least two weeks without improvement in this prospective crossover study. Clinical signs, semi‐quantitative scoring assessments from veterinarians and owners, photographic records of the oral lesion and possible side effects were recorded before (week 0) and 2, 4 and 6 weeks after subcutaneous administration of rFeIFN‐ω. The stomatitis scores of 17 cats were significantly decreased on the 4th and 6th weeks after additional therapy (*P* = 0.0001 and 0.0003). Ten cats (10/17, 58.8%) responded well to treatment that can reduce prednisolone dosage, and two of them were even in complete remission and stopped medical treatment. Seven cats (7/17, 41.2%) presented with moderate or mild remission without reducing prednisolone dosages. No noticeable side effects were noted after six weeks of treatment.

These results demonstrate that rFeIFN‐ω can be an excellent additional agent to control refractory FCGS.

Disclosures

No disclosures to report

Source of Funding

No funding

## ESVIM‐P‐12

### ESVIM—European Society of Veterinary Internal Medicine

#### Determination of the plasma proteome of healthy dogs using Liquid Chromatography—Mass spectrometry

##### PG Doulidis^1^, C Frizzo Ramos^1^, B Kuropka^2^, N Luckschander‐Zeller^1^, A Rodríguez‐Rojas^1^, IA Burgener^1^

###### ^1^Veterinary Medicine University Vienna, Vienna, Austria; ^2^Free University, Berlin, Germany

Bloodwork is a widely used diagnostic tool in veterinary medicine as diagnosis often relies on blood biomarkers. However, biomarkers available in veterinary medicine often lack sensitivity or specificity. Mass spectrometry (MS)‐based proteomics technology has been extensively used in biological fluids and offers great potential for a more comprehensive characterization of the plasma proteome. In this study, we aimed to identify and quantify plasma proteins in a cohort of healthy dogs and compare two techniques for depleting high‐abundance plasma proteins to enable the detection of lower‐abundant proteins. We used surplus lithium‐heparin plasma from 30 healthy dogs representing diverse breeds, separated into five randomized groups of pooled plasma from six individuals each. Depletion of high‐abundance plasma proteins using a commercial kit was compared to depletion with an in‐house method using Blue‐Sepharose, whereas plasma protein without depletion was used as control. Across all samples, the most abundant proteins were apolipoprotein A and B, albumin, alpha‐2‐macroglobulin, fibrinogen beta chain, fibronectin, complement C3, serotransferrin and coagulation Factor V. Neither of the depletion techniques achieved significant depletion of high‐abundance proteins, possibly indicating low specificity for canine proteins. However, the two different depletion methods showed substantial differences in the fold‐change of many proteins. Our results suggest that although the abundance of various proteins can be significantly different, these depletion methods do not contribute to the determination of higher number of proteins in the canine plasma. Determining the healthy canine proteome will be the first step in associating diseases with plasma proteomic patterns and contribute to individualizing the therapeutic approach for each patient.

**Disclosures**

No disclosures to report

## ESVIM‐P‐13

### ESVIM—European Society of Veterinary Internal Medicine

#### Pharmacodynamics of Standard and Low Dose Once Daily Esomeprazole in Healthy, Medium to Large Breed Dogs

##### AL Ostronic^1^, S Zhang^2^, CL Gremillion^1^, JM Steiner^3^, KM Tolbert^3^, EN Gould^4^

###### ^1^School of Veterinary Medicine & Biomedical Sciences, Texas A&M University, College Station, USA; ^2^UT Southwestern Medical Center, Dallas, USA; ^3^Gastrointestinal Laboratory, Texas A&M University, College Station, USA; ^4^Gastrointestinal Laboratory, Texas A&M University, College Station, USA

Esomeprazole, the active and presumed more potent formulation of omeprazole, is a proton pump inhibitor (PPI) used for reduction of gastric acid secretion in dogs. However, esomeprazole is cost‐prohibitive for some patients, especially larger dogs, and has the potential for gastrointestinal (GI) adverse events (AEs) with standard, twice‐daily dosing. If found equally efficacious, a reduction in esomeprazole dose or dosing frequency would be advantageous. In humans, therapeutic efficacy is determined pharmacodynamically by the mean percentage time (MPT) the intralumenal gastric pH is >3 or 4 (MPT3 or MPT4, respectively) during treatment. Our study aim was to compare the efficacy of a once daily lower (0.5 mg/kg) versus standard (1 mg/kg) oral dose of esomeprazole in healthy, larger breed dogs. A secondary aim was to identify differences in AEs between doses.

Healthy dogs (*n* = 9) of >1 year of age and > 20 kg body weight were enrolled in a prospective, randomized, double blinded, crossover study. Continuous pH capsules were adhered to the gastric mucosa of each dog with radiographic guidance, and intragastric pH was continuously recorded for up to 6 days. Esomeprazole (0.5 or 1 mg/kg) was administered by mouth on days 2–6 of each treatment arm. Owners scored the daily severity of GI AEs (e.g., fecal scores, the presence of vomiting, and hyporexia). A multivariate mixed effects model was used to assess differences in the MPT3 and MPT4, and fecal scores, between treatments across time (SAS 9.4 statistical software).

No significant differences in the MPT3 or MPT4 were identified between treatments for any time point (*P* > 0.05). Both esomeprazole doses significantly increased intragastric pH on days 2 and 3, compared to pre‐treatment (*P* = 0.0008). Six (66%) and two (22%) dogs achieved the MPT efficacy pH goals established in humans for either both doses, or only a 1 mg/kg dose, respectively. One dog failed to reach these goals regardless of dose. While not statistically significant, AEs occurred only with a 1 mg/kg dose (*n* = 2 dogs), and included diarrhea, vomiting, and hyporexia.

Both doses of esomeprazole were equally effective in raising intragastric pH when given orally once daily to healthy dogs. While AEs were uncommon, a dose reduction was successful in eliminating the GI AEs seen with a standard dose. As the human efficacy goals were achieved in only a subset of dogs, further studies are necessary before once daily dosing of esomeprazole can be used for routine administration.

**Disclosures**

No disclosures to report

## ESVIM‐P‐16

### ESVIM—European Society of Veterinary Internal Medicine

#### Current epidemiology and therapeutic trends of feline chronic rhinitis—a retrospective study

##### R Gaspar^1^, MJ Dias^2^, G Vicente^1^, R Noiva^1^, R Oliveira‐Leal^2^

###### ^1^Veterinary Teaching Hospital, Fac. Vet. Med., ULisboa, Lisboa, Portugal; ^2^Center for Interdisciplinary Research in Animal Health, Fac. Vet. Med., ULisboa, Lisboa, Portugal

Chronic rhinitis (CR) is one of the most common causes of nasal disease in cats. Clinical presentation can be indistinguishable from nasal tumors (NT) and definitive diagnosis relies on histopathology of nasal biopsies. Studies focusing on epidemiological characterization of these nasal disorders are scarce, requiring an updated revision.

This study aims to assess signalment and clinical findings of cats with CR and NT and evaluate the current therapeutic trends of feline CR.

A cross‐sectional retrospective study of feline cases submitted for rhinoscopy and/or blinded nasal biopsies due to chronic nasal signs (>10 days) in a veterinary teaching hospital between January 2018 and December 2021 was conducted. A total of 50 cats were included and divided into two groups according to final diagnosis: CR or NT. Cats with nasopharyngeal polyps, oronasal fistula, foreign bodies, and stenosis were excluded. Age and clinical signs were compared in‐between groups using Mann‐Whitney and Chi‐Square test, respectively (*P <* 0.05 considered significant). When available, information concerning the medical management of CR was further detailed using descriptive statistics.

A total of 50 cases were recruited; CR cases totaled 28/50 (56%) and 22/50 (44%) were NT cases. Median age at diagnosis was 6.5 years (range: 10 months to 17 years) for CR and 11 years (range: 2–15 years) for NT. Nasal discharge and sneezing were the most reported clinical findings, being present in 35/50 (70%; 19 CR versus 16 NT) and 30/50 (60%; 17 CR vs. 13 NT), respectively. There was no significant difference between groups concerning age (*P* = 0.08), presence of nasal discharge (*P* = 0.76), or sneezing (*P* = 0.9). Among CR cases, 24/28 (86%) were classified as lymphoplasmacytic while 4/28 (14%) were neutrophilic. Concerning NT, 7/22 (32%) were lymphoma while 15/22 (68%) were non‐hematopoietic tumors (12 carcinomas, 1 sarcoma, and 2 melanomas). Information regarding medical treatment was available in 19/28 (68%) of CR cases, of which 15/19 (79%) received steroids combined with antibiotics, 3/19 (16%) only received steroids (oral ± inhaled), and 1/19 (5%) only received antibiotic treatment. Among those receiving antibiotics (16/19; 84%), doxycycline was prescribed in 7/16, combined doxycycline and azithromycin in 5/16, potentiated amoxicillin in 3/16, and marbofloxacin in 1/16.

This study reinforces the overlap of age and clinical findings between CR and NT, being both similarly prevalent in cats with chronic nasal signs. Lymphoplasmacytic rhinitis were overrepresented among CR cases. More than three‐quarters of CR cases were treated with combined prednisolone and antibiotics, being doxycycline the most frequently prescribed.

**Disclosures**

Leal, R. O.: Speaking & consultancies: Veterinary Information Network (USA), Vet2Vet (IT), Boehringer Ingelheim, Dechra (PT).

**Source of Funding**

This work was supported by FCT—Fundação para a Ciência e Tecnologia IP, grant UIDB/00276/2020 and by LA/P/0059/2020—AL4AnimalS

## ESVIM‐P‐17

### ESVIM—European Society of Veterinary Internal Medicine

#### Owners’ perspective about the use of mirtazapine transdermal ointment in cats—a survey‐based study

##### S Carvalho^1^, B Mendoza^2^, MJ Dias^3^, I Tirelli^4^, A Corsini^4^, R Oliveria‐Leal^3^

###### ^1^Veterinary Teaching Hospital, Fac. Vet. Med., ULisboa, Lisboa, Portugal; ^2^IVC Evidensia, Bom Jesus Veterinary Hospital, Braga, Portugal; ^3^Centre for Interdisciplinary Research in Animal Health, Fac.Vet.Med., ULisboa, Lisboa, Portugal; ^4^Department of Veterinary Medical Sciences, University of Parma, Parma, Italy

Mirtazapine is a tetracyclic, noradrenergic and specific serotonergic antidepressant used as an appetite stimulant in cats. Despite its historical off‐labeled oral administration in cats, it was recently licensed for use as a transdermal ointment in this species. Being a topical product, it requires a good compliance for success. To the authors knowledge, little is known about owners’ feedback on this therapeutic option. This study aims to evaluate the owners’ perspective about the use of mirtazapine transdermal ointment in cats.

A cross‐sectional survey‐based study was conducted. An online survey with 14 multiple‐choice and open‐ended questions was developed and uploaded using an electronic platform (Google Forms). Questions focused on efficacy feedback, side‐effects and overall perception of mirtazapine ointment. Electronic medical records of two European Veterinary Teaching Hospitals were searched for cats in which mirtazapine ointment was prescribed from March 2021 to January 2023. Owners were contacted by e‐mail, asking to fulfill the survey. Results were detailed with descriptive statistics.

From 100 contacted owners, 69 answers were obtained. The topical application was found easy by 67/69 (97%) of the owners. Those that considered it difficult mentioned cat's behavior and ears debris as the main causes. Following the manufacturer instructions, 63/69 (91%) owners confirmed the application in alternating ears, contrasting to 6/69 (9%) which admitted applying always in the same ear. Side effects were reported by 14/69 (20%), being the most common: vocalization (5/14, 36%), erythema (4/14, 29%) and agitation (4/14, 29%). Most of the respondents (53/69; 77%) considered that transdermal mirtazapine efficiently stimulates the appetite. A total of 9/69 (13%) owners had previously administered oral mirtazapine formulation to their cats before trying the transdermal option. While 7/9 (78%) considered transdermal more beneficial due to an easier administration, the remaining (2/9, 22%) preferred oral mirtazapine due to its lower cost. Reasons for mirtazapine prescription were detailed in 56 answers, being the most common: chronic kidney disease (18/56; 32%) and chronic enteropathy (12/56; 21%). Length of therapy was variably described by owners, being mirtazapine most frequently applied during 7 days (18/69, 26%) or 14 days (12/69, 17%).

This study highlights the owners’ perception on transdermal mirtazapine use, reinforcing its applicability and effectiveness as an appetite stimulant in cats. Chronic kidney disease was the most frequent cause for its prescription. Side effects were reported by a minor percentage of owners and were mild, supporting the safeness of this formulation.

**Disclosures**

Leal, R. O.: Speaking & consultancies: Veterinary Information Network (USA), Vet2Vet (IT), Boehringer Ingelheim, Dechra (PT). Corsini, A.: Speaking & consultancies: MYLAV Veterinary Laboratory, Boehringer Ingelheim.

**Source of Funding**

This work was supported by FCT—Fundação para a Ciência e Tecnologia IP, grant UIDB/00276/2020 and by LA/P/0059/2020—AL4AnimalS.

## ESVIM‐P‐18

### ESVIM—European Society of Veterinary Internal Medicine

#### Outcome and prognostic factors in dogs diagnosed with immune‐mediated thrombocytopenia in Ireland (2009–2020)

##### E López Bailén^1^, A Duclos^2^, D Mullany^2^, K Le Boedec^3^, B Cuq^2^

###### ^1^Langford Vets, Small Animal Referral Hospital, University of Bristol, Bristol, UK; ^2^UCD School of Veterinary Medicine, Dublin, Ireland; ^3^Centre Hospitalier Vétérinaire Frégis, Arcueil, France

Immune‐mediated thrombocytopenia (IMT) is associated with premature destruction of platelets, resulting in severe and potentially fatal bleeding. Presence of melaena, increased blood urea nitrogen (BUN) concentration, or blood transfusion requirement at presentation were previously identified as negative prognostic factors. The objectives of this study were to describe the outcome and potential prognostic factors in dogs diagnosed with IMT in a veterinary referral hospital in Ireland (2009–2020).

Fifty‐two dogs were diagnosed with IMT in this single‐centre, retrospective study. Short‐ and long‐term survivals were analysed using Cox proportional‐hazards regression models.

Non‐associative IMT was diagnosed in thirty‐eight dogs (73%). Associative IMT was diagnosed in twelve dogs (23%), with six (50%) being associated with neoplasia. Melaena and hyperbilirubinaemia were negatively associated with survival [hazard ratio (HR) 2.02, 95% confidence interval (CI) 0.91–4.44, *P* = 0.08 and HR 3.99, 95% CI 1.11–14.32, *P* = 0.03, respectively]. Thirteen dogs (25%) required the addition of a second immunosuppressive drug, which was significantly associated with longer hospitalisation (*P* = 0.002). Vincristine administration (15 dogs; 28.8%) did not significantly impact hospitalisation duration or survival. The median survival time was 1084 days. Survival rate was 69% at 2 weeks [95% CI 55%–80%], 61% at 3 months [95% CI 47%–73%], and 52.7% at 2 years [95% CI 38%–65%].

This is the first study investigating long‐term outcome in dogs diagnosed with IMT in Ireland. Increased BUN, anaemia, blood transfusion requirement or vincristine administration did not have a significant impact on hospitalisation time or survival. Presence of melaena and hyperbilirubinaemia was negatively associated with survival.

**Disclosures**

No disclosures to report

## ESVIM‐P‐19

### ESVIM—European Society of Veterinary Internal Medicine

#### Evaluation of association between hemoparasite infection risk and blood phenotype, gender and breed in potential feline blood donors of portugal and spain

##### R Pimenta da Silva, R Ferreira, I Mesa‐Sanchez, I Cardoso, L Rocha, B Correia

###### Banco de Sangue Animal, Porto, Portugal

Emerging data in human medicine suggests an association between blood type and the susceptibility to certain microorganisms, such as Plasmodium falciparum, Helicobacter pylori and SARS‐CoV‐2. An association between ethnic groups and infectious diseases has also been suggested. In cats, blood phenotypes A, B or AB do not seem to predispose cats to seropositivity for feline coronavirus.

To determine if there is a relationship between hemoparasite infection risk and blood phenotype, gender or breed in a population of potential feline blood donors in Portugal and Spain.

All potential feline blood donors from a veterinary blood bank in Portugal and Spain who underwent analysis for infectious agents (*Feline Immunodeficiency Virus*, *Feline Leukemia Virus*, *Mycoplasma* spp. *and Bartonella henselae*) between January and December 2022 were considered. Three possible associations were investigated: (1) presence of infectious agents and blood phenotype; (2) presence of infectious agent and gender; and (3) presence of infectious agents and breed. Statistical analysis was performed using the SPSS 28.0 software. The Chi‐square test was applied.

A total of 6371 potential blood donors were considered. (1) 6202 cats were type A, 169 were type B and 28 were type AB. 491 cats were positive to some infectious agent; of them, 479 were type A, 11 were type B and 1 was type AB. No association was found between the blood type and the presence of infectious agents (*P* > 0.05). (2) 265 males and 221 females were positive to some infectious agent. An association was found between the gender and the presence of infectious agents *X* ^2^(3) = 12.378; *P* = 0.06. (3) 8 breeds were tested. An association was found between the breed and the presence of infectious agents with *X* ^2^ (21) = 41.721; *P* = 0.05. Domestic shorthair cats were the group that showed biggest susceptibility to the presence of hemoparasites (*n* = 369).

The results obtained in this research suggest that the presence of infectious agents in potential feline blood donors is influenced by the gender and breed, while it is not influenced by the blood phenotype.

**Disclosures**

No disclosures to report

## ESVIM‐P‐20

### ESVIM—European Society of Veterinary Internal Medicine

#### Prevalence, age and disease associations in Yorkshire terriers with increased blood urea nitrogen

##### A Álvarez^1^, M Suárez^2^, J Puig^3^, Y Fernández^4^

###### ^1^AniCura Imavet Referencia Veterinaria, Santiago de Compostela, Spain; ^2^Rof Codina Veterinary Teaching Hospital, Lugo, Spain; ^3^AniCura Ars Veterinaria, Barcelona, Spain; ^4^AniCura Imavet Referencia Veterinaria, Santiago de Compostela, Spain

Anecdotally, Yorkshire terriers have been said to have a higher incidence of increased blood urea nitrogen (BUN), often associated with a normal creatinine, in comparison with other breeds. This abnormality might suggest a breed‐related finding but there is limited information in the veterinary literature about this finding.

The aim of this study was to determine the prevalence of increased BUN with normal creatinine in a referral population of Yorkshire terriers along with its age and disease associations.

The medical records of Yorkshire terriers, which had a serum biochemistry panel performed by the same analyser (Idexx Catalyst One) including at least BUN and creatinine were retrospectively reviewed. Data collection included the following: age, dietary history, non‐steroidal anti‐inflammatory drugs (NSAID) or glucocorticoids administration and final diagnosis.

Three hundred Yorkshire terriers were included. An elevated BUN was observed in 77/300 dogs (25.7%) and 57/300 (19.0%) dogs had an elevated BUN without an increased creatinine (EBUN). The median BUN and creatinine in EBUN dogs were 34 mg/dL (range: 28–132 mg/dL; reference range: 7–27 mg/dL) and 0.9 mg/dL (range: 0.2–1.6 mg/dL; reference range: 0.5–1.6 mg/dL), respectively. There were 223/300 dogs (74.3%) without elevated BUN and creatinine (NBUN). EBUN dogs were significantly older than NBUN dogs (median: 10 years vs. 8 years; *P* < 0.01). The age distribution in EBUN dogs was as follows: <1 year: 7/57 (12.3%), 1–5 years: 6/57 (10.5%), 5–10 years: 15/57 (26.3%) and >10 years: 29/57 (50.9%). The dietary history was available in 26/57 (45.6%) EBUN and 98/223 (44.0%) NBUN dogs, respectively. There was no statistically significant difference between the number of EBUN and NBUN dogs eating a commercial or home‐made diet (*P* = 0.519). The previous administration of NSAIDs or glucocorticoids was known in 45/57 (78.9%) EBUN and 171/223 (76.7%) NBUN dogs, respectively. They were administered to 18/45 (40.0%) EBUN and 44/171 (25.7%) NBUN dogs, respectively. There was no statistically significant difference between the number of EBUN and NBUN dogs receiving or not receiving NSAIDs or glucocorticoids (*P* = 0.06). The most frequent complaints in EBUN dogs included neurological (13/57; 22.8%); orthopaedic (10/57; 17.5%) and oncological (9/57; 15.8%) diseases. In NBUN dogs included neurological (53/223; 23.8%); gastrointestinal (38/223; 17.0%) and orthopaedic (32/223; 14.3%) diseases.

Approximately, one in five Yorkshire terriers had an increased BUN without a concurrent increased creatinine. Half of these dogs were older than 10 years and the most common complaints included neurological, orthopaedic and oncological diseases.

**Disclosures**

No disclosures to report

## ESVIM‐P‐22

### ESVIM—European Society of Veterinary Internal Medicine

#### Clinical and imaging findings, bacterial isolates and antimicrobial resistance in 14 dogs with tracheal and/or bronchial collapse: a retrospective study of 14 cases

##### N Mateu^1^, A Rodriguez‐Cobos^1^, M Labayru^1^, M Molinero^2^, S Atencia^1^

###### ^1^AniCura VETSIA Veterinary Hospital, Madrid, Spain; ^2^IDEXX Laboratories, Barcelona, Spain

Collapse of the tracheo‐bronchial tree is a common cause of cough in dogs. The most reliable method for diagnosis is bronchoscopy. However, studies examining the clinicopathological, imaging findings and the micro‐organisms involved in this process are still scarce.

The aim of this retrospective study is to describe the clinicopathologic abnormalities, radiographic findings, principle bacteria involved, and antimicrobial susceptibilities in a population of dogs with tracheal and/or bronchial collapse from a referral hospital in Spain.

A total of 14 dogs referred between 2017 and 2022 were included. All patients met the inclusion criteria: diagnosis of tracheal and/or bronchial collapse through bronchoscopy and a history of chronic coughing.

Out of the 14 dogs included in the study, 71% (10/14) were small breed (<10 kg) and 79% (11/14) were ≥7 years old. The most frequently reported breed in this study was the Yorkshire terrier (YST) (6/14, 43%).

Leukocytosis with neutrophilia was the most common clinical pathology finding (6/14), followed by mild to severe increases in alkaline phosphatase (6/14) and alanine aminotransferase (6/14) activities. Cytology of the bronchoalveolar lavage fluid (BALF) was consistent with a septic inflammatory process with the presence of intracytoplasmic microorganisms in 57% (8/14) of dogs.

The most common radiological finding was generalised bronchointerstitial pattern (4/14, 33% of the cases), followed by alveolar pattern (3/14, 21%). However, 33% of the patients showed no radiographic alterations.

Bronchoscopy showed tracheo‐bronchial collapse in 6 dogs, tracheal collapse in 4, and bronchial collapse in the other 4 dogs. Left sided bronchial collapse was more frequently seen (9/10) than right cranial bronchial collapse (4/10). Collapse of the left caudal bronchus was detected in 78% of the patients, and 58% dogs had collapse of the left main bronchus.

*Bordetella bronchiseptica* spp. and *Mycoplasma* spp. real time‐PCRs were positive in 2/9 and 2/9 of patients respectively.

As reported in previous studies, the most frequent bacteria isolated in this study was *Escherichia coli (3/14). Enterococcus* spp. was also frequently isolated in the present study (3/14); 100% of *E. coli* and *Enterococcus* spp. were susceptible to amoxicillin‐clavulanate.

Eleven of the patients had received antibiotics prior to referral and, despite this, BALF culture was positive in 13/14 of the patients, which justifies performing BALF cultures in patients who do not respond to antibiotic treatment. The only patient with a negative BALF culture was receiving antibiotics at the time the BALF was performed, which could justify this result.

**Disclosures**

M. Molinero works at IDEXX laboratories Internal Medicine department.

## ESVIM‐P‐23

### ESVIM—European Society of Veterinary Internal Medicine

#### Clinical manifestations, endoscopic findings and outcome of palatine tonsil crypt foreign bodies in dogs: 7 cases (2020–2022)

##### B Boot, A Lamoureux, A Petitpre

###### Small Animal Hospital Anicura Nordvet, Plouvain, France

Palatine tonsils are a common location of oral foreign bodies (FB) in humans. Dogs have unique anatomic particularities, with the presence of palatine tonsils crypts. FB in this localization have rarely been described with only two cases reported; both presented for unrelated clinical signs.

The aim of this case series was to describe the clinical manifestations, endoscopic findings and outcome of FB in palatine tonsils crypts of dogs.

Medical records of dogs who had FB removed from their palatine tonsils crypts were reviewed, and data were extracted.

Seven dogs were included. Throat clearing was the most common clinical signs reported (7/7), followed by vomiting (3/7), ptyalism (2/7) and dysphagia (1/7); with an acute onset in all. Lesions of the palatine tonsils were observed in 5/7 dogs. Foreign bodies were removed under endoscopic guidance in all dogs; a vegetal FB was found in all but one (6/7). Complete resolution of clinical signs was reported in the five dogs for which follow‐up information was available.

To the best of our knowledge, this case series is the first one to describe the clinical presentation and outcome of dogs with palatine tonsils crypt FB. Palatine tonsils crypts are a rare but possible localization for FB in dogs and should be explore even in the absence of macroscopic lesions of the tonsils, since some dogs could have normal palatine tonsil appearance. Acute throat clearing should raise the suspicion for palatine tonsil crypt FB. The prognosis seems good after removal.

**Disclosures**

No disclosures to report

## ESVNU‐P‐1

### ESVNU—European Society of Veterinary Nephrology and Urology

#### Capillary electrophoresis in urine from healthy cats: comparison with canine patterns

##### L Gil^1^, M Wsol^2^, PF Navarro^2^, S Fernández‐Barredo^3^

###### ^1^Universidad Católica de Valencia San Vicente Mártir, Valencia, Spain; ^2^Universidad Católica de Valencia San Vicente Mártir, Valencia, Spain; ^3^Laboratory CEDIVET, Valencia, Spain

Few studies have been done to evaluate the patterns obtained by Capillary Electrophoresis (CE) in healthy cats, and only one publication describes range references and patterns in healthy dogs.

The aim of this preliminary study is to compare the patterns obtained by Capillary Electrophoresis (CE) in urine from healthy cats and dogs.

Samples from 14 healthy cats were collected from 2020 to early 2022 and prepared for CE using protein dialysis columns. After dialysis, sample migration was conducted in Minicap Sebia equipment, using a diluted feline serum as a control curve. The obtained curves were compared to those obtained previously from 123 dogs in an earlier publication. The Anderson‐Darling test was selected to assess the normality hypothesis, whereas a comparison of equality of variances was performed using the ANOVA test.

Urinary capillary electrophoresis was carried out and the result was obtaining an electrophoretic curve, that was divided into five fractions F1 (albumin), F2 (alpha1‐globulins), F3 (alpha2‐globulins), F4 (beta‐globulins) and F5 (gammaglobulins). The comparison of the urinary proteinograms between healthy animals of the feline and canine species showed statistically significant differences in the fractions F2 (alpha1‐globulins), F4 (beta‐globulins) and F5 (gammaglobulins). The F2 and F5 were decreased in the cats' group compared to dogs, which could be associated with lower Urine Protein Creatinine Ratio (UPCR) values detected in cats. Regarding the increase in F4 in cats compared to dogs, it can be associated with a greater excretion of uromodulin in cats, which has a protective effect at the urinary level. The results of this preliminary study, point out significant differences between the fractions observed in the urine of healthy dogs and cats. Whether these differences are due to the presence of distinct proteins or different quantities of the same proteins, remains unclear. More animals and ancillary techniques are necessary to answer the questions raised considering these variations.

**Disclosures**

No disclosures to report

**Source of Funding**

This work was supported by Universidad Católica de Valencia San Vicente Mártir (grant UCV 2016‐226‐001/UCV 2018‐226‐001).

## ESVNU‐P‐2

### ESVNU—European Society of Veterinary Nephrology and Urology

#### One‐step, non‐surgical placement of permanent low‐profile cystostomy tubes in nine dogs

##### C Lea, D Kelly

###### Southern Counties Veterinary Specialists, Ringwood, UK

Low‐profile cystotomy tubes have typically been placed during coeliotomy or have been used as replacement tubes in dogs with an existing stoma.

Medical records of nine dogs in which non‐surgical placement of a low‐profile cystostomy tube was attempted were reviewed. In all cases, a MIC‐KEY G Tube was used, with an accompanying graduated peel‐away dilator.

Four dogs had detrusor‐urethral dyssynergia, four had obstructive neoplasia and one dog had degenerative lumbosacral disease. Tube placement was successful in 7/9 cases. The two cases in which tube placement was unsuccessful required conversion to coeliotomy to repair the bladder defect and to place the tube. Mean duration that tubes were in place was 17 months (range 4 days to 38 months). Three dogs were euthanised with their cystotomy tubes in place. The remaining four dogs regained normal urinary tract function, therefore no longer necessitating the need for a cystotomy tube. Tube replacement was required in 6/7 dogs, with two dogs requiring two replacements and one dog requiring three replacements. Reasons for replacement were inadvertent tube removal by the patient (33%), breakage of the tube considered a consequence of repeated use (33%), deflation of the balloon (22%), and excess tissue around the stoma site (11%). Mean time until first tube replacement tube was 116 days. During follow up, urine culture was performed in three dogs. Culture was positive in all three. Leakage of urine from the newly created stoma in the absence of a cystopexy did not appear to be a problem in any dog.

Non‐surgical placement of low profile cystotomy tubes is a feasible, less invasive alternative to surgical placement. Although, conversion to surgery may be required if non‐surgical placement is unsuccessful. Complications such as inadvertent tube removal are encountered following both surgical and non‐surgical placement. The lack of cystopexy in non‐surgical placement does not appear to be problematic.

**Disclosures**

No disclosures to report

## ESVNU‐P‐3

### ESVNU—European Society of Veterinary Nephrology and Urology

#### Comparison between urine protein‐creatinine ratios of samples obtained from healthy cats at home and in hospital

##### P Liu^1^, H Hsiao^1^, B Su^2^

###### ^1^National Chung Hsing University, Taichung, Taiwan; ^2^Institute of Veterinary Clinical Sciences, National Taiwan University, Taipei, Taiwan

The urine protein‐creatinine ratio (UPC) is used to quantify and monitor proteinuria in small animal. Setting and related circumstance of urine collection in dogs was reported with higher UPC which could be highly associated with stress. Up to now, no similar findings in healthy dogs and cats were reported. Cats are very sensitive creatures. We hypothesize the timing of collecting UPC samples in cats would be influenced by environmental stress. The aim of this study was to evaluate the effect of environmental stress on UPC in healthy cats.

Twelve cats were enrolled this prospective, nonmasked study. Owners collected a urine sample from their cat at home (AH) by free catch and the second sample was collected by veterinarian using cystocentesis in hospital (IH). Urinalysis, including dipstick, sediment, urine creatinine‐cortisol ratio (UCCR) and UPC were performed within 12 h after collection. Owners graded their cats' stress level form 0 to 10 (0 being non stressed and 10 being severely stressed) during AH urine collection, during transport to their appointments, and at the time of survey administration in the hospital examination room.

The cats' stress level was significantly different in each environment (*P* <  0.05). Stress score was highest IH (median, 10; range, 7–10), followed during transport (median, 6; range, 0–9) and then AH (median, 0; range, 0‐–2). The UCCR results of IH samples (median, 2.60 × 10^6^; range, 1.32–3.19 × 10^6^) were significantly higher than AH samples (median, 2.14 × 10^6^; range, 1.08–3.63 × 10^6^) (*P* < 0.01). Notably, ten of 13 paired samples (76.9%) demonstrated higher UPC ratio in hospital samples. The median of UPC of AH and IH samples was 0.09 (range, 0.065–0.186) and 0.11 (range, 0.070–0.236), respectively. The UPC was not significantly different between AH and IH urine sample (*P* = 0.064).

This study suggests that environmental stress of urine collection in cats is associated with UCCR differences. However, the UPC ratio was not significantly influenced by environmental stress.

**Disclosures**

No disclosures to report

## ESVNU‐P‐4

### ESVNU—European Society of Veterinary Nephrology and Urology

#### The prevalence of disease in diagnosis‐naïve older cats presented for health screening to two UK‐based veterinary clinics

##### J. P. Bestwick^1^, J. Elliott^2^, I. Orriss^2^, R. F. Geddes^1^

###### ^1^ Clinical Science and Services, Royal Veterinary College, Hertfordshire

^2^ Comparative Biomedical Sciences, Royal Veterinary College, London

Risk of some feline medical diseases increases with age, including chronic kidney disease (CKD) and hyperthyroidism, however, clinical signs can be dismissed by owners as ‘age‐related changes’. Quantifying the prevalence of disease in older cats would help veterinarians explain the value of health screening.

Cats (≥8 years old), without prior medical diagnoses, that underwent routine health screening at two UK‐based primary care veterinary clinics over a 52‐month period (02/10/2018 to 14/02/2023), were retrospectively analysed. At screening visits, history, physical examination, blood tests for biochemistry ± TT4, packed cell volume and total solids were performed. When possible, systolic blood pressure measurement (Doppler method; in 97.1% of cases) and urinalysis (cystocentesis; in 41.2% of cases; ± bacterial culture if indicated on sediment examination) were performed.

A total of 549 cats were screened with 199/549 (36.2%; 95% CI 32.2–40.3%) cats receiving ≥1 diagnosis. Median age of the cats was 13.5 (interquartile range: 11.3–15.8) years old. Diagnoses of hyperthyroidism, azotaemic CKD and hypertension were made in 89/549 (16.2%; 95% CI 13.1–19.3%), 62/549 (11.3%; 95%‐CI 8.6%–13.9%) and 37/533 (6.9%; 95%‐CI 4.8%–9.1%) cats, respectively. Bacteriuria (positive urine culture) was identified in 22/226 (9.7%; 95%‐CI 5.9%–13.6%) urine samples. Other diagnoses made included suspected neoplasia (8 cats), diabetes mellitus (8 cats), hepatopathy (3 cats) and suspected primary gastrointestinal disease (2 cats).

Routine medical health screening in diagnosis‐naïve senior cats is useful in detecting disease, many of which would benefit from early veterinary intervention. Longitudinal screening is needed to determine the incidence rate of these important medical conditions.

**Disclosures**

R. Geddes has received funding from Petplan, Royal Canin, an RVC Internal Grant, The Academy of Medical Sciences and The Everycat Foundation; has previously had a consultancy agreement with Boehringer Ingelheim; has received speaking honoraria from Boehringer Ingelheim, Idexx and Royal Canin.

J. Elliott has Consultancy agreements with: Elanco Ltd, CEVA Animal Health Ltd, Boehringer Ingelheim Ltd, MSD Animal Health Ltd., Orion Incorp, Idexx Ltd, Waltham Petcare Science Institute, Invetx Inc and Zoetis Ltd. received grant funding from Elanco Ltd, Waltham Centre for Pet Nutrition, Royal Canin SAS, Idexx Ltd., CEVA Animal Health. He is a member of the International Renal Interest Society which receives sponsorship from Zoetis.

I. Orriss receives research funding from Haomamedica Ltd.

J.P. Bestwick is currently undertaking a PhD studentship funded by Royal Canin SAS and previously completed an MPhil studentship funded by BSAVA PetSavers.**Source of Funding**

Royal Canin SAS

## ESVNU‐P‐5

### ESVNU—European Society of Veterinary Nephrology and Urology

#### Ultrasound assessments of cats associated with polycystic kidney disease caused by feline PKD1 genetic mutation

##### Dr. Jaturanratsamee^2^, Dr. Petchdee^1^

###### ^1^Faculty of veterinary medicine, Kasetsart University Kamphaeng, Saen, Thailand; ^2^Faculty of veterinary Medicine, Kasetsart University Kamphaeng, Saen, Thailand

Genetic variations associated with polycystic kidney disease (PKD) help explain some of this condition's complex phenotypes. A promising method for identifying clinical signs is ultrasonography. This study aimed to compare the kidney characteristics of cats with wild‐type cats and cats with PKD1 gene mutations. Ultrasonography was used to determine the characteristics of the kidney. A total of 30 cats of various breeds were included, with an average age of 39.74±8.68months. The findings revealed that 23.33% of cats had PKD1 mutations, with Persian and Persian‐related breed cats having the highest prevalence. Our findings revealed kidney characteristics in wild‐type cats and cats with gene mutations. A correlation between cyst formation in cats and gene‐mutated cats was discovered using ultrasonography. The discovery of a renal cyst on ultrasonography at less than 12 months of age could support the gene mutations. Ultrasonographic examination, in conjunction with genetic studies, may help to recognize the phenotypic variability of PKD1. By decreasing PKD mortality and morbidity in cats, the PKD1 phenotypic profile will direct therapeutic outcomes.

**Disclosures**

No disclosures to report

**Source of Funding**

Faculty of Veterinary Medicine, Kasetsart University

## ESVNU‐P‐6

### ESVNU—European Society of Veterinary Nephrology and Urology

#### Association of Fibroblast growth factor‐23 (FGF‐23) with parameters of renal dysfunction in dogs and cats

##### I Schäfer^1^, N Nagler^1^, SF Müller^1^, A Holtdirk^2^, T Tanja^2^, E Müller^1^, J Luckner^1^

###### ^1^Laboklin GmbH & Co. KG, Bad Kissingen, Germany; ^2^Dr. med. Kottmann—Clinical Research Organization, Hamm, Germany

Fibroblast growth factor 23 (FGF‐23) is a phosphaturic hormone used for monitoring of chronic kidney disease (CKD) in humans. Its value as a biomarker in canine and feline CKD still needs to be determined. The aim of this study was to evaluate the correlation of FGF‐23 with parameters of renal dysfunction in dogs and cats.

Left‐over serum samples from dogs and cats were collected for four groups (I: creatinine within reference interval, II‐IV: creatinine concentration according to IRIS II‐IV, *N* = 10 each). FGF‐23 was determined in serum using a quantitative ELISA kit (FGF‐23 ELISA Kit, Kainos Laboratories, Tokyo, Japan). Kruskal Wallis test was used to calculate statistical significance and Spearman's correlation testing for correlation analysis.

Results were available for 40 dogs and cats, each. In both species FGF‐23 concentrations showed statistically significant differences between study group I and IV (*P* < 0.001, both species), I and III (dogs: *P* < 0.001, cats: *P* = 0.001), and II and IV (dogs: *P* = 0.005, cats: *P* = 0.01). In dogs FGF‐23 concentrations correlate with IRIS‐staging (*ρ* = 0.879), urea (*ρ* = 0.770), creatinine (*ρ* = 0.762), and phosphorus (*ρ* = 0.742) (*P* < 0.001, each). In cats FGF‐23 concentrations correlate with urea (*ρ* = 0.835), IRIS‐staging (*ρ* = 0.806), creatinine (*ρ* = 0.764), and phosphorus (*ρ* = 0.532) (*P* < 0.001 each).

FGF‐23 strongly correlated with IRIS‐staging and urea in both, dogs and cats. In both species, although the correlation was strong, serum phosphorus concentration did not correlate as strong with FGF‐23 as would be expected for a phosphaturic hormone.

**Disclosures**

No disclosures to report

## ESVNU‐P‐7

### ESVNU—European Society of Veterinary Nephrology and Urology

#### Alterations in canine magnesium levels caused by Leishmania infantum‐induced chronic renal disease

##### MA Daza González^1^, L Solorzano^2^, B Pérez‐Montero^3^, G Miró^4^, ML Fermin^4^

###### ^1^Veterinary Teaching Hospital. Universidad Complutense de Madrid, Madrid, Spain; ^2^La Latina Veterinary Clinic, Madrid, Spain; ^3^Veterinary Teaching Hospital, Universidad Complutense, Madrid, Spain; ^4^Faculty of Veterinary Medicine, Universidad Complutense de Madrid, Madrid, Spain

Magnesium (Mg) is an essential electrolyte in the body due to its important role in multiple cellular functions. Its renal excretion places Mg as a possible biomarker of the glomerular filtration rate and the severity of chronic kidney disease (CKD). In recent years, the relationship between Mg and CKD has been studied in human and cats; dog studies are very limited. The purpose of the present study was to explore the association between serum total magnesium (tMg) and CKD caused by canine leishmaniosis (LCan), one of the main causes of glomerular pathology in dogs in the studied region.

A population of 53 dogs with CKD due to LCan was selected. Their serum tMg was measured in residual heparinized plasma, stored at −40°C, by the laboratory that also had performed the routine biochemical analysis. The dogs included in the study were classified into four groups according to the severity of CKD, as defined in the International Society of Renal Interest (IRIS); IRIS 1 (*n* = 21), IRIS 2 (*n* = 10), IRIS 3 (11), IRIS 4 (*n* = 11). A group of 21 healthy dogs was included, in which the concentration of tMg in plasma was determined.

ANOVA test showed significant differences in the concentration of tMg between the study groups (*P* < 0.001). Duncan test revealed significant differences between the IRIS 4 (2.86 ± 0.76 mg/dL) and IRIS 3 (2.09 ± 0.39 mg/dL) groups, and the previous ones with the IRIS 2 (1.72 ± 0.36 mg/dL), IRIS 1 (1.62 ± 0.2 mg/dL) and control group (1.68 ± 0.18 mg/dL). Spearman's correlation test showed a positive, strong, and significant correlation of tMg with plasma creatinine concentrations (*P* < 0.0001, *r* = 0.68155). When the data were fit to a simple straight line regression, the average slope was 0.1988 mg/dl tMg per 1 mg/dl creatinine (Mg = 0.1988 × crea + 1.4335), *R* ^2^ = 0.6424.

The results of this study showed alterations in tMg levels at different IRIS groups, and a correlation between tMg and creatinine. IRIS 3 and IRIS 4 dogs had the highest prevalence of hypermagnesemia (63.64% and 81.82% respectively), and showed significantly upper tMg levels than IRIS 1 and IRIS 2 dogs. The development of hypomagnesemia in some of these patients (IRIS I, 19.05%; IRIS II, 30%) deserves special attention, due to the importance of Mg in the maintenance of vascular tone.

This study places Mg as a potential biomarker of CKD severity. Further studies are required to assess the effect of magnesemia alterations on the progression of canine CKD.

**Disclosures**

No disclosures to report

## ESVNU‐P‐8

### ESVNU—European Society of Veterinary Nephrology and Urology

#### Effect of subcutaneous bypass (SUB) on proteinuria in cats with ureteral obstruction

##### O Vigouroux^1^, G Basse^2^, G McLauchlan^1^, J O'Keeffe^1^, M Gerou‐Ferriani^1^

###### ^1^AURA Veterinary, London, UK; ^2^1A Aldridge Road Villas, London, UK

Placement of a subcutaneous ureteral bypass (SUB) device for cats with a ureteral obstruction (UO) is the recommended treatment (ACVIM Consensus). The presence of mild proteinuria, suspected secondary to the implant, has not been described to the author's knowledge. This is of particular interest in the long term management of these cases which often have concurrent chronic kidney disease (CKD).

The aim of this study was to document the effect of SUB placement on proteinuria in cats.

Medical records of cats that had SUB placed due to a UO between 2018 and 2023 were reviewed. Cases were included if they had a urinalysis with urine protein to creatinine ratio (UPCR) prior to the SUB placement and at follow up at 1 month (FU1), 3 months (FU3), and/or 6 months (FU6) post‐placement. Urinalysis in all cases was performed for clinical reasons. Nine cats met the inclusion criteria.

The mean UPCR at presentation was 0.527 (Range: 0.07–1.23, reference interval: 0–0.2). UPCR was documented in 8 patients at FU1 and FU3 and 6 patients at FU6. Means UPCR at FU1, FU3 and FU6 were 1.13, 1.17 and 1.74 respectively. Urine culture was negative at all follow up examinations. The difference between the mean UPCR at FU1, FU3 and FU6 compared to presentation was 0.54, 0.67 and 1.14 respectively. Mean UPCR at FU1 and FU3 were significantly higher than at presentation (*P* = 0.048 and *P* = 0.034 respectively). At FU1 7/8 patients had an increase in UPCR compared to presentation.

Despite the relatively small sample size, this study suggests that a SUB placement is commonly associated with an increase in UPCR in cats. This increase appears to be permanent (3 and 6 months follow up), and not related to the immediate post‐placement period. The UPCR can increase to levels that typically require treatment in patients with CKD and without a SUB.

**Disclosures**

No disclosures to report

## ESVNU‐P‐9

### ESVNU—European Society of Veterinary Nephrology and Urology

#### Development of Cardiorenal Syndrome type IV (Chronic renocardiac syndrome) in cats attended to a nephrology service

##### MA Daza^1^, B López^2^, A Caro^3^

###### ^1^Veterinary Teaching Hospital. Universidad Complutense de Madrid, Madrid, Spain; ^2^Veterinary Teaching Hospital. Universidad de Córdoba, Córdoba, Spain; ^3^Faculty of Veterinary Medicine. Universidad Complutense de Madrid, Madrid, Spain

The cardiorenal syndrome type 4 (CvRD 4) occurs when a chronic kidney disease (CKD) contributes to a cardiac dysfunction. This study's primary goal is to evaluate the physiopathological phenotype of CvRD 4 in 105 cats with CKD who visited a nephrology facility.

Depending on whether a cat developed CvRD 4 or not, the general population was divided into two groups [CvRD 4− (*n* = 47), CvRD 4+ (*n* = 58)]. Having at least one of the following clinical findings during examination after receiving a CKD diagnosis was required for inclusion in the CvRD 4+ group: heart murmur or gallop rhythm, systemic arterial hypertension or hypertrophic cardiomyopathy (HCM). Cats exibited these characteristics before being diagnosed with CKD belonged to the CvRD 4‐ group. Hyperthyroid cats and those CvRD4 previosly diagnosed were escluded.

Between groups no differences were found regarding mean age (9.2 ± 4.9 vs. 10.4 ± 4.4 years old), sex, breed, hematocrit, eletrolytes, UPC or IRIS stage. Group CvRD 4+ had higher glucose levels than CvRD 4− (147 mg/dl vs. 122 mg/dl, *P* = 0.021).

CvRD 4+ showed a heart rate significantly higher than CvRD 4− (183 ± 31 bpm vs. 166 ± 30 bpm, *P* = 0.039). No differences were found regarding gallop rhythm, although there were variations in the auscultation of a murmur (CvRD 4+ 78.9% vs. CvRD 4‐ 21.1%; *P* = 0.001).

In the CvRD 4+ group, hypertension was more common (CvRD 4‐: 4.3 % *vs*. CvRD 4+: 53.4%). 38.7 % of the CvRD 4+hypertensive patients had a murmur.

The development of CvRD 4 was not related with the presence of echocardiographic HCM findings (CvRD 4‐: 37%, CvRD 4+: 63%, *P* > 0.05). Early hypertensives (40% CvRD 4+) may not yet exhibit echocardiographic abnormalities due, among others, to the heart's adaptive and protracted response to hypertension. Regarding the type of MHC that was presented—regional (CvRD 4−: 35.3%, CvRD 4+: 64.7%), or global (CvRD 4−: 40%, CvRD 4+: 60%—no differences were detected.

The difference in survival (CvRD 4−: 469± 120 days versus CvRD 4+: 623 ±99 days; *P* = 0.038) was statistically significant. The days of survival were determined using either the date of death or the patient's last visit.

Almost half of the general population had CvRD 4 development. In comparison to the group without CvRD 4, the group with CvRD 4 had a higher percentage of cardiac abnormalities, hypertension, and higher blood glucose levels. There was no decreased life expectancy with the CvRD 4.

**Disclosures**

No disclosures to report

## ESVONC‐P‐1

### ESVONC—European Society of Veterinary Oncology

#### Implementation of an Elekta Infinity linear accelerator with advanced treatment technology

##### M Kleiter ^1^, A Krischak ^1^, W Lechner ^2^, D Georg ^2^, B Wolfesberger ^1^

###### ^1^ University of Veterinary Medicine Vienna, Vienna, Austria; ^2^ Medical University of Vienna, Vienna, Austria

Radiotherapy in Veterinary Medicine has developed into a recognized subspecialty in Europe with an increasing demand for advanced treatment techniques. These advanced techniques allow high treatment accuracy. The aim of this study was to evaluate a first small patient cohort after implementation of a new state‐of‐the‐art linear accelerator.

An Elekta Infinity linear accelerator and a RayStation treatment planning system, which support image‐guided (IGRT), intensity and volumetric arc modulated (IMRT, VMAT) as well as stereotactic radiotherapy (SRT), were installed in 2022. Patients receiving radiotherapy between December 2022 and Mid‐March 2023 were assessed for utilization of the newly available treatment techniques.

Seventeen dogs, six cats and two rabbits were irradiated for various spontaneous occurring neoplasms in the study period. The majority of patients suffered from tumors in the head‐and‐neck region (64%) and presented with macroscopic disease (88%). IGRT was used in 22/25 patients. First VMAT plans were delivered after six‐weeks of familiarization to the new machine and thereafter applied in all but two patients (*n* = 10). SRT was not prescribed in the study period yet. Daily treatment protocols were administered in 20 and hypofractionated protocols in 15 patients. Acute radiation side effects were mild in all patients.

IGRT and especially the transition to VMAT allowed highly precise and fast delivery of complex treatment plans with superior sparing of organs‐at‐risk. The next exciting challenge will be the utilization of SRT in the near future.

**Disclosures**

No disclosures to report

## ESVONC‐P‐2

### ESVONC—European Society of Veterinary Oncology

#### Platelet count changes in cats with lymphoma following repeated vincristine administration

##### M Marceglia, A Zoia, PM Petini

###### San Marco veterinary clinic, Veggiano, Italy

Vincristine is an antimicrotubule chemotherapy drug that is used to treat haemopoietic tumour such as feline lymphoma. In healthy dogs and in those with ITP, vincristine administration increases platelet count. This information is not available for cats. Conversely, myelosuppression is a reported adverse effect. Therefore, the aim of this study was to determine platelet count variation following vincristine administration in cats with lymphoma.

Retrospective cohort study including cats with a cytological/histological diagnosis of lymphoma presented between 2010 and 2023. Each vincristine administration was recorded as a sequential event. Platelet count was performed before (−2 to 0 days) and after (7 ± 2 days) each vincristine administration with an ADVIA haematology analyser. All blood smears were evaluated by a clinical pathologist. Samples with platelet clump were excluded from analysis. Normality of continuous variable were evaluated by Shapiro‐Wilk test; changes in platelet count before and after each treatment were assessed with paired t‐test for normally distributed data or Wilcoxon Signed‐Ranks test for non‐normally distributed data or for sample size ≤20 cats; frequency of thrombocytopenic cats (platelet count reference interval = 130–430 × 103 μL) before and after each treatment was also reported. Significance was set at *α* = 0.05. Twenty‐seven cats were included at the first vincristine treatment. Two cats before and two cats after vincristine treatment were thrombocytopenic. There was no significant difference between pre‐ (mean, 333.3 ± 166.3 × 103 μL) and post‐ (mean, 393.4 ± 206.6 × 103 μL) platelet count (mean difference = 60.0 × 103μL; *t* = 1.78, *P* = 0.085). Twenty‐five cats were included at the second vincristine treatment. One cat before and one cat after vincristine treatment were thrombocytopenic. There was no significant difference between pre‐ (mean, 302.1 ± 112.8 × 103 μL) and post‐ (mean, 356.2 ± 132.5 × 103 μL) platelet count (mean difference = 54.2 × 103 μL; *t* = 1.99, *P* = 0.058). Sixteen cats were included at the third vincristine treatment. One cat before and one cat after vincristine treatment were thrombocytopenic. There was no significant difference between pre‐ (median, 273.5; IQR, 212.5–368 × 103 μL) and post‐ (median, 310.5; IQR, 229.5–408.5 × 103 μL) platelet count (median difference =54.5 × 103 μL; *W* = 37.5, *P* = 0.201). Finally, nine cats were included at the forth vincristine treatment. One cat before and one cat after vincristine treatment were thrombocytopenic. There was no significant difference between pre‐ (median, 337; IQR, 222.5–360.5 × 103 μL) and post‐ (median, 327; IQR, 225.5–482 × 103 μL) platelet count (median difference =10 × 103 μL; *W* = 14.5, *P* = 0.359).

Repeated vincristine administration does not cause thrombocytopenia in cats with lymphoma and it is not contra‐indicated in thrombocytopenic cats.

**Disclosures**

No disclosures to report

## ESVONC‐P‐3

### ESVONC—European Society of Veterinary Oncology

#### Outcomes and prognostic factors in canine T cell lymphoma treated with lomustine, vincristine, cyclophosphamide, and prednisone chemotherapy

##### A Einhorn, E Yas, G Dank, E Hanael, M Kent

###### Kiryat Ono, Israel

The most common first line treatment for canine lymphoma is a chemotherapy protocol that includes cyclophosphamide, doxorubicin, vincristine, and prednisone (CHOP). Canine T cell lymphoma has been found to have a significantly poorer prognosis than B‐cell lymphoma. There have been several studies investigating alternative protocols for T cell lymphoma. This retrospective study investigated the use of a cyclophosphamide, lomustine, vincristine, and prednisone protocol for naïve T cell lymphoma patients. Medical records of dogs treated for T cell lymphoma at a veterinary teaching hospital from 2017 to 2022 were reviewed. Response rate, toxicity, progression‐free survival and survival time were compared to historically reported results with CHOP chemotherapy. Factors potentially related to prognosis were statistically analyzed. Twenty‐eight dogs were included in the study. Of the 26 dogs that had a response to the study's chemotherapy protocol the median progression free survival (PFS) time was 166 days (95% CI 119–213 days). Median survival time (MST) for the whole study group was 318 days (95% CI 248–374 days). Twenty‐four dogs experienced gastrointestinal adverse events during the protocol, with 79% of these being grade 1 or 2 as per VCOG‐CTCAE. This protocol has shown similar median PFS time but a prolonged MST compared with previous studies for canine high grade T cell lymphoma treated with CHOP, alongside minimal toxicity, and suggests the inclusion of lomustine in first line chemotherapy protocol against canine T cell lymphoma.

**Disclosures**

No disclosures to report

## ESVONC‐P‐5

### ESVONC—European Society of Veterinary Oncology

#### Retrospective evaluation of lung lobectomy and adjuvant treatment for primary pulmonary carcinoma in dogs: a multi‐institutional study

##### E Treggiari ^1^, G Romanelli ^1^, P Valenti ^2^, V Montinaro ^2^, M Rossanese ^3^

###### ^1^ Centro Specialistico Veterinario, Milan, Italy; ^2^ Clinica Veterinaria Malpensa AniCura, Samarate, Italy; ^3^ Royal Veterinary College, Queen's Mother Hospital for Animals, Hatfield, UK

Primary lung cancer is relatively common in dogs. The literature on long‐term follow‐up and outcome is sparse and further information is needed. The purpose of this study was to describe signalment, histopathological diagnosis and outcomes of dogs undergoing lung lobectomy with or without adjuvant chemotherapy for primary lung carcinomas and to identify factors associated with survival. The medical records of four European institutions were reviewed to identify dogs undergoing lung lobectomy for a primary lung carcinoma between 2005 and 2022.

Dogs were included if they had undergone advanced imaging procedures in the absence of disseminated disease (pulmonary metastatic disease), lung lobectomy with a histopathological diagnosis of epithelial cancer and if they had a follow‐up of at least 6 months or less if marked as deceased. Dogs were excluded if medical records were incomplete. Data collected included signalment, body weight, examination findings, preoperative diagnostic findings, surgical treatment and time, occurrence of perioperative complications, time to discharge, tumour dimensions, histopathological features, recurrence and use of chemotherapy. Outcome measures evaluated were survival time and time to progression (TTP).

A total of 89 dogs were included in the study. Median age was 11 years (range 5–17 years) and median body weight was 23 kg (range 2.5–47 kg). Overall median survival time (MST) was 252 days (range 6–1558 days) and overall TTP was 140 days (range 7–684 days). The 1‐, 2‐ and 3‐year survival rate was 61%, 47% and 30%, respectively.

Presence of clinical signs at presentation, presence of pleural effusion, completeness of surgical margins, histopathological features (histological grade, tumour type) and use of chemotherapy did not influence survival. Factors associated with an increased risk of death included tumour size and lymph node metastasis: patients with maximum tumour diameter ≥5 cm had a reduced survival compared to patients with smaller tumours (MST 284 days vs. 717 days, 95% CI 8–719, *P* = 0.005), and dogs with histologically confirmed lymph node metastasis had a reduced survival compared to patients with no evidence of lymph node metastasis (MST 162 days vs. 614 days, 95% CI 39–760, *P* < 0.001). None of the assessed variables influenced TTP.

In conclusion, dogs with primary pulmonary carcinoma may have a better prognosis and prolonged survival if no lymph node metastasis are identified and in case of maximum tumour diameter <5 cm. Further studies are needed to elucidate the role of adjuvant chemotherapy in case of clinically aggressive lung cancer.

**Disclosures**

Dr. Valenti and Dr. Treggiari are consultants for WizzVet, Dr. Montinaro is a consultant for Vallonea Lab and Dr. Rossanese is co‐founder of VetSpoke.

## ESVONC‐P‐6

### ESVONC—European Society of Veterinary Oncology

#### Thermographic assessment of soft tissue sarcomas in dogs

##### E Hanael ^1^, T Buber, A Rice, T Shasha, Y Osokin, G Dank ^2^

###### ^1^ HT Vet Hod, HaSharon, Israel; ^2^ Hod, HaSharon, Israel

Medical infrared thermal imaging is a non‐invasive imaging modality that measures the surface body heat. This technology has been evaluated both in human and veterinary oncology, however the reports in veterinary medicine were controversial regarding the use as a screening tool.

The aim of this study was to compare the steady state temperature of soft tissue sarcomas to the adjacent healthy tissue using infrared thermography.

Forty dogs with soft tissue sarcomas were included in this study. Optic and thermographic images of neoplastic lesions and bordering normal skin were prospectively acquired. Marking of the mass and healthy region s were conducted to calculate mean temperatures. Following thermographic assessment, in order to diagnosed tumor type, biopsy or tumor resection was conducted and submitted to histopathologic. The study included 25 grade 1, 8 grade 2 and 4 grade 3 sarcomas. Three cases did not have grading performed. For each case mean steady state temperature of mass and adjacent healthy skin regions was calculated and recorded. Using paired *t*‐test analysis, the difference of steady state temperature of the two regions was examined.

There was a significantly higher temperature in the soft tissue sarcomas when compared to the adjacent healthy tissue (*P* < 0.01).

This study suggests that use of infrared thermography and comparing the difference of steady state temperature of soft tissue sarcomas to adjacent healthy tissue can be used as a screening tool and aid in detection of soft tissue in dogs.

**Disclosures**

All authors are employed by HT Vet

**Source of Funding**

HT Vet

## ESVONC‐P‐7

### ESVONC—European Society of Veterinary Oncology

#### Feasibility of using flow cytometric analysis of Ki‐67 expression in canine cutaneous mast cell tumours—a pilot study

##### P Odatzoglou, RHewitt, H Wong, K Hughes, C Hare, JM Dobson, B Blacklaws

###### Department of Veterinary Medicine, University of Cambridge, Cambridge, UK

Mast cell tumours (MCTs) are the most commonly encountered canine cutaneous tumour. Histologic grade is considered the most consistent and reliable prognostic factor available for canine cutaneous MCTs. Several markers of proliferation have been evaluated to assist prognosis including Ki‐67, a protein found in the nucleus, and its levels have been correlated with proliferation and prognosis. Ki‐67 expression is currently measured using immunohistochemistry (IHC). Ki‐67 expression in MCTs has not been evaluated by flow cytometry (FC). This pilot study aimed to assess the feasibility of evaluating Ki‐67 expression in canine MCTs using FC to guide treatment and prognostication.

All dogs underwent staging as per standard procedure. Those deemed surgical candidates had their MCT excised. FC samples were collected immediately following excision on the fresh tissue sample and submitted for FC analysis following a cellularity count. Samples were stained for dead cells, CD45, CD117 and Ki‐67 expression. FC analysis was performed immediately, acquiring a minimum of 100,000 events/ sample. Where a sufficient volume of sample remained this was additionally acquired on an imaging FC. The remainder of the excised tissue was fixed in 10% neutral‐buffered formalin and Ki‐67 IHC was performed. Ki‐67 counting on IHC was performed manually by a board‐certified veterinary pathologist and automatically using QuPath, an image analysis software.

Seven dogs with cutaneous (cMCTs, *n* = 5) and subcutaneous (scMCTs, *n* = 2) were included in this study. Samples from five dogs (3 cMCTs and 2 scMCTs) had sufficient cellularity. FC optimisation was performed on the initial two scMCT samples. Imaging FC data were used to validate the Ki‐67 staining with Ki‐67 specifically being localized to the nuclear regions of CD117+ cells. Two cMCTs were grade 2/low‐grade (Patnaik/Kiupel) and one was grade 3/high‐grade.

Ki‐67+ cell percentage of all live/CD45+/CD117+ cells obtained with FC were 6.56% and 3.55%, for the low‐grade cMCTs and 11.22% for the high‐grade cMCT. This was compared to the Ki‐67+ IHC manual count (versus QuPath Ki‐67+ tumour‐cell count) of 0.60% (vs. 0.60%), 0.68% (vs. 0.59%) for the low‐grade cMCTs and 69.43% (vs. 24.96%) for the high‐grade cMCT.

This study shows that Ki‐67 expression of mast cells from cMCTs can be measured by FC. The Ki‐67 percentage determined by FC showed similar trends to those defined immunohistochemically. This suggests that, following threshold optimisation, FC could be used to estimate Ki‐67 expression of canine cMCTs by FNA in a less invasive and timelier manner than IHC of surgically excised tumours.

**Disclosures**

No disclosures to report

## ESVONC‐P‐8

### ESVONC—European Society of Veterinary Oncology

#### Round cell tumours and the feline tarsus: a fatal attraction

##### G Lollo^1^, S Sabattini^1^, R Zaccone^1^, A Rigillo^2^, C Ferrari^1^, L Marconato^1^

###### ^1^University of Bologna, Ozzano emilia (Bo,) Italy; ^2^I‐Vet srl—Veterinary diagnostic lab, Flero (BS), Italy

Although tarsal neoplasms are rare, a predilection of round cell tumours (RCTs) and soft tissue sarcomas (STSs) for the feline hock region has been described. Among RCTs, lymphoma, plasmocytoma and histiocytic sarcoma have been more frequently reported. The differential diagnosis among RCTs is challenging, because of similar clinical presentation and common histologic features. Treatment is likewise challenging, and a standard‐of‐care does not exist.

The aim of this retrospective study was to confirm the high prevalence of RCTs in the tarsus, characterize tumor histotypes, and compare clinical features between RCTs and STSs. For each case, information regarding signalment, clinical history and presentation, original diagnosis, treatment and follow‐up was retrieved. All RCTs were histologically reviewed and immunohistochemistry with CD3, CD20, MUM‐1, CD18, IBA‐1 and E‐CAD was carried out. In six cases with additional formalin‐fixed and paraffin‐embedded material available, transmission electron microscopy (TEM) was performed.

Thirty‐two cats with tarsal neoplasia diagnosed between January 2000 and December 2022 were identified, including 14 RCTs, 14 STSs, 2 hemangiosarcoma, 1 osteosarcoma and 1 fibrolipoma. Former RCT diagnoses included lymphoma (*n* = 3), plasmocytoma (*n* = 2), histiocytic sarcoma (*n* = 2), feline progressive histiocytosis (FPH; *n* = 1) and undifferentiated RCT (*n* = 6). Following immunohistochemistry, diagnosis changed in 79% cases, identifying 5 plasmacytomas, 4 histiocytic sarcomas, 2 B‐cell‐lymphomas, 2 FPH and 1 undifferentiated RCT. TEM was not reliable for RCT differentiation.

Male cats were significantly overrepresented in the RCT group compared with STS (93%; *P* = 0.004). Half of the cats with RCT experienced previous trauma in the hock and 37.5% received hindlimb vaccination.

Among cats with RCTs, treatment included surgery alone (*n* = 5), CHOP (*n* = 3), surgical excision and adjuvant melphalan (*n* = 1), and prednisolone (*n* = 1). Two cats did not receive any treatment and for the remaining two the information was not available.

Among cats with STSs, treatment information was available for 9/14 cases and consisted of surgical excision only.

The median survival of cats with RTC was significantly lower compared with STS (212 days vs. not‐reached; *P* = 0.027), with 1‐year survival rates of 18.2% and 83.3%, respectively. In contrast, time to progression was not significantly different (*P* = 0.259) between the two groups, due to the high frequency of local recurrences in STSs treated with marginal excision.

This study confirms the high prevalence of RCTs in the feline tarsus. Immunohistochemistry was crucial for the diagnostic framework. Tarsal RCTs were associated with a poor prognosis. Male cats were predisposed, and local trauma may be involved in RCT pathogenesis.

**Disclosures**

No disclosures to report

## ESVONC‐P‐9

### ESVONC—European Society of Veterinary Oncology

#### Surgery and adjuvant electrochemotherapy compared to surgery alone for treatment of canine soft tissue sarcomas

##### SC Ramos^1^, MJ Macfarlane^1^, AF Santos^2^, T Sparks^3^, GA Polton^1^

###### ^1^North Downs Specialist Referrals, Bletchingley, UK; ^2^Institute of Biomedical Sciences Abel Salazar, Porto, Portugal; ^3^Waltham Petcare Science Institute, Leicestershire, UK

The predominant challenge in the management of canine soft tissue sarcomas (STS) is local tumour control, for which surgical excision with wide margins is the standard treatment. Depending on the histological grade and margin assessment, adjuvant treatment may be recommended. Electrochemotherapy (ECT) combines chemotherapeutic drugs with electric pulses, facilitating cellular uptake of cytotoxic agents, thus increasing their cytotoxicity. ECT in canine STS has been reported. Preliminary studies showed low treatment toxicity and suggested effectiveness at reducing incidence of recurrence following incomplete excision.

The first aim of this retrospective study was to investigate the recurrence rate (RR) and time to recurrence (TTR) in dogs with STS solely treated with surgical excision and those also treated with adjuvant ECT. The second aim was to evaluate adverse effects associated with ECT.

Medical records of one oncology center were retrospectively reviewed to identify dogs with completely staged, non‐metastatic, cutaneous or subcutaneous incompletely excised intermediate‐grade STS, that underwent surgical excision alone or surgical excision followed by ECT. The ECT protocol was uniform and consisted of two treatments, two weeks apart, with intravenous bleomycin followed by sequential application of trains of biphasic pulses. TTR was defined as the time interval between surgery and detection of local recurrence.

Twenty‐four dogs were included in the analysis; 14 underwent surgery (group I) and 10 had surgery followed by ECT (group II). Both groups were similar in terms of tumour location, size and mobility.

Follow‐up duration for the two groups was comparable. Median follow‐up (range) was 491 days (282–750) for group I and 716 days (176–805) for group II. Kaplan Meier median (mean) TTR estimates were 420 days (411 days) for group I and median not reached (632 days) for group II. Recurrence rate was 57% for group I and 30% for group II. The recurrence rate for group II still appeared lower after differences in tumour size were considered. For cases with known dimensions (*n* = 22), recurrence rate was higher in larger (≥ 5 cm diameter) than smaller tumours (70% vs. 30%, respectively). Seven patients (70%) that received ECT experienced only mild local toxicity (erythema) that did not require further intervention.

Our results suggest that adjuvant ECT for the treatment of canine incompletely excised intermediate‐grade STS may be associated with a lower recurrence rate. ECT was well tolerated with minimal local toxicity. Our study also showed that tumour size may be a prognostic factor, consistent with published literature.

**Disclosures**

No disclosures to report

## ESVONC‐P‐10

### ESVONC—European Society of Veterinary Oncology

#### Evaluation of staging in 504 dogs with aggressive peripheral nodal B cell lymphoma across 16 Oncology referral centres

##### CIK Hawkes^1^, L Blackwood^1^, J Morris^2^, S Bavcar^3^, C Auquier^4^, S Benjamin^5^, J Borrego^6^, S Bottin^7^, O Davies^8^, I Desmas‐Bazelle^9^, A Einhorn^10^, CM Figueroa‐Gonzalez^9^, K Holenova^11^, E Kritsotalaki^12^, K Peak^13^, K Smallwood^14^, E Treggiari^15^, P Valenti^16^, M Virgen^17^, S Ray^2^, Q Fournier^7^

###### ^1^University of Edinburgh, Edinburgh, UK; ^2^University of Glasgow, Glasgow, UK; ^3^Small Animal Specialist Hospital, Sydney, Australia; ^4^University of Liege, Liege, Belgium; ^5^Davies Veterinary Specialists, Hitchin, UK; ^6^Clinico Hospital Auna Especialidades Veterinarias, Valencia, Spain; ^7^Aura Veterinary, Guildford, UK; ^8^Highcroft Veterinary Referrals, Bristol, UK; ^9^Royal Veterinary College, London, UK; ^10^Koret School of Veterinary Medicine, Rehovot, Israel; ^11^Wear Referrals, Bradbury, UK; ^12^Southfields Veterinary Specialists, Basildon, UK; ^13^Anderson Moores, Winchester, UK; ^14^North Downs Specialist Referrals, Bletchingley, UK; ^15^Centro Specialistico Veterinario, Milan, Italy; ^16^Clinica Veterinaria Malpensa, Milan, Italy; ^17^Auna Especialidades Veterinarias, Valencia, Spain

There is no current consensus on staging of dogs with multicentric lymphoma, even in standard texts. The purpose of this study was to describe the staging of 504 dogs with aggressive peripheral nodal B cell lymphoma (APNBCL) across sixteen oncology referral centres between 2014 and 2021.

Five hundred and four dogs with histologically (79 cases) or cytologically (425) confirmed APNBCL were included. Immunophenotyping was most commonly achieved by flow cytometry (250 dogs, 49.6%), compared with immunocytochemistry (100 dogs, 19.8%) PCR for antigen receptor rearrangement (PARR) in 97 dogs (19.2%) and immunohistochemistry in 71 dogs (14.1%).

Abdominal imaging was performed in 298 dogs (59.1%); abdominal ultrasound was the most frequently used modality (270 dogs, 53.7%), computed tomography (CT) in 27 dogs (5.3%) and abdominal radiographs in 1 dog (0.3%). Liver fine needle aspirate cytology was carried out in 152 patients (30.2%) and splenic cytology in 165 patients (32.7%). Thoracic imaging was performed in 271 dogs (53.8%); radiographs in 235 (46.8%), and CT in 36 cases (6.9%). Although gold standard staging has historically involved bone marrow sampling, this was infrequently performed (54 dogs, 10.7%). All dogs had a complete blood count and serum biochemistry at diagnosis. Blood smear evaluation by a clinical pathologist occurred in 378 dogs (75%).

Most dogs with APNBCL were diagnosed and immunophenotyped by cytology and flow cytometry. Histopathology and immunohistochemistry were uncommonly used. Imaging was commonly performed, however more invasive tests such as bone marrow biopsy, liver and spleen cytology were relatively infrequently utilised. Test selection likely also reflects prudent use of finances, prioritising therapy rather than investigation.

**Disclosures**

No disclosures to report

## ESVONC‐P‐11

### ESVONC—European Society of Veterinary Oncology

#### A retrospective clinico‐pathologic study of 37 dogs with urethral carcinoma (2012–2022)

##### G Ghisoni, A Foglia, C Agnoli, S Sabattini, S Perfetti, F Dondi, LM Marconato

###### Ozzano dell'Emilia (BO) Italy

Transitional cell carcinoma (TCC) is the most common urinary tract cancer in dogs. Primary urethral TCC (uTCC) is rare and a female sex predisposition has been documented. Treatment is influenced by clinical stage and the presence of urethral obstruction (UO); surgery is usually not an option, and radiation therapy has not been well documented. Chemotherapy and cyclooxygenase inhibitors (COXi) are standard‐of‐care in dogs with uTCC. Overall, prognosis is often poor because of local, nodal and distant progression.

The aim of this retrospective study was to review clinicopathologic features, treatment modalities and potential contributing and prognostic factors, including presence of UO, urinary tract infection and azotemia at diagnosis, stage and therapeutic approach.

Medical records of dogs with histologically‐confirmed uTCC were reviewed: 37 dogs (30 females, 27/30 spayed, and 7 intact males) of a median age of 11 years (range, 6–15) and median body weight of 18 kg (range, 6–43) were identified. At admission, all dogs had stranguria, pollakiuria and hematuria; urethral thickening was the main clinical finding (50%) and UO was detected in 6 (16%) dogs. Twelve dogs (32%) had lymph node metastasis (*n* = 10; 84%), distant metastases (*n* = 1; 8%) or both (*n* = 1; 8%). Therapeutic strategies included chemotherapy and COXi (*n* = 28; 75%), chemotherapy and COXi combined with radiation therapy (*n* = 4; 11%), debulking laser and COXi (*n* = 2; 5%), debulking laser (*n* = 1; 3%), COXi alone (*n* = 1; 3%) and urethral stent positioning (*n* = 1; 3%). Drugs administered as first‐line protocol included gemcitabine (*n* = 16; 50%), vinblastine (*n* = 7; 22%), gemcitabine and carboplatin (*n* = 4; 13%), carboplatin (*n* = 2; 6%), chlorambucil (*n* = 2; 6%) and mitoxantrone (*n* = 1; 3%). Based on Response Evaluation Criteria in Solid Tumors, 1 (3%) dog obtained complete remission, 9 (28%) partial remission, 17 (53%) were stable and 5 (16%) progressed. Four of the 6 dogs with UO experienced obstruction resolution or improvement after the first chemotherapy dose. Dogs were followed‐up for a median time of 166 days (range, 1–1215). Median local time to progression was 171 days (range, 74–250), while median time to metastasis was not reached. Median survival time was 350 days (range, 170–530), with 1‐year survival rate of 34%. Dogs with UO at the time of diagnosis had higher risk of local progression (Hazard Ratio [HR]: 5.5) and tumor‐related death (HR: 3.7); UO was the only significant negative prognostic factor. According to our results, overall survival was longer than previously reported, and a multimodal therapeutic approach should be considered to improve outcome of dogs with uTCC.

**Disclosures**

No disclosures to report

## ESVONC‐P‐12

### ESVONC—European Society of Veterinary Oncology

#### A retrospective study of clinical outcome in response to short chemotherapy protocol for feline intermediate‐ and large cell lymphoma

##### M Larsen^1^, M Arendt^2^

###### ^1^Evidensia Specialist Animal Hospital in Helsingborg, Helsingborg, Sweden; ^2^University of Copenhagen, Frederiksberg C, Denmark

The objective of this study was to evaluate the long term response to treatment with an ultra‐short maintenance‐free treatment protocol of cats with lymphoma.

In a retrospective study, 25 cats with cytologically or histologically confirmed intermediate‐ or large cell lymphoma were included. All cats were treated with a maintenance free induction protocol of cyclophosphamide, vincristine and prednisolone (COP) for 8 weeks, or shorter if the response was inadequate. Patients with no or unsatisfying response (*n* = 9), were discontinued and switched to either a short CHOP protocol, lomustine‐based treatment or radiation therapy. Response to treatment with COP/CHOP protocol was categorized into responders (complete and partial remission) and non‐responders (stable disease or progressive disease).

Of the 16 responders, 10 cats achieved complete remission with a median progression free interval (PFI) of 879 days and 6 cats achieved partial remission, median PFI 152.5 days. For all responders, median PFI was 347 days. For all non‐responders (*n* = 9) median PFI was 18 days, the group included 4 cats with renal involvement.

Response to therapy was significantly related to long term lymphoma free survival.

The outcome of this study suggests, that cats with lymphoma, being treated with a short chemotherapy protocol without maintenance and achieving complete remission, have a high probability of enduring long time progression free interval, comparable with progression free intervals in previous studies using longer treatment protocols.

**Disclosures**

No disclosures to report

## ESVONC‐P‐13

### ESVONC—European Society of Veterinary Oncology

#### Erythrocyte and platelet characteristics as indicators of metastasis in canine carcinomas and sarcomas

##### A Mulder, A Goddard, P Pazzi

###### University of Pretoria, Onderstepoort, South Africa

Erythrocyte and platelet characteristics have been studied in humans as prognostic indicators in carcinoma, sarcoma, and metastatic disease. In contrast, few studies have investigated erythrocyte and platelet characteristics as indicators of metastasis in dogs with carcinoma and sarcoma.

Erythrocyte and platelet variables, derived from the Advia 2120i, and subjective erythrocyte and platelet morphology were retrospectively evaluated on samples from forty‐nine dogs with carcinoma or sarcoma that underwent complete postmortem examination. The aim of the study was to identify differences in erythrocyte and platelet characteristics in tumour‐bearing dogs compared to healthy dogs, and to evaluate these characteristics as indicators of metastasis. Tumour‐bearing dogs were compared to twenty healthy age‐controlled dogs, additionally tumour‐bearing dogs with metastasis were compared to tumour‐bearing dogs without metastasis.

Compared to healthy dogs, tumour‐bearing dogs had significantly decreased haematocrit (*P* < 0.001) and reticulocyte haemoglobin content (*P* = 0.01), and increased anisocytosis (*P* < 0.001), polychromasia (*P* < 0.001), macrocytosis (*P* = 0.017), codocytes (*P* = 0.011), leptocytes (*P* = 0.031), absolute reticulocyte count (*P* = 0.02), platelet concentration (*P* < 0.001), platelet volume distribution width (*P* < 0.01) and plateletcrit (*P* < 0.01). The only significantly different characteristics in tumour‐bearing dogs with metastasis compared to tumour‐bearing dogs without metastasis, were a decreased mean cell volume (*P* < 0.005) and an increase in polychromasia (*P* = 0.034).

Significant differences in erythrocyte and platelet characteristics exist between dogs with carcinoma and sarcoma compared to healthy dogs, but only mean cell volume and polychromasia were significantly different in tumour‐bearing dogs with metastatic disease.

**Disclosures**

No disclosures to report

## ESVONC‐P‐14

### ESVONC—European Society of Veterinary Oncology

#### Is Chemotherapy Useful In Feline Mammary Tumours?

##### N Del Castillo ^1^, N Rayón ^2^, S Márquez ^3^, C De la Riva ^3^, R Ruano ^4^, C Aceña ^5^, E Rollón ^6^, B Tormo ^3^, S Moreta ^7^, V Domingo ^8^

###### ^1^ Alfonso X El Sabio, CV Surbatan, OncoMascotas, CVM Delicias Villanueva de la Cañada, Madrid Spain; ^2^ CV Surbatan, Madrid, Spain; ^3^ OncoMascotas, Madrid, Spain; ^4^ HV Mediterraneo, Madrid, Spain; ^5^ Zaragoza University, Zaragoza, Spain; ^6^ CV Canymar, Cádiz, Spain; ^7^ CVM Delicias, Madrid, Spain; ^8^ Atypia, Granada, Spain

Feline mammary tumours (FMT) represent 17% of neoplasms in female cats,^1^ and are very aggressive, with a high metastatic rate.^1,2^ Clinical stage and histological grade are prognostic factors.^3^

Surgery has been accepted as the main treatment in FMT,^4^ although, through its metastatic ability, most of the cats will die within a year. There are few publications about adjuvant chemotherapy treatment after surgery, and its effectiveness has not been proved.

Forty‐six female cats with TMF were included retrospectively in this study [stages I (13%/6), II (39.10%/18), III (47.80%/22); grade I (15.20%/7), II (45.6%/21), III (39.10%/18)]. Fourteen cats did not receive chemotherapy treatment (30.40%) and 32 cats (69.6%) were treated with different antineoplastic drugs (doxorubicin (68.75%/22), mitoxantrone (25%/8), carboplatin (3.125%/1), carboplatin plus toceranib (3.125%/1)). Thirty cats (65,20%) received NSAID and 16 (34,80%) did not, with or without adjuvant chemotherapy.

Our results showed a median survival time (MST) of 599.97 days (334 days for cats that did not received chemotherapy, 469.07 days for cats treated with doxorubicin, 657.19 days for mitoxantrone, 200 days for carboplatin and 300 days for carboplatin plus toceranib). These results were not statistically significant (*P* = 0.596) in univariate analysis (Kaplan Meier).

Literature describes a MST of 4–12 months after surgery for tumours greater than 3 cm and stage III.^3,5^ The use of different chemotherapeutics drugs has been described as an adjuvant treatment to surgery in FMT; doxorubicin has showed a MST of 448 days (*n* = 67),^6^ and mitoxantrone 480 days (*n* = 12).^7^ There are also publications comparing survival time when the only treatment is surgery or when surgery is combined with different chemotherapeutic drugs. In these publications, carboplatin has not showed a statistical benefit (*n* = 16),^8^ neither doxorubicin (*n* = 34), or metronomic chemotherapy combined with meloxicam (*n* = 23). MST was 421 days for cats treated with surgery and carboplatin,^8^ and 430 days for FMT treated with surgery and metronomic chemotherapy (MST was 338 days for cats treated only with surgery, *n* = 80).^9^

The use of chemotherapy as adjuvant treatment to mastectomy in TMF stays questionable nowadays, based on bibliographic references and our results data. Mitoxantrone seems to contribute to a better prognosis, but the few numbers of cats included could alter the results.

**Disclosures**

No disclosures to report

## ESVONC‐P‐15

### ESVONC—European Society of Veterinary Oncology

#### Toceranib (Palladia) as a first line therapy in macroscopic canine oral squamous cell carcinoma: a case series

##### M Garcia‐de la Virgen, S Vázquez‐Martínez, A Lara‐Garcia, J Borrego‐Massó

###### IVC Evidensia Aúna Especialidades Veterinarias, Paterna (Valencia), Spain

Toceranib has been documented to provide clinical benefit in dogs with a variety of carcinomas. Long‐term disease control can be achieved in canine oral squamous cell carcinomas (OSCCs) with a compartmental surgical approach. When local therapy is not feasible, cases managed with cytotoxic chemotherapy has a low response rate and short progression free survival. The aim of this retrospective study was to evaluate efficacy and toxicity of OSCCs to toceranib in combination with non‐steroidal anti‐inflammatories (NSAIDs) as first line therapy in the macroscopic setting.

Dogs with a cytological or histopathological diagnosis of naïve macroscopic OSCC (mandible, maxilla or lingual), complete work‐up consisting of physical examination (PE), three view thoracic radiographs (TR) or computed tomography (CT) and bloodwork; with any stage were included. Toceranib was administered PO (2.4–2.75 mg/kg) on a Monday, Wednesday and Friday (MWF) schedule alongside an NSAID at standard dose. Further analgesia was administered as needed. Response evaluation was performed at 8 weeks of therapy and follow‐up staging was performed every 3 months thereafter including PE, imaging and bloodwork. Data collected included signalment, response to therapy, staging, adverse events (AE) (according to VCOG‐CTCAEv2), dose reductions, progression free survival (PFS) and overall survival (OS). Overall response rate (ORR) (following cRECIST v1.0), clinical benefit (CB) median PFS and OS were evaluated.

Nineteen dogs were included, stage I (*n* = 1), stage II (*n* = 4), stage III (*n* = 11) and stage IV (regional lymph node *n* = 3, lung *n* = 1). Median weight and age were 10.5kg and 10 years respectively. Median toceranib dose administered was 2.5 mg/kg (2.2–3 mg/kg). Toxicity was mild, with 11 total AE episodes, 82% grade I/II and 8% grade III. All hematological AEs were neutropenic episodes, grade I (*n* = 3). Gastrointestinal AEs were grade I/II (*n* = 4) and grade III (*n* = 2). Metabolic AEs included creatinine elevation, grade I (*n* = 1) and grade II (*n* = 1). No grade IV/V AEs occurred. ORR and CB were 66.7% and 83% respectively (5 CR, 7 PR, 5 SD). PFS and OS were 111 and 216 days respectively. PFS for dogs that achieved objective response (CR and PR) was 347 days.

Although the small number of patients included and retrospective nature of this study, we observed a low toxicity and a CB in the majority of dogs with macroscopic OSCC treated with a toceranib and a NSAID combination.

**Disclosures**

No disclosures to report

## ESVONC‐P‐16

### ESVONC—European Society of Veterinary Oncology

#### Evaluation of the CLOP protocol as induction in canine non‐indolent T‐cell lymphoma

##### S Vázquez‐Martínez, M García‐de la Virgen, J Borrego‐Massó, A Lara‐García

###### IVC Evidensia Aúna Especialidades Veterinarias, Paterna (Valencia), Spain

Canine non‐indolent T‐cell lymphoma (cNITCL) is associated with low response rates and short progression free survivals (PFS) with standard CHOP protocols, as a result alternative alkylating‐based induction protocols are currently being evaluated. The CLOP protocol is a modification of the Madison‐Wisconsin protocol with similar schedule, drugs and dosages, except that doxorubicin is substituted with lomustine at 60 mg/m^2^. The aim of this retrospective study was to assess the efficacy and safety of CLOP induction in cNITCL and to compare it with a standard CHOP protocol.

Cases of confirmed multicentric cNITCL with cytology and immunocytochemistry or flow cytometry, naïve to therapy and treated with CLOP or Madison‐Wisconsin (19 or 25 weeks), were retrospectively selected. Data collected included signalment, physical examination, staging (complete bloodwork, thoracic and/or abdominal imaging), adverse events (AEs) (VCOG‐CTCAEv2), response (cRECISTv1.0) and median PFS. Kaplan‐Meier estimation and Log‐Rank test were used to determine and compare median PFS between groups.

Twenty‐five dogs of different breeds were included, with median age and weight of eight years and 19.5 kg, respectively. All dogs were classified as stage III‐IV (substage *a* = 9 and substage *b* = 16) and 11 dogs were hypercalcemic. Fourteen dogs were treated with CLOP and 11 with CHOP. Hematological AEs (neutropenia, thrombocytopenia) were more frequent, *n* = 49 CLOP group vs. *n* = 29 CHOP group. Gastrointestinal AEs were less frequent, *n* = 5 CLOP group vs. *n* = 10 CHOP group. Dose‐limiting AEs (grade III/IV) with CLOP were *n* = 17 (neutropenia =15, increased ALT =2) and *n* = 3 with CHOP (gastrointestinal =1, neutropenia =2); no dog discontinued treatment due to toxicity. Overall response rate (ORR) was 85.7 % (CR 71.4%, *n* = 10) with CLOP protocol and 81.8% (all CR *n* = 9) with CHOP protocol. Only seven dogs treated with CLOP and four dogs treated with CHOP completed the protocol. The median PFS was 149 days in the CLOP group and 125 days for CHOP group. Median PFS of dogs experiencing CR was CLOP = 232 days and CHOP = 153 days. CLOP CR 6‐months PFS rate was 70% and 1‐year PFS rate was 27%. CHOP CR 6‐months PFS rate was 44% and 1‐year PFS rate was 11%. No statistically significant differences were observed for PFS between groups.

CLOP was well tolerated despite higher prevalence of neutropenic episodes and provided a similar response rate and PFS than CHOP in this small population of dogs with multicentric cNITCL.

**Disclosures**

No disclosures to report

## ISCAID‐P‐1

### ISCAID—International Society for Companion Animal Infectious Diseases

#### Seroprevalence of toxoplasma‐specific antibodies in European cats suspected of toxoplasmosis

##### D Breu^1^, C Wenk^2^, E Müller^1^

###### ^1^Laboklin GmbH & Co KG, Bad Kissingen, Germany; ^2^Laboklin GmbH & Co KG, Basel, Switzerland

*Toxoplasma gondii* is an obligatory intracellular protozoan parasite. Ingestion of oocyst‐contaminated soil/water or oocyst containing meat poses a risk for human infection. Cats are definitive hosts and responsible for zoonotic excretion of oocysts.

We aimed to evaluate the prevalence of *Toxoplasma*‐infection in domestic cat populations, suspected to have toxoplasmosis. The sera of 6131 cats were analysed for antibodies (IgG/IgM) against *Toxoplasma gondii* by means of ELISA. The samples were collected during 2021 and 2022 from 28 European countries. The median age was 5 years. Sex distribution was 45% males and females equally, 10% being unknown. Results for IgG/IgM titres (NTU) were classified as ‘positive/infection’ when IgG^+^ >55 and/or IgM^+^ >11, ‘borderline’ when IgG^+−^ 50–55 and/or IgM^+−^ 9–11, and ‘negative’/no infection when IgG^−^ <50 and /or IgM^−^ <9.

Among the 6131 cats, IgG (IgM)‐Abs were detected in 35.3% (18.5%), while 64.4% (75%) were negative, 0.3% (6.5%) borderline, 15.6% dual seropositive and 61.8% dual seronegative. Cats positive to IgG (IgM)‐Abs had a median age of 8 (7) years (*P* > 0.05).

A total of 5213 samples (85%) came from seven European countries: Germany (3195) > Romania (681) > Austria (397) > Belgium (293) > Czechia (244) > UK (228) and Switzerland (175). Among them, single Ab‐positivity was variable as follows.

IgG^+^: ~26% (UK, Belgium) ~35% (Germany, Romania) and ~43% (Austria, Czechia, Switzerland). IgM^+^: 16% (Belgium, Romania), 18% (Germany, UK) and 22% (Switzerland, Czechia, Austria). Borderline IgG‐Abs (IgG^+−^) were rarely detected (0–<2%), whereas borderline IgM^+−^‐Abs were found in all seven countries and up to 5‐times more often (~9%) in Switzerland.

Dual seropositivity (IgG^+^/IgM^+^) was most frequently found in cats from Austria, Czechia and Switzerland (19%), unlike 13% in cats from Belgium and UK. Positive results for IgG^+^/IgM^−^ (IgG^−^/IgM^+^) were most frequent in cats from Austria 21% (3%) and Czechia 19% (4%), unlike Romania 13% (2%) and the UK 11% (5%).

Our study suggested that positivity to IgM and/or IgG‐Ab varied widely among the European feline populations. The highest positivity for IgG and/or IgM was found in cats from central European countries (Austria, Czechia, Switzerland), all lying in a temperate zone. Positivity to IgG‐Abs generally indicates the status of (chronic) infection in the absence of oocyst‐shedding and clinical symptoms. Elevated titres of IgM‐Abs may suggest recent exposure and (active) infection with potential oocyst‐shedding. Since oocyst‐shedding poses a high zoonotic risk, the cases of borderline IgM‐results should be rechecked after 2–4 weeks.

**Disclosures**

The authors are employed at the Laboklin GmbH & Co.KG Bad Kissingen, Germany (D.Breu) and Laboklin GmbH & Co.KG Basel, Switzerland (C. Wenk). E. Müller is owner/manager of the Lboklin GmbH & Co KG Bad Kissinegn, Germany.

## ISCAID‐P‐2

### ISCAID—International Society for Companion Animal Infectious Diseases

#### Serum cardiac troponin I as an indicator of myocarditis in dogs diagnosed with leptospirosis

##### K Khor, L Lau, M Mazlan, M Mohammad Sabri, R Abdullah

###### Universiti Putra Malaysia UPM, Serdang, Malaysia

The extent of cardiac involvement in canine leptospirosis is poorly understood. However, myocarditis that develops in humans with leptospirosis is an infectious and potentially fatal zoonotic disease. In clinical settings, cardiac biomarkers specific for myocardial damage have been used for the detection of myocarditis. This study quantified and compared the severity of myocarditis in dogs with leptospirosis. The study also determined the serum cardiac troponin I (cTnI) concentrations in healthy dogs (fresh cadaver obtained from student practical) and dogs with leptospirosis. Archived hearts in formalin from 7 healthy dogs and 11 dogs diagnosed with leptospirosis were used in the study. All healthy dogs showed normal blood profiles and urinalysis findings and were negative for *Leptospira* antibody and antigen, ehrlichiosis, and heartworm disease. Based on the microscopic agglutination test, dogs with leptospirosis showed *Leptospira* antibody titers ranging from 1:100 to 1:800. The blood (*n* = 6), urine (*n* = 8), and organ (*n* = 4, 2 liver and 2 kidneys) samples were positive for the organism. Eight dogs were infected with *L. interrogens* (3 with serovar Bataviae), 2 with *L. borgpetersenii* serovar Javanica, and 1 with *L. kirschneri* serovar Australis. The mid‐section of the left ventricular free wall, and papillary muscle tissue samples were subjected to histopathological examinations. The inflammation scores in the endocardium, myocardium, and pericardium of the ventricular free wall and papillary muscle tissues were significantly higher (*P* < 0.05) in dogs with leptospirosis than in healthy dogs. Dogs with leptospirosis showed significantly higher (*P* < 0.05) serum cTnI concentrations than healthy dogs. In conclusion, the study showed that the serum cTnI concentration is a useful marker for the detection of myocarditis in dogs.

**Disclosures**

No disclosures to report

**Source of Funding**

This research was funded by Ministry of Higher Education (MOHE), Malaysia under the Funda‐mental Research Grant Scheme (FRGS); FRGS/1/2016/SKK02/UPM/02/1/5524930

## ISCAID‐P‐3

### ISCAID—International Society for Companion Animal Infectious Diseases

#### Dynamics of selected inflammatory markers during treatment of feline infectious peritonitis

##### D Pavlin, A Nemec Svete, L Tršar, M Štrljič, S Koprivec, N Tozon

###### Veterinary faculty Ljubljana, Slovenia, Ljubljana

In human patients with COVID‐19 it has been shown, that dynamic of different inflammatory parameters can serve as a prognostic factor for outcome. These parameters include C‐reactive protein as an acute phase protein and cytokines interleukin (IL)‐6, IL‐1ß and tumor necrosis factor (TNF)‐α.

The aim of our study was to evaluate changes of concentration of serum amyloid A (SAA) as one of the major acute phase proteins in cats, and above‐mentioned cytokines in cats treated for FIP. The rationale was to determine dynamic of these variables during FIP treatment to select possible prognostic factors for FIP therapy. For this purpose, we used leftover blood samples, which were collected for diagnostics and clinical monitoring. In healthy control group, the samples were collected as a part of wellness check‐up during routine procedures.

Blood samples of seven cats, which were treated with drug containing GS‐441524 for 12 weeks and five healthy control cats were included in the study. Five patients had wet and two had dry FIP; their ages ranged from 5 months to 11 years. Serum samples were taken before and 4, 8 and 12 weeks after start of the therapy. All included patients were considered cured, since they completed the 12‐week observation period without relapse. Serum concentrations of SAA, IL‐6, IL‐1ß and TNF‐α were determined using species specific ELISA kits.

TNF‐α significantly increased after therapy (*P* = 0.028) from a median concentration of 77.4 pg/mL (IQR: 46.5–103.5 pg/mL) to 114.2 pg/ml (IQR: 111.0–135.0 pg/mL). Median concentration after therapy was significantly higher compared to the control group (99.0 pg/mL, IQR: 78.7–99.2 pg/mL; *P* = 0.042). SAA and IL‐1ß slightly increased after therapy, from median concentration 50.9 to 54.5pg/mL and 20.5 to 21.8 pg/mL, respectively. IL‐6 decreased from median concentration 61.1 to 53.4 pg/mL. However, the changes in these three variables did not reach statistical significance. Furthermore, there were no significant differences (*P* > 0.05) of the last three variables compared to the control group.

Increase of TNF‐α after successful treatment is similar to human patients with COVID‐19, where it was found that expression of TNF‐α is increased in patients who recovered. In contrast to human patients, therapy of FIP did not elicit any significant change in the concentration of other cytokines. Based on these results we may conclude, that TNF‐α is a possible candidate for a prognostic factor in treatment of cats with FIP.

**Disclosures**

No disclosures to report

**Source of Funding**

Slovenian research agency, project J4‐2546

## ISCAID‐P‐4

### ISCAID—International Society for Companion Animal Infectious Diseases

#### The association between immune‐mediated thrombocytopenia diagnosed by flow cytometry and canine monocytic ehrlichiosis

##### W Boontuboon,

###### Nakhonpathom, Thailand

Flow cytometric assay (FCA)‐based direct antiglobulin test has emerged as a valuable tool in the definitive diagnosis of immune‐mediated thrombocytopenia (IMT) in dogs by detecting platelet‐bound Immunoglobulin G (IgG) on the platelet surface, which is a hallmark of this condition. IMT in dogs is commonly characterized by severe thrombocytopenia (platelet count ≤30,000/μL). However, the association between IMT‐positive dogs diagnosed by FCA and platelet concentrations remains unexplored, particularly in the context of canine monocytic ehrlichiosis, an endemic parasitic disease in Thailand.

This study aims to investigate the potential association between the presence of IMT‐positive dogs using FCA and the magnitude of platelet concentrations, as well as other relevant variables, including *Ehrlichia canis (E. canis)* infection.

EDTA‐whole blood samples were obtained from 87 client‐own thrombocytopenia dogs and 8 healthy dogs. FCA was performed to distinguish between negative and positive for IMT, as a cut‐off value is 10% of IgG‐coated platelet. Hematological and biochemistry profiles were determined. All dogs' blood was tested for *E. canis* antibodies and genomic DNA (PCR). Mann‐Whitney U and Chi‐square test were used to determine the statistical differences between IMT‐negative and IMT‐positive groups. Multivariable logistic regression was used to identify IMT risk factors.

IMT‐positive dogs had a significantly lower median age of 4.5 years (range 2.0–8.5, *P* = 0.04) compared to IMT‐negative dogs with a median age of 7 years (range 4–11). The median concentration of platelet using automate count was significantly lower in IMT‐positive dogs at 16.5 × 10^3^/μL (range 8–49.5, *P <* 0.01) compared to IMT‐negative dogs with a median platelet concentration of 75 × 10^3^/μL (range 31.5–142). *E. canis* positive by PCR were 50% (11/22) and 17.8% (13/73) in IMT‐positive and ‐negative groups, respectively. The factors associated with IMT‐positive using multivariable logistic regression were found a significant association with age (year) (OR = 0.79; 95%CI: 0.67–0.94, *P* = 0.010), platelet automate count (10^3^/μL) (OR = 0.97; 95% CI: 0.96–0.99, *P* = 0.002), and *E. canis* positive by PCR (OR = 3.98; 95%CI: 1.11–14.29, *P* = 0.030).Study results indicate that younger, lower platelet automate count concentrations and a positive *E. canis* by PCR are risk factors for IMT‐positive diagnosed by FCA. Interestingly, dogs with *E. canis* infection were four times more likely to have IMT than dogs without *E. canis* infection. It can aid in diagnosing and treating conditions related to *E. canis* infection and other conditions that increase the risk of IMT development.

**Disclosures**

No disclosures to report

## ISCAID‐P‐5

### ISCAID—International Society for Companion Animal Infectious Diseases

#### Impact of DNA extraction methods and culture‐independent approaches on canine lung mycobiota analysis

##### A Fastrès^1^, C Angebault^2^, B Taminiau^1^, G Daube^1^, F. Botterel^2^, C Clercx^1^

###### ^1^University of Liège, Liège, Belgium; ^2^UR Dynamic UPEC, Hôpitaux Universitaires Henri Mondor, Créteil, France

There is growing evidence, at least in humans, that fungi play a role in lung health preservation and in disease development and progression. Our understanding of fungal implication in such conditions relies on accurate and reproducible data acquisition. One of the critical steps in mycobiota analysis concerns DNA extraction as fungi are protected by complex cell wall that resists to classical lysis protocol. There is also a need to limit biases introduced by contaminant DNA, susceptible to result in a wrong mycobiota representation. This concern is of particular importance in healthy lungs where fungi are rare.

In this study, we compared 2 protocols of DNA extraction and 2 sequencing approaches to analyze the lung mycobiota (LMy) of 8 healthy dogs. DNA from bronchoalveolar lavage fluid samples were extracted using either the DNeasy Blood and Tissue kit with the pre‐treatment for Gram‐positive bacteria preceded by a mechanical lysis on FastPrep‐24 (Protocol A), or the QIAsymphony DSP DNA Midi kit preceded by a mechanical lysis on TissueLyser and an enzymatic lysis (Protocol B). DNA were then analyzed by amplicon sequencing targeting the internal transcribed spacer (ITS) 2. DNA extracted with protocol B were also analyzed by shotgun metagenomics analysis (MetaMIC). Except for the step of DNA extraction, sequencing and data analysis were performed for all samples at the same time and in the same laboratory.

Comparison between extraction protocols using ITS amplicon profiling revealed that β‐diversity was significantly different (*P* = 0.013) with a greater inverse Simpson index in protocol A compared to B (median [interquartile range]: 6.2 [5.4–7.6] *versus* 1.8 [1.6–2.3]; *P* = 0.008, respectively). Only 2 phyla, Ascomycota and Basidiomycota, were found with protocol B *versus* 6 with protocol A. In only 2 samples, a similar predominant genus (*Malassezia*) was identified with the 2 protocols. Shotgun analysis resulted in a small number of fungal DNA fragment identification. It might partly be due to bioinformatics techniques used to process sequences that were designed for human samples. No real correlation was found between ITS amplicon profiling and shotgun results.

In conclusion, the DNA extraction protocol and the techniques used to sequence DNA and process sequences have a great impact on LMy determination. Accordingly, LMy comparison between studies using different extraction and sequencing techniques is not recommended. The use of bioinformatic tools design for dogs is warranted. The rarity of the LMy of healthy dogs may explain the difficulty in obtaining accurate and reproducible data.

**Disclosures**

No disclosures to report

**Source of Funding**

Personal funding

## ISCAID‐P‐6

### ISCAID—International Society for Companion Animal Infectious Diseases

#### Characterisation of clinical bleeding diathesis, and laboratory haemostatic aberrations and survival in 180 dogs (2005–2019) naturally infected with angiostrongylus vasorum

##### AS Thomsen^2^, MP Petersen^3^, JL Willesen^1^, MBT Bach^1^, IN Kieler^1^, AT Kristensen^1^, J Koch^1^, LN Nielsen^1^

###### ^1^ University of Copenhagen, Frederiksberg C, Denmark; ^2^ FAVNA dyrlægehuset Gentofte, Gentofte, Denmark; ^3^ Vejen dyrehospital, Vejen, Denmark;

Bleeding diathesis is a known complication in dogs infected with *Angiostrongylus vasorum (A. vasorum)*. This retrospective study investigated the clinical and laboratory haemostatic differences in *A. vasorum* positive dogs with and without signs of bleeding and the impact of bleeding on survival.

Breed, sex, age, type of clinical bleeding, haematocrit and a range of haemostatic tests, including thromboelastography and derived velocity curves were retrospectively registered from *A. vasorum* positive dogs. All parameters were compared between dogs with and without signs of bleeding using univariate analyses. A binomial and a multinomial regression model were applied to examine specific indicators in the bleeding dogs. *P*‐values were false discovery rate adjusted, and adjusted *P* < 0.05 was considered significant.

One hundred and eighty dogs entered the study, including 65 dogs (36.1%) presenting with bleeding diathesis. Different types of cutaneous and mucosal bleeding were the most common clinical findings. Twenty dogs presented with neurological signs associated with intracranial and intra‐spinal bleeding. One hundred and thirty‐seven dogs had haematological and/or haemostatic laboratory analyses performed. Haematocrit, platelet count_Advia2120i_, thromboelastographic angle, maximum amplitude, global clot strength, maximum rate of thrombin generation and total thrombin generation were decreased, while prothrombin time was prolonged in bleeding dogs. The survival rate of bleeding dogs was lower at hospital discharge and one month after diagnosis than in dogs without signs of bleeding. Several haemostatic aberrations were detected in *A. vasorum* positive dogs with bleeding diathesis. Bleeding was identified as an important negative prognosticator in *A. vasorum* positive dogs.

**Disclosures**

LNNielsen is head of the Veterinary Diagnostic laboratory at University of Copenhagen, where the hemostasis testing is performed. The tests are also offered to clinical practice.

## ISCAID‐P‐8

### ISCAID—International Society for Companion Animal Infectious Diseases

#### Resistance of Leishmania infantum to allopurinol in dogs in Europe—a digital droplet PCR assay for quantifying S‐adenosylmethionine synthetase (METK) gene copy number

##### A Kehl^1^, J Almeida Fonseca^1^, G Baneth^2^, T Di Muccio^3^, E Müller^1^, I Schäfer^1^

###### ^1^Laboklin GmbH & Co. KG, Bad Kissingen, Germany; ^2^Koret School of Veterinary Medicine, The Habrew University, Rehovot, Israel; ^3^Istituto Superiore di Sanità, Department of Infectious Diseases, Unit of Vector, Rome, Italy

*Leishmania* (L.) *infantum* is one of the causative agents of visceral leishmaniasis and domestic dogs are the main reservoir. The drug allopurinol is used for the long‐term maintenance of dogs with canine leishmaniasis or as a first line drug, therefore drug resistance is a major issue in veterinary and human medicine. Copy number (CN)‐variation of the of S‐adenosylmethionine synthetase (*METK*) gene was related to resistance to allopurinol in *Leishmania* strains.

Aim of this study was the validation of a digital droplet polymerase chain reaction (ddPCR) assay to quantify the CN of *METK* of *L. infantum* directly in left‐over blood samples of infected dogs.

Assay specific for *METK* and glyceraldehyde‐3‐phosphate dehydrogenase (*GAPDH*, as reference gene) of *L. infantum* were tested in ddPCR. Specificity was confirmed, cross‐reactivity with canine deoxyribonucleic acid (DNA) was ruled out. Sensitivity was lower compared to the PCR used for diagnosing *L. infantum* which uses kinetoplast minicircle DNA present in several copies per cell. We were able to quantify *METK* reliably if at least 500 pathogens/ml blood were present. Accuracy was confirmed by quantifying *METK*‐CN in the WHO strain MHOM/TN/80/IPT‐1 described as having only one copy of *METK*.

The validated method was used to quantify *METK‐*CN in 42 field cases: *L. infantum* in 13 dogs seemed to have only one copy, 21 had two and 8 exhibited three or more copies of the *METK* gene.

The CN‐variation of the *METK* gene may be indicative for allopurinol resistance in dogs and may present a possible marker for increased resistance.

**Disclosures**

No disclosures to report

## ISCAID‐P‐10

### ISCAID—International Society for Companion Animal Infectious Diseases

#### Prevalence and risk factors for *Mycoplasma* spp. positivity in cat blood donor units from Portugal, Spain and Belgium in 2022: retrospective study on 7573 blood donations from 4121 healthy donor cats

##### E Roels^1^, C Debie^2^, S Giraud^2^, R Ferreira^3^, K Gommeren^2^

###### ^1^University of Liège, Liège, Belgium; ^2^University of Liège, Liège, Belgium; ^3^BSA—Animal Blood Bank, Porto, Portugal

Haemotropic mycoplasmas are epi‐erythrocytic parasitic bacteria that can cause haemolytic anaemia. Prevalence for haemoplasma infection varies geographically among *Mycoplasma* species. Male adult outdoor non‐pedigree cats are at increased risk for infection. Some studies identified an association between haemoplasmas and retroviruses. As *Mycoplasma* spp. can be transmitted via blood transfusion, routine quantitative polymerase chain reaction (qPCR) screening of donor cats is recommended. This retrospective study assessed prevalence and risk factors for *Mycoplasma* spp. positivity in cat donor units from Portugal, Spain, and Belgium. A private blood bank database of cat donations performed in 2022 was reviewed. Studied risk factors for *Mycoplasma* spp. positivity included age, sex, pedigree, blood type, geographic area, season, and retroviral co‐infection (FeLV and FIV). A multiple generalised estimation equation model was used to account for repeated blood donations on a same cat. A total of 7573 blood donations from 4121 privately‐owned mixed breed donor cats from Portugal (*n* = 4034, 97.9%), Spain (*n* = 70, 1.7%), and Belgium (*n* = 17, 0.4%) were studied. Most cats donated blood once (*n* = 1996, 48.4%); the remainder donated twice (*n* = 1099, 26.7%), three (*n* = 725, 17.6%) or four (*n* = 301, 7.3%) times. Two‐hundred and twelve Portuguese cats tested positive at least once for *Mycoplasma* spp. leading to an estimated prevalence of 5.3% (95% CI: 4.6–5.9). The prevalence did not significantly differ between Portuguese regions (*P* = 0.28). Two cats in Spain had positive *Mycoplasma* spp. qPCR, whilst all Belgian cats were negative. The small sample sizes in these countries prevented robust prevalence estimation. Among positive Portuguese cats, 30 cats donated blood >1 time in 2022: 26 cats were negative first then subsequently tested positive, 3 cats were positive on two occasions, and 1 cat was initially positive and subsequently tested negative. Blood units collected from male cats were at higher risk for *Mycoplasma* spp. positivity (OR 1.9, *P* < 0.001). Increased risk was also observed for blood units that tested positive for FeLV either by serology and/or qPCR (OR 2.9, *P* = 0.0018) and for blood donations performed in winter (OR 2.5, *P* < 0.0001). None of the other studied risk factors was associated with *Mycoplasma* spp. positivity. European cat blood donors displayed a low prevalence of *Mycoplasma* spp. with an increased risk in cats affected with FeLV and male cats. The seasonality for *Mycoplasma* spp. positivity, with an increased risk in winter, remains to be elucidated. Positive *Mycoplasma* spp. qPCR results identified in previously negative donors emphasizes the importance of testing on every donation instead of annually.

**Disclosures**

No disclosures to report

## ISCAID‐P‐11

### ISCAID—International Society for Companion Animal Infectious Diseases

#### A multivalent vaccine against Lyme borreliosis applied to dogs using different vaccination schedules: characterization of the humoral immune response

##### C Wilczek^1^, J Wenderlein^2^, S Hiereth^2^, R Straubinger^2^

###### ^1^LMU Munich, Institute of Infectious Diseases and Zoonoses, Munich, Germany; ^2^LMU Munich, Institute for Infectious Diseases and Zoonoses, Munich, Germany

Climate change will cause intensified tick activity and thus an increase in tick‐borne diseases like Lyme borreliosis (LB) caused by spirochaetes from the *Borrelia burgdorferi* sensu lato (*Bb*sl)‐complex. In Europe, the most prevalent genospecies responsible for LB are *B. afzelli* (*Ba*), *B. garinii* (*Bg*), and *B. burgdorferi* sensu stricto (*Bb*ss). While the protection of dogs using tick removal and repellents or acaricides is necessary, the best protection against LB provides an OspA‐based bacterin vaccine blocking the transmission of *Bb*sl organisms. This vaccination is important to dogs’ health. In Germany, bivalent and trivalent whole‐cell lysate LB vaccines for dogs are available. While the addition of a booster vaccination in an equine LB‐vaccination regimen is known to increase antibody levels, no information regarding the most suitable vaccination schedule for dogs is available.

To test the effectiveness of the current vaccination scheme, vaccinations were performed with a commercial, multivalent vaccine against Lyme borreliosis, and vaccinated dogs were monitored over an observation period of 13 months. Thereby, according to the owner's choice, two vaccination schedules were used. In V‐basic, dogs were vaccinated on days 0, 21, and 365, while in V‐plus an additional booster vaccination was applied on day 180. Residual blood samples from diagnostic interventions were collected until 390 days after the first vaccination and specific antibody levels against *Borrelia*‐antigens in blood serum samples were measured and characterized using a two‐tiered test including a kinetic ELISA and a line immunoassay.

After an increase in antibody levels directly after the basic immunization, decreasing vaccine‐induced antibody levels were detected after the second injection in both groups. Dogs in V‐plus displayed higher antibody levels after the booster vaccination on day 180 and on day 365 compared to V‐basic. As the basic vaccination and yearly booster are advised to be applied in spring, we assume that dogs in V‐basic might not be protected adequately against LB in autumn, when tick activity and *Bb*sl transmission are high. It is therefore advised to apply an additional booster injection on day 180 when vaccinating dogs against LB.

**Disclosures**

No disclosures to report

## ISCAID‐P‐12

### ISCAID—International Society for Companion Animal Infectious Diseases

#### Leptospira in dogs in Hong Kong: serology and urinary shedding

##### WYJ Tam^1^, O Nekouei^2^, F Rizzo^3^, CL Tsang^3^, YR Choi^4^, M Staples^5^, YM Shuen^6^, F Woodhouse^7^, J Gray^7^, P Beczkowski^1^, CT McDermott^1^, JA Beatty^1^, VR Barrs^3^

###### ^1^Department of Veterinary Clinical Sciences, Jockey Club College of Veterinary Medicine and Life Sciences, City University of Hong Kong, Kowloon Tong, Hong Kong Special Administrative Region of China

^2^Department of Infectious Diseases and Public Health, Jockey Club College of Veterinary Medicine and Life Sciences, City University of Hong Kong, Kowloon Tong, Hong Kong Special Administrative Region of China

^3^Jockey Club College of Veterinary Medicine and Life Sciences, City University of Hong Kong, Kowloon Tong, Hong Kong Special Administrative Region of China

^4^Centre for Animal Health and Welfare, Jockey Club College of Veterinary Medicine and Life Sciences, City University of Hong Kong, Kowloon Tong, Hong Kong Special Administrative Region of China

^5^World Health Organisation Collaborating Centre for Reference and Research on Leptospirosis, at Queensland Health Forensic and Scientific Services, Brisbane, Australia

^6^Pet Cares, Tai Po, Hong Kong Special Administrative Region of China

^7^Society for the Prevention of Cruelty to Animals (Hong Kong), Wan Chai, Hong Kong Special Administrative Region of China

*Leptospira interrogans* causes leptospirosis in dogs and is a zoonotic infection. Vaccination is effective but protection is limited to specific serogroups. This study aimed to determine the prevalence and distribution of *Leptospira* serogroups and prevalence of urinary shedding in dogs in Hong Kong.

Microagglutination testing (MAT) of 22 serogroups was performed on residual diagnostic sera from 284 owned dogs. Titres above 1:100 were considered positive. Urine from 164 dogs undergoing neutering was tested for pathogenic leptospire DNA by *LipL32* quantitative polymerase chain reaction (qPCR). Potential risk factors assessed included signalment, alanine transferase, alkaline phosphatase, bilirubin and creatinine.

Overall seroprevalence was 9.86% (28/284) and 2.38% of tested dogs (5/210) had leptospiruria (median load 9.30 copies/ng of DNA [range 0.34–232], with a median Ct value of 36.36 [range 30.33–37.00]). In total, 53 positive antibody titres were obtained from 28 seropositive dogs, with 35.71% (10/28) seropositive dogs reactive to >1 serogroup (range 2–6). Fourteen distinct serogroups were detected at titres up to 1:3200, most frequently Pyrogenes (16.98%, 9/53), Copenhageni (13.21%, 7/53), Autumnalis (11.32%, 6/53), Cynopteri (11.32%, 6/53) and Ballum (11.32%, 6/53). Antibodies against vaccination serogroups Canicola (5.66%, 3/53), Pomona (3.77%, 2/53) and Grippotyphosa (3.77%, 2/53) were also detected.

Dogs in Hong Kong are exposed to a wide range of *Leptospira* serogroups. Current *Leptospira* vaccines are unlikely protect against the most prevalent serogroups. While prevalence of urinary shedding from dogs is low, gloves should be worn to handle canine urine to prevent zoonotic transmission.

**Disclosures**

No disclosures to report

**Source of Funding**

The study was supported by grants from CityU, HK to VRB (Project No 9380113) and JB (No. 9380111).

## ISCAID‐P‐13

### ISCAID—International Society for Companion Animal Infectious Diseases

#### Leishmania infantum specific IL‐2 production after oclacitinib and ciclosporin treatments in ex vivo stimulated canine blood

##### Dr. Murillo, Ms. Jiménez, Dr. Bardagi, Dr. Solano

###### Universitat Autònoma de Barcelona Bellaterra, Cerdanyola del Vallès, Spain

Canine leishmaniosis (CanL) caused by *Leishmania infantum* (*Li*) is a frequent infectious disease in endemic areas. The immune system of dogs plays an important role in the clinical manifestation of the infection. Th1‐like immune response involves cytokines such as IFN‐γ, IL‐2, and TNF‐α, which are associated with controlling the infection and mild to moderate disease in dogs. Atopic dogs are under treatment with ciclosporin A (CsA) or oclacitinib (Oc) to improve their clinical signs. CsA is also used in systemic immune‐mediated disease in dogs. Both drugs might inhibit T‐cell function. The effect of these drugs on cytokines expression, particularly IL‐2, is crucial to investigate because subclinical infection and clinical illness are common in endemic areas. The present study aimed to assess the effect of Oc and CsA on IL‐2 production in *ex vivo* canine blood stimulated with *Li*. Thirty dogs were recruited based on physical examination, blood work, cytology of lesions, antibody levels against *Li* ELISA and IFN‐γ concentration against *Li* soluble antigen (LSA) in a whole blood stimulation assay (WBA). Dogs were classified as healthy seronegative and non‐IFN‐γ producers (group 1, *n* = 11), healthy seronegative/seropositive and IFN‐γ producers (group 2, *n* = 9), and sick dogs with leishmaniosis and IFN‐γ producers, belonging LeishVet I and IIa stages (group 3, *n* = 10). WBA was performed with 12 conditions: medium, LSA, concanavalin A (ConA), Oc (168 ng/mL (Oc168) and 337 ng/mL (Oc337), CsA (200 ng/mL), LSA + Oc168, LSA + Oc337, LSA + CsA, ConA + Oc168, ConA + Oc337 and ConA + CsA. Supernatants were harvested after 5 days of incubation for the measurement of canine IL‐2 by ELISA. IL‐2 concentrations in LSA‐stimulated WBA were different only when comparing LSA (mean 3.33, SD+1.44) and CsA (mean 1.51, SD+0) in IFN‐γ producers (groups 2 and 3) (*P* < 0.05). CsA decreased IL‐2 concentration in ConA‐stimulated WBA in all groups (*P* < 0.01). In groups 1 and 2, significant increases were observed when ConA was compared with ConA+Oc337 (*P* < 0.05). In group 1, these significant increases were observed at both concentrations of Oc (*P* < 0.01). In conclusion, CsA decreased IL‐2 production in WBA when stimulated with LSA and ConA in all groups. On the contrary, Oc at 168 and 337 ng/mL did not reduce IL‐2 production.

**Disclosures**

Mar Bardagi has consulted for and/or received lecturing honorarium from Zoetis and Elanco.

**Source of Funding**

This study was partially funded by Zoetis.

## ISCAID‐P‐14

### ISCAID—International Society for Companion Animal Infectious Diseases

#### Evaluation of complementary stem cell therapy in the treatment of kidney disease secondary to canine leishmaniasis

##### M González, R Barrera, B Macías‐García, P Nicolás, JI Cristóbal, A Durán, A García, F Duque, P Ruíz

###### Extremadura University, Cáceres, Spain

Canine leishmaniasis (CanL) causes a massive deposit of immune complexes, which causes a great impact on the kidney through proliferative and inflammatory phenomena. Although there are drugs to treat the parasite, the immunopathogenic context described can persist for months. It is in this period that immunomodulation can be crucial, and therapies such as allogeneic mesenchymal stem cells of adipose origin (aAMSC) could improve the renal recovery of dogs with kidney disease secondary to CanL, the evaluation of this hypothesis thus becomes the objective of this study.

A total of 20 dogs with renal disease and CanL were studied. Of them, 10 dogs formed the group treated with miltefosine and allopurinol (CG), and another 10 dogs in the cellular therapy group (CTG), which received the same treatment together with an intravenous dose of 1–1.3 × 106 aAMSC per kilogram. Complete blood and urine analysis were performed at 0 and 30 days. Renal damage was evaluated by measuring traditional markers such as plasma creatinine (pCr) and urea (pUrea), as well as the urinary protein creatinine ratio (UPC), and others such as plasmatic cystatin C (pCysC), plasmatic symmetric dimethylarginine (pSDMA), and urinary ratio of neutrophil gelatinase‐associated lipocalin (uNGAL/c). In addition, the urinary proteome was analyzed by UHPLC‐MS/MS by ESI‐Q‐TOF and compared by bioinformatic analysis (Qlucore Omics Explorer version 3.6, Lund, Sweden) in 13 of the dogs (CTG, *n* = 7; CG, *n* = 6). CTG = 6).

The results (median, 25th–75th percentiles) did not show statistically significant differences in any of the biomarkers evaluated: pCr (mg/dL; CG = 1.2, 0.8–1.5; CTG = 1, 0.8–2.45), pUrea (mg/dL; CG = 85, 27–134.3; CTG = 46.5, 23.5–39), pCysC (mg/L; CG = 0.22, 0.2–0.28; CTG = 0.25, 0.2–0.37), pSDMA (μg/ dL; CG = 20, 13.75–28.5; CTG = 18.5, 11.5–29.25), uNGAL/c (pg/mg; CG = 83.89, 34.82–234.31; CTG = 32.52, 5.48–92.84), UPC (CG = 4.67, 2.24–4.88; CTG = 2.29, 1.70–5.31). The bioinformatics analysis of the urinary proteome did not find separation between groups, although some individuals showed a certain tendency to differentiation, because of that only 4 variables, *Secreted phosphoprotein 1*, *Peptidase S1 domain‐containing protein*, *WAP four‐disulfide core domain protein 2*, and *Ig‐like domain‐containing protein* were significantly increased in the CTG, some of them related to immune response and the renewal of the extracellular matrix.

In conclusion, the intravenous administration of a single dose of aAMSC in a complementary way of treatment was not enough to show benefits in dogs with renal disease and CanL. According to the findings, future studies could be helpful to assess other administration protocols of treatment.

**Disclosures**

No disclosures to report

## ISCAID‐P‐15

### ISCAID—International Society for Companion Animal Infectious Diseases

#### Advantage of matrix‐assisted laser desorption/ionization time‐of‐flight (MALDI‐TOF) mass spectrometry for bacterial identification in cats with pyothorax

##### N Tangmahakul, P Chanchaithong, S Disatian Surachetpong

###### Faculty of Veterinary Science, Chulalongkorn University, Bangkok, Thailand

Pleural effusion in cats can occur due to several causes, such as feline infectious peritonitis, heart diseases, neoplasms and pyothorax. To confirm evidence of pyothorax or bacterial infection, bacterial culture and antimicrobial susceptibility testing (AST) should be conducted.

Medical data from 173 cats with pleural effusion were retrospectively retrieved. The data collected from medical records included disease history, fluid analysis data, bacterial culture results, and AST results. Bacterial species was identified using Matrix‐Assisted Laser Desorption/Ionization Time‐of‐Flight (MALDI‐TOF) mass spectrometry (Bruker Daltonics, Germany), and antimicrobial susceptibility testing was performed using Vitek® automated AST system based on minimum inhibitory concentration (MIC) determination.

Facultative anaerobic bacteria were cultured from 53 out of 173 cats (30.64%) that included gram‐positive bacteria (26/53 or 49.06%) and gram‐negative bacteria (27/53 or 50.94%). The five most common genera were *Pasteurella* spp. (22.64%), *Staphylococcus* spp. (15.09%), *Enterococcus* spp. (9.43%), *Streptococcus* spp. (7.55%) and *Rhodococcus* spp. (7.55%). Methicillin‐resistant staphylococci were found in 3 of 53 samples (5.66%), which consisted of Methicillin‐resistant *Staphylococcus epidermidis* (3.77%), Methicillin‐resistant *Staphylococcus arlettae* (1.89%). Second‐line antimicrobials were recommended for treatment from the susceptible (S) category that were Amoxicillin/clavulanic acid + Fluoroquinolones (18/53 or 33.96%), Amoxicillin/Clavulanic acid (14/53 or 26.42%), Fluoroquinolone (8/53, 15.09%) and either Amoxicillin/clavulanic acid or Fluoroquinolone (6/53 or 11.32%). However, 7 cats (13.21%) needed broader‐spectrum to last‐resort antimicrobials including Vancomycin, Imipenem, Amikacin and/or Gentamicin for treatment because of multidrug‐resistant bacterial infection.

This study presents common bacterial species associated with feline pleural effusion that are mostly susceptible to available second‐line antimicrobials in small animal medicine. However, treatment using third‐line and last‐resort antimicrobials are essential in case of the infection caused by MDR bacteria that should be selected based on AST results.

**Disclosures**

No disclosures to report

## ISCAID‐P‐16

### ISCAID—International Society for Companion Animal Infectious Diseases

#### The effect of oclacitinib and ciclosporin A on IL‐17a production against Leishmania infantum stimulated canine blood

##### Ms. Jiménez, Dr. Murillo, Dr. Bardagi, Dr. Solano

###### Universitat Autònoma de Barcelona Bellaterra, Cerdanyola del Vallès, Spain

Canine leishmaniosis caused by *Leishmania infantum* (*Li*) is a frequent sandfly‐borne disease. Th1‐like immune response with production of interferon gamma (IFN‐γ) is found in subclinical infection to moderate disease, while more severe disease is characterized by a Th2‐like immune profile. Interleukin‐17a (IL‐17a) is associated with the control of *Li* infection in dogs acting in synergy with IFN‐γ. Canine atopic dermatitis (CAD) is a common skin disease. Oclacitinib (Oc) or ciclosporin‐A (CsA) are treatment options for CAD while CsA is also used in other immune‐mediated diseases. Both drugs might affect the immune response against *Li*. The aim of this study was to investigate and compare the effect of Oc and CsA on IL‐17a production against *Li ex vivo* in dogs. Thirty dogs were enrolled based on physical examination, cytology, antibody levels against *Li* ELISA, and IFN‐γ production against *Li* soluble antigen (LSA) in a whole blood stimulation assay (WBA). Dogs were classified as healthy seronegative and non‐IFN‐γ producers (group 1, *n* = 11), healthy seronegative/seropositive and IFN‐γ producers (group 2, *n* = 9), and sick dogs with leishmaniosis and IFN‐γ producers (group 3, *n* = 10). WBA was performed with twelve conditions: medium, LSA, concanavalin A (ConA), Oc at 168 ng/mL (Oc168) and 337 ng/mL (Oc337), CsA (200 ng/mL), LSA + Oc168, LSA + Oc337, LSA + CsA, ConA + Oc168, ConA + Oc337 and ConA + CsA. Supernatants were harvested after 5 days of incubation for measurement of IL‐17a ELISA. IL‐17a concentrations were similar in LSA + Oc168 and LSA + Oc337 compared with LSA in all groups studied. IL‐17a statistical differences were noted in group 2 comparing LSA + CsA (median 1.2, IQR 19.7 pg/mL) with LSA (596.9, 1434.5 pg/mL), LSA + Oc168 (452.5, 895 pg/mL), and with LSA + Oc337 (387.3, 628.3 pg/mL). In group 3, IL‐17a concentrations were significantly lower in LSA + CsA (0, 0), compared with LSA (133.6, 520.9 pg/mL) and with LSA + Oc337 (59.2, 269.7 pg/mL). CsA decreased statistically IL‐17a concentration in all groups in ConA when compared with the rest of the conditions. In group 1, a significant increase was observed when ConA (24856, 11020 pg/mL) was compared with ConA + Oc168 (37950, 24028 pg/mL). In group 2, this significant increase of IL‐17a was observed at both doses of Oc. In conclusion, CsA decreased IL‐17a production in LSA and ConA while Oc at both doses tested did not reduce or even increased the production of IL‐17a.

**Disclosures**

Mar Bardagi has consulted for and/or received lecturing honorarium from Zoetis and Elanco

**Source of Funding**

This study was partially funded by Zoetis

## ISCAID‐P‐17

### ISCAID—International Society for Companion Animal Infectious Diseases

#### Serum amino acid profile in canine sepsis and its relationship with survival

##### E Gori, F Bartoli, C Di Franco, A Briganti, V Habermaass, V Marchetti

###### University of Pisa, Pisa, Italy

Sepsis, as a life‐threatening multiorgan dysfunction syndrome, may cause alteration of a variety of endogenous substances, such as amino acids (AAs). In fact, metabolomics is an emerging tool for studying pathophysiology, for identifying prognostic biomarkers and new therapeutic targets of septic patients. The aim of this study was to evaluate serum AAs in sepsis compared to healthy dogs, and among sepsis survivors and non‐survivors.

Sepsis was defined as the presence of an infectious focus with fulfillment of systemic inflammatory response syndrome criteria and healthy dogs belonged to the hospital blood‐donor program. Serum L‐Serine (SER), L‐Aspartic acid, L‐Glutamic acid, Glycine, L‐Histidine (HIS), L‐Arginine (ARG), L‐Threonine (THR), L‐ Alanine (ALA), Proline (PRO), L‐Cysteine, L‐Tyrosine (TYR), L‐Valine, L‐Methionine (MET), L‐Lysine, L‐Isoleucine (ILE), L‐Leucine (LEU), L‐ Phenylalanine, Tryptophan (TRP) were analyzed with a high‐performance liquid chromatography amino acid analyzer on leftover serum samples.

A total of 55 dogs were enrolled: 30 dogs with sepsis and 25 healthy controls. Sepsis had a variety of different causes: digestive (*n* = 15), reproductive (*n* = 5), bite wounds/skin abscesses (*n* = 4), nephrourologic (*n* = 3), respiratory (*n* = 2), and oncologic (*n* = 1). Of 30 septic dogs, 18 (60%) died or were euthanized for disease severity prior to discharge, the remaining 12 dogs survived. Most of AAs (SER, HIS, ARG, THR, ALA, PRO, TYR, ILE and TRP) were significantly lower in septic dogs than healthy controls, whereas MET and LEU were higher in septic dogs (all *P* < 0.01). Non survivors had a significantly lower TRP than survivors (22.9 ± 12.2 vs. 36.7 ± 18 nmol/mL, *P* = 0.01). No other differences in AAs between septic and healthy, or septic survivors and non‐survivors were found.

Changes in AAs concentrations during sepsis could be associated with increased muscle protein turnover due to a catabolic state (enriched biosynthesis of the branched‐AAs, such as LEU), oxidation (MET is strictly related to homocysteine), decreased energy supply and organ dysfunction (e.g., decrease in ARG synthesis in the kidneys in septic patients). TRP resulted interestingly lower in in our septic and non‐survivors. TRP is involved in serotonin metabolism, and it is metabolized from intestinal microbiota to uremic toxins, associated with acute kidney injury in septic patients.

Serum AAs profile in dogs with sepsis differs significantly from that in healthy dogs. Some of these serum AAs changes are consistent with humans’ sepsis, but some different alterations are present. Further studies are needed to investigate the relationship between AAs abnormalities and sepsis, especially considering sepsis severity and Sequential Organ Failure Assessment (SOFA) score.

**Disclosures**

No disclosures to report

## ISCAID‐P‐18

### ISCAID—International Society for Companion Animal Infectious Diseases

#### Comparison of Real‐Time PCR and culture of bronchoalveolar lavage fluid in dogs for the detection of Bordetella bronchiseptica infection: a retrospective study of 12 cases

##### N Mateu^1^, A Rodriguez‐Cobos^1^, M Labayru^1^, M Molinero^2^, S Atencia^1^

###### ^1^AniCura VETSIA Veterinary Hospital, Madrid, Spain; ^2^IDEXX Laboratories, Barcelona, Spain

*Bordetella bronchiseptica (Bb)*, a microorganism involved in canine infectious respiratory disease complex, is a relatively common pathogen isolated in young dogs. It can be detected by real‐time PCR or culture from bronchoalveolar lavage fluid (BALF).

The aim of this retrospective study was to compare the efficacy of *Bb* detection by real‐time PCR and BALF culture in a population of dogs from a referral hospital in Spain.

A total of 12 referred dogs diagnosed with *Bb* infection between 2018 and 2022 were included. All patients met the inclusion criteria: positive real‐time PCR or BALF culture result as well as a history of chronic coughing. Clinical pathology, thoracic imaging and bronchoscopy findings were reviewed.

All patients except one had received antibiotic treatment prior to referral. Out of the 12 dogs included in the study, 58% (7/12) were small breed (<5 kg), and 42% (5/12) were ≤1 year old. Leukocytosis with neutrophilia was the most common clinical pathology finding (10/12), followed by a moderate increase (twice to three times the reference value) in alkaline phosphatase activity (3/12). BALF cytology was consistent with a septic inflammatory process with the presence of intracytoplasmic microorganisms in 9 of the 12 dogs.

The most common radiological finding was generalised bronchointerstitial pattern (7/12). Bronchoscopy showed airway mucus hypersecretion in 80% (8/10) of the patients. Tracheal and/or bronchial collapse was detected in 50% (5/10) of the dogs that underwent bronchoscopy. Two of the patients did not have bronchoscopy performed due to their small size.

The real‐time PCR and BALF culture detected *Bb* in 100% (12/12) and 67% (8/12) of the dogs respectively. Co‐infection with *Mycoplasma* spp. was detected in 25% (3/12) of cases. Despite the fact that 11 of the 12 patients in the study had received antibiotics prior to referral, positive culture results were obtained. The only patient who did not receive antibiotics had a negative culture for Bordetella.

The use of real‐time PCR to detect *Bb* in BALF samples appeared to be more sensitive than culture in this group of dogs.

**Disclosures**

M. Molinero works at IDEXX laboratories Internal Medicine department.

## ISCAID‐P‐19

### ISCAID—International Society for Companion Animal Infectious Diseases

#### Co‐infections and risk factors in stray cats in Gran Canaria (Spain): Dirofilaria immitis, Bartonella henselae, feline leukemia and feline immunodeficiency

##### SN García Rodríguez^1^, M González González^2^, JI Matos^1^, N Costa‐Rodríguez^1^, E Carretón Gómez^1^, JA Montoya‐Alonso^1^

###### ^1^Research Institute of Biomedical and Health Sciences (IUIBS), Arucas, Spain; ^2^Faculty of Veterinary Medicine, University of Las Palmas de Gran Canaria (Spain), Arucas, Spain

The abandonment and uncontrolled breeding of cats in the urban and rural areas of the island of Gran Canaria (Spain) have been heavily involved in the stray overpopulation of this feline species. This has led to the possibility of concurrent co‐infections with certain feline pathogens. *Dirofilaria immitis* and *Bartonella henselae* are two zoonotic pathogens that have a global impact on the cat. In addition, feline immunodeficiency virus (FIV) and feline leukemia virus (FeLV) are immunosuppressive viruses.

The aim was to determine the prevalence of *D. immitis*, *B. henselae*, FIV and FeLV in stray cats from Gran Canaria.

A total of 65 blood samples from stray cats were collected between September 2020 and July 2021. Sera were analyzed for detection of antibodies against *D. immitis*, specific antibodies against FIV, antigens of FeLV, and molecular detection (PCR) of *B. henselae*.

Antibodies against *D. immitis* and against FIV were found in 16 (24.6%) and 2 (3.1%) of the cats, respectively. Antigens of FeLV were found in 15 (23.1%) of them. Also, DNA of *B. henselae* was detected in 2 (3.1%) cats.

Moreover, 5 (7.7%) were positive of anti‐*D. immitis* antibodies and antigens of FeLV, while 2 (3.1%) developed co‐infection by *D. immitis* and FIV. One cat (1.5%) showed co‐infection by *D. immitis* and *B. henselae*, and none of the cats presented co‐infections by FeLV and FIV. Likewise, *B. henselae* was not present as co‐infection with FeLV or FIV.

This study is the first epidemiological report of these four pathogens in stray cats in Gran Canaria. The seroprevalences and co‐infections of the studied pathogens were similar or higher than those reported in other countries (Thailand, Egypt, Mexico, China, and Greece, among others), whose results vary from 0% to 4.6% for *D. immitis*, from 4.3% to 24.5% for FeLV, from 2.5% to 33.9% for FIV, and from 2.9% to 59.6% for *B. henselae*. Furthermore, the results obtained for *D. immitis* are higher than the general data for domestic cats studied in Gran Canaria (21.3%). Also, molecular detection of *B. henselae* has been studied in fleas infesting cats in Tenerife (Canary Islands, Spain) (2.3%). These results showed that the lack of prevention of infectious diseases and the uncontrolled overpopulation of feral cats poses a risk for the development and growth of infectious diseases in feral and domestic cats, as well as present a greater risk of transmission to humans and other animals.

**Disclosures**

No disclosures to report

## SCH‐P‐1

### SCH—Society of Comparative Hepatology

#### A retrospective study investigating the grade and temporal progression of feline gall bladder sludge and association with concurrent disease

##### G Cattaneo, B Guy, PJ Watson, KE McCallum

###### The Queen's Veterinary School Hospital, Cambridge, UK

There is limited and conflicting evidence on the significance of gall bladder sludge (GBS) in cats and its association with concurrent disease.

This retrospective study aimed to determine presenting signs, clinicopathological findings, concurrent diseases, grade, and progression of feline GBS; it is the first study to investigate grade and temporal progression in cats.

The electronic database of all cats that underwent abdominal ultrasonography between 2016 and 2021 in one institution was searched; 191 cases with an ultrasonographic diagnosis of GBS were selected. Case files were examined for clinical details at the time of documentation of GBS. A semi‐objective ultrasonographic scoring system of GBS severity was adapted from previous canine studies; grade 1: GBS occupying up to 25% of gall bladder volume; grade 2: 26%–50%; grade 3: 51%–75% and grade 4 over 75%. Cases with follow‐up ultrasonographic data were categorized according to timescales (<4 weeks, 1–6 months, 6–12 months, >12 months) and progression of sludge (regressive, progressive, recurrent, or resolved). Results are presented as number or percentage of cats for categorical variables and median [range] with reference intervals for biochemical data.

In the present study, 28% of cats displayed acute (<2 weeks) and 49% chronic (>2 weeks) gastrointestinal symptoms prior to the documentation of GBS which included hyporexia, anorexia, vomiting and/or diarrhoea. The rest were asymptomatic or displayed non‐gastrointestinal signs. In total, 30.2% of cats with GBS had neoplastic disease (non‐hepatobiliary), whilst 20.1% had gastrointestinal disease, 14% urogenital disease and 11.2% hepatobiliary disease (including primary or metastatic neoplasia). 21% (*n* = 41) of cases possessed follow‐up ultrasonographic data; sludge resolved in 43.9%, remained static in 17.1%, progressed in 19.5%, regressed in 9.8% and recurred in 9.8% of cases. GBS was assessed as grade 1 (61.6%, *n* = 117), followed by grade 2 (30%, *n* = 57), grade 3 (6.3%, *n* = 12) and grade 4 (2.1%, *n* = 4). The median values for ALT (50 IU/L [12–1278], ref. 17–62), ALP (28 IU/L [7–1278], ref. 10–93) and total bilirubin (2.7 μmol/L [1.7–486], ref. 0–11) in all cats with biochemistry results at the time of GBS documentation remained within the reference intervals.

This study is the first to document grade and progression of GBS in cats. Sludge was predominantly low grade with variable progression. Neoplasia was the most common concurrent disease process and the presence of GBS was not associated with elevated median hepatobiliary markers.

**Disclosures**

No disclosures to report

## SCH‐P‐2

### SCH—Society of Comparative Hepatology

#### Biliary hamartoma: a case series in small animals

##### I Jolly‐Frahija^1^, F Valls‐Sanchez^1^, D Murgia^1^, R Hattersley^1^, A Ribas‐Latre^2^, P Nelissen^3^, J Aguiar^1^

###### ^1^Dick White Refferals Six Mile Bottom, Cambridgeshire, UK; ^2^Hospital Veterinario de Referencia Veterios, Madrid, Spain; ^3^Frontier Veterinary Specialists, Hergolding, Germany

Biliary duct hamartomas are well‐recognized in human medicine. Often known as ‘von Meyenberg complexes’, they are benign bile duct malformations, frequently incidentally diagnosed during abdominal imaging or laparotomy. In veterinary medicine, these congenital hepatic cystic disorders of the liver remain poorly described.

The aim of this case series was to describe clinical presentation, common diagnostic findings, histopathologic results and outcomes of biliary hamartoma in three dogs and one cat.

The main presenting clinical signs were vomiting (*n* = 2/3 dogs), diarrhoea (*n* = 2/3 dogs and the cat) and weight loss (in the cat). Physical examination findings included cachexia and a palpable cranial abdominal mass in the cat. In all dogs, no physical examination abnormality was detected. Haematology was unremarkable and biochemistry showed moderate to severe increase in liver enzymes activities, with all cases having increased alanine aminotransferase activity. Abdominal ultrasonographic examination revealed a large cystic mass in the left hepatic lobe in all dogs. Enlargement of the left liver lobes and a mixed heterogenous echogenicity within those lobes was observed in the cat. Abdominal computed tomography of two dogs confirmed the large, very well‐defined but irregularly marginated mass in the left division of the liver. The mass appeared to comprise a complex, polycystic structure with numerous internal septa. Histopathologic examination of the liver mass from the three dogs was consistent with the presence of biliary proliferation, which was well‐demarcated and with the absence of atypia or mitotic activity. The presence of hepatocytes within the stroma of the lesion was indicative of a developmental abnormality, consistent with a Von Meyenberg complex. Histopathologic examination of tru‐cut biopsy of the left liver lobe of the cat revealed a markedly disrupted hepatic architecture by the presence of numerous small ducts, most consistent with bile ducts. The epithelial cells lining these ducts exhibited again a relatively bland histological appearance and low mitotic activity consistent with biliary hamartoma. Liver lobectomy was the treatment of choice for all three dogs but was not performed in the cat due to its extensive nature. One dog was alive 12 months after the diagnosis. Two dogs and the cat had favorable outcomes at 3–4 months from the diagnosis, but their follow‐up was lost thereafter.

In conclusion, biliary hamartoma should be considered a differential diagnosis in patients with hepatic cystic masses and gastrointestinal signs. This diagnosis was associated with a good prognosis in all cases described.

**Disclosures**

No disclosures to report

